# Evolution of the rodent *Trim5* cluster is marked by divergent paralogous expansions and independent acquisitions of *TrimCyp* fusions

**DOI:** 10.1038/s41598-019-47720-5

**Published:** 2019-08-02

**Authors:** Guney Boso, Esther Shaffer, Qingping Liu, Kathryn Cavanna, Alicia Buckler-White, Christine A. Kozak

**Affiliations:** Laboratory of Molecular Microbiology, National Institute of Allergy and Infectious Diseases, Bethesda, Maryland USA

**Keywords:** Molecular evolution, Restriction factors

## Abstract

Evolution of cellular innate immune genes in response to viral threats represents a rich area of study for understanding complex events that shape mammalian genomes. One of these genes, *TRIM5*, is a retroviral restriction factor that mediates a post-entry block to infection. Previous studies on the genomic cluster that contains *TRIM5* identified different patterns of gene amplification and the independent birth of *CypA* gene fusions in various primate species. However, the evolution of *Trim5* in the largest order of mammals, Rodentia, remains poorly characterized. Here, we present an expansive phylogenetic and genomic analysis of the *Trim5* cluster in rodents. Our findings reveal substantial evolutionary changes including gene amplifications, rearrangements, loss and fusion. We describe the first independent evolution of *TrimCyp* fusion genes in rodents. We show that the *TrimCyp* gene found in some *Peromyscus* species was acquired about 2 million years ago. When ectopically expressed, the *P. maniculatus* TRIMCyp shows anti-retroviral activity that is reversed by cyclosporine, but it does not activate Nf-κB or AP-1 promoters, unlike the primate TRIMCyps. These results describe a complex pattern of differential gene amplification in the *Trim5* cluster of rodents and identify the first functional *TrimCyp* fusion gene outside of primates and tree shrews.

## Introduction

Throughout millions of years of evolutionary history, retroviruses have adapted to living in concert with their hosts. This co-evolution has led to the co-option of cellular proteins used to aid viral replication as well as the emergence of innate immune factors produced by the host to antagonize viral infection. Over the past several decades, studies on retroviral replication have identified various genes that act to restrict infection by a variety of retroviruses^1–3^. One of the most well studied retroviral restriction factors is encoded by the gene *TRIM5*, a member of the Tripartite Motif (TRIM) gene family^4,5^. The TRIM family of genes produces proteins with three main domains: RING (R), B-Box (B) and Coiled Coil (CC) with a variable fourth domain^6,7^. Some members of this family, including *TRIM5*, encode a fourth domain called B30.2 or SPRY^7^. The TRIM family of genes has been shown to have a variety of functions in the cell, including innate immunity, autophagy, apoptosis and cellular differentiation^5,7–10^.

While the retroviral restriction by the longest isoform of *TRIM5*, *TRIM5α*, was originally discovered in rhesus macaques^4^, later studies found TRIM5α mediated inhibition of various retroviruses to be widespread among mammals^5,11–14^. Several studies on the mechanism of retroviral restriction by TRIM5 suggest that the incoming retroviral capsid lattice is recognized by the SPRY domain of TRIM5 following multimerization^4,5,15–17^. This leads to the proteasome dependent degradation of the viral capsid before reverse transcription can be completed^16–18^. In addition to its function as a direct retroviral antagonist, it has also been shown that TRIM5 can act as a pattern recognition receptor of the retroviral capsid structure and activate innate immune signaling through interactions with NF-κB and AP-1^19^.

Not long after the initial discovery of TRIM5 as a retroviral restriction factor, it was demonstrated that the restriction of HIV-1 infection in owl monkey cells is caused by the expression of an unusual fusion protein made up of the N-terminal R-B-CC domains of TRIM5 and a cyclophilin A (CypA) domain at the C-terminus^20,21^. Further analysis of the owl monkey genome sequence revealed the presence of a retrotransposed, intronless *CypA* gene between exons 7 and 8 of the *TRIM5* gene in this species^20^. The resulting fusion protein, called TRIMCyp, was shown to be a potent inhibitor of HIV-1 with the CypA domain replacing the capsid binding function of the SPRY domain found in TRIM5^20,21^. A few years after this discovery, an independently evolved *TRIMCyp* fusion gene, with a distinct retroviral restriction capacity was identified in some species of the genus *Macaca*^22–26^. This convergently evolved macaque *TRIMCyp* results from insertion of a retrotransposed *CypA* gene downstream of the *TRIM5* exon 8^22–24^. A third, more ancient but currently inactive *TRIMCyp* fusion gene was identified in Old World monkeys and gibbons, called *TRIMCypA3*^27^. More recently, the independent genesis of a *TrimCyp* fusion gene was discovered in the genome of *Tupaia belangeri* (Northern tree shrew)^28^. This *TrimCyp* contains an early stop codon in the retrotransposed *CypA* domain and does not have an antagonistic effect on any of the exogenous retroviruses tested^28^.

Previous studies on the cluster of *Trim* genes containing *Trim5* in various mammals identified differential expansions of the *Trim* genes, likely generated via gene duplication^11,29^. The two species of rodents that were examined, mouse and rat, were found to have at least 8 and 3 copies of the genes belonging to the *Trim5* clade, respectively. In this study, we set out to produce a more comprehensive analysis of the evolution of the *Trim5* locus in the order Rodentia. Our genomic and phylogenetic analyses identified the presence of lineage specific gene duplications, losses and a variety of genomic organizational structures in the rodent *Trim5* cluster. Our findings also revealed the independent evolution of a *TrimCyp* fusion gene in at least two rodent lineages. We confirmed the presence of *TrimCyp* in both RNA and DNA of multiple species belonging to the genus *Peromyscus* indicating a minimum insertion time of approximately 2 million years, and showed that *CypA* was acquired by *Peromyscus* species through retrotransposition into the *Trim5* locus^30,31^. Our findings also show that the TRIMCyp of *P. maniculatus* has antiviral activity against HIV-1 in a dose-dependent manner when ectopically expressed, but unlike the primate *TRIMCyp* fusion genes, it does not activate NF-κB or AP-1 promoters, genes involved in innate immune responses. Collectively, these results document a complex history of genomic remodeling of the rodent *Trim5* cluster and produce evidence of the independent genesis of *TrimCyp* fusion genes in the order Rodentia, all of which may reflect evolving responses to novel retroviral challenges.

## Results

### Genomic organization of the rodent *Trim5* locus

**Figure 1:**
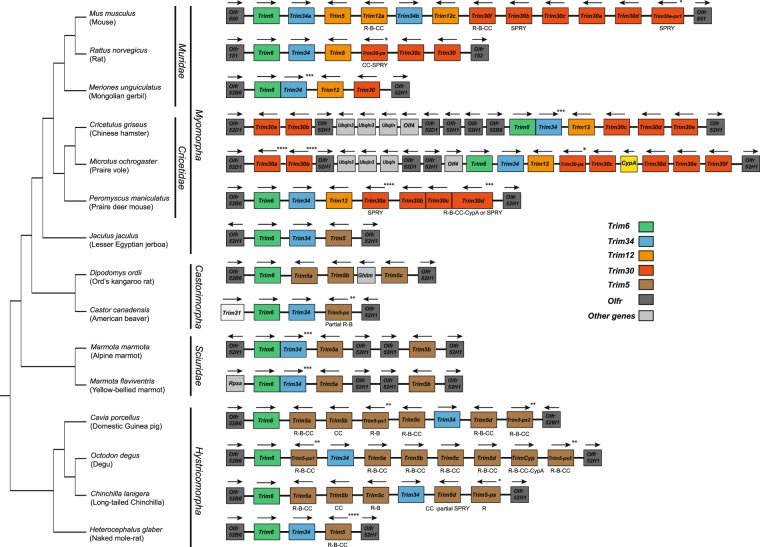
Genomic organization of the *Trim5* locus in rodent genomes. Cladogram indicating the evolutionary relationship between the rodent species is shown to the left. The species tree was generated via TimeTree^45^. The color-coding scheme for the different *Trim* gene clades is indicated in the figure. The *Trim31* gene in the American beaver genome is indicated with a white box. The *CypA* gene found in the *M. ochrogaster* genome is indicated with a yellow box. The predicted domain architecture of the *Trim* genes that encode proteins that differ from the wild type R-B-CC-SPRY are indicated below each box. Arrows indicate transcriptional direction. R; Ring, B: B-Box, CC; Coiled coil, CypA; Cyclophilin A. Olfr; Olfactory receptor, Olf; Olfactomedin, Ubqln; Ubiquilin, Ghitm; growth hormone-inducible transmembrane protein. Rpsa; ribosomal protein SA. *; Annotated as a pseudogene, **; Not annotated, likely pseudogene, ***; Annotated as part of the same gene or RNA, ****; Located next to a sequencing gap. Schematic is not drawn to scale.

**Figure 2:**
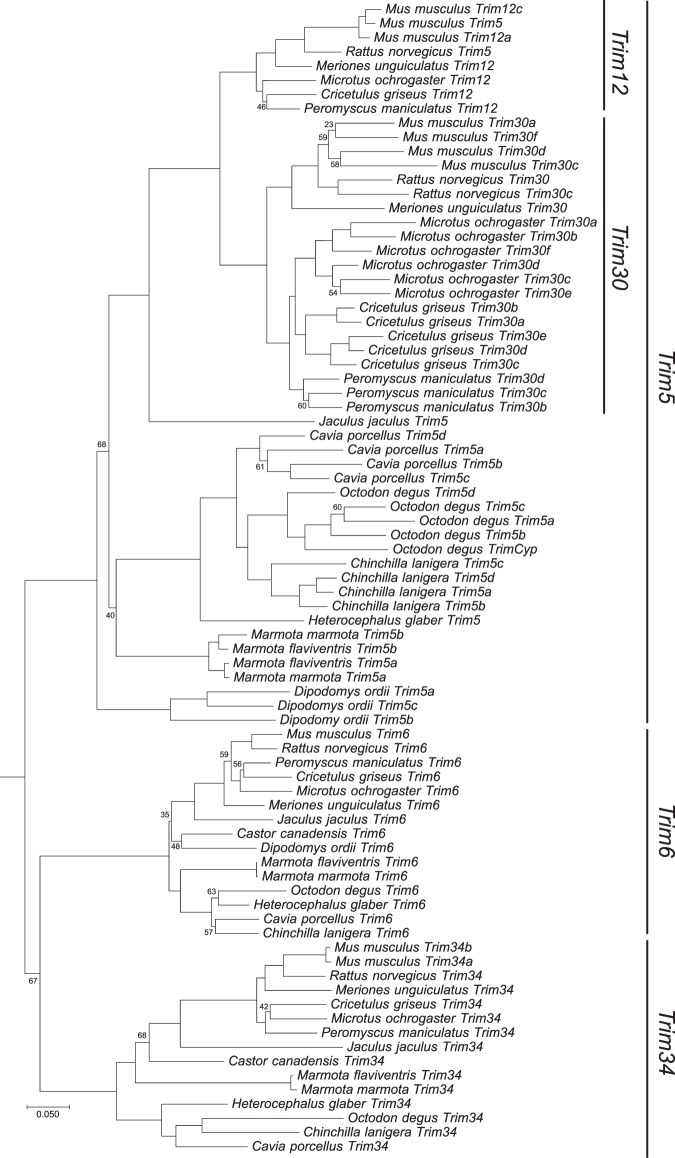
Phylogeny of the rodent *Trim5*, *Trim6* and *Trim34* R-B-CC domains. Open reading frames (ORFs) coding for the R-B-CC domains of annotated *Trim* clade of genes in the *Trim5* cluster of selected rodents were aligned and maximum likelihood trees were generated using RAxML with 500 replicates. Bootstrap values that are below 70 are shown at the relevant nodes. The tree was rooted with Human *TRIM22*. Phylogenetic grouping of each gene clade is indicated to the right of the tree.

**Figure 3:**
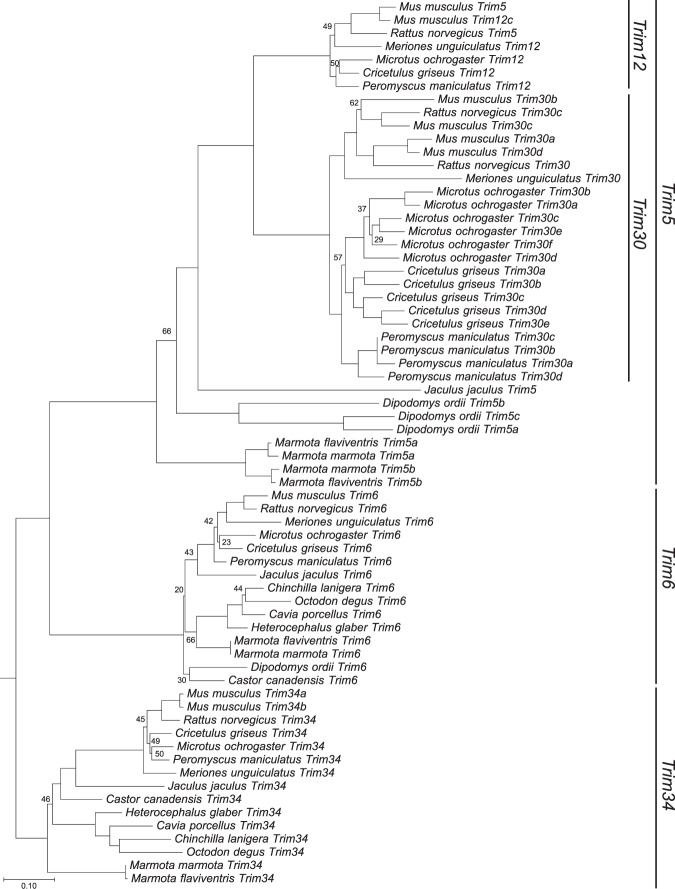
Phylogeny of the rodent *Trim5*, *Trim6* and *Trim34* SPRY domains. ORFs coding for the SPRY domains of annotated *Trim* clade of genes in the *Trim5* cluster of selected rodents were aligned and maximum likelihood trees were generated using RAxML with 500 replicates. Bootstrap values that are below 70 are shown at the relevant nodes. The tree was rooted with Human *Trim22*. Phylogenetic grouping of each gene clade is indicated to the right of the tree.

The *Trim5* cluster of genes in rodents contains members of three distinct *Trim* genes: *Trim6*, *Trim34* and *Trim5*. *Trim5* includes two additional monophyletic groups designated *Trim12* and *Trim30*. A previous description of the genomic organization of this cluster in rodents compared the then available rat and mouse genomes^29^. At the present time, there are 22 assembled and annotated rodent genomes in the NCBI database, containing at least two species from each of four of the five rodent suborders. To produce a more comprehensive picture of the genomic organization in the rodent *Trim5* cluster, we utilized the BLAST tool on the NCBI website^32,33^ to search these 22 annotated genomes for the coding sequences (CDS) of the mouse *Trim12c*, *Trim6* or *Trim34a* genes (Table 1). We excluded seven genomes from our analysis as the *Trim5*-like genes were split between different scaffolds or large sequencing gaps precluded an accurate picture of the genomic organization. Fifteen of the 22 genomes contained *Trim6* and *Trim5*-like genes in the same scaffold or chromosome with no large or problematic sequence gaps. As shown in Fig. 1, 14 of the 15 analyzed genomes contained *Trim6* and *Trim34* genes in this cluster, as was previously described for the reference genomes of mouse and rat^29^. Twelve of the 15 genomes analyzed also contained several copies of *Trim5*-like genes as annotated by NCBI’s gene and mRNA prediction algorithms (Fig. 1 and Supplementary Table S1). *Olfr* (olfactory receptor) genes flank this *Trim* cluster in all species and occasionally are found within this cluster (Fig. 1). To describe the evolutionary relationship between the *Trim* genes in this locus, we generated separate phylogenetic trees using the aligned CDS of the *Trim* genes corresponding to either the R-B-CC (Fig. 2, Supplementary Fig. S1A, Supplementary Data S1 and Supplementary Data S3) or SPRY (Fig. 3, Supplementary Fig. S1B, Supplementary Data S2 and S4) domains. These results revealed the paralogous expansion of genes belonging to the *Trim5* clade in most of the rodent species we analyzed (Fig. 1). We used our phylogenetic analysis and the mouse and rat genome nomenclatures^34^ to name the *Trim5* paralogs in each of the species we analyzed (Fig. 1). Notably, our phylogenetic analysis of the genes belonging to the *Trim5* clade revealed the clustering of the paralogs from the same species rather than orthologs with the same gene designation based on existing nomenclature (Figs 2, 3). This suggests that either similar paralogous expansions occurred in this locus in different lineages or that gene conversion events after speciation homogenized the sequences resulting in species specific clustering of the *Trim5* paralogs. This phenomenon was observed both for newly identified and named *Trim5* genes as well as previously recognized genes such as *Trim30c*.

**Table 1:** Genome assemblies analyzed for *Trim5* orthologs.

Species	Assembly Genbank ID	Assembly Level/ Scaffold N50	Reason for exclusion from analysis
*Mus musculus*	5015798	Chromosome/52,589,046	
*Mus caroli*	4428938	Chromosome/122,627,250	Large gaps in the *Trim5* cluster
*Mus pahari*	4428958	Chromosome/111,406,228	Large gaps in the *Trim5* cluster
*Rattus norvegicus*	1156538	Chromosome/14,986,627	
*Meriones unguiculatus*	4620268	Scaffold/374,687	
*Mesocricetus auratus*	562298	Scaffold/12,753,307	Large gaps in the *Trim5* cluster
*Cricetulus griseus*	7620648	Scaffold/19,581,774	
*Microtus ochrogaster*	504458	Chromosome/17,270,019	
*Peromyscus maniculatus*	869028	Scaffold/3,760,915	
*Nannospalax galili*	1095108	Scaffold/3,618,479	*Trim5 cluster* is split between different scaffolds
*Jaculus jaculus*	406368	Scaffold/22,080,993	
*Dipodomys ordii*	1420568	Scaffold/11,931,245	
*Castor canadensis*	4088328	Scaffold/317,708	
*Ictidomys tridecemlineatus*	317808	Scaffold/8,192,786	*Trim5 cluster* is split between different scaffolds
*Marmota marmota*	2704858	Scaffold/31,640,621	
*Marmota flaviventris*	2033181	Scaffold/17,178,480	
*Fukomys damarensis*	1195548	Scaffold/5,314,287	*Trim5 cluster* is split between different scaffolds
*Heterocephalus glaber*	362148	Scaffold/20,532,749	
*Cavia porcellus*	175118	Scaffold/27,942,054	
*Octodon degus*	375158	Scaffold/12,091,372	
*Chinchilla lanigera*	397218	Scaffold/21,893,125	
*Urocitellus parryii*	7141158	Scaffold/3,964,291	*Trim5 cluster* is split between different scaffolds

The genomic organization of the *Trim5* cluster showed similar patterns for species belonging to the suborders *Myomorpha* and *Scuridae*; in most species, *Trim6* and *Trim34* genes were upstream of at least one copy of a gene belonging to the *Trim5* clade in an antisense orientation relative to *Trim6* and *Trim34* (Fig. 1). Most members of the suborder *Hystricomorpha* differ from other rodents in that there are one or more copies of the *Trim5* clade of genes located between *Trim6* and *Trim34* (Fig. 1). Also, most *Hystricomorpha* species lacked the sequence coding for the SPRY domain in the *Trim5* paralogs found in this locus, with the exception of a sequence coding for a partial SPRY domain found in the genome of chinchilla (Fig. 1). Moreover, in two species in the suborder *Castorimorpha*, we observed a pattern different from the other suborders; *Dipodomys ordii* genome lacks a *Trim34* gene altogether, and the *Castor canadensis* genome only contains a partial/pseudo *Trim5* gene (Fig. 1). It is important to note that in the currently available assembly of *Castor canadensis* this cluster is found in a genomic locus that is not in a region of conserved synteny with the other species. Thus, we cannot rule out the possibility of a mistake in the assembly of this genome.

The previous analysis of the mouse and rat *Trim5* genes revealed the presence of two distinct monophyletic groups in this clade: *Trim12* and *Trim30*^29^. Our results show that this phylogenetic grouping only exists in the species belonging to the families *Cricetidae* and *Muridae* (Fig. 2). Moreover, genomes we analyzed from the species belonging to these two families show a recurring pattern with a similar genomic organization in this locus; single *Trim6* and *Trim34* genes followed by a single gene belonging to the *Trim12* clade. Each species also contains at least one *Trim30* gene while the only species that contains multiple *Trim12* and *Trim34* clade genes is *Mus musculus*. Notably, some members of the family *Cricetidae*, including *Cricetulus griseus*, *Microtus ochrogaster* (Fig. 1) and *Mesocricetus aureus* (data not shown), also contain *Trim30*-like genes upstream of *Trim6* separated by *Olfr* and ubiquilin genes (Fig. 1).

### Evolution of multiple *TrimCyp* fusion genes in rodents

**Figure 4:**
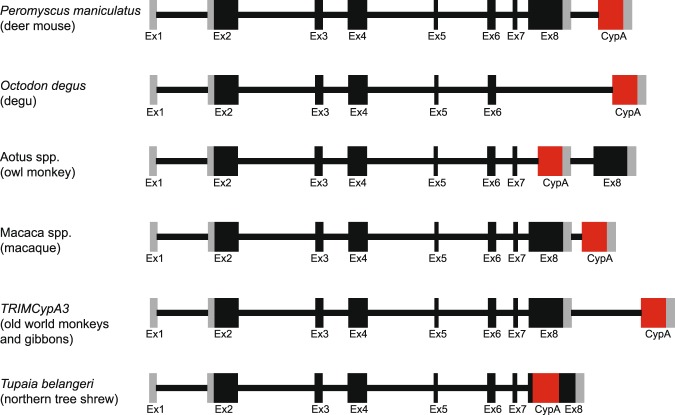
Schematic of the genomic organization of *TrimCyp* genes identified in mammals. Gray regions indicate the 5′ and 3′ untranslated regions. Black boxes indicate CDS. CypA insertions are shown in red. Schematic is not drawn to scale.

Our genomic database analysis of the rodent *Trim5* cluster revealed the presence of an intronless *CypA* sequence in or near a *Trim5* gene in at least three species; *Microtus ochrogaster*, *Peromyscus maniculatus* and *Octodon degus*. In both *P. maniculatus* and *O. degus*, *CypA* is annotated as part of a predicted *TrimCyp* fusion gene/mRNA, while in the genome of *M. ochrogaster*, *CypA* in this locus is not identified in the NCBI annotation and is not clearly located within a *Trim5* gene (Fig. 1). Since each of these *CypA* insertions are in different positions in this locus they are independent insertions. Comparison of the *TrimCyp* fusion genes of *P. maniculatus* and *O. degus* to those previously identified in primates and tree shrews revealed a similar pattern in the location of the *CypA* insertion (Fig. 4). Despite arising independently in distant lineages, these *CypA* insertions are downstream of Exon7 (or Exon 6 in the case of *O. degus*) and upstream, downstream or inside of Exon 8 (Fig. 4). The resulting pattern substitutes the SPRY domain retroviral core binding function in TRIM5 with that of CypA as found in various primate TRIMCyps^15,16^. The *CypA* insertion in *P. maniculatus* is similar to what was previously reported in macaques^22–24^, where the *CypA* sequence is inserted downstream of Exon 8 of a *Trim5*-like gene where it leads to the alternatively spliced product, *TrimCyp* (Fig. 4). Notably, in contrast to what was observed in other lineages, in the *TrimCyp* fusion gene of *O. degus*, exon 7 and exon 8 of *Trim5* are absent and the *CypA* insertion is located about 4 kb downstream of exon 6 (Fig. 4). These multiple independent acquisitions of *TrimCyp* fusion genes in the order Rodentia are the first identified outside the primate and tree shrew lineage.

### Independent genesis of a *TrimCyp* fusion gene in the genus *Peromyscus*

**Figure 5:**
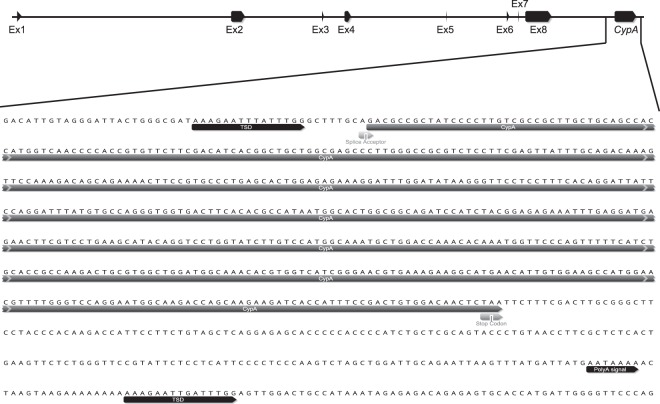
Retrotransposed *CypA* gene in the *Trim5* cluster of *P. maniculatus*. (Upper panel) Schematic of the genomic organization of the *P. maniculatus TrimCyp* gene. (Lower panel) DNA sequence of the retrotransposed *CypA* gene in the *Trim5* cluster of the *P. maniculatus* genome is shown with various features indicated. Figure was created using Geneious^43^. TSD; target site duplication.

To investigate the evolution and function of *TrimCyp* fusion gene in the genus *Peromyscus*, we isolated DNA and RNA from four species belonging to this genus; *P. maniculatus*, *P. polionotus*, *P. leucopus* and *P. californicus*. Genomic DNA sequencing confirmed the presence of *CypA* in the *Trim5* locus in the genome of *P. maniculatus* (Fig. 5). Notably, the sequence of both *CypA* and the intronic region between *CypA* and Exon 8 of *Trim30d* was identical to the sequence in the assembled genome of this species, confirming the accuracy of this sequenced segment in this particular genome assembly. The sequence of *CypA* in the *Trim5* cluster of *P. maniculatus* and the immediate surrounding region is shown in Fig. 5. Similar to what was observed in other lineages^20,22,27,28^, this *CypA* insertion bears the hallmarks of a LINE-1 mediated retrotransposition: It is an intronless copy of *CypA* that is surrounded by target site duplications of 15 bp and contains a 3′ poly A sequence (Fig. 5).

**Figure 6:**
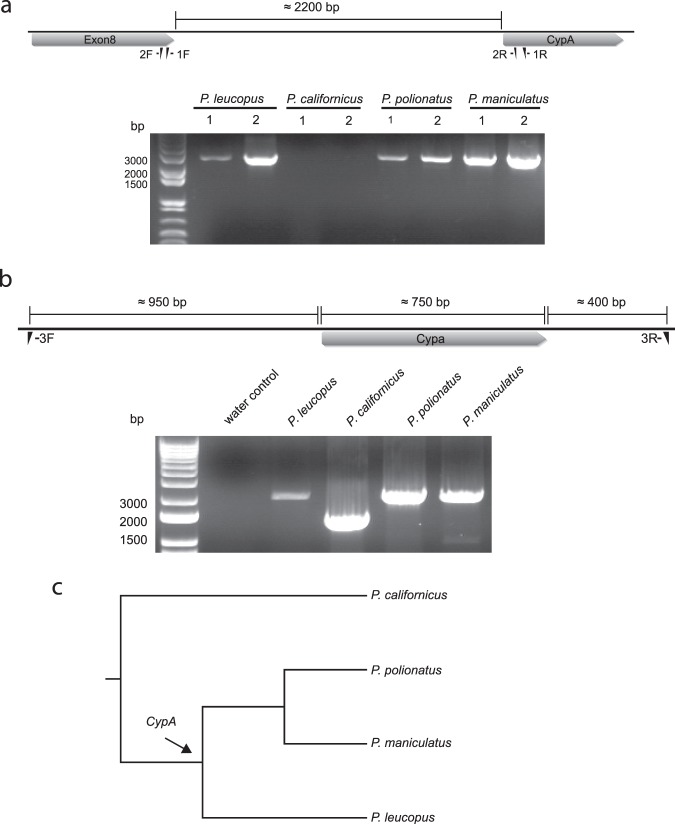
Evolution of *TrimCyp* in the genus *Peromyscus*. (**a**) Upper panel shows the schematic of the region containing the exon8 of *Trim30d* of *P. maniculatus* and *CypA*. Figure was created using Geneious^43^. Primers used in the PCR are denoted. (Lower panel) PCR products were generated using the genomic DNA of the indicated *Peromyscus* genus species with the primer sets 1 or 2 shown in the upper panel. Gel picture is representative of 2 independent experiments. For the uncropped version of the gel picture see Supplementary Fig. 7A. (**b**) Upper panel shows the schematic of the genomic region surrounding the *CypA* in the *Trim5* cluster of *P. maniculatus*. Figure was created using Geneious^43^. Primers used in the PCR are denoted. Approximate length of each of the indicated regions is shown above the figure. (Lower panel) Agarose gel showing the PCR products that were amplified using the genomic DNA of the indicated *Peromyscus* genus species with the primers in the upper panel. Gel picture is representative of 2 independent experiments. For the uncropped version of the gel picture see Supplementary Fig. 7B. (**c**) Cladogram depicting the evolutionary relationship between the *Peromyscus* genus species tested for *CypA* presence. Arrow indicates the suggested insertion point of the *CypA* gene in the *Trim5* locus. The species tree was generated with TimeTree^45^.

To test for the presence of *TrimCyp* in the four *Peromyscus* species in our possession, we designed two sets of primers to amplify the sequence between exon 8 of *Trim30d* and *CypA* (Fig. 6a). This region was successfully amplified in *P. maniculatus*, *P. polionotus* and *P. leucopus*, but no PCR product was obtained for *P. californicus* (Fig. 6a). To confirm the absence of a retrotransposed *CypA* gene in the *Trim5* cluster of *P. californicus*, we designed primers from the intronic region surrounding the inserted *CypA* gene (Fig. 6b). We generated PCR products of ~2100 base pairs (bp) for *P. maniculatus*, *P. polionotus* and *P. leucopus* while *P. californicus* produced a smaller PCR product of ~ 1400 bp consistent with the absence of CypA in this region of the *P. californicus* genome (Fig. 6b). These findings indicate that *P. californicus* lacks this specific *Trim5*-associated retrotransposed *CypA* sequence. The varying PCR band intensities between different species and primer sets are likely due to mismatches between the genomic sequence and the primers, which were designed using the genomic sequence of *P. maniculatus*. However, the sequences of the PCR products are consistent with the conclusion that among the *Peromyscus* species tested, only *P. californicus* lacks the retrotransposed *CypA* sequence in this genomic location. Comparison of the genomic region immediately surrounding *CypA* in *P. californicus* and *P. maniculatus* is shown in Supplementary Fig. S2. Next, we tested the presence of *TrimCyp* and *TrimSPRY* transcriptional isoforms of *Trim30d* in the *Peromyscus* species. Consistent with the results obtained using the genomic DNA, *TrimCyp* cDNA was successfully amplified in *P. maniculatus*, *P. polionotus* and *P. leucopus*, but no PCR product was obtained for *P. californicus* (Supplementary Fig. S3A). Notably, *P. maniculatus*, *P. polionotus* and *P. leucopus* also express a *Trim30d* isoform with a SPRY domain coding sequence at the C-terminal end (Supplementary Fig. S3B). Identity of these isoforms were confirmed by sequencing. In addition, sequencing revealed that the retrotransposed *CypA* gene in this region from both *P. polionotus* and *P. leucopus* contains premature stop codons in *CypA* at different locations (Supplementary Fig. S4). The phylogenetic tree of these four species reveals that *P. maniculatus*, *P. leucopus* and *P. polionotus* are evolutionarily closer to each other than they are to *P. californicus* (Fig. 6c). These results indicate that the *CypA* retrotransposition into the *Trim5* cluster of the genus *Peromyscus* likely happened in the most recent common ancestor of *P. maniculatus*, *P. leucopus* and *P. polionotus*, with a minimum insertion time of approximately 2 million years^30,31^.

### Functional analysis of the *Peromyscus TrimCyp*

**Figure 7:**
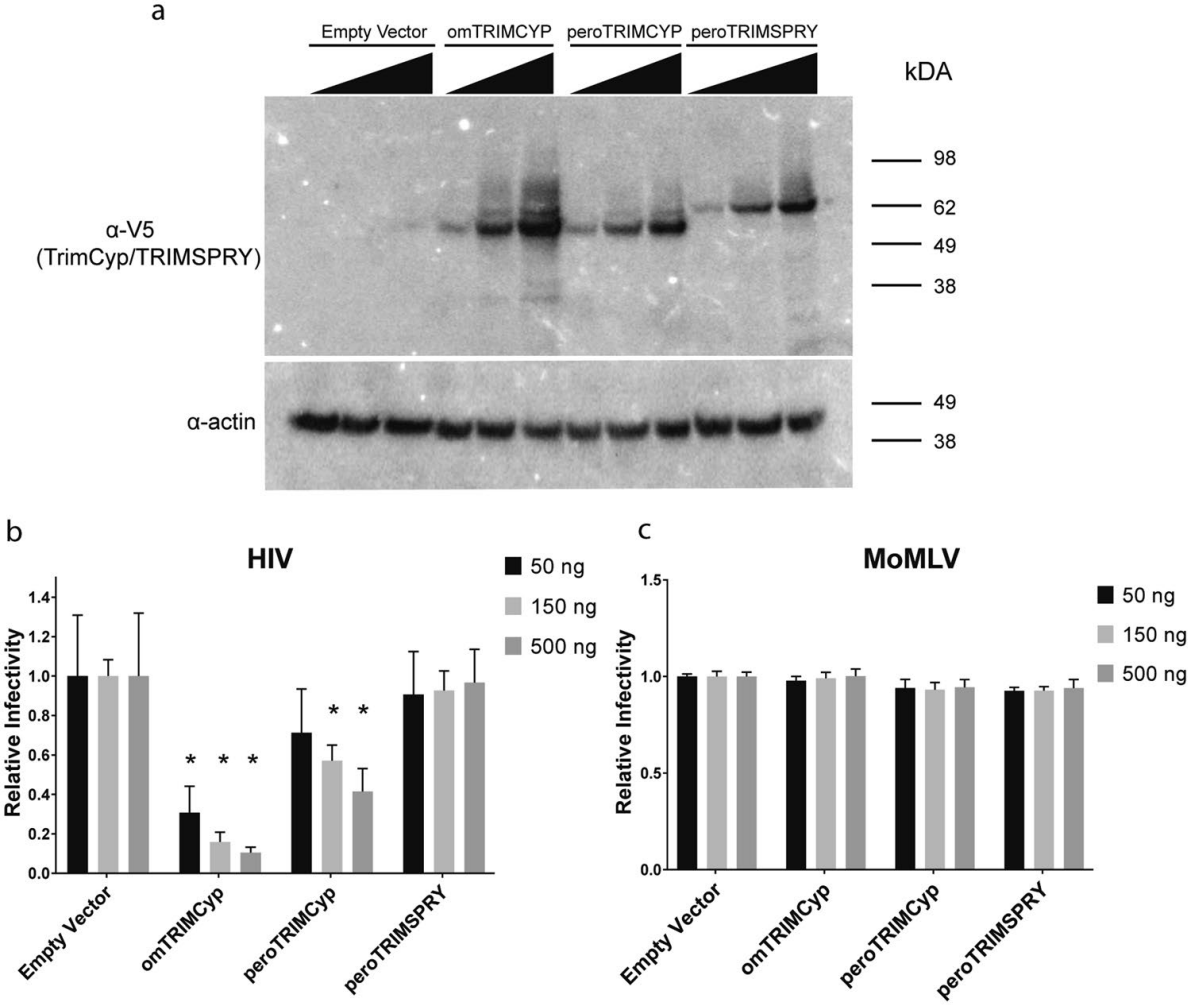
Evaluation of the antiretroviral activity of *P. maniculatus* TRIMCyp and TRIMSPRY. (**a**) Immunoblotting analysis of HEK293T cells transfected with 50, 150 or 500 ng of plasmids expressing the indicated genes was done using the indicated antibodies. For the uncropped version of the immunoblots see Supplementary Fig. S7C,D. Blot is representative of 3 independent experiments. (**b**) and (**c**) HEK293T cells were infected with (**b**) HIV-RenLuc or (**c**) MLV-LacZ 2 days following a transfection with indicated amounts of the plasmids expressing omTRIMCyp, peroTRIMCyp, peroTRIMSPRY or vector only. Normalized (**a**) Renilla luciferase or (**b**) Beta-galactosidase values are shown relative to the empty vector control. Error bars indicate standard deviation. Values represent average of 3 independent experiments. Statistical significance comparing each of the indicated samples individually to the corresponding dose of the empty vector control was estimated with Student’s t-test. *p value < 0.001.

Independently evolved *TrimCyp* fusion genes were discovered in different primate lineages in large part due to their ability to restrict various retroviruses^20,22^. To evaluate the potential retroviral antagonism of the TRIMCyp fusion protein we found in *P. maniculatus*, we generated C-terminal V5-tagged constructs of the *P. maniculatus Trim30d* ORF with either a CypA (peroTRIMCyp) or a SPRY domain (peroTRIMSPRY) at the C-terminus. As a positive control we used a C-terminal V5-tagged owl monkey *TrimCyp* (omTRIMCyp) construct. We ectopically expressed these constructs in HEK293T cells at different dosages (Fig. 7a). omTRIMCyp expression led to a strong inhibition of VSV-G pseudotyped, single cycle HIV-1 (Fig. 7c) as previously reported^19,20^. This inhibition was more prominent with higher doses of omTRIMCyp, while also statistically significant at each dose (Fig. 7b). We observed a more modest and dose-dependent inhibition of HIV-1 by peroTRIMCyp (Fig. 7b). At the highest dose, peroTRIMCyp expression resulted in a statistically significant, 60% inhibition of HIV-1 infectivity (Fig. 7b). Notably this restriction was reversed by the addition of cyclosporine prior to infection, as has been reported for primate TRIMCyps (Supplementary Fig. S5)^20,22–24^. In contrast, we did not observe any inhibition of HIV-1 in cells ectopically expressing peroTRIMSPRY (Fig. 7b). Since peroTRIMCyp and peroTRIMSPRY share the same R-B-CC domains, the difference we observed in HIV-1 restriction by these proteins is likely due to the variation in the capacity of these proteins to interact with HIV-1’s capsid lattice. This possibility is supported by the fact that an alignment of the amino acid sequence of CypA from peroTRIMCyp and omTRIMCyp reveals almost 90% identity, while the sequence of the SPRY domain is less than 40% identical between peroTRIMSPRY and the antiretroviral rhesus TRIM5α (Supplementary Fig. S6). We also tested for antagonism of a rodent retrovirus; Moloney murine leukemia virus (MoMLV), by omTRIMCyp, peroTRIMCyp and peroTRIMSPRY (Fig. 7c). Ectopic expression of different doses of omTRIMCyp, peroTRIMCyp or peroTRIMSPRY did not inhibit infection by VSV-G pseudotyped MoMLV in HEK293 cells. These findings duplicate the known antiviral profile of omTRIMCyp^20^ and indicate that the TRIMCyp fusion protein of *P. maniculatus* has the capacity to antagonize at least one retrovirus, HIV-1.

**Figure 8:**
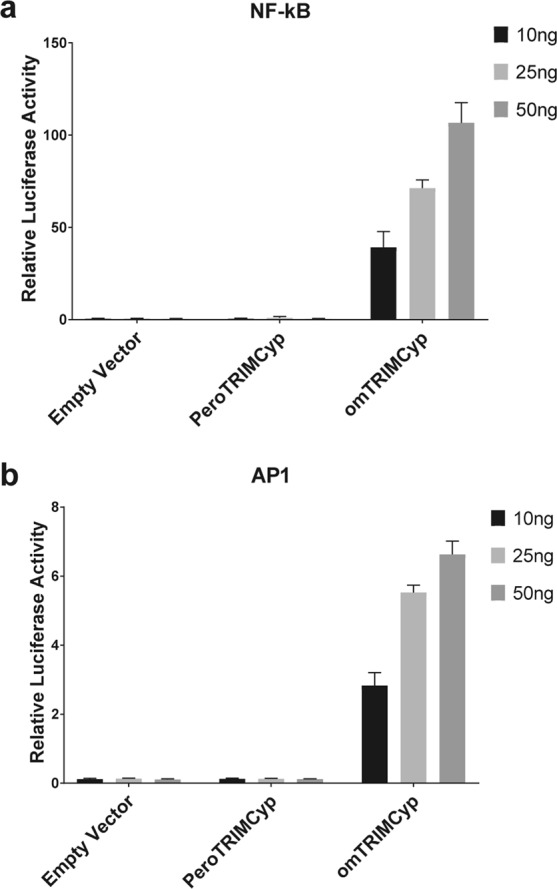
Ectopic expression of peroTRIMCyp does not activate Nf-κB or AP-1 signaling. HEK293T cells were co-transfected with the indicated amounts of omTRIMCyp, peroTRIMCyp or empty vector together with luciferase reporter plasmids containing either (**a**) Nf-κB or (**b**) AP-1 promoters. 20 ng of pRL-TK was added to determine transfection efficiency. Values shown represent firefly luciferase readings normalized to Renilla luciferase. Error bars indicate standard deviation. Values represent average of 3 independent experiments.

Previous studies showed that the TRIMCyp fusion protein of owl monkey can act as a pattern recognition receptor of the incoming retroviral capsid lattice and promote innate immune signaling^19,35^. To investigate the ability of peroTRIMCyp to similarly induce innate immune signaling, we tested the effects of ectopic expression of omTRIMCyp and peroTRIMCyp on transcriptional luciferase reporter constructs with Nf-κB or AP-1 response elements in HEK293T cells. Similar to what was previously observed^19,35^, omTRIMCyp overexpression led to a robust induction of both Nf-κB and AP-1 promoter activities in a dose-dependent manner (Fig. 8a,b). In contrast, ectopic expression of peroTRIMCyp had no effect on either Nf-κB or AP-1 driven luciferase reporter expression (Fig. 8a,b). These results indicate that, unlike omTRIMCyp, overexpression of peroTRIMCyp does not activate Nf-κB or AP-1 promoters.

## Discussion

Our genomic and phylogenetic analyses of the *Trim5* cluster identify two major and recurrent types of genomic alterations of the rodent *Trim5* cluster (Fig. 1). These evolutionary events include differential gene duplications and the acquisition and retention of *CypA* fusions that can have antiretroviral functions. Paralogous expansion of a gene or a gene family can have a variety of effects including increased gene expression levels or gain of a new function^36^. In the case of rodent *Trim5*, such gene duplications have produced *Trim5* loci in different lineages that differ in the number and organization of *Trim* genes. Evolutionary modification of *Trim5* genes also include multiple *TRIMCyp* fusions in primates and also in tree shrews, and here we show that rodents make similar use of the same evolutionary mechanism to produce antiviral genes. This type of convergent evolution in different taxa may reflect responses to similar environmental challenges, especially increases in endogenous or exogenous retroviral activity. The fact that the products of these parallel gene amplification and fusion events are fixed in different lineages supports their having a positive effect on species fitness.

Notably, the *Trim* gene amplification in the *Trim5* cluster of the rodent species we examined is limited to *Trim5*, while *Trim6* and *Trim34* tend to remain single copy genes. *Olfr* genes, the largest known gene family in mammals^37^, flank both sides of the *Trim5* cluster and inserted into the *Trim5* cluster in some species (Fig. 1). In addition, multiple copies of the members of the ubiquilin gene family are found in this locus^38^ (Fig. 1). The paralogous expansion of multiple unrelated gene families in the same genomic locus suggests that recombination driven expansion of one gene family may have facilitated the expansion of the others. Thus, it is possible that in various rodent lineages the *Trim5* clade of genes may have been expanded due to their genomic proximity to the *Olfr* and ubiquilin genes and these duplicated genes may have been fixed in the genome due to selection pressure from unknown retroviruses.

In several members of the family *Cricetidae* we found one or more copies of the genes belonging to the *Trim5* clade far upstream of *Trim6*, separated by multiple copies of olfactory receptors and ubiquilin genes (Fig. 1). Unexpectedly, the paralogous genes from the same species clustered together in the phylogenetic trees rather than with their orthologs (Figs 2, 3). This finding indicates that either these expansions happened in a common ancestor of these species followed by gene conversion events after speciation or that comparable duplications happened independently in various lineages. Our analysis of the genomic assembly of these species also suggests that the duplicated *Trim30* genes that are far upstream remained intact and not pseudogenized throughout the evolution of this lineage. This implies that some manner of selection pressure was exerted on these genes either by exposure to various retrovirus challenges over millions of years or they have retained the cellular function of the original *Trim5* gene.

In this study, we also describe the first chimeric *TrimCyp* fusion genes outside of primates and tree shrews^20,22–24,26,28^. Our genome analyses identified *TrimCyp* fusion genes in three rodent lineages, three species in the genus *Peromyscus*, the species *O. degus* and *M. ochrogaster*. The *CypA* insertion found in the *Trim5* cluster of all of these lineages seem to be the result of a LINE-1 mediated retrotransposition of *CypA* close to the 3′ end of a *Trim5*-like gene (Fig. 5 and data not shown). The *TrimCyp* gene we discovered in the *Trim5* cluster of *M. ochrogaster* differs from what we found in other rodent lineages in that this *CypA* gene is located about 10 kb away from the end of the exon 8 of the *Trim30c* gene of *M. ochrogaster* (Fig. 1). While we do not know if a fusion protein is made, a similar *CypA* retrotransposition was previously identified more than 10 kb away from the *TRIM5* gene in old world monkeys and gibbons^27^. That study identified an alternatively spliced *TrimCyp* in several species^27^. Even though NCBI’s annotation algorithm did not include *CypA* in the *Trim5* cluster of *M. ochrogaster* as part of a *TrimCyp* gene, it is possible that this represents another independent evolution of a *TrimCyp* gene in the order *Rodentia*. In the genus *Peromyscus*, the *CypA* insertion into the *Trim5* locus is found in the same orthologous location in three species, *P. maniculatus*, *P. leucopus* and *P. polionotus*, suggesting that the retrotransposition event happened in an ancient common ancestor. The fact that we were unable to find a *CypA* sequence in this location in the genome of *P. californicus* indicates that either the *CypA* retrotransposition happened only after these species diverged, or that the *CypA* sequence was lost in *P. californicus*.

Previous studies identified at least 5 independent retrotranspositions of *CypA* into the *TRIM5* locus in primates^20,22,27,28^ as well as a pseudogenized TrimCyp fusion gene in fish genomes^39^. Moreover, a preliminary BLAST search of the annotated mammalian genomes housed in the NCBI database using the ORF of *P. maniculatus TrimCyp* as a probe revealed the presence of another *TrimCyp* fusion gene in the species *Sorex araneus* (European shrew) (data not shown). Combined with the three independent *CypA* insertions we found in rodents, these findings suggest that periodic selective pressures likely acted on these species leading to the fixation of the *CypA* sequence in the *Trim5* locus.

Our results also revealed that the ectopic expression of the TRIMCyp fusion protein from *P. maniculatus* had an inhibitory effect on HIV-1, while the splice variant TRIMSPRY containing the same R-B-CC domains had no effect (Fig. 7b). Both CypA and SPRY domains of this TRIM5 variant seem to have been maintained in multiple species in the genus *Peromyscus* for at least 2–5 million years^30,31^. Moreover, the genomic sequence of *P. maniculatus* reveals the presence of several other *Trim5*-like genes in this region (Fig. 1). Since we found that both TRIMCyp and TRIMSPRY transcriptional isoforms are made, we cannot conclude whether one capsid recognizing domain is selected over the other in these species. It is possible that TRIMCyp isoform had a selective advantage over the TRIMSPRY isoform in a common ancestor millions of years ago based on its ability to antagonize unknown, ancient retroviruses, leading to its fixation in this lineage. Moreover, it is possible that TRIMCyp and TRIMSPRY isoforms antagonized different unknown retroviruses throughout the evolution of these species, since both isoforms remain intact.

While none of the laboratory mouse TRIM5-like proteins were previously shown to inhibit infection by any of the exogenous retroviruses tested, one previous study demonstrated that replacing the SPRY domain of mouse TRIM5 paralogs with a primate CypA led to the recognition and inhibition of the incoming HIV-1 by *Trim12* but not by *Trim30* variants^40^. Notably, this retroviral restriction function was correlated with the ability of these *Trim12* and *Trim30* genes to activate the innate immune promoters such as NfκB and AP-1^40^. In contrast to these findings, we found that peroTRIMCyp expression did not activate Nf-κB or AP-1 promoters even though it had an inhibitory effect on HIV-1 infection (Figs 7, 8). It was recently shown that TRIMCyp from northern pig-tailed macaque (npmTRIMCyp), similarly does not activate Nf-κB signaling and only has a minor effect on AP-1 promoter activity^35^. Similar to our results for peroTRIMCyp, npmTRIMCyp was also shown to have a restrictive effect on some retroviruses but not others^35^. These findings suggest that the correlation between the retroviral restriction and the innate immune activation function of TRIMCyp fusion genes is likely more complicated than currently modelled or hypothesized. We also cannot rule out the possibility that peroTRIMCyp may activate other signaling pathways.

While our functional analysis focused on the *TrimCyp* fusion gene found in the genus *Peromyscus*, further analysis of the *TrimCyp* gene we discovered in *O. degus* may shed light on the function and independent evolution of this novel fusion gene. Our BLAST search of the current assembly of *O. degus* in the NCBI database failed to identify any SPRY sequences near any of the *Trim5* paralogs in this cluster (Fig. 1). The absence of *Trim5*-associated SPRY domain was also observed in the genome of *Cavia porcellus* (guinea pig) (Fig. 1). It is important to note that we cannot rule out the possibility that the lack of detection of a *Trim5* encoded SPRY domain in this locus of the genomes of guinea pig and degu may be due to errors in these genome assemblies.

This study provides an extensive genomic and phylogenetic analysis of the paralogous cluster that contains the innate immune antiretroviral gene *Trim5* in rodents. Our results show that this locus has been remodeled through multiple recurrent evolutionary mechanisms in various taxa belonging to the order Rodentia, mechanisms that involved selective gene duplications and conservation of fusions resulting from CypA retrotransposition. We identify, for the first time, the multiple independent evolution of *TrimCyp* fusion genes in the largest order of mammals, Rodentia, at least one of which is antiviral.

## Materials and Methods

### Reagents and plasmids

HEK293T cells were obtained from ATCC (Manassas, VA). GP2-293 packaging cells were purchased from Clontech (Mountain View, CA). The following cells were obtained from Coriell (Camden, NJ); *P. leucopus* skin fibroblasts (Catalog No: AG22618), *P. californicus* skin fibroblasts (Catalog No: AG22620), *P. polionotus* lung fibroblasts (Catalog No: AG22348), *P. maniculatus* kidney cells (Catalog No: AG20716). Each cell line was grown in DMEM (Thermo Fisher, Waltham, MA) supplemented with 10% fetal bovine serum, 1% penicillin and 100 µg/mL streptomycin. Owl monkey prostate tissue was kindly provided by Dr. Boris Skopets (National Institutes of Health, Bethesda, MD). This study was carried out in strict accordance with the recommendations in the Guide for the Care and Use of Laboratory Animals of the National Institutes of Health and the procedure to produce the owl monkey fibroblast culture was in accordance with the guidelines of the Committee of the Care and Use of Laboratory Animals under the NIAID-approved animal study protocol LMM5, which was approved by the Institutional Animal Care and Use Committee (IACUC). All experiments were approved by IACUC and conducted following the relevant guidelines and regulations.

The following plasmids were obtained from Promega (Madison, WI): pRL-TK, pGL4.32[*luc2P*/NF-κB-RE/Hygro], and pGL4.44[*luc2P*/AP1 RE/Hygro]. pEF6 – V5 TOPO and PCR 2.1 TOPO plasmids were purchased from Thermo Fisher (Waltham, MA). The pVSV-G plasmid was obtained from Clontech (Mountain View, CA). pBR HIV NL4.3 *nef*-IRES-*Renilla* Δ*env* plasmid was previously described^41^ and was a kind gift from Dr. Sumit Chanda (SBP Discovery, La Jolla, CA). pCL-MFG-LacZ was obtained from Novus Bio (San Diego, CA). Anti-V5 monoclonal mouse antibody was purchased from Thermo Fisher (Waltham, MA) (Catalog No: R960-25). Anti-Beta-actin rabbit polyclonal antibody was purchased from Sigma-Aldrich (St. Louis, MO) (Catalog No: A5060). Rabbit anti-mouse-HRP and goat anti-rabbit-HRP secondary antibodies were obtained from Southern Biotech (Birmingham, AL).

### PCR and sequencing

PCRs for genomic DNA samples were performed using Amplitaq Gold (Thermo Fisher) with the following program: 95 °C for 3 min; 35 cycles of 95 °C for 30 s, 54 °C for 30 s, and 72 °C for 150 s; and 72 °C for 5 min. Primers used to screen for the presence of *CypA* downstream of a *Trim30d* gene in the genomic DNA of the *Peromyscus* species are shown in Supplementary Table S2. The primers designated 4 F and 4 R were used for sequencing the retrotransposed *CypA* that is part of the *TrimCyp* gene in the genomic DNA of the various *Peromyscus* species (Supplementary Table S2).

Reverse transcription (RT)–PCR for total RNA samples was performed using Superscript III one step RT-PCR kit (Thermo Fisher) with the following program: 55 °C for 30 min; 95 °C for 3 min; 35 cycles of 95 °C for 30 s, 55 °C for 30 s, and 72 °C for 150 s; and 72 °C for 5 min. The primers designated 5 F and 5 R were used to amplify *TrimCyp* cDNA from the *Peromyscus* species (Supplementary Table S2). PCR and RT-PCR products were analyzed by 1% agarose gel electrophoresis and cloned into the PCR 2.1 TOPO (Thermo Fisher) plasmid before sequencing. Gel images were acquired using GENE FLASH (Syngene, Frederick, MD).

### DNA and RNA isolation

Genomic DNA was isolated from the *Peromyscus* cells using PureLink Genomic DNA Mini Kit (Thermo Fisher), according to the manufacturer’s instructions. Total RNA was isolated from *Peromyscus* cells and owl monkey prostate tissue using PureLink RNA Mini Kit (Thermo Fisher), following the manufacturer’s instructions.

### Plasmid construction for cDNA expression

TOPO cloning strategy was used for the construction of the V5-tagged pEF6 expression plasmids. To this end, total RNA from the relevant species was subjected to RT–PCR using Superscript III One Step RT-PCR kit (Thermo Fisher) with the following primers (Supplementary Table S2): for *omTRIMCyp*, 6 F and 6 R; for *peroTrimCyp*, 7 F and 7 R. *peroTrimSPRY* that encodes for the same R-B-CC domain as *peroTrimCyp* was initially amplified using the primers 8 F and 8 R. This PCR product was then used as a template for amplification of the *peroTrimSPRY* CDS using the primers 8 F and 9 R. PCR products were gel extracted using QIAquick gel extraction kit (QIAGEN, Germantown, MD) and cloned into pEF6-V5 TOPO plasmid (Thermo Scientific) following the manufacturer’s instructions. This plasmid contains a C-terminal V5 tag. cDNAs were cloned in frame with the V5 tag. All plasmids were sequenced.

### Genomic database search of the *Trim5* locus in rodent species

CDS of *Trim12c* (NM_001146007), *Trim34a* (NM_030684) or *Trim6* (NM_001013616) from the mouse reference assembly were used as probes in the initial BLAST searches of 22 annotated rodent genome assemblies housed in the NCBI database (Table 1)^32,33^. BLASTn searches were conducted using the following parameters: gap costs, 5 and 2 (existence and extension); match/mismatch scores, + 2/−3; repeat masking filter turned off; expect threshold, 10^−20^. Genomes that showed multiple BLAST hits at different scaffolds/chromosomes as well as those that contained large sequencing gaps in the specific region of the scaffold/chromosome encompassing the *Trim5* cluster were excluded from further analysis. Predicted mRNA from each annotated gene in the predicted *Trim5* cluster that contained one or more BLAST hits was extracted to be used in sequence alignments (Supplementary Table S1).

### Sequence alignment and phylogenetics

Alignments of the CDS regions of the annotated *Trim5*, *Trim6* and *Trim34* clade of genes were done using MUSCLE as implemented in Geneious 10.0.9 using the default settings^42,43^. Manual adjustments were made to the alignments to remove any large indels that appeared in more than a few genes. For each gene, part of the ORF coding for the R-B-CC domains were aligned separately from the part that encodes for the SPRY domains. Predicted pseudogenes were extracted from each relevant genome using either the BLAST aligned regions or annotated RNAs as a guide. Phylogenetic grouping of pseudogenes was determined by aligning them with a subset of *Trim6*, *Trim34* and *Trim5* clade genes. Relevant regions of the CDS of human *TRIM22* (NM_006074) was used in the alignments to root the phylogenetic trees. Maximum likelihood phylogenetic trees were generated using RAxML with the General Time Reversible + G + I model with 500 bootstraps for branch support^44^.

### Immunoblotting

HEK293T cells were grown in a 12 well plate as 200,000 cells/well and transfected with 50, 150 or 500 ng of either of the following plasmids; pEF6 – omTRIMCyp – V5, pEF6 – peroTRIMCyp – V5, pEF6 – peroTRIMSPRY – V5 or pEF6 – empty. Transfections were done using Fugene HD (Promega, Madison, WI), according to the manufacturer’s instructions. 2 days after the transfections, cells were counted, and 500,000 cells were lysed using RIPA buffer (Thermo Fisher) and lysates were sonicated using Q700 sonicator (QSonica, Newtown, CT). Equal amounts of lysates were loaded to a 4–12% Bis Tris polyacrylamide gel, and immunoblotting was performed on a nitrocellulose membrane after the transfer. Images of the immunoblots were produced using ChemiDoc MP imaging system (Bio-Rad, Hercules, CA).

### Virus production and infection assays

For the production of VSV-G pseudotyped, single cycle HIV-1 with Renilla luciferase reporter (HIV-RenLuc), HEK293T cells were seeded at 60% confluency in 60 mm dishes and transfected with 10 ug of pBR HIV NL4.3 *nef*-IRES-*Renilla* Δ*env* and 2 ug of pVSV-G plasmids using Fugene HD. 3 days after transfection, supernatant was collected and filtered through 0.45 µM filters (Millipore, Burlington, MA). For the production of VSV-G pseudotyped, LacZ reporter expressing Moloney murine leukemia virus (MLV-LacZ), GP2-293 cells were seeded at 60% confluency in 60 mm dishes and transfected with 10 ug of pCL-MFG-LacZ and 2 ug of pVSV-G plasmids using Fugene HD. 3 days post-transfection, supernatant was collected and filtered through 0.45 µM filters (Millipore).

For viral infectivity tests, HEK293T cells were transfected with 50, 150 or 500 ng of either one of the following plasmids; pEF6-empty, pEF6-omTRIMCyp, pEF6-peroTRIMCyp, pEF6-peroTRIMSPRY. 2 days later, transfected cells were counted and plated to a 96 well white bottom assay plate (Corning Costar, New York, NY) as 10000 cells/well for infection with 5 uL of filtered supernatant containing either HIV-RenLuc or MLV-LacZ. 5 µM of cyclosporine (Sigma-Aldrich) was added to the relevant wells one hour prior to infection for the infection rescue assay, while the control wells received the equivalent volume of dimethyl sulfoxide. The drugs were removed one day later. Three days post infection Renilla luciferase or LacZ reporter expression was detected using Renilla-Glo (HIV-1) or Beta-Glo (MoMLV) assay systems (Promega), respectively, according to the manufacturer’s instructions.

### Dual-luciferase transfection assay

HEK293T cells were co-transfected in a 12-well plate with 10, 25 or 50 ng of either of the following plasmids pEF6-empty, pEF6-omTRIMCyp, pEF6-peroTRIMCyp together with 100 ng of either pGL4.32[*luc2P*/NF-κB-RE/Hygro] or pGL4.44[*luc2P*/AP1-RE/Hygro] using Fugene HD (Promega), according to the manufacturer’s instructions. 20 ng of pRL-TK was added to each transfection to control for transfection efficiency. 48 hours post transfection, cells were collected, and luciferase reporter activities were measured using Dual-Glo luciferase assay system (Promega).

### Statistical analysis

The unpaired student’s t test was utilized to calculate statistical significance as implemented in GraphPad Prism (version 7.0).

## Supplementary information

Supplementary Information

## Data Availability

All data generated or analyzed during this study are included in this published article and its supplementary information files. The Partial genomic DNA sequence surrounding the retrotransposed *CypA* gene and the complete CDS of the TrimCyp and TrimSPRY isoforms of the *Trim30d* gene from *Peromyscus* species are available in Genbank under accession numbers MK585560 to MK585565 and MN078251 to MN078253

## References

[1] Duggal NK, Emerman M (2012). Evolutionary conflicts between viruses and restriction factors shape immunity. Nat Rev Immunol.

[2] Yan N, Chen ZJ (2012). Intrinsic antiviral immunity. Nat Immunol.

[3] Zheng YH, Jeang KT, Tokunaga K (2012). Host restriction factors in retroviral infection: promises in virus-host interaction. Retrovirology.

[4] Stremlau M (2004). The cytoplasmic body component TRIM5alpha restricts HIV-1 infection in Old World monkeys. Nature.

[5] Yap MW, Nisole S, Lynch C, Stoye JP (2004). Trim5alpha protein restricts both HIV-1 and murine leukemia virus. Proc Natl Acad Sci USA.

[6] Reymond A (2001). The tripartite motif family identifies cell compartments. EMBO J.

[7] Sardiello M, Cairo S, Fontanella B, Ballabio A, Meroni G (2008). Genomic analysis of the TRIM family reveals two groups of genes with distinct evolutionary properties. BMC Evol Biol.

[8] Rajsbaum R, Garcia-Sastre A, Versteeg GA (2014). TRIMmunity: the roles of the TRIM E3-ubiquitin ligase family in innate antiviral immunity. J Mol Biol.

[9] Mandell MA (2014). TRIM proteins regulate autophagy and can target autophagic substrates by direct recognition. Dev Cell.

[10] Uchil PD (2013). TRIM protein-mediated regulation of inflammatory and innate immune signaling and its association with antiretroviral activity. J Virol.

[11] Si Z (2006). Evolution of a cytoplasmic tripartite motif (TRIM) protein in cows that restricts retroviral infection. Proc Natl Acad Sci USA.

[12] Ylinen LM (2006). Isolation of an active Lv1 gene from cattle indicates that tripartite motif protein-mediated innate immunity to retroviral infection is widespread among mammals. J Virol.

[13] Sawyer Sara L., Emerman Michael, Malik Harmit S. (2007). Discordant Evolution of the Adjacent Antiretroviral Genes TRIM22 and TRIM5 in Mammals. PLoS Pathogens.

[14] Schaller T, Hue S, Towers GJ (2007). An active TRIM5 protein in rabbits indicates a common antiviral ancestor for mammalian TRIM5 proteins. J Virol.

[15] Diaz-Griffero F (2006). Requirements for capsid-binding and an effector function in TRIMCyp-mediated restriction of HIV-1. Virology.

[16] Stremlau M (2006). Specific recognition and accelerated uncoating of retroviral capsids by the TRIM5alpha restriction factor. Proc Natl Acad Sci USA.

[17] Ganser-Pornillos BK (2011). Hexagonal assembly of a restricting TRIM5alpha protein. Proc Natl Acad Sci USA.

[18] Lukic Z (2011). TRIM5alpha associates with proteasomal subunits in cells while in complex with HIV-1 virions. Retrovirology.

[19] Pertel T (2011). TRIM5 is an innate immune sensor for the retrovirus capsid lattice. Nature.

[20] Sayah DM, Sokolskaja E, Berthoux L, Luban J (2004). Cyclophilin A retrotransposition into TRIM5 explains owl monkey resistance to HIV-1. Nature.

[21] Nisole S, Lynch C, Stoye JP, Yap MW (2004). A Trim5-cyclophilin A fusion protein found in owl monkey kidney cells can restrict HIV-1. Proc Natl Acad Sci USA.

[22] Wilson SJ (2008). Independent evolution of an antiviral TRIMCyp in rhesus macaques. Proc Natl Acad Sci USA.

[23] Brennan G, Kozyrev Y, Hu SL (2008). TRIMCyp expression in Old World primates Macaca nemestrina and Macaca fascicularis. Proc Natl Acad Sci USA.

[24] Virgen CA, Kratovac Z, Bieniasz PD, Hatziioannou T (2008). Independent genesis of chimeric TRIM5-cyclophilin proteins in two primate species. Proc Natl Acad Sci USA.

[25] Newman RM (2008). Evolution of a TRIM5-CypA splice isoform in old world monkeys. PLoS Pathog.

[26] Liao CH, Kuang YQ, Liu HL, Zheng YT, Su B (2007). A novel fusion gene, TRIM5-Cyclophilin A in the pig-tailed macaque determines its susceptibility to HIV-1 infection. AIDS.

[27] Malfavon-Borja R, Wu LI, Emerman M, Malik HS (2013). Birth, decay, and reconstruction of an ancient TRIMCyp gene fusion in primate genomes. Proc Natl Acad Sci USA.

[28] Mu D (2014). Independent birth of a novel TRIMCyp in Tupaia belangeri with a divergent function from its paralog TRIM5. Mol Biol Evol.

[29] Tareen SU, Sawyer SL, Malik HS, Emerman M (2009). An expanded clade of rodent Trim5 genes. Virology.

[30] Platt RN, Amman BR, Keith MS, Thompson CW, Bradley RD (2015). What Is Peromyscus? Evidence from nuclear and mitochondrial DNA sequences suggests the need for a new classification. J Mammal.

[31] Cornejo-Latorre C, Cortés-Calva P, Álvarez-Castañeda ST (2017). The evolutionary history of the subgenus Haplomylomys (Cricetidae: Peromyscus). Journal of Mammalogy.

[32] Altschul SF, Gish W, Miller W, Myers EW, Lipman DJ (1990). Basic local alignment search tool. J Mol Biol.

[33] Camacho C (2009). BLAST+: architecture and applications. BMC Bioinformatics.

[34] Smith CL (2018). Mouse Genome Database (MGD)-2018: knowledgebase for the laboratory mouse. Nucleic Acids Res.

[35] Zhu JW (2018). Activation of NF-kappaB induced by TRIMCyp showing a discrepancy between owl monkey and northern pig-tailed macaque. Mol Immunol.

[36] Nei M, Rooney AP (2005). Concerted and birth-and-death evolution of multigene families. Annu Rev Genet.

[37] Hayden S (2010). Ecological adaptation determines functional mammalian olfactory subgenomes. Genome Res.

[38] Marin I (2014). The ubiquilin gene family: evolutionary patterns and functional insights. BMC Evol Biol.

[39] Boudinot P (2011). Origin and evolution of TRIM proteins: new insights from the complete TRIM repertoire of zebrafish and pufferfish. PLoS One.

[40] Lascano J, Uchil PD, Mothes W, Luban J (2016). TRIM5 Retroviral Restriction Activity Correlates with the Ability To Induce Innate Immune Signaling. J Virol.

[41] Manganaro L (2015). HIV Vpu Interferes with NF-kappaB Activity but Not with Interferon Regulatory Factor 3. J Virol.

[42] Edgar RC (2004). MUSCLE: multiple sequence alignment with high accuracy and high throughput. Nucleic Acids Res.

[43] Kearse M (2012). Geneious Basic: An integrated and extendable desktop software platform for the organization and analysis of sequence data. Bioinformatics.

[44] Stamatakis A (2014). RAxML version 8: a tool for phylogenetic analysis and post-analysis of large phylogenies. Bioinformatics.

[45] Hedges SB, Marin J, Suleski M, Paymer M, Kumar S (2015). Tree of life reveals clock-like speciation and diversification. Mol Biol Evol.

