# Supplementary material for: Evolution of the rodent Trim5 cluster is marked by divergent paralogous expansions and independent acquisitions of TrimCyp fusions
Source: Sci Rep. 2019 Aug 2;9:11263. doi: 10.1038/s41598-019-47720-5 (PMC6677749; doi:10.1038/s41598-019-47720-5)
Supplement: Supplementary file 1 — Supplementary Information [file 41598_2019_47720_MOESM1_ESM.pdf]

Supplementary Information for

Evolution of the rodent *Trim5* cluster is marked by divergent paralogous expansions and independent acquisitions of *TrimCyp* fusions

Guney Boso, Esther Shaffer, Qingping Liu, Kathryn Cavanna, Alicia Buckler-White, Christine A. Kozak

Supplementary Figures

Supplementary Figure S1. Phylogeny of pseudogenes in the rodent *Trim5* cluster. ORFs coding for **(A)** R-B-CC or **(B)** SPRY domains of a subset of the Trim clade of genes in the rodent *Trim5* cluster were aligned together with the indicated pseudogenes. Maximum likelihood trees were generated using RaxML with 500 replicates. Bootstrap values below 70 are shown at the relevant nodes. Each tree was rooted with Human *TRIM22*.

Supplementary Figure S2. Alignment of the genomic region around *CypA* from *P. californicus* and *P. maniculatus*. Genomic DNA sequence that surrounds the retrotransposed *CypA* gene found in the *Trim5* cluster of the indicated *Peromyscus* genus species was aligned. Dots indicate identical nucleotides to the *P. maniculatus*. Dashes indicate gaps in the alignment. TSD: Target site duplication.

Supplementary Figure S3. Analysis of the *TrimCyp* and *TrimSPRY* transcriptional isoforms of the *Trim30d* gene of the *Peromyscus* genus species. (A) (Upper panel) Schematic of the mRNA of the *TrimCyp* isoform of *Trim30d* of *P. maniculatus* is shown. Primers used for Reverse Transcription (RT) - PCR are denoted. (Lower panel) Agarose gel showing the RT-PCR products that were generated using the total RNA of the indicated *Peromyscus* genus species with the primers shown in the upper panel. Gel picture is representative of 2 independent experiments. **(B)** (Upper panel)

Schematic of the mRNA of the *TrimSPRY* isoform of *Trim30d* of *P. maniculatus* is shown. Primers used for RT - PCR are denoted. (Lower panel) Agarose gel showing the RT-PCR products that were amplified using the total RNA of the indicated *Peromyscus* genus species with the primers shown in the upper panel. Gel picture is representative of 2 independent experiments.

Supplementary Figure S4. Alignment of the *CypA* sequence of the *Peromyscus* genus species. DNA sequence that codes for the CypA domain of the TRIMCyp protein of the indicated *Peromyscus* genus species was aligned. Three letter amino acids are shown below each codon. Dots indicate identical nucleotides/amino acids to the *P. maniculatus*. Locations of the stop codons are annotated.

Supplementary Figure S5. Cyclosporine reverses HIV-1 inhibition by peroTRIMCyp. HEK293T cells were infected with HIV-RenLuc 2 days after a transfection with 500 ng of the plasmids expressing omTRIMCyp, peroTRIMCyp, peroTRIMSPRY or vector only. 1 hour prior to infection, cells were treated with either 5 µM cyclosporine or equivalent volume of dimethyl sulfoxide. Renilla luciferase values are shown. RLU: Relative light units. Error bars indicate standard deviation. Values represent average of 2 independent experiments.

Supplementary Figure S6. CypA domain of *P. maniculatus* TRIMCyp shows strong similarity to the CypA domain of *Aoutus nancymae*. Amino acid alignments

comparing **(A)** CypA domains of *P. maniculatus* and Aotus nancymae (owl monkey) TRIMCyp and **(B)** SPRY domains of *P. maniculatus* TRIM30D and *Macaca mulatta* (rhesus macaque) TRIM5 are shown. Dots indicate identical amino acids to the *P. maniculatus* sequence.

Supplementary Figure S7. Uncropped gels and blots. A. Uncropped version of the gel picture shown in Figure 6A. **B.** Uncropped version of the gel picture shown in Figure 6B **C.** Uncropped version of the V5 antibody immunoblot shown in Figure 7A **D.** Uncropped version of the actin antibody immunoblot shown in Figure 7A.

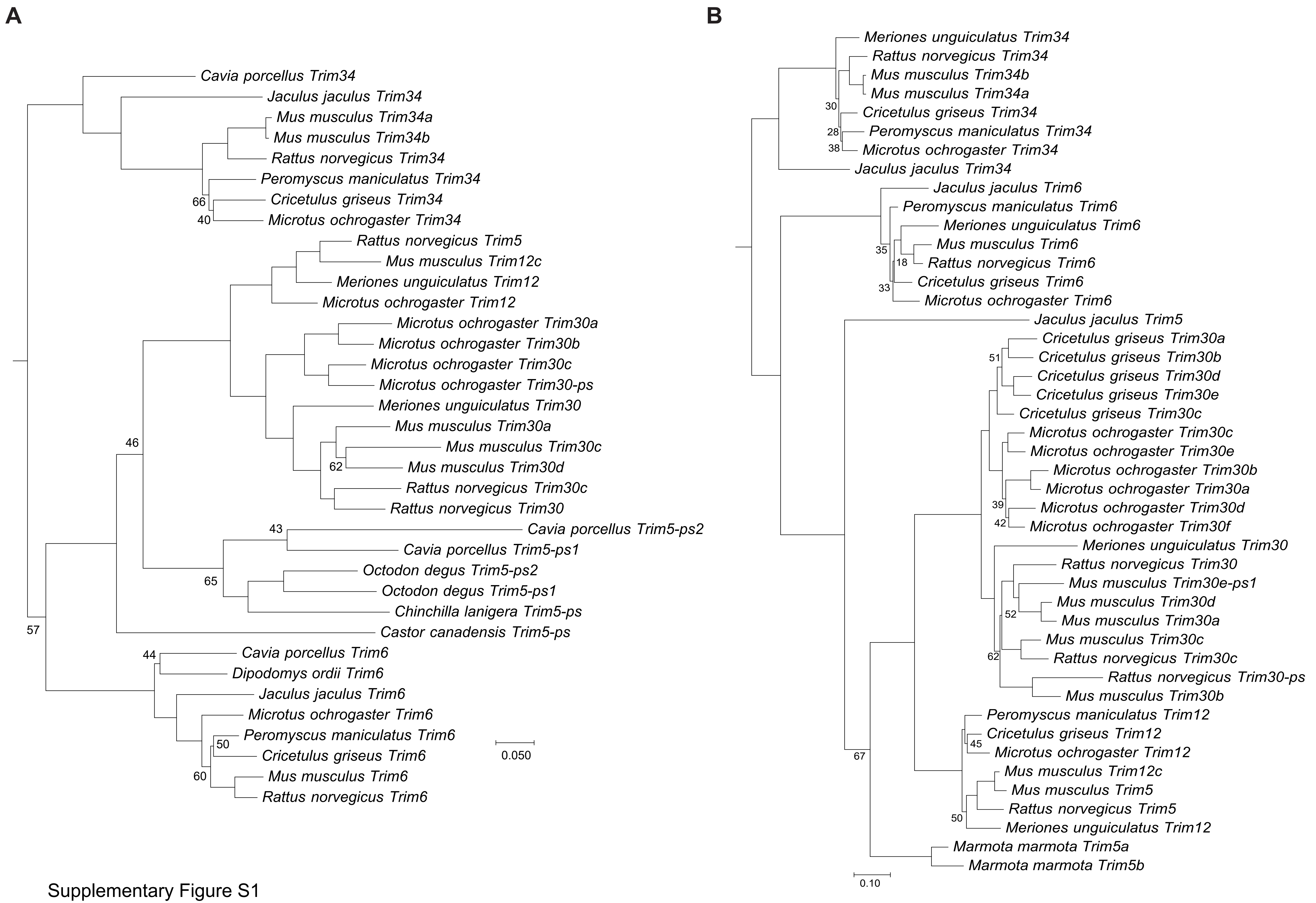

Supplementary Figure S1

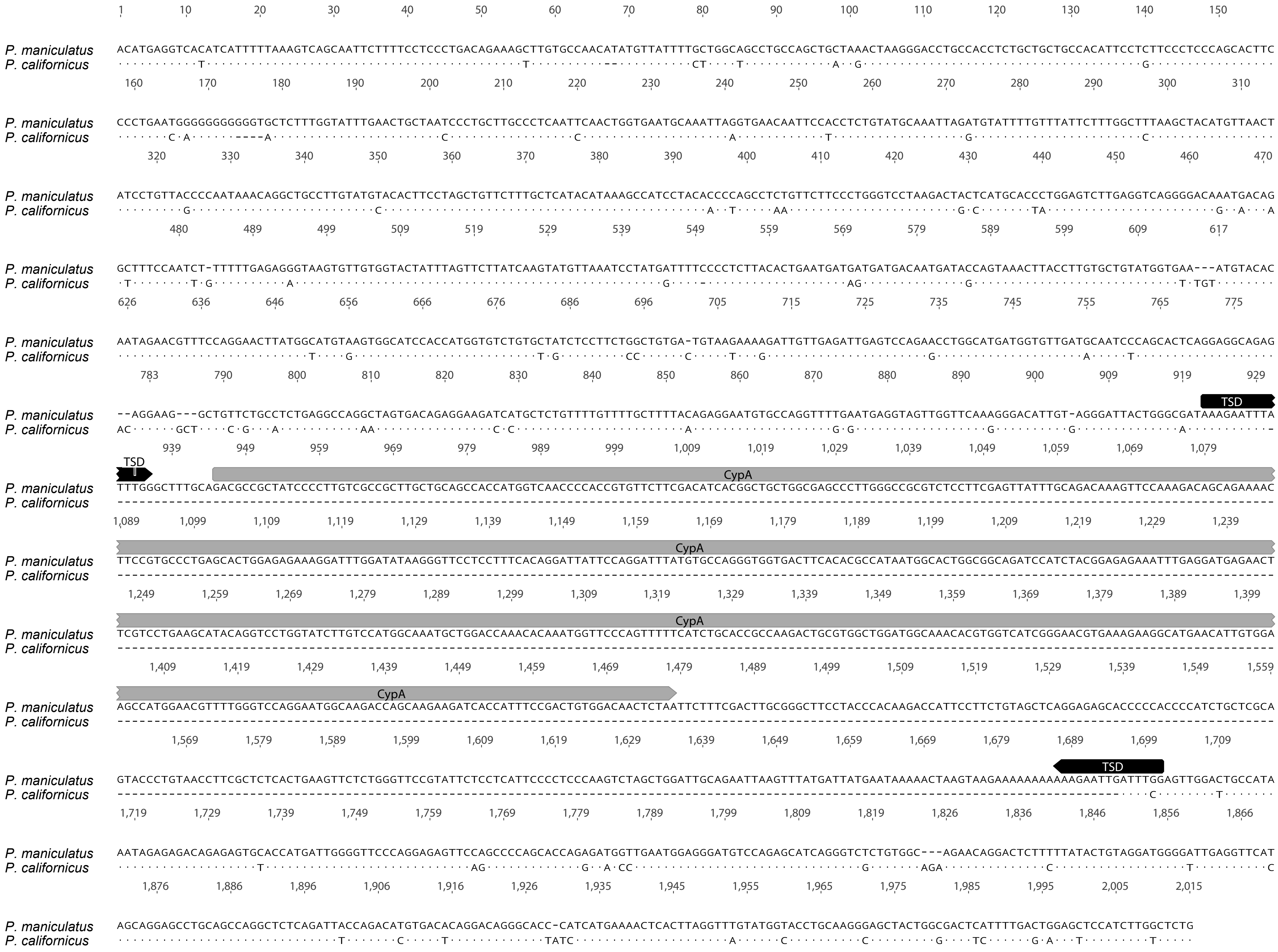

Supplementary Figure S2

Supplementary Figure S3

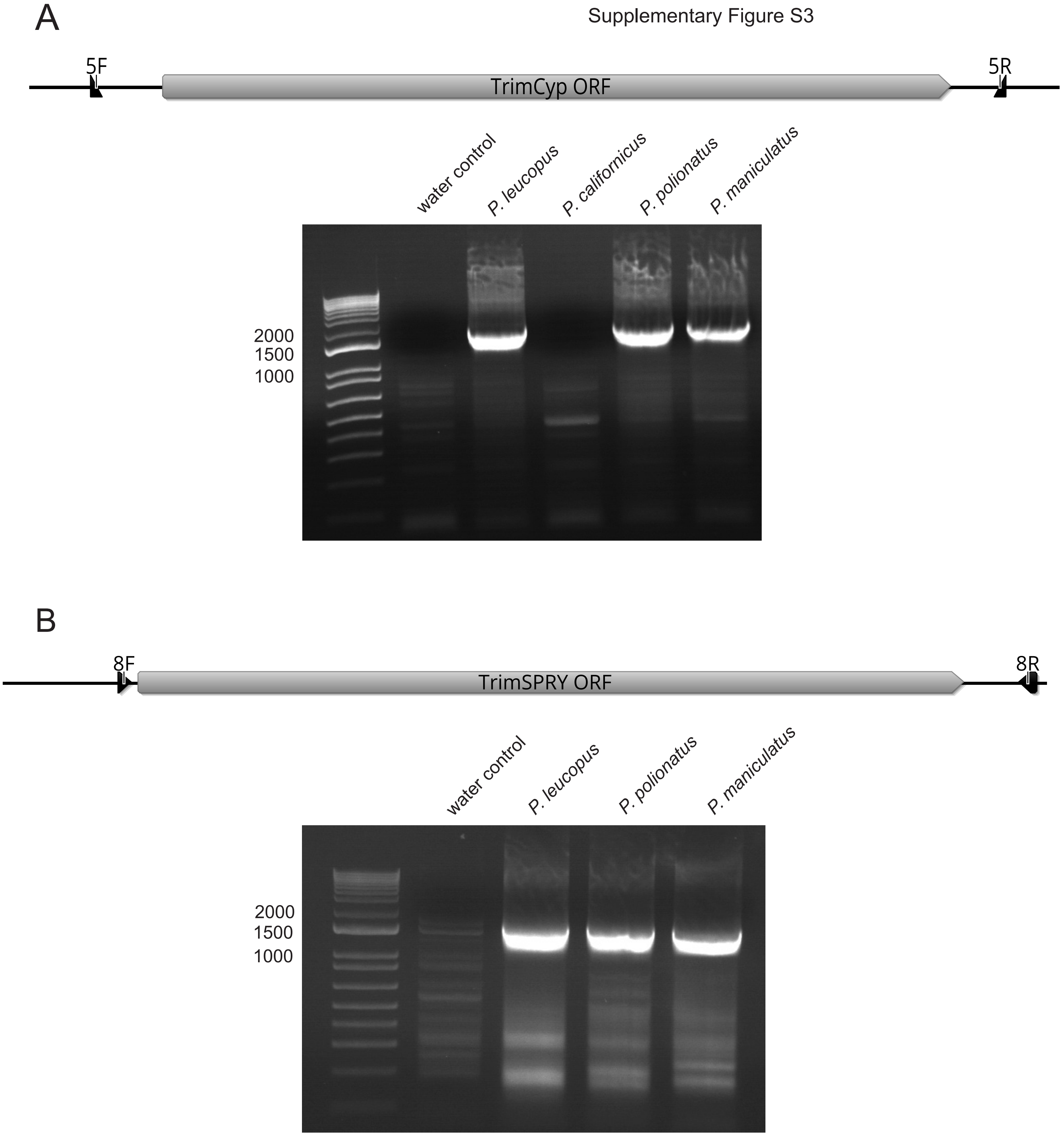

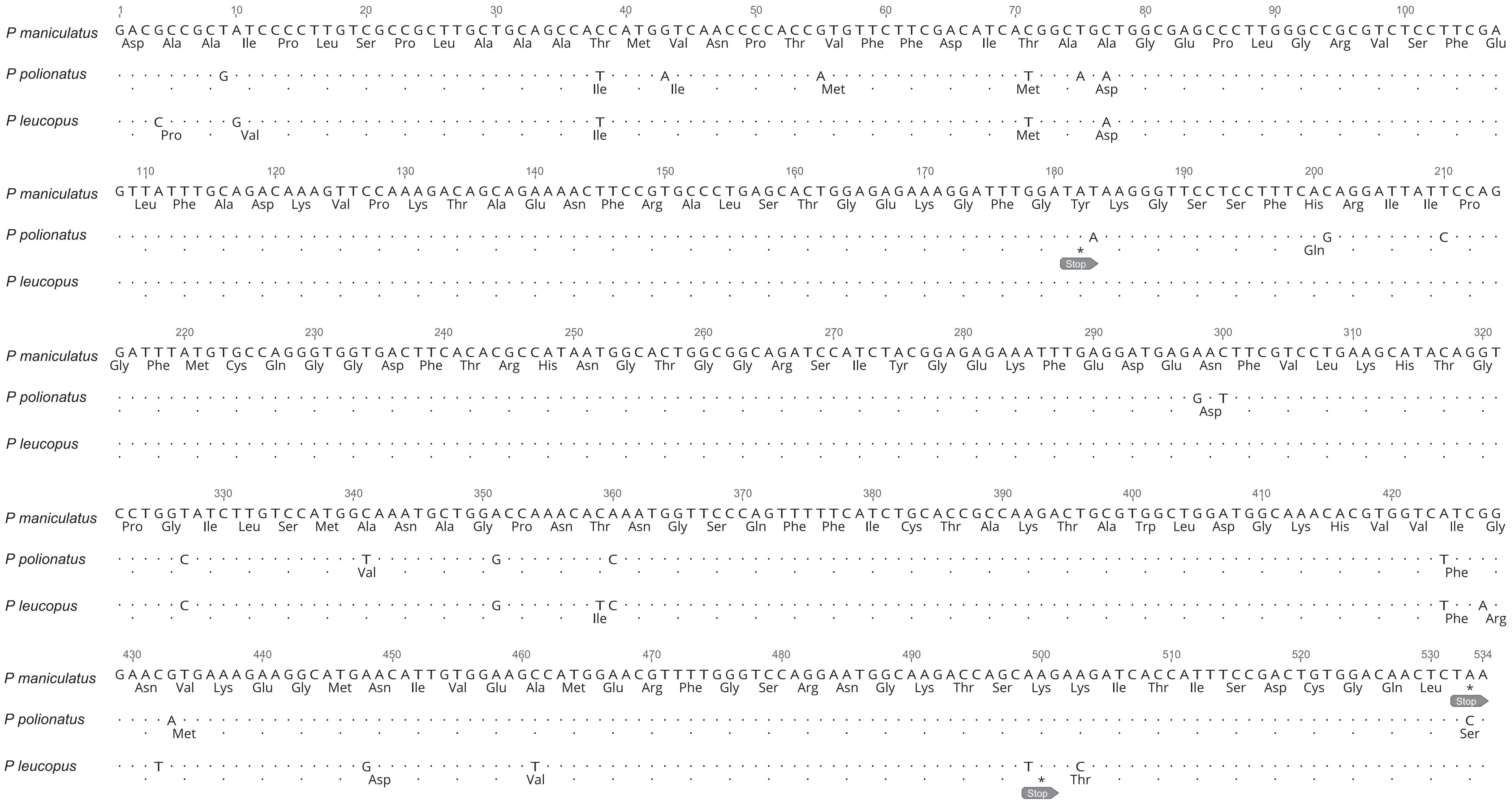

Supplementary Figure S4

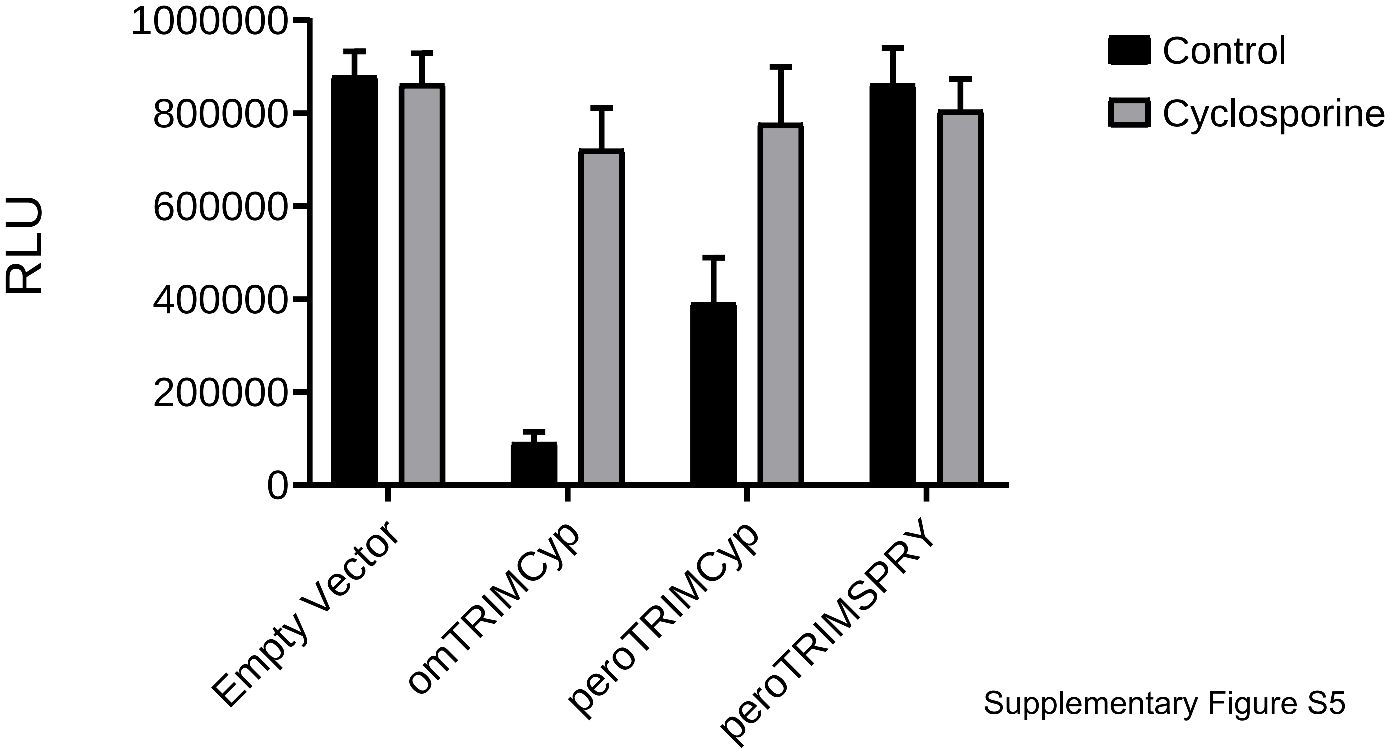

Supplementary Figure S5

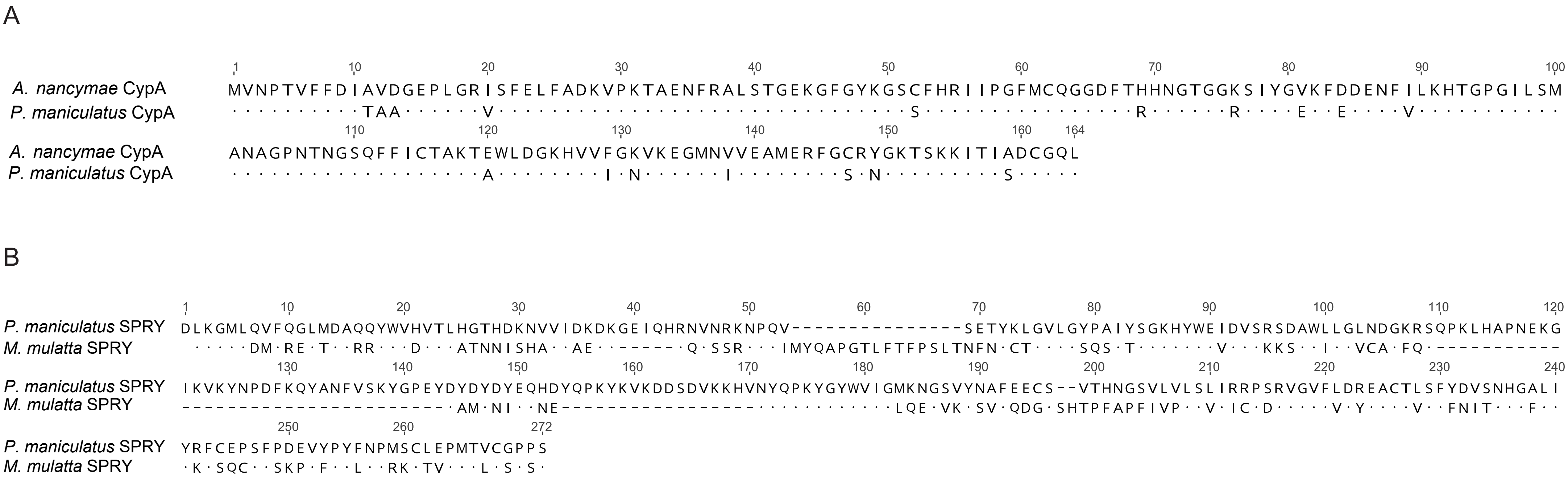

Supplementary Figure S6

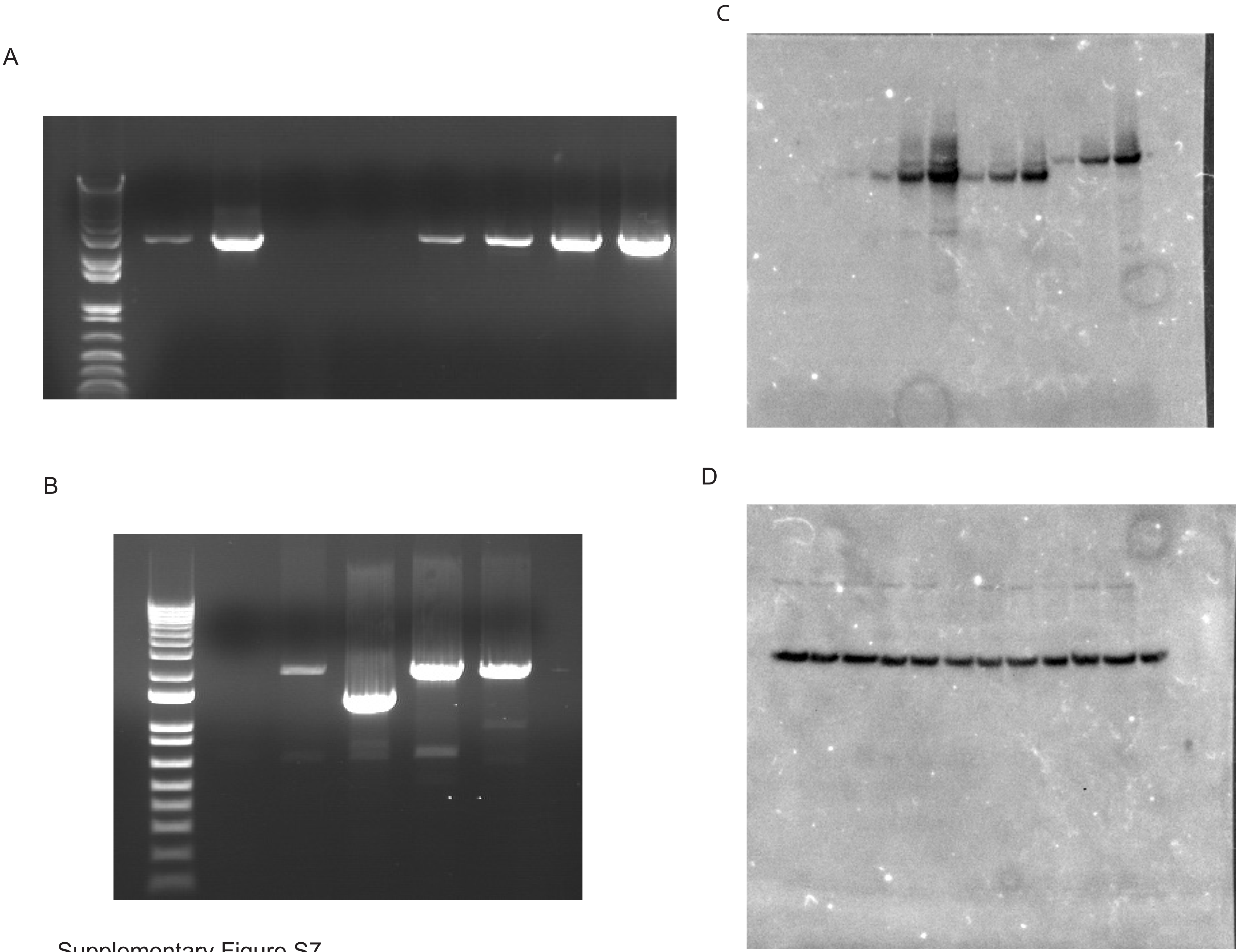

Supplementary Figure S7

Supplementary Data

Supplementary Data S1. Alignment of the partial CDS of *Trim6*, *Trim34* and *Trim5* genes of the indicated rodent species coding for the R-B and CC domains.

Supplementary Data S2. Alignment of the partial CDS of *Trim6*, *Trim34* and *Trim5* genes of the indicated rodent species coding for the SPRY domain.

Supplementary Data S3. Alignment of the partial CDS of a subset of *Trim6*, *Trim34* and *Trim5* genes, as well as the indicated pseudogenes of the select rodent species coding for the R-B and CC domains.

Supplementary Data S4. Alignment of the partial CDS of a subset of *Trim6*, *Trim34* and *Trim5* genes, as well as the indicated pseudogenes of the select rodent species coding for the SPRY domain.

Supplementary Data S1

>Castor_canadensis_Trim6

ATGACTTCAGCCGTCCTGGTAGACATCCGAGATGAAGTAACTTGTCCTATCTGCCTGGAGCTCCTGATGGAACCTC
TGAGTATAGACTGTGGCCACAGCTTCTGCCAGGACTGCATCACAAGGAATAGTGAGGAATCGGTGATCTGCCAAG
A---
AGGGAAGAGCAGCTGTCCTGTGTGTAAGACCAGCTACCAGCCTGGGAACCTCCGGCCTAATCGGCACCTGGCCAA
CATAGTGAGGAGGCTCAGAGAGGTAGTGTTGGGCCCTG---
GGAGGCAGCCAAAGGTAACTCTTTGTGCACTCCATGGAGAGAAACTCCAGCTCTTCTGTAAGGAGGATGGGAGG
CTAATTTGCTGGCTTTGTGAGCGTTCTCAGGAGCACCGTGGTCACCACACATTTCTCATGGAAGAGGTTGCCCAGG
AGTACCAGGAAATGTTCCAGGAGTCTCTGAAGAAACTAAGGAGAGATCAGCAGGAAGCTGAGAGGTTAAAAGCT
GTTATAAGAGAGAAGAGGACATCCTGGAAGAATCAGATGGAGCCTGAGAGACACAGGATCCAGACAGAGTTTAA
TCAGCTGCGAAGAATCCTGGACAGAGAAGAACAGTGGGAACTGAAAAAGCTGGAGGAGGAAGAGAGGAAAGG
GCTGAGTATTATAGAAAAAGCTGAGGGTGAGCTGATCCATCAGAGCCAGTCCCTGACAGAGCTCA---
TCTCAGACCTGGAGCGGCGGTGCCAGGGGTCTACAGTAGATCTGCTGCA-GGATGTGAACAATGTCACG-------
AAAA----------GGAGTGAGTTCTGGACCTTGAGGAAGCCCCAAGCTCTCCCA---
ACCAAGCTGAAAAGTGTATTTCGGGCC

>Castor_canadensis_Trim34

ATGGCTTCAAAAATCCTGATGAACATGTCGGAGGAGGTGACTTGTCCCATCTGCTTAGAGCTTTTGACGGAACCCC
TGAGTCTAGAGTGTGGCCACAGTTTCTGCCAAGCTTGCATCACCGAGAACAATAAGAAGGCAATGATAAGCTCAG
C---
AGAGGAAAGCAGCTGTCCTGTATGTGGTATCAGATACTCACTTGAAAATCTATGGACAAATCAGCATCTGGCTGG
CATAGTGGAGAGACTCAAGGAAGTCAAGTTGAAGCCTG---
ACATTGAGAATCAGAGAGATTTCTGTGTGCGTCATGGAGAGAAACTTCTACTCTTCTGCAAGGAAGATAGGAAGG
TCATTTGCTGGCTTTGTGAGCGTTCTCAGCAGCACCGTGGTCACCATACCTTCATCCTTGAAGAAGCAGTTGAGGA
ATGTCAGGAGAAGCTTCAGGCAGCTCTCAAGAGGCTGAGGAAAGAGCAACAGGAAGCTGAGAAGTTGGAAGCT
GACATCAGAGAACAGAGAATTTCTTGGAAGTATCAGATACAGACTGAAAGGCAAAGGATACAAACAGAATTTAAT
CAGCTTAGAAGCATCCTGGACGGTGAGGAGCAGAGGGAGCTGAAAAGGTTGGAGGAAGAGGAGAAGAAGATAC
TGGATAACTTGGCAGAGGCTGAGGATGAGCTGGCTCAGCAGAACCAGTTGGTGAAAGGGCTCA---
TCTCAGATCTGGAGTGTCGGAGTCAGTGGTCAACAACAGAGCTTCTGGA-GGACAGAAGTGGAATCATG-------
AAGT----------GGTGTGAGTGCTGGACACTGAAGAAGCCAAAAGCTGTTCCC---
AAGAAACTGAAGATTGTGTTCCATGCA

>Cavia_porcellus_Trim5a (LOC100716056), mRNA

ATGGCTTCATCCATCCTGGAGAATATCAAGGAGGAGGTGACCTGCCCCATCTGCCTGAATCTCATGACAGAACCTG
TGAGCACTGACTGTGGCCACACTTTCTGTAAACCATGCATCACTTCCATCTACAAG---TCCATGGAACATGAACA---
AGGAGTGAGCCACTGCCCTGTGTGCCGAGTCCCTTACCAGTTTGAAAACCTGAGGCCCATTCGCCATATGGCCAAC
ATAGTGGAGAGGCTCAGGGAGTTGACACTGAGCCCAA---------------
AGGCAGATCACTGTGACCTCCATGGGGAGAAACTCCTGCTGTTCTGTAAGGAGGACAGAAAGGTCATTTGCTGGC
TTTGTGAGCGTTCTCAGGAACATCATGGTCACAAAGTGGTTCTCATGCAGGAGGCTTCACAGGAGTATCAGGAGA
TGTTCCATGAAGTACTGGAGAAGCTGATGGAGTTGGAGAAAGATTTTGCAAGGTTGAAAGCTCACATTGAAAAAG
AAAGAACTTCCTGGAAGAATCAAATACAGGGGGAGATAGAAAGTGTCCAGACTGCGTTTAAGCAAATGAGAGCC
ACCCTGGACTCTGAGGAGGAGACACGAGTATAAAAACTGAAGGCAGAGGAGGAAGGTATTCTTAATGGCCTGGC
AGACTCTGAGAGGGAGCTGACCCAGCACAATCAGCTACTGAGAGACCTCA---
TCTCAGATGTGCAGCATCGGCTGCAGGGGTCAGCATTGGCGATGCTGCAGGGGTCCGAATGATGTTGTACATCAA

TCAAA----------GGATTAAGTTACTGACTCTGAAGCCACTAAGAACTTTTCCC---
AGGGAGCAGCGGATCATGTTCCGAGCT

>Cavia_porcellus_Trim5b (LOC100715776), mRNA

ATGGAGGAGGCTGTAGAGGAGTATCAGGAGAAGCTCCATCAAGTGTTGGAGAAACTAACTAAGGATGAGAAAG
AGTGTGAGAAGTGGAAAACTCACATCAAACAAGAAAGAACTTCTTGGAA--

---GGATGTAAAGGACTTTGTG-------GAAA----------
GGAATAAGTTATTGACTGTGCAGCAACCAAGAACTTTTCCC---AAGGAGAAAAGGATCGTGTTCAAATCT

>Cavia_porcellus_Trim5c

ATGGCTTTATCCATGCTACACAGTGTCAAGGAGGAGGTGACCTGCCCCATCTGCCTGGAGCTCATGACAGAACCTG
TGAGCACTGACTGTGGCCACACTTTCTGTAAACCATGTATCACTGCAAACTACGAG---TCCAGGGAACATGAACA---
AGGAGTGAGCCACTGCCCTGTGTGCCGAGTCCCTTACCAGTTTGAAAATCTGAGGCCCAGTCGCCAGGTGGCCAA
CATAGTGCAAGGGCTCAGGGAGTTGACACTGAGCCCAA--------------
AGGTAGATCACTGTGACCTCCATGGGGAGAAACTGCTGCTGTTCTGTAAGGAAGATGAGAAGGTCATTTGTTGGC
TGTGTGAGCGTTCTCAGCAGCACCGTGGTCACAACGTGGTTCTCATGCAGGAAGCTGCACAGGAATGTCGGGAGA
AGCTCTATCTAGTGTTGGAGAAACTAACTAAGGATGAGAAAGAGTGTGAGACGTGGAAAGCTCACATCGAACAA
GAAAGAACTTCTTGGAAGCGTCAAATACAGGGGGAGATAGAAAGTGTCCAGACTGCGTTCAAGCAAATGAGAGC
CACGCTGGACTCTGAGGAGGAGATACAACTGCAAAAACTGAAGACAGAGGAGGAAGGTATTCTGAATGGCCTGG
CCGAGTCTGAGAGGGAGCTGACCCAGCAAAGCCAGCTGGTGAGAGAGCTCA---
TCTCAGATGTGCAGCATCGGCTGCAGGGGTCAGTTATGTCCATGCTGCA-GGATATGAAGGACACAGTA-------
GAAA----------GGAGTAGGTTATTGACTGTGAAGCAACCGAGAACTTTTCCC---
AAGGAACAGCGGATCACGTTCCAAGCT

>Cavia_porcellus_Trim5d

ATGGCTTCATCAATCCTGGAGAATGTCAAGGAGGAGGTGACCTGCCCCATCTGCCTGGAGCTCATGACAGAACCT
GTGAGCACTGACTGTGGCCACACTTTCTGTAAACCGTGCATCACTTCAAGTTATGAG---TCCACAGAACGCGAAAA-
--
AGGCGTCAGCCAGTGCCCTGTGTGCCGAGTCCCTTACCAGTTTGAAAACCTGAGGCCCACTCGCCATATGGCCAAC
ATAGTGGAGAGGCTCAGGGAGTTGACACTGAGCCCAA--------------
AGGCAGATCACTGTGCCCTCCATGGGGAGAAACTCCTGCTGTTCTGTAAAGAGGACGGAAAGGTCATTTGCTGGC
TTTGTGAGCGTTCCCAGGAGCACCGTGATCACAATGTTCTTCTCCTGGAGGAGGCTGCACAGGAGTATCGGGAGA
AGCTACACAAAACATTGAAGAAGCTGATGAAAGATAAGAAAGAGTCTGAGAAGTGGAAAGCGCACATCGAAGAA
GAAAGAACTTCCTGGAAGAATGAAATACAGGGGGAGATAGAAAGTGTCCAGGCTGCATTTAAGCAAATGAGAGC
CACCCTGGACTCTGAGGAGGAGATACAACTACAAAAACTGAAGACAGAAGAGGAAGATATTCTGAATGGCCTGG
CAGAGTCTGAAAGGGAGCTGACCCAGCAGAGCCAGCTGGTGAGAGAGCTCA---
TCTCAGATGTGCAGCATCGGCTGCAGGGGTCGGTGATGGCGATGCTGCA-GGATGTGAAGGACACCGTG-------
GAAA----------GGAGTAAGTTATTGACTGTGAAGCAACCAAGAACTTTTCCC---
AAGAAACAAAGGATTTTGTTCCAAGCT

>Cavia_porcellus_Trim6

ATGACTTCAAGAGTTTTGGTGGACATCCGAGAGGAGGTGACCTGTCCCATCTGCCTGGAGCTGCTGACAGAACCA
TTGAGCATAGACTGTGGCCACAGCTTCTGCCAAGCCTGCATCGCAGAGAACACTGAGAAATCAGTGATTGGCCAA
GA---
AGAGGAGAGCAGCTGTCCTGTGTGCCAGACCAGCTACCAACCCCAGCACCTCCGGCCTAATCGGCACTTGGCCAA
CATAGCAGAGCGGCTCCGAGAGGTAGTGTTGGGACCAG---
AAAGACAGACAACGGTCATTCTTTGTGCACAGCATGGAGAGAAACTCCAGCTTTTCTGTAAGGAAGATGGGACGC
TAATTTGCTGGCTTTGTGAGCGTTCTCAGGAGCACCGCGGTCACCACACATTCCTCATGGAGGAGGTAGCCCAGG
AGTACCAGGAAATGTTCCAAGAGTCGCTGAAAAAGTTGAGGAAGGAAGAGGAGGAAGCTGAGAGACTAAAAGC
TGTTATCAGAGAAAAGAGGGCATCCTGGAAGAATCAGATGGAGCCTGAGAGGCACAGGATCCAGAAAGAGTTTA
ATCAACTGCGAAACATCCTAGACAAAGAAGAACAACGGGAACTGAGGAAGCTGGAGGAGGAACAGAGGAAGGG
GTTGAGCATTATCGAGAAAGCTGAGGGTGAGGTGATCCGCCAGAGCCAGTCCCTGAGAGAGCTCA---
TCTCAGATCTGGAGTTCCGATGCCACGGCTCCACAGTGGATCTGCTGCA-GGATGTGAATGATCTCACA-------
CAAA----------GGAGTGAGTTCTGGGCCCTGAGGAAGCCCCAAGTGCTCCCC---
ACCAAGCTGAGAAGTGCCTTTCGAGCC

>Cavia_porcellus_Trim34

ATGGCTTCCAAAACCCTGGCAAACCTAGAGAAGGAGGTGACCTGCCCCATCTGCCTGGAGCTGCTGACAGAACCC
CTGAGTCTAGGCTGTGGCCACAGCTTGTGCCAAGCCTGCGTCACTCTGCACAACGAGAAGACAGG---------------
AAAAGACAGCGGCTGTCCTGTGTGTGGAATCAGGGACCCCCGTGGAAATCTGTGGCCTAATCACCACCTGGCTGA
CATAGTGGAGAAACTCAGGGAGGTGAAGTTGAGCACTG---
GCATTGGAAAGAAGGGAGATTTCTGTGCCCTCCATGGAGAGAAACTTCAACTCTTCTGTAAGGATGATGGAAAAG
TCATTTGCTGGCTCTGCGAGCGTTCTCAGGAGCACCGTGGTCACCATACATTCCTCATGGAGGAGGTAGCCCAGGA
GTGTCAGGAGAAGCTACAGGCAGCTCTCAAGAGGCTGAGGAAAGAGCAGCAGGAAGCTGAGAAATTGAAAGCT
GACATCAGAGAAGACAAACTTTCTTGGAAGTATCAGATGCAGAATGAGAAACAAAGGATACGAGTAGAATTTAAC
TGGTTTAGACACATCCTAGAAAATGAGGAACAGAGGGAGCTGCGAAGATTGGAGAAAGAAGAGAAGGCGAAAC
TGGATATTTTGGCACAAGCTGAGGCTGAGCTGGTACAGCAGAGCCAGGTGGTGACAGAGCTAA---
TCTCAGATCTGGAGCGCCGCAGTCTGTGGTCAGCAGTGGAGCTGCTGCA-GGACATGAGTGGAATTATG-------
AAAT----------GGAGTGAGATCTGGACACTAAGGAAGCCAAAAACTCCTTCC---
AAGAGATTGAAGAGAGGGCTTTGTGCT

>Chinchilla_lanigera_Trim5a

ATGGCTTCGTCAGTCCTGCAAAATGTCAAGGAGGAGGTGACTTGCCCCATCTGCCTGGAGCTCATGACAGAACCT
GTGAGCACTGACTGTGGCCACACTTTCTGCAAACTCTGCATCACTTCCAACTATGAG---TCGACGAAACATGAACA-
--
AGGAGTGGGCAACTGCCCCGTGTGCCGAGTCACTTACCACATTGAAAACCTGAGGCCCAGTCGACATGTGGCCAA
TATAGTGGAGAGGCTCAGGGAGGTGACCTTGACTCCAC---------------
AGGCAGATCAGTGTGACCTCCATGGGGAGAAGCTTGTGCTGTTCTGTTGGCAAGACGGGAAGGTCCTTTGCTGGC
TTTGTGAGCGTTCTCAGGTGCACCGGGGTCACCACACGCTTCTCATGGAGGAGGCTGCGCAGGAGTATCGGAGG
GGTCTTGAGCAAGTGCTGGAGAAGCTGCTGATGGAGGAGAAAGAGTTTGAGAAGTGGAATGCTCACATCGAAGA
AGAAAGAACTTCCTGGAAGAATCAAATACAGGGGGAGAGAGAAAGTGTCCGGGCTGCGTTTCAACAAATGCGAG
CCACCCTGGACTCTGAGGAGAGGACGCACCTGCAGAAGCTGCAGACAGAGGAGCAAGGTGTTCTGAATGGCCTG
GCCGAGTCTGAAAAGGAGTTGGCCCAGCAGGCCTGGGAGGTGAGGGAGCTCA---
TCTCAGATGTGCGGCACCGGCTGCAGGGGTCCACAGTGGCCATGCTGCA-GGATGTGAAGAATACCATG-------

GAAA----------GGTGTCAGTTATTCACTGTGAAGAGACCAAGAACTTTTCCA---
AAGAATCACAGGATCATGTTTCAAGCT

>Chinchilla_lanigera_Trim5b (LOC102008935), transcript variant X2, mRNA

ATG---

CGAGCCACCCTGGACTCTGAGGAGAGGACCCACCTGCAGAAGCTGCAGACAGAGGAGCAAGGTGTTCTGAATGG
CCTGGCCGAGTCTGAAAAGGAGCTGGCCCAGCAGGCCCGGGAGGTGAGGGAGCTCA---
TCTCAGATGTGCGGCACCGGCTGCAGGGGTCCACAGTGGCCATGCTGCA-GGATGTGAAGGACATGCTT-------
AGAA----------GGAGTAAGGTATTCACTGTGAAGAAACCAAGAACTTTTCCC---
AAGAAACAGAGGACCGTGTTCCAAGCT

>Chinchilla_lanigera_Trim5c

ATGGCTTCGTCAGTCCTGGAAGATGTGAAGGAGGAGGTGACCTGCCCCATCTGCCTGGAGCTCATGACAGAACCT
GTGAGCACCGACTGTGGCCACACTTTCTGCAAACTCTGCATCACTTCAAGCTATGTG---TTGACAGAACATGAACA-
--
AGGAGTGAAAAAATGTCCTGTGTGCAGAGACACTTATCAGTTTGAAAACCTGAGGCCCAGTCGACATGTGGCCAA
TATAGTGGAGAGGCTCAGGGACATGACCCTGACCCACA---------------
AGGCAGATCAGTGTGACCTCCATGGGGAGAAACTCCTGCTGTTCTGTATGACAGATGAGAAATTCATTTGCTGGCT
TTGTGAGCATTCTCAACAGCACCGCGGTCACTACACGCTTCTCATGCAGGAGGCTGCACAGGTCTATTGGACGAGA
CTCCAGGAAGTGCTGCAGAAGCTGCTGAAGGATGAGAAAGAGTTTGAGAAGTGGAACGCTCACATCGACGAAGA
AAGGACTTCCTGGAAG---
--GCA-GGA-----
----------------------------GG---

>Chinchilla_lanigera_Trim5d

ATG---

CGAGCCACCCTGGACTCTGAGGAGAGGACGCACCTGCAGAAGCTGCAGACAGAGGAGCAAGGTGTTCTGAATGG
CCTGGCCGAGTCTGAAAAGGAGTTGGCCCAGCAGGCCTGGGAGGTGAGGGAGCTCA---
TCTCAGATGTGCGGCACCGGCTGCAGGGGTCCACAGTGGCCATGCTGCA-GGATTTGAAGGACCCTGCG-------
GAAA----------GGTGTCAGTTATTGGCTGTGAAGAAACCAAGAACTTTTCCC---
AAGAATCAGAGGATCGTGTTTCAAGCT

>Chinchilla_lanigera_Trim6

ATGACTTCAAGAGTTCTAGTGGACATCCGAGAGGAGGTGACCTGCCCCATCTGCCTGGAGCTCCTGACAGAACCTT
TGAGCATAGACTGTGGCCACAGCTTCTGCCAAGCCTGCATCACAGAGAACAGTGAGAAATCAGGGCGCCACCAAG
A---

ACAAGAGAGCAGCTGTCCTGTGTGCCAGACCCGTTACCACCCCCGGAGCCTCCGGCCTAACCGGCACCTGGCCAA
CATAGCAGAGAGGCTGAGAGAGGTTGTGTTGGGACCTG---
GAGAGCAACCAAGGGTCGTTCTTTGTGCATACCATGGAGAGAAACTCCAGCTCTTCTGTAAGGAGGACGGGAAGC
TCATTTGCTGGCTTTGTGAGCGTTCTCAGGAGCACCACGGTCACCACACGTTCCTCATGGCGGAGGTAGCCCCAGA
GTACCAGGAAATGTTCCAGGAGTCCCTGAAGAAGTTGAAGAAAGAAGAGGAGGAAGCTGAGAGACTAAAAGCT
GCTATCAGGGAGAAGAGGGCATCCTGGAAGAATCAGATGGAGCCTGAGAGACACAGGATCCAGAAAGAGTTTAA
TCAGCTGCGAAGCATCCTGGACAAAGAGGAGCAGCGGGAACTGAGGAAGCTGGAGGAGAAAGAGAGGAAGGG
GCTGAGCATTCTAGAGAAAGCTGAGGGTGAAGTGATCCGCCAGAGCCAGTCCCTCAGAGAGCTCA---
TCTCAGACCTGGAGTTCCGGTGCCAGGGGTCCACTGTAGATCTGCTGCA-GGATGTGAATGATCTCACC-------
CAAA----------GGAGTGAGTTCTGGGCCCTGAGGAAGCCGCAAGTGCTCCCC---
ACCAAGCTGAGAAGTGCCTTTCGAGCC

>Chinchilla_lanigera_Trim34

ATGGCTTCCAAAGCCCTGGCAAAGCCAGAGAAGCAGGTGACTTGCCCCATCTGCCTGGAGCAGCTGACAGAACCA
CTGAGTCTAGGCTGCGGCCACAGCTTATGCCAAGCCTGCGTCACCGTGGACAGCGAGGAGGCAGAGACCGGCTC
AGG---
GAAGGAGCGCGGCTGTCCCCTGTGCGGGTGCAGGGACGTCCGCAGGGATCTGGAGGCTGATCGGCCCCAGGCTG
ATGAAGTGGAGAAACTCCGGGAGGTGCAGTTGAGCACAG---
GCACTGGGAAGAAGGCAGATTTCTGTGCACTGCATGGAGAGAAACTCCAACTCTTCTGTCAGGAGGACCAGAAGT
TCCTCTGCTGGCTCTGCGAGCGTTCTCGGGAACACAGGGGCCACCACACGTTCCTCAGGGAGGAGGTAGCCCGGG
AGTGTCAGGAGAGACTCCAGGCAGCTCTCCAGAGGCTGTGGGCAGAGCAGCAGGAAGCTGAGAAGTTGAAAGCT
GCCATCAGGGAAGATAGAGCTTCTTGGAAGGGTGAGGTGCACACCGAGAGACAAAGGATACGAGGAGAATTCTT
TTGGCTTAGAAGCATCCTGAACATTGAGGAGCAGATAGAGCTGCACAGATTGGAGGAGGAAGAAAAAAAGACAC
TGGACAGTTTGGCGCAAGCTGAGGATGAGCTGGCTCAGCAGAGCCAGGTGCTGAAAGAGCTCA---
TCTCAGATCTGGAGCGTCGCAGTCAGTGGTCCACAGTGGAAGTGCTGCA-GGACATGAGTGGCATTATG-------
AAAT----------GGAGTGAGATCTGGACGCTGAGGAAGCCAAAATCTCCTTCT---
ACAAAACTGAAGCGTGTATTTCAGGCC

>Cricetulus_griseus_Trim6

ATGACTTCAGCAGTTCTGGTGGACATCCGAGATGAAGTCACCTGCCCTATCTGCTTGGAGCTACTGACAGAACCCC
TTAGTATAGATTGTGGCCACAGCTTCTGCCAGGACTGCATCACAGGAAGCAGTGACAAGTCAGTGCCCAACCAAG
A---
AGGGAAGAACCGCTGTCCTGTGTGCCGGACAGCCTACCAGCCCGAGAACCTCCGGCCTAATCGACACCTGGCCAT
CATAGTGAAGAGGTTCAGAGGGACCGTGTTGGGCCCAG---
GGAAGCAGAGAGAGGTCATTCTTTGTGGGCTTCATGGAGAGAAACTCCAGCTTTTCTGCAAGGAAGATGGGAAAT
TAATTTGCTGGCTTTGTGAGCGATCTCAAGAACACCATGGTCATCACACATTCCTCATGGAGGAGGTGGCCAAGGA
CTACCAGGAGATGTTCCAGGAGTCTCTGAAGAAGCTGAGGAAGGAGCAGCAGGAAGCCGAGCGGCTAAAAGCTC
TTATCCAAGAGAAGAGGGAGTCCTGGAAGAATCAGGTGGAGCCTGAGAGACACCGGATCCGGACGGAGTTTAAG
CACCTGCGGAGCATCCTGGACCGGGAGGAGCAGAGGGAACTGAGGAAACTGGAAGTAGAAGAGAAGAAGGGTC
TGAGCATCATAGAGAAAGCGGAGGGGGACCTGATCCACCAGAGTCAGTCATTGAAAGATCTCA---
TCTCAGACCTGGAGCACCGGTGCCAGGGGTCCACAGTGGAGCTGCTGCA-GGATGTGAGCGATGTCACA-------
AAAA----------GGAGTGAGTTCTGGACCCTGAGGAAGCCTCAAGCTCTCCCC---
ACCAAGCTGAAAAGTTTGTTTCGAGCC

>Cricetulus_griseus_Trim12

ATGGCTTCAGCATTCATGGCGAATGTAAAGGAGGAAGTGACCTGTCCTATCTGTCTGGACCTGATGGTGGAACCT
GTGAGTACAGATTGTGGTCACAGCTTCTGCCGAGCCTGCATCACACTGAACTACGAG---
TCCATCAAAGGCAAAGA---
GGGAGAGTTCATCTGCCCTGTGTGCCGAGTTACTTACCTGTTTGGGAATCTGAGGCCTAATCGACATGTGGCCAAC
ATAGTGGAGAGACTCAAAGGGTTCAAGTCCAGTCCAG---
AGGAGGAGCAGAAGGTCAGTGTCTGTGCAAAGCATGGAGAGAAACTCCAGCTCTTTTGTGAGAAGGACAAGGTG
GCCATCTGCTGGCTTTGTGAGCGATCTCAGGAACACCGAGGACACCAAACAGTTCTCATCGAAGAGGTGGCCCAT
GAGTACAAGGAGAAGCTCCAGGCAGCTCTGAAGAAGCTGATGGCAGACAAGAAAAAATTAGAGAACTGGAAAG
ATGATCTTCAGAAGGAGAAAGCTTCCTGGGAGAACAAAATTCAGAAAGATGTAGAAAATGTTCAGACAGAGTTTA
GAAAACTGGGAGACATTCTGGACTCTGAGAAGAAGAGTGAGCTGCAGAAACTGATGCAAGAGAAGGAAGGCAT
CATGAACAGCCTGGCAGAGGCTGAAGATAAACATGCCCAGCAGAGCAAGTTACTAGGAGACCTCA---
TCTCAGATGTGGAGCATCAGTTGCAGTGCTCAACCATGGAAATGCTGCA-GGGTGTGGATGGCATCATA-------
TCAT----------GGAGTCATGCCTTTTCACTCAAGAGACCCAAAACTATCCCC---
AAGGAACGAAGAAGAGTGTTCCGAGCC

>Cricetulus_griseus_Trim30a

ATGGCCTCATCAGTTCTGGAGATAATCAAGGAGGAGGTGACCTGTCCTATCTGCCTGGACCTCATGGTGGAACCT
GTGAGTACAGATTGTGGTCACAGCTTTTGCCGAGCCTGCATCACACTGAACTATGAA---
TCCATCAAAGGCAAAGA---
AGAAGAGGGCATCTGCCCTGTGTGTGGAGTTACTTACCTGTTTGGGCATCTGAGACCTAATCGGCATGTGGCCAA
CATAGTGGAGAGTCTACTGGGGTTCAAGTCCAACCCAG---
AGGAGGAGCAGAAGGTCAATGTCTGTGTACAACATGGAGAGAAACTTCAGCTCTTCTGTGAGAAGGACATGGTG
GCCATCTGCTGGCTTTGTGAGAGATCCAAGGATCACCGTGGTCACCAAACAGCTCTCATTGAAGAGGTGGCCCAT
AAGTACAAGGAGAAGCTCCAGGCTGCTATGGAGATGCAAATGGCAAACAAGAAAAAATGTGATGAATGGGAATG
TGACCTGCAAAAGGAGAGAACTTTCTGGGAGACCCAAATACAGAGTGATGTAGAAAATGTTCAGATGGAGTTTAA
AAGATTGCGGGGATTCTTGGACTCCAAGGAGAAGAATGAGGTGCAGAAGCTGATGCAAGAGAAGGAAGACGTT
ATGAACAGCCTGGCAGAGTCTCAAAATGAAGTGATGAAGCAGAGGGAGGCAGTGAGAGACCTCA---
TCTCAGATGTGGAGCATCAGTTAGACTGCTCAACCATGGAAATGCTGCA-GGGTGTGAATTCTGTCCTA-------
ACAA----------GGAGTCAGACCTTAATACTGACACAGCCCAAAATGGTCCCA---
AGAAAACAGAGAAGGAACTTTCAAGCT

>Cricetulus_griseus_Trim30b

ATGGCCTCATCAGTTCTGGAGATAATCAAGGAGGAGGTGACCTGTTCTATCTGCCTGGACCTCATGGTGGAACCTG
TGAGTACAGATTGTGGTCACAGCTTTTGCCGAGCCTGCATCACACTGAACTATGAA---TCCATCAAAGGCAAAGA--
-
AGAGGAGGGCATCTGCCCTGTGTGTGGAGTTACTTACCTGTTTGGGCATCTGAGACCTAATCGGCATGTGGCCAA
CATAGTGGAGAGTCTACTGGGGTTCAAGTCCAACCCAG---
AGGAGGAGCAGAATGTCAATGTCTGTGCACAACATGGAGAGAAACTTCAGCTCTTCTGTGAGAAGGACATGGTG
GCCATCTGCTGGCTTTGTGAGAGATCTCAGGATCACCGTGGTCACCAAACAGCTCTCATTGAAGAGGTGGCCCATG
AGTACAAGGAGAAGCTCCAGGCTGCTCTGGAGATGCAGATGGCAAATGAGAAAATATGTGATGAATGGGAAGAT
GACCTGCAAAAGGAGAGAACTTTCTGGGAGAACCAAATACAGAGTGATGTAGAAAATGTTCAGATGGCATTTAAA
GGACTTCAGGAATTCCTGGACTTCAAGGAGAAGAGTGAGAAGCAGAAGCTGATGCAAGAGAAGGAAGATGTTAT
GAACAGCCTGGCAGAGTCTCAAAATGAGCTGATGAAGCAGAGGGAGGCAGTGAGAGACCTCA---
TCTCAGATGTGGAGCATCAGTTGGACTGCTCAACCATGGAAATGCTGCA-GGGTGTGAATTCTGTCCTA-------

ACAA----------GGAGTCAGACCTTAAGATTAAAACAGCCCAAAATGGTCCTA---
AGAGAAAAGAGAAGGATCTTCCAAGCT

>Cricetulus_griseus_Trim30c

ATGGCCTCATCAGTCCTTGTGAAGATCAAGGAGGAGGTGACCTGTCCTATCTGCCTGGAGCTCCTGAGGGAACCT
GTGAGTACAGATTGTGACCACAGCTTCTGCCGAGCCTGCATCACACTGAACTACCAG---
TCCAGCAAAGGCAAAGA---
AGAGGAGGGCATCTGCCCTGTGTGCCAAGTTACTTACCTGTTTGGGAATCTGAGACCTAATCGACAAGTGGCTAA
CATAGTGGAGAGAATAACAGAGTTCAAGTCCAGCCCAG---
AGGAGGAGCAGAAGGTCAATGTCTGTGCACAACATGGAGAGAAACTCCAGCTCTTCTGTGAGAAGGACATGGTG
ACCATCTGCTGGCTTTGTCAGAGATCTCAGGATCACCGTGGTCACCAAACAGCTCTCATTGAAGAGGTGGCCCATA
AGTACAAGGAGAAGCTGCAAATAGCTCTGCAATTACAGATGGCAAATGAGAAAAGCTGTGATGAACGGAAAAAG
TATCTTCAAAAGGAGAGAAATTACTGGGAGAACCAAATACAGAGTGATGTAGAAAATGTTCAGATGGAGTTTAAA
GGACTTCGGGAATTCTTGGACTCCAAAAAGAAGAGTGAGCTGCAGAAGCTGATGAAAGAGAAGGAAGACGTTAT
GAACAGCCTGGCAGAGTCTCAAAATGAGCTGGAGAAGCAGAGAGAGGCAGTGAGAGACCTCA---
TCTCAGATATGGAGCATCAGTTGGACTGCTCAACCTTGGAAATGCTGCA-GGGTGTGAATTCTGTCCTA-------
ACAA----------GGAGTCAGACCTTAAGAGTGAAACTACCCAAAATAATCCCA---
AGAAAACAGAGAAGCATCTTCCGAGCT

>Cricetulus_griseus_Trim30d

ATGGCCTCATCAGTCCTGATGAAGATCAAGGAGGAGGTGACCTGTCCTATCTGCCTGGAGCTCCTGAGGGAACCT
GTGATTGCAGATTGTGACCACAGCTTCTGCCGAGACTGCATCACACTGAACTATGAG---
TCTAGTAAAGGCAAAGA---
AGAGGAGGGCATCTGCCCTGTGTGCCAAGTTACTTACCTGTTTGGGCATCTGAGACCTAATCAACATGTGGCCAAC
ATAGTGGAGAGAATAACAGAGTTCAAGTCCAGCCCAG---
AGGAGGAACAGAAGGTCAATGTCTGTGCACAACATGGAGAGAAACTCCAGCTCTTCTGTGAGAAGGACATGGTG
GCCATCTGCTGGCTTTGTGAGAGATCCCAGAATCACCGTGGTCACCAAACAGCTCTCATTGAAGAGGTGGCCCATA
AGTACAAGGAGAAGCTACAAACAGCTCTGCACTTGCAGATGGCAAATGAGAAAACATGTGATGAATGGGAAAAG
TATCTTCAAAAGGAGAGAACTTACTGGGAGAACCAAATACACAGTGAGGAAGAAAATGTTCAGATGGCATTTGAA
GGACTGTGGGAATTCCTGGACTCCAAGAAGAAGAGTGAGCTGCAGAAGCTGATGAAGGAGAAGGAAGACGTTAT
GAACAGCCTGGCAGAGTCTCAAAATGAGCTGGAGAAGCAGAGAGAGGCAGTGAGAGACCTCA---
TCTCAGATATGCAGCATCACTTGCACTTCCCAACCATAGAAATGCTGCA-GGGTGTGAATTCTGTCCTA-------ACAA-
---------GGATTCAGACCTTAAGAGTGAAACTACCCAAAATAATCCCA---AGAAAACAGAGAAGCATCTTCCGAGCT

>Cricetulus_griseus_Trim30e

ATGGCCTCATCAGTCCTGATGATGATCAAGGAGGAGGTGACCTGTCCTATCTGCCTGGAGCTCCTGAAGGAACCT
GTGATTGCAGATTGTGACCACAGCTTCTGCCGAGCCTGCATCACACTGAACTATGAG---TCTAGTAAAGTCAATGA-
--
AGGGGAGGGCATCTGCCCTGTGTGCCGTGTTACGTATATGTTTGGGAATCTGAGGCCTAATCGACATGTGGCCAA
CATTGTGGAGAGGCTCAAGGGGTTCAAGTCCAGCCCAG---
AGGAGGAGCAAAAGCTCAATGTCTGTGCACAACATGGAGAGAAACTCCAGCTCTTCTGTGAGAAGGACATGGTG
GCCATCTGCTGGCTTTGTGAGAGATCTCAGGATCACCATGGTCACCAAACAGCTCTCATTGAAGAGGTGGCCCTTA
AGTACAGTGGGAAGCTCCAGGCAGCTCTGCAGACACAGATGGCAAATGAGAAAAGATGTGATGAATGGGAAGAT
TATCTTCAAAAGGAGAGAACTTACTGGGAGAACCAAATACACAGTGAGGAAGAAAATGTTCAGATGGCATTTGAA

GGACTGTGGGAATTCCTGGACTCCAAGAAGAAGAGTGAGCTGCAGAAGCTGATGAAGGAGAAGGAAGACGTTAT
GAACAGCCTGGCAGAGTCTCAAAATGAGCTGGAGAAGCAGAGAGAGGCAGTGAGAGACCTCA---
TCTCAGATATGCAGCATCACTTGCACTTCCCAACCATAGAAATGCTGCA-GGGTGTGAAATCTGTCCTA-------
ACAA----------GGAGTCAGACCTTAAGAGTGACACTACCCACAATGATCCCA---
AGAAAACAGAGAAGCATCTTCCGAGCT

>Cricetulus_griseus_Trim34

ATGGCTTCAGCAGTTCCGATGAATGTAAAAGAGGAGACCATTTGCCCTATCTGCCAAGAGCTTTTGAAAGAACCCC
TGAGTTTAGGCTGTGGCCACAATGTATGCCAAGCCTGCATCACCATGAACAAGAAGAATGCAGTGGTCAACAGCA
A---
AGGGAAAAGCAGCTGTCCTGTGTGTGGTACTAGATTCTTATTTAAAAATCTACAGGTTAATCGGCATCTGGCAGAT
ATAGTAGCAAGACTTAGGGAAGTCAAGTTGAACCCTG---
ACACTGGGACAAAAAGAGATCTCTGTATACATCACGGAGAGAAACTCCTTCTCTTCTGTAAGGAAGATAGGAAGG
TCATTTGCCGACTTTGTATGCATTCTCGGGAGCATCTTGATCACAACACCTTCCTCCGGGAGGAAGCAGTCAAGGA
ATATCAGGAGACTCTCCAGAAAGCTCTCAAGAGGCTGAGGGAGGAGCAGGAGAAGGCAGAGAAATTGGAAGCT
GACATCAAAGAAGACAGAATCTCCTGGAAGTGCCAGATCCAGAATGAGAGACAAAGGATACAAACTGGGTTTAA
TGAGCTTAGGAGAACCCTGAACGAGGAGGAACAGAGAGAGCTGAAAAGACTGGGGGAGGAGGAGCAGCTGAT
AGAGGACAGCCTGGCAGAGGCGGAGGCTGAGCTGGCTCAACAGAGCCAGTTGGTGGAAGAACTTA---
TCTCAGATCTGGAGCGTCGGTGCCAGTGGTCAGCCATAGAGCTGCTGCA-GGATATGAGCGGTATCTTG-------
AAAT----------GGAGTCAGATCTGGACATTGAAGAAGCCAAAAGTGGTTTCT---
AAGAAACTGAAGGTGGTATTCCAAGCT

>Dipodomy_ordii_Trim5b

ATGGCTTTAGCAGTCTTGACCAACATAAAGGAAGAGGTGACCTGCCCCATCTGTCTGGAGCTCATGATAGAACCCA
TGAGCATAGACTGTGGACACAGCTTCTGCCAAGCCTGTATCACATCAAATGATGAT---TCTACCATAGGCCCAAG---
AGGGGAGACCAACTGTCCTGTGTGTCGAATCCCTTACACCGTTGAGAACCTCCGGCCTAATAGACATGTGGCCAAC
ATAGTGCACAGTCTCAAGGAGGCTAGGTTGATCCTAG---AGGAAG---
AGAATGTGCATCACTGCGCAGAGCATGGTGTGAAACTCCAGCTTTTCTGTAAGGATGATGGGAAGGTGATTTGCT
GGCTTTGTGAGAGATCTTTACAGCACCGTGGTCACCACACATACCTCTTAGAGGAGGTTGCCCAGGAGTACCAGA
ACAAGCTCCTGGTAGCTGTGAAGAAGATGAAGAAGAAGAAGGAAGAATCTGAGAAGTGGAAAGCTGAACTCCAA
GAAGAGAAAATTTTATGGAAGAAGCACATACAGGTCAAGAGAGAAAACGTTTGGGCAAAGTTTGAAAAACTGAG
AGGCATCTTGGCTTCGGAGGAGGCAAAAGAGCTGCAAAGGCTGAAAGAAGATGATGAAAATGTTTTGAATAGTC
TGGAAAAGTCTGAAAATGAACTGGCCCAGAGAAATGAGTTGGTAACAGAGCTCA---
TCTCAGAGCTAGAGCATCGGTTACATGGATCAAGGATGGAGATGCTGCA-GGATGTTAATGAAATCACA-------
AAAA----------GATGTGATATCTTGACAATGGAGAAGCCAATAACTTTCCCC---
AAGGAAGAACGGAGATTGTACCAAGCT

>Dipodomys_ordii_Trim5a

ATGGCTTCATCAATCTTGGCCAATGTAAAGGAGGAGGTGACCTGCCCCATCTGTCTGGAGCTCATGATTGAACCCA
TGAGCATAGACTGTGGACACAGCTTCTGCCAAGCCTGTATCACATCAAGCTATAAA---TCCACTATAGGCCC------
AAGAGGACCTGTGTGCCCCATATGTCGATTCCCTTATGCCTTTGAGAGCCTCCGGCATAATCGACATGTGGCCAAC
ATAGTGCAGAATCTCAGGGAGGTCAAGTTGAACTCAC------
AGGAGCAGACTGTGCATCACTGCACACAGCATGGTGAGAAACTCCAGCTCTTCTGTAAGGACGATAGGAAGGTGA
TTTGCTGGCTTTGTGAGCGATCTCAGCAGCATCATGGACACCGTACATGCCTCTTGGAGGAGGTTGCCCAGGAGTA

CCAGGGCAAGTTCTGGGCAGCTCTGAACAAGCTAAGGAAGAACAAGGAAGAATCTGAGAAGTGGAAAGCTGAAC
TCCAACAAGAGGAAATGTCTTGGAAGACTCAAATACAGGGCAAGATACAAAATGTTCAGGCAGATTTTATACAAC
TGAGAGGCATCCTGGACTCTGAGGAGGAGAAAGTACTGCAAAAGCTGAAAAAGGAAGAGGAAGATGGTTTGAA
AAGGTTTGCAGTGTCAGAAAACAAGCTGACCCGGCAGAGCAATTGGGTGACCAAGCTCA---
TCTCAGATGTGGAGCATCTCTTAAAAGGGTCCACAATGGAGATGCTGCA-GAATGAGAATACCATCATG-------
AGAA----------GTTGTGAGCTATTGACAATGAAGAAACCAGTAACGTTCCCA---
AAGAAAAAACAGACTGTGTTCCCAGTA

>Dipodomys_ordii_Trim5c

ATGGCTTCAGCAATCTTGGCCAACATAAAGGAAGAGGTGACCTGCCCCATCTGCCTGGAGTTCATGATAGAACCTA
TGAGCATAGACTGTGGACATATCTTCTGTCAAGCCTGTATCACCTCAAGCTATAAA---TTCAACATGGGCACAGG---
ATCGGAGAGCAATTGTCCTGTGTGTCAAAGCCCTTACAAATTTGAGAACTTACGACTTCATCGACACATGGTCAAC
ATAGTGCAGAGTCTCAAGAAAGCCACGTTGAGCTCAA------
AGGAGCAGAATGTGCATCATTGTGCACGGCATGGTGAGAAACTCCAGCTCTTCTGTAAGGATGATGAGGAGGTG
ATTTGCTGGCTTTGTGAGCGATCTCAGCAGCACTGTGGTCACAACACATGCCTCTTGGAGGAGGTTGCCCAGGAGT
ACCAGAACAAGCTCCAGGCAGCTCTGGACAAGCTGAGGAAGAACAAGGAAGAATCTGAGAAGTGGAAAGCTGA
ACTCCAACAAGACAAAACTTCTTGGCAGACTCAAATACAGGGCAAGATACAAAATGTCCAGGCAGATTTTACACAA
TGGAAAGGTATCCTGGACTCTGAGGAGGAGAAAGTACTGCAAAAGCTGAAAAGGGAGGAGGAAGATGGTTTAC
AAGCCCTGGCAGTGTCAGAAAATGAGCTGACCCAGCAGAGTAAGCTTGTGACTGAGCTCA---
TCTCAGATGTGGAGCATCAGTTAGAAGGGTCCACAATGGAGATGCTGCA-GGATGTGAATACCATCATG-------
AGAA----------GATGTGAACTCTTGACAATGAAGAAGCCAATACCTTTCCCC---
AAGAAAGAACAGACAGTGTTCCCAATA

>Dipodomys_ordii_Trim6

ATGAGTTCAGCAGTTCTGGTGGACATCCAAGAGGAGGTGACTTGCCCCATCTGCCTGGAACTACTCACAGAACCCC
TGAGCATCGACTGTGGCCACAGCTTCTGCAAAGCCTGCATCACAAGGAACAGTGAAGAATCAGTGCGCCACCGAG
A---
AGAGAAGAGCAGCTGTCCTGTGTGCAAGGCCAGCTACCAGCCTGGGAGCCTCCGGCCTAATCGGCATCTGGCCAA
CATAGTAAAGAGGCTCAGGGAGGTAGTGTTGGGCCCAG---
GGAAGCAGGCAAAGGTCATTCTTTGTGCACAGCATGGTGAGAAACTCCAGCTCTTCTGTAAGGAGGACGGGGAG
TTGATTTGCTGGCTTTGTGAGCGATCTCAGGAGCACCGTGGTCACCACACATTCCTCATGGAGGAGGTTGCCCAGG
AGTACCAGGAAATGTTCCAGGAGTCTCTGAAGAAGCTGAGAAAAGAGCAGCAAGAAGCTGAGAGGCTAATAGCT
GTGATAAGAGAGAATAGAACATCTTGGAAGAGTCAGATGGAGCCTGAGAGACATAGAATCCAGAAAGAATTTAA
TAAGCTGCGGAACATTCTGGACAGAGAGGAGCAGCAGGAACTGAAGAAGCTGGAGGAGGAAGAGAGGAAAGG
GTTGGGCATTATGGAGAAAGCAGAGGGTGAGCTGATCCACCAGAACCAGTCCCTGAGGGAGCTCA---
TCTCAGACCTGGAGCGGCGATGCCAGGGATCCACAGTAGATCTGCTACA-GGACGTGAGTGATGTCACG-------
AAAA----------GGAATGAGTTCTGGACCTTGAGGGAGCCTCTGGCTCTCCCC---
ACCAAGCTGAGAAGTAAGTTCCGAGCC

>Heterocephalus_glaber_Trim5

ATGGCCTCCTCAGTTCTGGTGGAGGTGAAGGAGGAGGTGACCTGCCCCATCTGCCTGGAGCTCCTGAAAGAACCT
ATGAGCACTGACTGTGGCCACAACTTCTGCAAACTGTGCATCACTGCAAACTGTGAG---TCCTCAGTACATGAGCA-
--
AGGAGTGAGCAGCTGCCCTGTGTGCTGAGTCACTTTCCACTTTGCAACCCTGAAGCCCAATTGACACATGGCCAAC

ATAGTGAAGAGGCTCAGGGGGTTGACCCTGATCCCGA---------------
GGGCAGATCACTGTGAACACCATGGTGAGAAACTCCTGCCCTTCTGTAAGGACGATGGGAAGGTCATTTGCTACC
TTTGTGAACATTCTCTGGAACACCAAGGTCACCACATGATGCTCATGAAGCAGGTTGCATGAGAGTACCAGGAGC
AGCTCCATGAAGTGCTGCAGAAGCTGATGAAGGATGAGAAAGAGTTTGAGAAGTTGAAAGCTGACATCAAAGAA
GAAAGAACTTCCTGGAAGAATCAGATACAGTGGGAGATAGAAAATGTCCAGAATGTGTTTAAACAATTGAGAGTC
ACCCTGGACTTTGAGGAGAAGAGGCTCCTGAGAAAACTGAAGACAGAGGAGGAACAGATTGTGAATAGCCTGGC
TGTTTCTGAAAATGAGCTGACCCAGCAAACCTAGCTGGCAAGAGAGCTCA---
TCTCAGATGTGGAGCATCGGTTGCAGGGGTCAGCAATGGAGATGTTGCA-GGT----------------------AAGA----------
----

>Heterocephalus_glaber_Trim6

ATGACTTCAAGAGTTTTGGTGGACATCCGAGAGGAGGTGACCTGTCCCATCTGCCTGGAGCTCCTGACAGAACCTT
TGAGCATAGATTGTGGCCACAGCTTCTGCCAAGCCTGCATCATAGAGAACAGTAAGAAATCAGCGACCAGCCAAG
A---A---
GAGAGCAGCTGTCCTGTGTGCCAGACCAGCTACCACCCCCGGAACCTCCGGCCTAATCGGCACCTGGCCAACATA
GCAGAGAGGCTCAGAGAGGTAGTGCTAGGACCAA---
GAAACCAGCCACAGGTCATTCTTTGTGTGCGCCACGGAGAGAAACTCCAGCTCTTCTGTAAGGAGCATGGGACGC
TAATTTGCTGGCTCTGCGAGCGTTCCCAGGAGCACCGTGGTCACCACACGCTCCTCATGGAGGAGGTAGCCCAGG
AGTACCAGGAAATGTTCCAAGAGTCCCTGAAGAAGTTGAGGAAAGACGAAGAGGAAGCTGAGAAGCTAAAAGCT
GTTATCAGAGAGAAGAGGGCATCCTGGAAGAATCAGATGGAGCCTGAGAGACACAGGATCCAGAAAGAGTTTAA
TCGGCTGCGAAGCATCCTGGACAAAGAGGAGCAGCGGGAACTGAGGAAGCTCGAGGAGGAAGCGAGGAAGGG
GCTGAGTATTATAGAGAAAGCTGAGGGTGAGGTGACCCACCAAAGCCAGTCCCTGAGAGAGCTCA---
TCTCAGACCTGGAGTACCGGTGCCAGGGCTCCACAGTGCATCTGCTGCA-GGATGTGAATGATCTCACA-------
CAAA----------GGAGTGAGTTCTGGACCCTGAGGAAGCCCCAAGAGCTCCCC---
ACCAAGCTGAGAAGTGCATTTCGAGCC

>Heterocephalus_glaber_Trim34

ATGGCTTTCCAAATCCTGGAGAACCTAGAGATGGAGATGACCTGTCCCATCTGCCTAAAGCTGCTGACAGAACCCT
TGAGTCTAGGCTGTGGTCACAGGCTTTGCAAAGCCTGCATCACTGTGGACAACCAGGAGGCAGCAGTTGGCTCAG
G---
AAAGGAGAGCAGCTGTCCTGTGTGTGGTGTCAGGTACCCACTCGGCAACCTGTGGGCTAATCGCCACCTGGCTGA
CATAGTGGAGAAATTCAGGGATGCAAAGCTGACCACAG---
GCATTGGGAAGAAGAGAGATTTCTGTGCACTCCATGGAGAGAAACTCCAACTCTTCTGTGAGGAGGATCGAAAG
GTCATTTGCTGGCTTTGCGAGTGTTCTGGGGAACACCCTGGTCACCACACGCTCCTCATGGAGAAGGTAGCCAAA
GAGTGTCAGGAGAAACTCCAGGCTGCTCTCAGGAGGCTGAGGAAAGAGCAACAAGAAGCTGAGAAGTTGCAAG
CTGACATCAGAGAAGACAGAATTTCTTGGAAGTATCAGATACAGACTGAGAGACAAAGAATACAGACAGAATTTG
ATCAGCTTAGAAGAATCCTGGACAATGAGGAACAGAGAGAACTGCAAAGATTGGAGGAGGAAGAAAAGAAGAC
ACTGGATCATTTGGCACAGGCTGAGGATGAGCTAGTTCAGCAGAGCCAGGTGGTAAAAGAGCTCA---
TCTCAGATCTGGAGCTTCGGAGTCAGTGGTCAGCAGTGGAACTGCTGCA-GGACATGAGTGGAATTATG-------
AAAT----------GGAGTGAGATCTGGACACTGAAGAAGCCAAAAACTCCTTCC---
AAGAAGCTGAAGAGTGTATTTCACGCC

>Homo_sapiens_Trim22

ATGGATTTCTCAGTAAAGGTAGACATAGAGAAGGAGGTGACCTGCCCCATCTGCCTGGAGCTCCTGACAGAACCT
CTGAGCCTAGATTGTGGCCACAGCTTCTGCCAAGCCTGCATCACTGCAAAGATCAAGGAGTCAGTGATCATCTCAA
G---
AGGGGAAAGCAGCTGTCCTGTGTGTCAGACCAGATTCCAGCCTGGGAACCTCCGACCTAATCGGCATCTGGCCAA
CATAGTTGAGAGAGTCAAAGAGGTCAAGATGAGCCCAC---
AGGAGGGGCAGAAGAGAGATGTCTGTGAGCACCATGGAAAAAAACTCCAGATCTTCTGTAAGGAGGATGGAAAA
GTCATTTGCTGGGTTTGTGAACTGTCTCAGGAACACCAAGGTCACCAAACATTCCGCATAAACGAGGTGGTCAAG
GAATGTCAGGAAAAGCTGCAGGTAGCCCTGCAGAGGCTGATAAAGGAGGATCAAGAGGCTGAGAAGCTGGAAG
ATGACATCAGACAAGAGAGAACCGCCTGGAAGAATTATATCCAGATCGAGAGACAGAAGATTCTGAAAGGGTTC
AATGAAATGAGAGTCATCTTGGACAATGAGGAGCAGAGAGAGCTGCAAAAGCTGGAGGAAGGTGAGGTGAATG
TGCTGGATAACCTGGCAGCAGCTACAGACCAGCTGGTCCAGCAGAGGCAGGATGCCAGCACGCTCA---
TCTCAGATCTCCAGCGGAGGTTGAGGGGATCGTCAGTAGAGATGCTGCA-GGATGTGATTGACGTCATG-------
AAAA----------GGAGTGAAAGCTGGACATTGAAGAAGCCAAAATCTGTTTCC---
AAGAAACTAAAGAGTGTATTCCGAGTA

>Jaculus_jaculus_Trim5

ATGACTTCAGAAATCCTGGCCTGTGTAAAAGAGGAGGTGACCTGTCCCATTTGCCTGGATCTCTTCATAGAACCCC
TGAGCATAGGCTGTGGTCACAGCTTCTGCAAAGCCTGCATCACTTTGATGTATGAG---TCCAAGAAAAACAAAGC--
-
TGGTCAGGGCACGTGCCCCGTGTGTCAGGCCAGTTACCAGTTAGAGAATCTGCAGCCTAATCGGCACCTAACCAA
CATAGTGGAGAGGCTCAAGGGGGTTCAGCTGAACCCAAAGCAGGAGAAGCAGAAGGAGTATCGCTGCGCGCAC
CATGGAGAGAAGCTGCAGCTCTTCTGTACGACAGACAGGCTGGTCATCTGCTGGCTTTGCGAGCGCTCTCAGGAA
CACCGTGGTCACAAGACATTGCTCCTGGAGGAAGTGGCCCAGGAGTACAAGGAGAAGCTCCAGGAAATTCTGCA
GAAACTGAGGGCAGAGGAGAAAGAATTTGAGAACCGGAAAGATGTCATTCAAAAAGAGAGAACCTACTGGAAG
AATCAAATAAAGGGAGAACAACAAAATGTTAAGGCGGTATTTACACAACTGGCACAAGGCTTGAACTCTGAGGAG
AAGAATGAGCTACAGAAGTTGCAGCAAGAGGAAAAGTATATTTTGGATAGTCTGCAAGAGTCTGAAAATGAACTG
GCCAAGCAGAAGCAGTTGGTGAGAGACCTCG---
TCTCAGAGCTGGAGAAGCGGTTGCAGGGGTCCTCCATGGACATGCTACA-GGATGTGAATGACATCATA-------
GAAA----------GGAGTGAGACCTTGACTCCGACGACACCAAAAACTTTCCCG---
AAAGGACAAAGACGAGAATTCCAAGGT

>Jaculus_jaculus_Trim6

ATGACGTCGGCACTCCTGGTGGACATCCGAGATGAAGTGACATGTCCCATCTGCCTGGAGCTCCTGACAGAACCC
CTGAGCATAGACTGTGGCCACAGCTTCTGCCAGGCCTGCATCATGGCCAATGATGACAATTCACTGGTCAGCCAAG
A---
AGGGAAGAGCAGCTGTCCTGTGTGCCACGCCAGCTACCAGACTGTGAACCTCCGGCCGAATCGACACCTCGCCAG
CATCGTGAAGAGGCTCAGAGATGTGGCCCTGGGCCCGG---
GGCAGCGGCTCGAGGCCATGCTTTGTGCGCTTCATGGGGAGAAGCTCCAGCTCTTCTGCAAGGAGGACGGGAAG
CTCATTTGCTGGCTTTGTGAGCGTGCTCAGGAGCACCACGGTCACCGAACATTCCTCATGGAAGAGGTGGCCCAG
GAGTACCAGGAGATGTTCCAGGAGTCTCTGAAGAAGCTAAAGAAAGAGCAGCAGGAAGCTGAGCGGCTAAAAAC
TGTCATCAGAGAGAAGAGGTCGTCCTGGAAGAATCAGCTGGAGCCCGAGAGACGCAGGATCCAGACAGAGTTCA
GTCGATTGAGAAGCATCCTGGACAGAGAGGAGCAGCGGGCACTGAAGAAGCTGGAAGAAGAACAGAGGAAGG
GGCTGAGCATCATAGAGAAGGCCGAGGGAGACCTGATCCACCAGAGCCAGTCCCTGACAGAGCTCA---
TCTCTGACCTGGAGCACCGCTGCCAGGGGTCCACGGTGGATCTGCTGCA-GGATGTGAGTGATGTCACA-------

AAAA----------GGAGTGAGTTCTGGACCCTGAGGAAACCCCAACCTCTCCCC---
ACCAAGCTGAGAAGCATGTTTCGAGCT

>Jaculus_jaculus_Trim34

ATGGCTGCAGCAGTCCTCAT---------
GGAGGAGGCCACATGTCCTATCTGCCTGGAGCTTTTGACAGAGCCCGTGAGTCTGGACTGTGGCCACAGCTCGTG
TCGAGCCTGCCTGTCCACGCA------------GGGCATCGGCCCCGC---
CAGGAAAACCAGCTGCGCTGTGTGTGGTACCCGGTGCTCAGCTGAGAACCTGGGGGTGAATCGGCCCCTGGCAG
GCATAGTAGAGAGACTCAGGGAGGTCAGGATGAGGACTG---
ACACTGAAAAGAAGGAAGACTTGTGTGTACGCCATGGGGAGAAGCTGCTTCTCTTCTGTGAGGAAGACAGGAAG
GTCATCTGCTGGCTCTGTGAGCGTTCTCGGGAGCATCATGGCCACCATACCTACCTCCTGGAGGAGGTAGCCACAG
AGTGTCAGGAGAAGCTTCGGAGTGCTCTCGAGAGACTGAGGAGGCAGCAGCGGGAAGCTGAGCAACTGGGAGC
TGACATCAGAGAAGAGAGGATTTCTTGGCAGGTTCAAATACAGACTGAGAGACAAAGGATACAAACAGGATTTA
ACCGGCTTCGAAGCATCCTAGACAGCGAGGAGCAGAGAGAGCTGCGGAGACTGGAGGCGGAGGAGCGGAAGAT
CCTGCACAGCCTGGCAGAGGCCGAGGCCGAGCTGGCTCAGCAGAGCCGGGTGGTGACCGAGCTCA---
TTGAGGATCTGGAGCGCAGAGGCCGGTGGTCGCCGAGGGAGCTGCTCCA-GGATATGAGTGGGATCATG-------
AAAT----------GGAGTGAGGTCTGGACACTGAAGAAACCAAAGGCTCTTCCC---
AAGAAACTGAAGACTGTATTCTGTACC

>Marmota_flaviventris_Trim5a

ATGGCTTCAGCAATCCTGGGGAATGTAAAGGAGGAGGTGACCTGCCCCATCTGCCTGGATCTCCTGACAAAACCT
CTGAGCATCGACTGTGGCCACAGCTTCTGCCAGGCCTGCATCACATCAAACTATGAG---TCCATGATGAGTCAAAA-
--
AGGGGAGAGCAGCTGCCCTGTGTGCAGAATCAGTTACGAGTTTGAGAACCTGCGGCCTAATCGGCATGTGGCCA
ACATAGTGGAGAGGCTCAGGGATGTCAAGCTGAACCCAG---
AGGAGGAGCAGAAGGTGTATCACTGTGCACGCCATGGAGAGAAACTCCTACTCTTCTGTAAGGAGGACAAGCAG
GTCATTTGCTGGCTTTGTGAGCGTTCTCAGGAGCACCGTGGACACTCCACATTCCTCTTGGAGGAGGTTGTCCGGG
AGTACCAGGAGAAGTTTGAGGAAATGTTGCAAAACCTGAAGAGATCCAGGGAAGAAGCTGAGGAGTGGAAAGT
TGACATCCAAACTGAGAGAACTTTCTGGAAGAACAAAATACAGAGTGAGGTGGAAAATGTCCAGATAGAGTTTGG
AAAACTGAGAGACATCCTCCAATCTGAGGAGAAGCAGGAGATTCAAAAGCTGAAGAAGGAGGAGGAAGTTATTA
TGAACAGCCTGACAGAGTCTGAAAGTGAGCTGATCCAGCAGAGCCAGGTGGTGAAAGATTTCA---
TCTCAGATCTGGAACATCGGTTGGAAGGGTCAACAGTTGAGATGCTGCA-GGATACGAATGATGTCATG-------
AGAA----------GGTGTAAGGACTTAATGCTGAGGAAGCCAAAAACTTTCCCC---
AAGGAACAAAGAAGAGTATTCCGAGCT

>Marmota_flaviventris_Trim5b

ATGGCTTCAGCAATCCTAGGGAATGTAAAGGAGGAGGTGACCTGCCCCATCTGCCTGGATCTCCTGACACAGCCT
CTGAGCCTTAACTGTGGCCACAGCTTCTGCCAGGCCTGCATCACATCAAACTATGAG---TCCATGATGAGTCAAAA-
--
AGGGGAGAGCAGCTGCCCTGTGTGCAGAATCAGTTACGAGTTTGAGAACCTGCAGCCTAATCGGCATGTGGCCAA
CATAGTGGAGAGGCTCAGGGGCGTCAAGCTGAACCCAG---
AGGAGGAGCAGAAGGTGTATCACTGTGCACGCCATGGAGAGAAACTCCTACTCTTCTGTAAGGAGGACAAGACG
GTCATTTGCTGGCTTTGTGAGCGTTCTCAGGAGCACCGTGGACACTCCACATTCCTCTTGGAGGAGGTTGTCCAGG
AGTACCAGGAGAAGTTTCAGGAAATGTTGCAAAACCTGGTGATAGCCAAGGAAGAAGCTGAGAAGTGGAAAGTT

GACATCCAAACTGAGAGAACTTTCTGGAAGAGCAAAATACAGAGTGAGGTAGAAAATGTCCAGGTAGAGTTTGG
AAAACTGAGAGACATCCTGAACTCTGAGGAGGGGCAGGAGATTCAAAAACTGAAGAATGAGGAGGAAGTTATTA
TGAACAGCCTGACAGAGTCTGAAAGTGAGCTGATCCCGCAGAGCCAGTTGGTGAAAGATGTCA---
TCTCAGATCTGGAACATCGGTTGGGAGGGTCAACAGTTGAGATGCTGCA-GGATATGGATGATGTCATG-------
AAAA----------GGTGTAAGGACTTAATGCTGAGGAAGCCAAAAACTTTCCCC---
AAGGAACAAAGAAGAGTATTCCGAGCT

>Marmota_flaviventris_Trim6

ATGACTTCAACAGTTCTGGGGGACATCCGCGATGAGGTGACCTGCCCCCTTTGCCTGGAGCTCCTGACCGAACCCT
TGAGCACAGACTGTGGCCACAGCTTCTGCCAGGCCTGCATCACAGAGAATAGAGAGGAATCAGAGATGAGCCAG
GA---
CAAGGAGCGCAATTGTCCTGTGTGCCAGACCAGCTACCAGCCTAGGAACCTCCGGCCTAATCGGCACCTGGCCAA
CATAGCGCAGCGGCTCAGAGAGGTAGTGTTGTGCCCAG---
GGAAGCAGCTGCAGGTAATTCTTTGTGCACACCATGGAGAAAAACTCCAGCTCTTCTGTAAAGAGGATGGGAAGC
TAATTTGCTGGCTTTGCGAGCGTTCTCAGGAACACCGTGGCCACCATACATTTCTCATGGAGGAGGTCGCCCAGGA
GTACCAGGAGATGTTCCAGGAGTCTCTGAAGAAGTTGAGGAAAGAACAGCAGGAAGCTGAGAAGCTAAGAGCTT
TTATCAAAGAGAAGAGGGCATCCTGGAAGACTCAGATGGAGCCCGAGAGACAGAGGATCCAGACAGAGTTTAGT
CAGCTGCGAAGCATCCTGGACAAAGAGGAACAGCGGGAACTGAAGAAGCTGGAGGAGGAAGAAAGGAAGGGG
CTGAGCATTATAGAGAAAGCCGAGGAGGAGGTGATCTACCAGAGCCAGTCACTGGAAGAGCTTA---
TCTCAAATCTGGAGCATCGATGCCAGGGCTCAGTAATGGATCTGCTGCA-GGATGTGAGTGATGTCATG-------
CAGA----------GGAGTGAATTCTGGACCCTGAGGGACCCCCCAGCTCTCCCT---
ACCAAGCTGAGAAGTATGTTCCGAGCC

>Marmota_flaviventris_Trim34

ATGGCTTCAAAAACCCTGGTGAACACACAAGAAAAGGCAAAATGTCCCATCTGCCTAAAGCATTTAACAGAGCTAC
TGACTCTAGGCTGTGGTCACAGCTATTGCCAAACATGCATCTCTAGGAACAAGAAA---TCTGTGACCAGCCCAGG--
-
AGGGGAAAACAGGTGTTTCATATGTAGTACCAGGAACTTATTTGGAAATACCAAAGCTAATCAGCCTCCAGCTGAC
GTAGCAGAAGGACTCAGGGAAGTCAAGTTGAGCCCTA---
GCACTGTGCAAAATGCAGACTTATGTGCATGCCATGGAGAGAAACTCCTACTCTTCTGTGTGGAGGATAGGAAGG
TAATTTGCTGGCTTTGTGAGCGTTCTCAAGAGCACCGTGGGCACCGAACATTACTCATGGATGAAGTGACCAAGG
AATGTCAGGACAAGCTCCAAGCAGTTCTCAAGAGGCTGAGGAAGGAACAAGAAGAAGCTGAGAAGTTGGAAGCT
GATATCCAAGAAGAGATCACTTCTTGGAAGTGTCAGGTAGAGACTGAGAGAAAAAGGATACAAATGGAATTTAAT
CATCTTAGAAGAATCCTGGACAGCGAGGAAGAGCGAGAGCTACAAAGATTGGATCGGGAGGAGAAGACGACAC
TGGATAGCTTGGCAGAAGCAGAGGATGAGCTGGTCTATCAGAAGCAGTTGGTGAAAGAGCTCC---
TCTCAGATCTAGAGCGTCGGAGTCAATGGTCAACACTGGAGCTACTGCA-GGACACAAGTGGAATCATG-------
AAAT----------GGAGTGAGATCTGGACTCTGAAGAAGCCAAAAACTGTCTCC---
AAGAAGCTGAAGACTGTATTCCGTGCT

>Marmota_marmota_Trim5a

ATGGCTTCAGCAATCCTGGGGAATGTAAAGGAGGAGGTGACCTGCCCCATCTGCCTGGATCTCCTGACAAAACCT
CTGAGCATCGACTGTGGCCACAGCTTCTGCCAGGCCTGCATCACATCAAACTATGAG---TCCATGATGAGTCAAAA-
--
AGGGGAGAGCAGCTGCCCTGTGTGCAGAATCAGTTACGAGTTTGAGAACCTGCGGCCTAATCGGCATGTGGCCA

ACATAGTGGAGAGGCTCAGGGGTGTCAAGCTGAACCCAG---
AGGAGGAGCAGAAGATGTATCACTGTGCACGCCATGGAGAGAAACTCCTACTCTTCTGTAAGGAGGACAAGCAG
GTCATTTGCTGGCTTTGTGAGCGTTCTCAGGAGCACCGTGGACACTCCACATTCCTCTTGAAGGAGGTTGTAAAGG
AGTACCAGGAGAAGTTTGAGGAAATGTTGCAAAACCTGAAGAGATCCAGGGAAGAAGCTGAGGAGTGGAAAGT
TGACATCCAAACTGAGAGAACTTTCTGGAAGAACAAAATACAGAGTGAGGTGGAAAATGTCCAGATAGAGTTTGG
AAAACTGAGAGACATCCTCCAATCTGAGGAGGAGCAGGAGATTCAAAATCTGAAGAACGAGGAGGAAGTTATTA
TGAACAGCCTGACAGAGTCTGAAAGTGAGCTGATCCAGCAGAGCCAGGTGGTGAAAGATTTCA---
TCTCAGATCTGGAACATCGGTTGGAAGGGTCAACAGTTGAGATGCTGCA-GGATACGAATGATGTCATG-------
AGAA----------GGTGTAAGGACTTAATGCTGAGGAAGCCAAAAACTTTCCCC---
AAGGAACAAAGAAGAGTATTCCGAGCT

>Marmota_marmota_Trim5b

ATGGCTTCAGCAATCCTAGGGAATGTAAAGGAGGAGGTGACCTGCCCCATCTGCCTGGATCTCCTGACAAAACCT
CTGAGCATCGACTGTGGCCACAGCTTCTGCCAGGCCTGCATCACATCAAACTATGAG---TCCATGATGAGTCAAAA-
--
AGGGGAGAGCAGCTGCCCTGTGTGCAGAATCAGTTACGAGTTTGAGAACCTGCAGCCTAATCGGCATGTGGCCAA
CATAGTGGAGAGGCTCAGGGGCGTCAAGCTGAACCCAG---
AGGAGGAGCAGAAGGTGTATCACTGTGCACGCCATGGAGAGAAACTCCTACTCTTCTGTAAGGAGGACAAGACG
GTCATTTGCTGGCTTTGTGAGCGTTCTCAGGAGCACCGTGGACACTCCACATTCCTCTTGGAGGAGGTTGTCCAGG
AGTACCAGGAGAAGTTTCAGGAAATGTTGCAAAACCTGGTGATAGCCAAGGAAGAAGCTGAGAAGTGGAAAGTT
GACATCCAAACTGAGAGAACTTTCTGGAAGAGCAAAATACAGAGTGAGGTAGAAAATGTCCAGATATAGTTTGGA
AAATTGAGAGACATCCTGAATTCTGAGGAGGAGCAGGAGATTCAAAATCTGAAGAACGAGGAGGAAGTTATTAT
GAACAGCCTGACAGAGTCTGAAAGTGAGCTGATCCCGCAGAGCCAGTTGGTGAAAGATGTCA---
TCTCAGATCTGGAACATCGGTTGGGAGGGTCAACAGTTGAGATGCTGCA-G---
GTAAGACTTTCAGCTTATCTGGGCCCTGAAGTGCCTC---------------------ATTCCCA---

>Marmota_marmota_Trim6

ATGACTTCAACAGTTCTGGGGGACATCCGCGATGAGGTGACCTGCCCCCTTTGCCTGGAGCTCCTGACCGAACCCG
TGAGCACAGACTGTGGCCACAGCTTCTGCCAGGCCTGCATCACAGAGAATAGAGAGGAATCAGAGATGAGCCAG
GA---
CAAGGAGCGCAATTGTCCTGTGTGCCAGACCAGCTACCAGCCTAGGAACCTCCGGCCTAATCGGCACCTGGCCAA
CATAGCGCAGCGGCTCAGAGAGGTAGTGTTGTGCCCAG---
GGAAGCAGCTGCAGGTAATTCTTTGTGCACACCATGGAGAAAAACTCCAGCTCTTCTGTAAAGAGGATGGGAAGC
TAATTTGCTGGCTTTGCGAGCGTTCTCAGGAACACCGTGGCCACCATACATTTCTCATGGAGGAGGTCGCCCAGGA
GTACCAGGAGATGTTCCAGGAGTCTCTGAAGAAGTTGAGGAAAGAACAGCAGGAAGCTGAGAAGCTAAGAGCTT
TTATCAAAGAGAAGAGGGCATCCTGGAAGACTCAGATGGAGCCCGAGAGACAGAGGATCCAGACAGAGTTTAGT
CAGCTGCGAAGCATCCTGGACAAAGAGGAACAGCGGGAACTGAAGAAGCTGGAGGAGGAAGAAAGGAAGGGG
CTGAGCATTATAGAGAAAGCCGAGGAGGAGGTGATCTACCAGAGCCAGTCACTGGAAGAGCTTA---
TCTCAAATCTGGAGCATCGATGCCAGGGCTCAGTAATGGATCTGCTGCA-GGATGTGAGTGATGTCATG-------
CAGA----------GGAGTGAATTCTGGACCCTGAGGGACCCCCCAGCTCTCCCT---
ACCAAGCTGAGAAGTATGTTCCGAGCC

>Marmota_marmota_Trim34

ATGGCTTCAAAAACCCTGGTGAACACACAAGAAAAGGCAAAATGTCCCATCTGCCTAAAGCATTTAACAGAGCTAC
TGACTCTAGGCTGTGGTCACAGCTATTGCCAAACATGCATCTCTAGGAACAAGAAA---TCTGTGACCAGCCCAGG--
-
AGGGGAAAACAGGTGTTTCATATGTAGTATCAGGAACTTATTTGGAAATACCAAAGCTAATCAGCCTCCAGCTGAC
GTAGCAGAAGGACTCAGGGAAGTCAAGTTGAGCCCTA---
GCACTGTGCAAAATGCAGACTTATGTGCACGCCATGGAGAGAACCTCCTACTCTTCTGTGTGGAGGATAGGAAGG
TAATTTGCTGGCTTTGTGAGCTTTCTCAAGAGCACCGTGGGCACCGAACATTATTCATGGATGAAGTGACCAAGGA
ATGTCAGGACAAGCTCCAAGCAGTTCTCAAGAGGCTGAGGAAGGAACAAGAAGAAGCTGAGAAGTTGGAAGCTG
ATATCCAAGAAGAGATCACTTCTTGGAAGTTTCAGGTAGAGACTGAGAGAAAAAGGATACAAATGGAATTTAATC
ATCTTAGAAGAATCCTGGACAGCGAGGAAGAGCGAGAGCTACAAAGATTGGATCGGGAGGAGAAGACGACACT
GGATAGCTTGGCAGAAGCAGAGGATGAGCTGGTCTATCAGAAGCAGTTGGTGAAAGAGCTCC---
TCTCAGATCTAGAGCGTAGGAGTCAATGGTCAACAATGGAGCTACTGCA-GGACACAAGTGGAATCATG-------
AAAT----------GGAGTGAGATCTGGACTCTGAAGAAGCCAAAAACTGTCTCC---
AAGAAGCTGAAGACTGTATTCCGTGCT

>Meriones_unguiculatus_Trim6

ATGACGTCAGCCATCCTGGTGGACATCCGAGATGAAGTCACCTGCCCTATCTGCTTGGAGCTCCTGACAGAACCCC
TGAGCATAGACTGTGGTCACAGCTTCTGCCAGGCCTGCATCATGGGAAATAGTGACAAGTCAGTGCGCAGCCAAC
A---
AGGGAAGAGCAGCTGTCCGGTGTGCCGAACCACCTACCAGCCTGGGACCCTCCGGCCTAATCGACACCTGGCCGT
CATAGTGAAGAGGCTTAAGGAGGTCATGCTGGGCCCCG---
GAAAGCAGCTCGAGGTCATTGTTTGTGCGCTTCATGGAGAAAAACTCCAGCTCTTTTGCAAGGAGGATGGGAACC
TAATTTGCTGGCTTTGTGAGCGGTCTCAGGAGCACCGTGGTCATCACACATTCCTCATGGAGGAGGTGGCCCAGG
AGTACCAGGAAATGTTCCAGGAGTCTCTGAAGAAGCTGAGGAAAGAGCAGCAGGAAACTGAGAGGCTGAAAGCT
CTTATCCGAGAGAAGAGGGAGTCCTGGAAGAATCAGGTGGAGCCTGAGAGACGCCGGATCCAGACAGAGTTCAA
GCAGCTTCGAAGCATCCTGGACAGGGAGGAGCAGCGGGAACTGAAAAAGCTGGAAGTGGAAGAGAAGAAGGG
GCTGAGCATCATAGACAAGGCCGAGGGTGACCTGACTCATCAGAGCCAGTCACTGAAAGAGCTCA---
TCTCAGACCTGGAGCACCGGTGCCAGGGCTCCACGCTGGAGCTGCTGCA-GGATGTGAGCGATGTCACA-------
AAAA----------GGAGTGAGTTCTGGACCCTGAGGAAGCCCAACCTCTCCCC---
ACCAAGCTGAAAAGTTTGTTTCGAGCC

>Meriones_unguiculatus_Trim12

ATGGCTTCAGAATTCATGGTAAATTTAAAGGAGGAGGTGACCTGTCCCATCTGTCTGGACCTGATGGTGGAACCC
GTGAGTGCAGATTGTGGTCACAGCTTCTGCCGAGCCTGCATCACACTGAACTATGAG---
TCCAGCAAAAGCAAAGA---
GGAAGAGTTCATCTGTCCTGTGTGCCGAGTTAGTTACCTGTTTGGGAATCTGAGGCCTAATCGACATGTGGCCAAC
ATAGTGGAGAGGCTCAAGGGCTTCAAGTCCAGCCCAG------
AAGAGCAGAAGGTGTTTCACTGTGCAAGGCACGGAGAGAAACTCCAGCTCTTCTGTGAGAAGGACAAGGTGGCC
ATCTGCTGGATTTGTGAGCGATCTCAGGAGCACCGTGGTCACCAAACAGCTCTCATTGAGGAGGTGGCCTGTGAG
TACAAGGAGAAGCTGCAGGCAGCTCTGCAGAAGCTGATGGCAGACAAGAAAGAATCTGAGAACTGGAAAGATG
ACCTCCAACAGGAGAGAACTTATTGGGAGAGTAAAATACAGAAAGATGTAGAAAACGTTCAGACAGAGTTTAAAC
GAATGGAAGACATCCTGGACTCTGAGAAGAAGAATGAATTGCAGAAGCTGATGCAAGAAAAGGAAGACATTATT
AACAGCCTGGCAGAGTCTGAAAATGAGCATGCCCAGCAGAGCAAGTTGCTAGGAGACCTCA---
TCTCAGATGTGGAGCATCAGTTGGGGTGCTCAGCCATGGAAATGCTGCA-GGGAGTAGACAGCATCATA-------

AACC----------GGAGTCATACTTTCTCGCTGACAAAGCCCAAAACCATCCCC---
AAGGAACAAAGAAGAGTGTTCCGAGCC

>Meriones_unguiculatus_Trim30

ATGGCCTCATCAATCCTGGCGAAGATAAAGGAGGAGGTGACCTGTCCCATCTGTCTGGAGCTCCTGAAGGAACCT
GTGAGTGCAGACTGTGGTCACAGCTTCTGCCGAGCCTGCATCACACTGAACTATGAG---
TCCAGCACAGGCAAAGA---
AGGGGAGGGCAGCTGCCCTGTGTGCCGAGTTAGTTACCTGTTTGGAAATCTGAGGCCCAATCGACATGCAGCCAA
TATAGTGGAGAGTCTCAAAGGGTTTAAGTCCATTCCTG---
AGGAGGAGCAGAAGGTAAATGTCTGTGAACAACATGGAGAGAAGCTCCAGCTCTTCTGTGAGAATGACATGACA
GCCATCTGCTGGCTTTGTGAGCGATCTCAGGATCATCGTGGTCACCACACAGCTCTCATTGAAGAGGCTGCTGAAA
AGTACAAGGGGAAGCTGCAGGCAGCTCTGCAGACACTGATGGCGAATGAAAAAACATGTGATGAATGGCAAGAT
GACCTCCAACAGGAGAGAACTTACTGGGAGGATCAAATACAGAGTGATGTAGACAATGTTCAGAAGGAATTTGA
AGGACTAAGAGACGTCCTGGCCTCCAAGGAGAATGAAGAGCTGCGGAAGCTGATGGAAGAGAAGGAAGATATT
GTACAAAGGCTGGAGAAGTCTGAAAATGAGCTCTCCAGGCAGAGGGAGTCAGTGAGAGACTGCA---
TCTCAGATGTGGAACATCATTTGGAGAGCTCAACCATGGAAATGCTGCA-GAATGTGAATTGTGTCCTA-------
AAAA----------GGAGTAAAGCTTTGAAACTGCAACAGCCTGAAATGATCCAG---AAAAAAAAAAGA---
ATATTCCATGTG

>Meriones_unguiculatus_Trim34

ATGTCTTCAGCAGTTCTGGTGAAGGTACAGAAGGATGCCGCTTGCCCTGTCTGCCGAGAGCTTTTGACAGAACCCC
TGAGTCTAGGCTGTGGCCACCATGTGTGCCAAGCCTGCATCACCAGGAACAAGACC---AAAGTAATCAACCCCAG-
--AGAGAAAAGCAGCTGTCCTGTGTGTGGTACCAGATTCTCAT--------------
CTAATCAGCCTCTGGCAGATGCAGTAGAGAGACTCAGGGAAGTCAAATTGAACCCTG---
ATGTTGGGGAAAAGGGAGATCTCTGTGCACAGCATGGAGAGAAACTCCTTCTTTTCTGTAAGGAAGACAAAAAAG
TCATTTGCTGGGTTTGTGAGCGTTCCCGGGAGCACCGTGGTCACCACACCTTCCTCCGGGAGGATGCAGTCAAGG
AATGTCAGGAGAATCTCCAGAACACTCTCAAGAGGCTGAGGAAGGAGAAGGGGAAGGTGGAGAAATTGGAAGC
TGACATCAAAGAAGACAGAATTTCCTGGAAGTGCCAGATCCAGACCGAAAAACAAAGGATACAAACGGGCTTTAA
CCAGCTTAGAAGAGTCCTGGACAAGGAGGAACAGAGAGAGCTGAGCAGACTGTGCGAAGAGGAGCACATGATA
CTGGACAACCTGGCAGAGGCGGAGGCTGAGCTGGCTCAGCAGAGCCAGTTGGTGGAGGATCTTA---
TCTCTGATCTAGAGAATCGGTGCCGGTGGTCAGAGACAGATCTGCTGCA-GGATATGAGCGGTATCTTG-------
AAAT----------GGAGTCAGATCTGGACACTGAAGAAGCCGAAAGCCATTTCC---
AAGAAACTGAAGACGGTATTCCAAGCT

>Microtus_ochrogaster_Trim6

ATGACAACCGTGCTTCTGGAGGACATCCGAGAGGAAGTCACCTGCCCTATCTGCTTGGAGCTCCTGACAGAACCCC
TGAGCATAGACTGTGGTCACAGCTTCTGCCAGGCCTGCATTACAGGGTACAGTCACAGGTCAGTGCTCAACCAAG
A---
AGGGAAGAGCATCTGTCCTGTGTGCCAGACCACCTACCAGCCTGGGAACCTCCGCCCTAACCGACACCTGGCCAC
CATAGTGAAGAGGCTCAAAGAGATCGTGTTGGGCCCGG---
GAAAGCAGCCAGAGGTCATTCTTTGTGCTCTTCATGGAGAGAAACTCCAGCTTTTTTGCAAGGAGGATGGGAAGT
TAATTTGCTGGCTTTGTGAGCGATCTCCGGAGCACCGTGGTCACCACACATTCCTCATGGAGGAGGTGGCTCAGG
AGTACCAGGAACTGTTCCAGGAGTCTCTGAAGAAGCTGAGGAAGGAGCAGCAGGAAGCTGAGAACCTAAAAGTT
CTTATCAAAGAGAAGAGGGAATCCTGGAAGAGTCAGGTGGAGCCTGAGAGACACCGGATCCAGACCGAATTTAA

GCAGCTGCGGAGCATCCTGGACCGAGAGGAGCAGAGGGAACTGAAGAAACTGGAAATAGAAGAGAGGAAGGG
GCTGAGCATCATAGAGAAGGCCGAGGGGGACCTGATCCACCAGAGCCAGTCACTAAAGGATCTCA---
TCTCCGACCTTGAGCACCGCTGCCAGGGGTCCACAGTGGAGCTGCTGCA-GGATGTGAGTGATGTCACA-------
AAAA----------GGAGTGAGTTCTGGACCCTGAGGAAGCCCCAAACTCTACCC---
ACCAAGCTCAGAAGTTTGTTCCGAGCC

>Microtus_ochrogaster_Trim12

ATGGCTTCAGAATTCATGATGAATGTCAAGGAGGAGGTGACCTGTCCTATCTGTCTGGACCTCATGGTAGAGCCTG
TGAGTGCAGATTGTGGTCACAGCTTCTGCCGAGCCTGCATCACACTGAACTATGAG---TCGAGCAAAGGCAAAGA-
--
GGGTGAGTTCATCTGTCCTGTGTGCCGAGTGAGTTACCTGTTTGGTGATCTGAGGCCTAATCGGCATGTGGCCAAC
ATAGTGGAGAGGCTCAAGGAGTTCAAGTCCAGCTCAG---
GGGAGGAGCAGAAGGTGAATGTCTGTGCAAAGCATGGAGAGAAACTCCAGCTCTTCTGTGAGAAGGACAAGGTG
GCCATCTGCTGGCTTTGTGAGCGATCTCAGGAGCACCGAGGACACCAAACAGCTCTCATTGAAGAGGCGGCCCAT
GAGTACAAGGGGAAGCTCCAGGTGTCTCTGCAAAAGCTGATGTCAGACAAGAAAGAATTAAAGAGCTGGGAAGA
TGACCTTCAAAAGGAGAGAACTTCCTGGGAGAATCAAATACAGAAAGATGTTGAAAACGTTCAGACAGAATTTAG
AAGACTAAGAGACACCCTGGACTCTGAAGAGAAGAATGAGCTGCAGAAGCTGACGCAAGAGAGGGAAGACATTC
TGAGCAACCTGGCAGAGTCTGAAAGTAAGCATGCCCAACAGAGCAAGTTGCTAGGAGACCTCA---
TCTCAGATGTGGAACGTCAGCTGCAGTGCTCAGCCATGGAGATGCTGCA-GGGAGTGGATGACATCATA-------
AAAT----------GGAGTCAGGCCTTTTCACTGACGAAGCCCAAAGCCATCCCC---
AAGAAACGAAGACGAGTGTTCCGAGCC

>Microtus_ochrogaster_Trim30a

ATGGCCTCATCAGTCCTGGGGATGGTCAAGGAGGAGGTGACCTGTCCTATCTGTCTGGACCTCATGGTAGAGCCT
GTGAGTGCAGATTGTGGTCACAGCTTCTGCCGAGCCTGCATCACACTGAACTATGAG---
TCCAGCAAAGACAAAGA---
AGGGGAGGGCATCTGCCCTGTGTGCCGAGTTAGTTACCTGTTTGGGAATCTGAGGCCTAATTGGCATGTGGCCAA
CATAGTGGAGAGGCTAACAGGTTTCAAGTCCAGCTCTG---
GGGAGGAGCAGAAGGTGAATGTCTGTGCAAAGCATGGAGAGAAACTCCAGCTCTTCTGTGAGAAGGACATGATG
ATTATCTGCTGGCTTTGTGAGAGATCTCAGGATCACCGTGGTCACCAAACAGCTCTCATTGAAGAGGTGGCCACTA
AATACAAGG---
GAGTGACAGGGAGTCCAT--
-------------------------GGTGGTCAGAGTTCACACTGTCTGAGATGTGGAG---------------------TCAATCTTTTGTC-----
-----------CCTA--------------------GGAGTCAGACCTTATGTCTGAAACAACCCAAAATGATTCCA---
AGAAAACAGAGTAAGATCTTCCGAGCT

>Microtus_ochrogaster_Trim30b

ATGGCCTCATCAGTCCTGGGGATGGTCAAGGAGGAGGTGACCTGTCCTATCTGTCTGGACCTCATGGTGGAGCCT
GTGAGTGCAGATTGTGGTCACAGCTTCTGCCGAGCCTGCATCACACTGAACTATGAG---
TCCAACAAACACGAAGA---
AGGAGAGAGCATCTGCCCTGTATGTCGAGATAGTTACCTGTTTGGGAATCTGAGGCCTAATTGGCATTTGGCCAAC
ATAGTGGAGAGGCTAACAGGGTTCAAGACAAGCTCAG---
GGGAGGAGCAGAAGGTGAATGTCTGTGCACAACATGGAGAGAAACTCCAGCTCTTCTGTGAGAAGGACATGATG
GCCATCTGCTGGCTTTGTGAGAGATCTCAGGAGTACTGTGGTCACCATACAGTTCTCATTGAAGAGGTGGCCACTA

AATACAAGGCGATGCTCCAGTCTGCCCTGGAGATGCAGATGGCTAATGAGGAAAGATGTGACCAGTGGGAAGAT
GACCTTCAAAAGGAGAGAACTTTCTGGGAGAAAAAAATACAGAGCAATGTAGAAAAGGTCCAGAAGAAGTTTAA
AGAAATGCACGAATTCCTGTATTCGGAGGAGAAGAATAAGCTGCAGAAGCTGAGGCAAAAGGAGGAAGACATTG
TCAAAAGACTGGAAAAGTCTGAAAATGATCTGGTGAAGCAGAGGGAGTCAGTGAGTGACCTCA---
TCTCAGATCTGGAGCATGGGTTGCAGTGCTCATCCATAGAGATGCTGCA-GGGTGTGAATCATGTCCTA-------
ACAA----------GGAGTCAGACCTTAAGTCTGAAACAGCCCAAAATGATTCCA---
AGAAAACAGAGAAAGATCTTCCGAGCT

>Microtus_ochrogaster_Trim30c

ATGGCCTCATTAGTACTGGGGATGATCAAGGAGGAGGTGACCTGTCCTATCTGCCTGGAGCTTCTGAAAGAACCT
GTGAGTGCCGATTGTGACCACAGCTTCTGCCGAGCCTGCATCACTCTGAACTATGAG---
TCCAGCAAAGGCAAAGA---
AGGTGAAGGTACCTGCCCTGTGTGCCGAGTTACTTATATGTTTGAGAATCTGAGGCCTAATCAGCATGTGACCAAC
ATAGTGGAGAGGCTCAAGGAGTTCAAGTCTAGCCCAG---
AGGAGGAACAGAAGGTCAATGTCTGTGCACAACATGGAGAGAAACTCCAGCTCTTCTGTGAGAAGGACATGGTG
GCCATCTGCTGGCTTTGTGAGAGATCTCAGGAGCACCGTGGTCATCAAACAGCTCTCATTGAAGAGGTGGCCAAT
AAGTACAAGGGGATGCTCCAGGCTGCCCTGAAGATGCAGATGGCTAATGAGGAAAGATGTGACCAGTGGGAAGA
CGACCTTCAAAAGGAGAAAGTTTCCTGGGAGAACCAAATACAGAGTGAGGTAGAAAACGTTGAGAAGGAGTTTA
AAGGACTGTGGGAATTCCTGAAAACTGAGGAGACTAATGAGCTGCAGAAGCTGATGAAAGAGAAGGAAGACATT
CTGGACATCTTGGAAGATTCTCAAAATGAGCTGAAGAAGCAGAGGGAGTCAGTGAGAGACCTCA---
TCTCAGATCTGGAGCATAGCTTGCAGTGCTCAACCATGGAGATGCTGCA-GGGTGTGATTTGTGTTTTA-------
ACAA----------GGAGTCAGTACTTAAGACTGAAACAGCCTAAAATGGTCCCG---
AGAAGAAAGAGAAAGATCTTCAGAGCT

>Microtus_ochrogaster_Trim30d

ATGACCTCATCAGTCCTGGAGATGATCAAGGAGGAGGTGACCTGTCCTATCTGCCTGGAGCTTCTGAAAGAACCT
GTGAGTGCCGATTGTAACCACATCTTCTGCCGAGCCTGCATCACTCTGAACTATGAG---
TCCAGCAAAGGCAAAGA---
AGGGGAGGGCATCTGCCCTGTGTGCCGAGAATGTTACCTGTTTGGGAATCTGAGGCCTAATTGCCATGTGGCCAA
CATAGTGGAGAGGCTCAAGGAGTTCAAGTCCAGCTCAG---
GGGAGGAGCAGAAGGTGAATGTCTGTATACAACATGGAGAGAAACTCCAGCTCTTCTGTGAGAAGGACATGGTG
GCCATCTGCTGGCTTTGTGAGAGATCTCAGGAGCACCGTGGTCACCAAACAGCTCTCATTGAAGAGGTGGCCAGT
AAGTACAAGGGGAAGCTCCAGGCTGCCCTGAAGATGCAGATGGCTAATGAGGAAAGATGTGACCAGTGGGAAG
ATGACCTTCAAAAGGAGAGAACTTTCTGGGAGAACCAAATACAGAGTGAGGTAGAAAACGTTGAGATGGAGTTG
AAAGGACTGCGGGAATTCCTGGACTCCAAGGAGAAGAATGAGGTTGAGAAGCTGATGAAAGAGAAGGAAGACA
CTATGAACATCCTGGAAGGTTCTCAAATTGAGCTGGTGAAGCAGAGGGAGTCAGTGAGAGACCTCA---
TCTCAGATCTGGAGCATGGGTTGCAGTGTTCAACCATGGAGATGCTGCA-GGGTGTGAATCATGTCCTA-------
ACAA----------GGAGTCAGAACTTAAGACTGAAACAGCCCAAAATGGTCCCA---
AGTAAACAGAGAAAGATCTTCCGAGCT

>Microtus_ochrogaster_Trim30e

ATGGCCTCATCAGTCCTGGGGATGGTCAAGGAGGAGGTGACCTGTCCTATCTGTCTAGAGCTTCTGAAGGAACCT
GTGAGTGCCGATTGTGACCACAGCTTCTGCCGAGCCTGCATCACTCTGAACTATGAG---
TCCAGCAAGGGCAAAGA---

AGGGGAGGGCGTCTGCCCTGTGTGCCGAGTGTGTTACCTGTTTGGGAATCTGAGGCCTATTCGACATGTGGCCAA
CATAGTGGAGAGGCTCAAGGAGTTCAAGTCCAGCCCAG---
AGGAGGAGCAGAAGGTCAATGTCTGTGCAAAGCATGGAGAGAAACTCCAGCTCTTCTGTGAGAAGGACATGGTG
GCCATCTGCTGGCTTTGTGAGAGATCTCAGGAGCACCGTGATCACCAAACAGCTCTCATTGAAGAGGTGGCCAAT
AAGTACAAGGGGAAGCTCCAGGCTGCCCTGGAGATGCAGATGGCTAATGAGGAAAGATGTGACCAGTGGGAAG
ACGACCTTCTAAAGGAGAGAACTTCCTGGGAGAAGCAAATACAGAGGGATGTAGAAAAAGTTCAGAAGGAGTTT
AAAGGACTGCGGGACTTCCTGGACTCCATGGAGAAGAATGAGGTGGAGAAGCTGATGAAAGAGAAGGAAATCA
TTCTGTATATCCTCAAAGATTCTCAAATTGACATGGAGTTCCAAAGGAAGTCAGTGAGAGAACTCA---
TCTCAGATCTGGAGCATCGGCTGCAGTGCTCAACCATAGAGATGCTGCA-GGGTGTGAATTGCATTTTA-------
ACAA----------GGAGTCAGAACTTAAGACTGAAACAGCCCCAAATGGTCGCA---
AGTAAACAGAGAAAGACCTGCCGAGAC

>Microtus_ochrogaster_Trim30f

ATGACCTCATCAGTCCTGGGGATGATCAAGGAGGAGGTGACCTGTCCTATCTGTCTGGACCTCATGGTGGAGCCT
GTGAATGCAGACTGTGGTCACAGCTTCTGCAGAGCTTGCATCACACTGAACTATGAG---TCCATCAAAATCAAAAA-
--
AGGGGAGGGTGTCTGCCCTGTGTGCCAAGTTACTTACCTGTTTGGGAATCTGAGGCCTAATTGGCATGTGGCCAA
CATAGTGGAGAGGCTAACAGGGCTCAAGACAAGCTCAG---
GGGAGGAGCAGAAGGTGAATGTCTGTGCAAAGCATGGAGAGAAACTCCAGCTCTTCTGTGAGAAGGACATGATG
GCCATCTGCTGGCTTTGTGAGAGATCTCAGGAGCACCGTGGTCACCAAACAGCTCTCATTGAAGAGGTGGCCAAT
AAGTACAAGGAGATGCTTCAGGCTGCCCTGGAGATGCAGATGGCTAATGAGAAAAGATGTGACCAGTGGGAAGA
CGACCTTCAACAAGAGAGAACTTCCTGGGAGAACCAAATACAGAGTGAGGTAGGAAACGTTCAGAAGGAGTTTA
AAGGACTGCGGGAATTCCTGGACTCCAAGGAGATGAATGAAGTGGAAAAGCTGATGCAAGAGAAGGAAGACAC
TATGAACAGCCTGGCAGTGTCTCAAAATGAGCTGGTGAAGCAGAAGGAGTCAGTGAGAGACCTCA---
TCTCAGATCTGGAGCAGCGGTTGCAGTGCTCAACCATAGAGATGCTACA-GGGTGTGAATAGTGTCCTA-------
ACAA----------GGAGTCAGAACTTAAGACTGAAACAGCCCAAAACGGTCCCA---
AGTAAACAGAGAAAGATCTTCCGAGCT

>Microtus_ochrogaster_Trim34

ATGGCTTCGACAGTTCCGATGAATGCAGAGAAGGGGGCCATTTGCCCTGTCTGCCATGAACTTTTGAAAGAACCC
ACGAGTCTAGGCTGTGGCCACATTGCATGCAAAGCCTGCATCACCACAAACAAGAAGAATGCAGTGATCAACCCC
AG---
AGGGAGAAGCAGCTGTCCTGTGTGTGGTACTAGATTCTCATTTGAAAATCTACAGGTTAATCAACATCTGGCAAAC
GTAGTAGAGAGACTTAGGGAAGTCAAGGTGAACCCTG---
ACATTGGGGAAAAAAGAGATCTCTGTATCCACCATGGAGAGAAACTCCTTCTCTTCTGTAAGGAAGATAGAAAGA
TCATTTGCTGGGTTTGTGAGCGTTCTCAGGAGCATCGTGGTCACCACACTTTCCTCCGGGAGGAAGCAGTCAAGGA
ATGTCAGGAGAATATCCAGAAAGCTCTCGAGAGGCTGAGGAAGGAACAGGAGAAGGCGAAGAAATTGGAAGCT
GACATTGAAGAAGACAGAATTTCCTGGAAGTGCCAGATCCAGACTGAGAGACAAAGGATACAAACAGGTTTTGAT
CAGCTTAGAAGAATCCTGGACGAGGAGGAGCAGAGAGAGCTGAAAAGACTTGGGGAAGAGGAGCAGCTGATAC
TCGACAGCCTGGCGGAGGCAGAAGCTGAGCTGGCTCAACAGAGCCAGTTGGTCCAGGAACTTA---
TTTCCGGTCTGGAGCTTCGGTGCCAGTGGCCAGTGACAGAGCTGCTGCA-GGATATGAGCGGTACCTTG-------
AAAT----------GGAGTCAGATCTGGACGTTGAAGAAGCCAAAAGCGGTTTCT---
AGGAAAGTGAAGAAGGTATTCCAAGCT

>Mus_musculus_Trim5

ATGGCTTCACAATTCATGAAGAATTTAAAGGAGGAGGTGACCTGTCCTGTCTGTCTGAACCTGATGGTGAAACCTG
TGAGTGCAGATTGTGGTCACACCTTCTGCCAAGGCTGCATCACGTTGTACTTTGAA---TCCATCAAATGTGATAA---
GGAAATGTTCAGTTGCCCTGTGTGCCGACTTAGTTACCAGTCTAGCAATCTGAGGCCTAATCTACATGTGGCCAAC
ATAGTAGAGAGGCTCAAAGAGTTCAAGCCCAGCCCAG---
AAGAGGAGCAGAAAGTGTTTAACTGTGCAAGACATGGAGAGAAACTCCAGCTCTTCTGTAGGAAGGACATGATG
GCCATCTGCTGGCTTTGTGAGCGATCTCAGGAGCACCGGGGCCACAAAACAGCTCTCATTGAAGAGGTGGCCCGG
GAGTACAAGGAGCAGCTGCAGGTAGTTCTGCAAAGGCTGATGGCAGATAAGAAAGAATTTGAAAACTGGAAAGA
TGAACTTCAGAAGGATAGAACTTACTGGGAGAATCAAATACAGAAAGATGTGGAGAATGTTCAGTCAGAGTTTAA
ACGAATGAGGGATATCATAGACTCAGAGGAGAAGAATGAATTGCAGAAGCTGAGGCAAGAGAAGGAAGACATT
CTCAACAACCTGGCAGAGTCTGAAAGTGAGCATGCTCAGCAGAGCAAGTTGCTAGAAGACTTCA---
TCTCAGATGTGGAACATCAGTTACAGTGCTCAGACATAGAAATACTACA-GGGTGTGGAGAACATCATA-------
AAAC----------GGAGTCTTACTTTTTCGATGAAGAAGCCCAAAACCATCGCC---AGGGAACAAAGAAA---
GTTCCGAGCC

>Mus_musculus_Trim6

ATGACTTCAACAGTCTTGGTGGACATCCGAGATGAAGTAACCTGCCCTATCTGCTTGGAGCTCCTGACAGAACCCC
TGAGCATTGATTGTGGCCATAGCTTCTGCCAGGTCTGCATCATAGGAAACAGTAATAATTCAGTGTTCGGCCAAGG

AGGGAGGAGCAGCTGTCCTGTGTGCCGGACCTCCTATCAGCCTGGGAACCTCCGTCCTAATCGGCACCTGGCCGC
CATAGTGAAGAGGCTCAGAGAGGTTGCGTTGTGCCCTG---
GAAAACAACTCGAGGTCATTTTTTGTGCGCTTCATGGAGAGAAACTCCAGCTCTTTTGCAAGGAGGATGGGAAGT
TAATTTGCTGGCTTTGTGAACGATCTCAGGAGCACCGTGGTCATCACACATTCCTCATGGAGGAGGTGGCCCAGG
AGTACCAGGACATGTTCCAGGAGTCTCTGAAGAAATTGAGGAGGGAGCAGCAGGAAGCCGAGAAGCTAAAAGCT
CTTATCCAGGAGAAGAGGGAATCCTGGAAGAGTCAGGTGGAGCCTGAGAAACGCCGGATCCAGACAGAGTTTAA
GCAGCTCCGAAGCATCCTGGACAGGGAGGAGCAGCGGGAACTGAAGAAACTGGAAGTGGAAGAGAGGAAGGG
GCTGAGCATCATAGAAAAGGCCGAGGGTGACCTGATCCACCAGAGCCAGTCACTGAAGGACCTCA---
TCTCAGACCTGGAGCACCGGTGCCAGGGGTCCACCGTAGAACTGCTGCA-GGATGTGGGTGATGTTACA-------
AAAA----------GGAGTGAGTTCTGGACCCTGAGGAAGCCCCAAGCTCTCCCC---
ACCAAGCTGAAAAGTTTGTTTCGAGCA

>Mus_musculus_Trim12a

ATGGCTTCACAATTCATGAAGAATTTAAAGGAGGAGGTGACCTGTCCTGTCTGTCTGAACCTGATGGTGAAACCTG
TGAGTGCAGATTGTGGTCACACCTTCTGCCAAGGCTGCATCACGTTGTACTTTGAA---TCCATCAAATGCGATAA---
GAAAGTGTTCATTTGCCCTGTGTGCCGAATTAGTTACCAGTTTAGCAATCTGAGGCCTAATCGAAATGTGGCCAAC
ATAGTAGAGAGGCTCAAAATGTTCAAGCCCAGCCCAG---
AAGAGGAGCAGAAAGTGTTTAACTGTGCAAGACATGGAAAGAAACTCCAGCTCTTCTGTAGGAAGGACATGATG
GCCATCTGCTGGCTTTGTGAGCGATCTCAGGAGCACCGTGGTCACAAAACAGCTCTCATTGAAGAGGTGGCCCAG
GAGTACAAGGAGCAGCTGCAGGTAGTTCTGCAAAGGCTGATGGCAGATAAGAAAAAATTTGAAAACTGGAAAGA
TGACCTTCAGAAGGATAGAACTTACTGGGAGAATCAAATACAGAAAGATGTGCAGAATGTTCGGTCAGAGTTTAA
ACGAATGAGGGATATCATGGACTCTGAGGAGAAGAAGGAATTGCAGAAGCTGAGGCAAGAGAAGGAAGACATT
CTCAACAACCTGGCAGAGTCTGAAAGTGAGCATGCTCAGCAGAGCAAGTTGCTAGAAGACTTCA---
TCTCAGATGTGGAACATCAGTTACAGTGCTCAGACATAGAAATACTGCA-GGGTGTGGAGAACATCATA-------
GAAC----------GGAGTCATACTTTTTCGATGAAGAAGCCCAAAGCCATCGCC---AGGGAACAAAGAAA---
GTTCCGAGCC

>Mus_musculus_Trim12c

ATGGCTTCACAATTCATGAAGAATTTAAAGGAGGAAGTGACCTGTCCTCTCTGTCTGAACCTGATGGTGAAACCTG
TGAGTGCAGATTGTGGTCACAGCTTCTGCCAAGGCTGCATCACGGTGTACTTTGAA---TCCACCAAATGCGATAA---
GGAAATGTTCAGTTGCCCTGTGTGCCGACTTAGTTACCAGTCTAGCAATCTGAGGCCTAATCTACATGTGGCCAAC
ATAGTAGAGAGGCTCAAAGAGTTCAAGCCTAGCCCAG---
AAGAGGAGCAGAAAGTGTTTAACTGTGCAAGACATGGAGAGAAACTCCAGCTCTTCTGTAGGAAGGACATGATG
GCCATCTGCTGGCTTTGTGAGCGATCTCAGGAGCACCGGGGCCACAAAACAGCTCTCATTGAAGAGGTGGCCCAG
GAGTACAAGGAGCAGCTGCAGGTAGTTCTGCAAAGGCTGATGGCAGACAAGAAAGAATTTGAAAACTGGAAAGA
TGACCTTCAGAAGGATAGAACTTACTGGGAGAATCAAATACAGAAAGATGTGGAGAATGTTCAGTCAGAGTTTAA
ACGAATGAGGGATATCATGAACTCTGAAGAGAAGAAGGAATTGCAGAAGCTGAAGCAAGAGAAGGAAAACATT
ATCAACAAACTGGCAGAGTCTGAAAATGAGCATGCTCAGCAGAGCAAGTTGCTAGAAGACTTCA---
TTTCAGATGTGGAACATCAGTTACAGTGCTCAGACATAGAAATACTGCA-GGGTGTGGAGAACATCATA-------
AAAC----------GGAGTCATACTTTTTCGATGAAGAAGCCCAAAGCCATCGCC---AGGGAACAAAGAAA---
GTTCCGAGCC

>Mus_musculus_Trim30a 1

ATGGCCTCATCAGTCCTGGAGATGATAAAGGAGGAAGTAACCTGTCCTATCTGTTTGGAGCTCCTGAAGGAACCT
GTGAGTGCTGATTGTAACCACAGCTTCTGCAGAGCCTGCATCACACTGAATTATGAG---
TCCAACAGAAACACAGA---
CGGGAAGGGCAACTGCCCTGTATGCCGAGTTCCTTACCCATTTGGGAATCTGAGGCCTAATCTACATGTGGCCAAC
ATAGTAGAGAGGCTCAAGGGATTCAAGTCCATTCCAG---
AGGAGGAGCAGAAGGTGAATATCTGTGCACAACATGGAGAGAAACTCCGGCTCTTCTGTAGGAAGGACATGATG
GTCATCTGCTGGCTTTGTGAGCGATCTCAGGAGCACCGTGGTCACCAAACAGCTCTCATTGAAGAGGTTGACCAA
GAATACAAGGAGAAGCTGCAGGGAGCTCTGTGGAAGCTGATGAAAAAGGCAAAAATATGTGATGAATGGCAGG
ATGACCTTCAACTGCAGAGAGTTGACTGGGAGAACCAAATACAGATCAATGTAGAAAATGTTCAGAGACAGTTTA
AAGGACTAAGAGACCTCCTGGACTCCAAGGAGAATGAGGAGCTGCAGAAGCTGAAGAAAGAGAAGAAAGAGGT
TATGGAAAAGCTGGAAGAGTCTGAAAATGAGCTGGAGGATCAGACAGAGTTGGTGAGAGACCTCA---
TCTCAGATGTGGAACATCATTTGGAGCTCTCAACCTTAGAAATGCTGCA-GGGTGCAAATTGTGTCCTG-------
AGAA----------GGAGTCAGTCCTTAAGCCTGCAACAGCCCCAAACTGTCCCC---
CAAAAGAGAAAAAGAACATTCCAAGCT

>Mus_musculus_Trim30c

ATGGCCTCCTCAGCTCTGGCAATGGCAAAGGAAGAGGTGACCTGTCCAATCTGTCTGGAGCTTCTGAAGGAACCT
GTGAGCACTGATTGTGGTCACAGCTTCTGCCAAACCTGTATCATACTGAACTATGTG---TCCAACAGAAGAATGGA-
--
TGGGGTAGGCAGCTGCCCTGTGTGCCGGGTTGGTTACCTGTTTGAGAATCTGAGACCTAATCAAAACATGATCAA
CATAGTAAAAAGGATCAAAGAGTTGAAGTCTATCCCAG---
AGGAGAAGGAGAAAGTGTTTTACTGTGCACAACATGGAGAGAAACTCAAGCTCTTCTGTAAGGAGGACAGGATG
GCCATCTGCTGGGTTTGTGAGAGATCTCAGAAGCACCGTGGTCACCAGACAGCTCTCATTGAAGAGGTGGACCAG
GAGTACAAGGAGAAGCTGCAGGCAGCTCTGAAGAAGCTGATGGAAAATGAAAAAAGATGTGATAAATGGCAGG
ATGACCTTCAGCAACAGAGAGCTGACTGGGAGAATCAAATACAGCGTGATGTGGAATATGTTCAGATGGAGCTTA
AAGGACTAAGAGACCTCCTGGACTCCAAGGAGAATGAGGGGCTGCAGGAGCTGAAGAAAGAGAAGGAAGAGGT
TATGGAAAAGCTGGAAGAGTCTGAAAATGAGCTAAGGGAGCAGACAGAGTTGGTGAGAGACCTCA---
TCTCAGATGTGGGACATCAGTTGGCGCTCTCAACCATGGAAATGCTGCA-GGGCATGAATTCTGTTCTA-------

ACAA----------GGATTCAGGTCCTGAGACTGAAACAGCCTCAAACTATCCCC---
CCAAAAAGAAGAAGAACATACCAAGTT

>Mus_musculus_Trim30d

ATGGCCTCATCAGTCCTGGAGATGATAAAGGAAGAAGTGACCTGCCCAATCTGTCTGGAGCTCTTGAAGGAATCT
GTGAGTGCTGACTGTAACCACAGCTTCTGCAGAGCCTGCATCACACTGCACTATGAG---
TCTAACAGAAACCCAGA---
AGGGGAGGGCAACTGCCCTGTGTGCCGAGTTCCTTACCTGTTTGAGAATCTGAGGCCTAATCGACATGTTGCCAAC
ATAGTAGAGAGGCTCAAGGGATTCAAGTCCATCCCAG---
AGGAGGAGCAGAAGGTTAATGCCTGTGTAGAACATGGAGAGAAACTCCAGCTCTTCTGTAAGGAGGACATGATG
GCCATCTGCTGGCTTTGTAAGCAATCCCAGGAGCACAGTGGTCACCAAACAGCTCTCATTGAAGAGGTCAACCATG
AATACAAGGAGAAGCTACAGGCAGCTCTGCAGAAGCTGATGGAAAATGAGAAAAGATGTGATGAATGGCAGGAT
GACCTTCAGCAACAGAGAGCTGACTGGGAGAACCAAATACACAGTGATATAGAAGACATTCAGATAGAGTTTAAT
GGACTAAGAGACCTCCTAGACTCCAAGGAGAATGAGAAGCTGCAGGAGCTGAAGAAAGAGAAGGAAGAGGTTA
TGGAAAAGCTGGAAGAGTCTGAACATGAGCTAAGGGAGCAGAGGGAGTTGGTGAGAGACCTCA---
TCTCATATGTTCAGCATCAGTTGGAGCTGTCAGTCATGGAAATGAAGCA-GGGTGTAAATTATGTCCTG-------
ACAA----------
GCATTCAGACCATGACACTGAAGCAGCCCCAAATAGTTCCCCCAAAAAGAAGAAGAGGAACATCCAAAGCT

>Mus_musculus_Trim30f

ATGGCCTCATCAATCCTCGAGATGATAAAGGACGAGGTAACCTGTCCTATCTGTCTGGAGCTCCTGAAGGAACCTG
TGAGCACTGATTGTGACCACAGCTTCTGTCGAGCCTGCATCACACTAAACTATGAG---
TCCAGCAAAAGCAGAGGCACAGAAGAGGGCAGCTGCCCTGTGTGCCGAGTTCGTGTCCCATTTGGGAATCTGAG
AACTAATCGACAAATGGCCAATATAGTTGAGAGGCTCAAGGGGTTCAAGTCCATTCCAG---
AGGGGGAGCAGAAAGTGAATGTCTGTGCACAACATGGAGAGAAACTCCAGCTCTTCTGTAAGAAGGACATGATG
GCCATCTGCTGGGTTTGTGAGAGATCTCAGGAGCACCATGGTCACCAAACAGCTCTCATTGAAGAGGTGGACCAT
GAGTACAAGGAGAAGCTACAGACAGCCCTGCAGAAGCTGATGGAAAATGAGAAAAGATGTGATGAATGGCAGG
ATGACCTTCAGCAACAGAGAGCTGACTGGGAGAACCAAATACAGAGCAGTGTAGAAAATGTTCAGAGACAGTTTA
ACGGATTAAGAGAGTTCCTGGACTCCAAGGAGAATGGGGAGCTGCATAGGCTCAAGGAAGAGAAGGAAGAGGT
TGTGAAAAGGCTGGGAGAGTCTGAAAATGAGCTGGTGCAGCAGAGGCAGTGGGTGAGAGACCTCA---
TCTCAGATGTGGAACATCAGTTGGAGCTCTCAACCATGGAAATGCTGCA-GAATGTGAATAATGTCCTG-------
AGAA----------GGAGTCAGACCTTGAGACTGAAACAGCCCCAAATGATCCCG---AAAAAA---------ACATACCAACCT

>Mus_musculus_Trim34a

ATGGCCTCAACAGGTCTGACGAATATACAGGAGAAGACCACTTGCCCTGTCTGCCAAGAGCTTTTGACCAAAGCCC
TGAGTCTAGGCTGTGGCCACCGTGTATGCCAAGCCTGTCTCATCACGAAGAAGAAT---GCAGTGATCAACCCCAG--
-
AGAGAAAAGCAGCTGTCCTGTGTGTGGTACTAGATTCTCGTTGGAAAATCTACAGGCTAATAAACATCTGGCAAAT
GTAGTAGAGAGACTCGGGGAGGTTAAATTGAAGCCTG---
ACATTGGGACAAAGAGAGATCTCTGTGTACACCATGGAGAGAAGCTCCTTCTCTTCTGTAAGGAGGACAAGAAGG
CCATTTGCTGGGTTTGTGAGCGTTCTCAGGAGCATCGTGGTCACCACACATTCCTCTGGGAGGAAGCAGTCAGGG
AATGTCAGGAGAATCTCCAGAAAGCTCTCACGAGGCTGAGGAAGGAGCAGGAGAAGGTGGAGACATTGGAAGC
TGACATCAAAGAAGACAGACTTTCCTGGAAGTGCCAGGTCCAGACTGAGAGACAAAGGATTCAAACAGGCTTTAA
TCAGCTTAGAAGAATCCTGGACAAGGAGGAACAGAGAGAGCTGAAAAGACTCAGAGAAGAGGAGCAGATGATA

CTGGACAGCCTGGCGGGGGCAGAGGCTGAGCTGGCTCAACAGAGTCAGTTGGTGGAGGAACTTA---
TCTCGGATCTAGAGCTGCGGCGTGAGTGGTCAGATACAGAGTTGCTGCA-GGATATGAGCGGTATTTTG-------
AAAT----------GGAGTCAGATCTGGACACTGAAGAAGCCAAAAGCAGTTTCT---
AAGAAACTGAGCATGGTATTCCAAGCT

>Mus_musculus_Trim34b

ATGGCCTCAACAGGCCCGACGAATATACAGGAGAAGACCACTTGCCCTGTCTGCCAAGAGCTTTTGACCAAAGCC
CTGAGTCTAGGCTGTGGCCACCTTGTATGCCAAGCCTGTCTCATCTCGAACAAGAAT---GCAGTGATCAACCCCAG-
--
AGGGAAAAGCAGCTGTCCTGTGTGTGGTACTAGATTCTCGTTGGAAAATCTACAGGCTAATAAACATCTGGCAAA
TGTAGTAGAGAGACTCGGGGAGGTTAAATTGAAGCCTG---
ACATTGGGACAAAGAGAGATCTCTGTGTACACCATGGAGAGAAGCTCCTTCTCTTCTGTAAGGAGGACAAGAAGG
TCATTTGCTGGGTTTGTGAGCGTTCTCAGGAGCATCGTGGTCACCACACATTCCTCTGGGAGGAAGCAGTCAGGG
AATGTCAGGAGAATCTCCAGAAAGCTCTCACGAGGCTGAGGAAGGAGCAGGAGAAGGTGGAGACATTGGAAGC
TGACATCAAAGAAGACAGACTTTCCTGGAAGCGCCAGGTCCAGACTGAGAGACAAAGGATTCAAACAGGCTTTAA
TCAGCTTAGAAGAATCCTGGACAAGGAGGAACAGAGAGAGCTGAAAAGACTCAGAGAAGAGGAGCAGATGATA
CTGGACAGCCTGGCGGGGGCAGAGGCTGAGCTGGCTCAACAGAGTCAGTTGGTGGAGGAACTTA---
TCTCGGATCTAGAGCTGCGGCGTGAGTGGTCAGATACAGAGCTGCTGCA-GGATATGAGCGGTATTTTG-------
AAAT----------GGAGTCAGATCTGGACACTGAAGAAGCCAAAAGCAGTTTCT---
AAGAAACTGAGCATGGTATTCCAAGCT

>Octodon_degus_Trim5a

ATGGCTTCATCAGTCTTGGAGAATGTCAAGGAGGAGGTGACCTGCCCCATCTGCCTGGAGTTCATGCAGGATCCT
GTAAGCGCAGACTGTGGCCATGTTTTTGTAAATTCTGCATCACCAGAAACTATGAG---TCCATGCAACATGAAGA-
--
AGGAGTGGGCCACTGCCCCATGTGCCGAACCACGTACCAGTTTGAAAACCTGAGGCCCAATCGCCATATGGCCAA
CATAGTGGAGAGCCTCGAGGAGATGACTCTGACCCTGA---------------
CAGCAGATCTCTGTGAGCTCCATGGGGAGAAACTCGTGCTCTTTTGTAAGGAGGATGAGAATGTCATTTGCTGGCT
TTGTGAGCGTTCTCAGAAGCACCGTGGTCACCACACTGTTCTCCTGGAGGAGGCCGAGCACGACTATCGGGAGAT
GCTTGGTAAAGTGCTAGAAAAGCTGAAGAAGGATGAGAAAGAGATGGAGAAGTGGGAAGCTCATATTGAAGAA
GAAAGAACTTCCTGGAAGAATCAAATACAGGAGGAGAGAGAAGAAGTTGAGGCTGAGTTTAAGAAAATGAGAA
ATATCCTGGACTCTGAGGAGAAAGAGTACTTGCATAAACTGAAGAAAGAGGAAAAAGATGTTCTGAGGGTCCTG
GAAGATTCTGAAAAGCAGCTGGCCCGGGAGGTCCAATACTTGAGGGAGCTCA---
TCTCAGATGTGCAGCATCAGCTGCAGGGGTCAGCGAGGGCCATGTTGCA-GGGCATGAAGGACACCATG-------
GAAA----------GGAGTAAGTCAGTCATTGTGCAGGAACCAAGAACTTGCCCC---
AAGCGACAGAAGATGGTGTTCCAAGCT

>Octodon_degus_Trim5b

ATGGCTTCCTCAGTCCTAGAGAATGTCAAGGAGGAGGTGACCTGCCCCATCTGCCTGGAGCTCATGAAGGATCCT
GTGAGCGCAGACTGCGGCCACTATTTCTGCAAACTCTGCATCACCTCAAACTATGAG---TCCACAAAACATGAAGA-
--
AGGAGTGGGCCACTGCCCCGTGTGCCGAATAACGTACCAGTTTGAAAACCTGAGGTCCAGTCGACATATGGCCAA
CATAGTGGAGAGACTCAGGACATTCCCACTGCCCCCAA---------------
AGGCAGATCTGTGTGAGCTCCATGGGGAGAAACTCCTGCTGTTTTGTAAAGAGGATGAGGAGGTCATTTGCTGGC

TTTGTGAGCGTTCTCAGAAGCACCGTGGTCACTGCACTCTTTTAATGGAGGAGGCTGAACGGGAATATCGGGAGA
TGCTCGGCAAAGTGCTACAAAAGCTGAAGATGGACGAGAAAGAGTTGGAGAAGTGGGAAGCTCATATTGAAGAA
GAAAAAACTTCCTGGAAGAATCAAATACAGGGGGAGATAGAAACTGTCCGGGATGAGTTTAAGAAAATGAGAAA
TGTGCTGGACTCTGAGGAGAAAAAACACCTGCAGAAACTAGAGAAAGAAGAAGAAGATGCTCTGAGGATCCTGG
CAGAGTGTGAAAAGAAGCTGGCCCAGGATTCTGGGTCGGTGAGGGCGCTCA---
TCTTAGATGTGCAACTTCGGCTGCAGGGGTCAGCCATGGCCTTATTGCA-GGGTGTGAAGGACACCATG-------
GAAA----------GGAGTAAGTTATTCACTGTGCAGAAACCAAGAACTTGCCCC---
AAGCGACAGAAGATGGTGTTCCAAGCT

>Octodon_degus_Trim5c

ATGGCTTCCTCAGTCCTAGAGAATGTCAAGGAGGAGGTGACCTGCCCCATCTGCCTGGAGCTCATGAAGGATCCT
GTAAGCACAGACTGCGGCCACTATTTCTGCAAACTCTGCATCACCTCAAACTATGAG---TCCATGCAACACGGAGA-
--
AGGTGTGGGCCGCTGCCCCGTGTGCCGAATCACCTACCAGTTTGAAAACCTGAGGCCCAGTCGACATGTGGCCAA
CATAGTAGAGAGGCTCCGGGAGTTGACACTGCCCTTGA--------------
AGGCAGATCTGTGTGAGCTCCATGGGGAGAAACTCCTGCTGTTTTGTAAAGAGGATGAGGAGGTCATTTGCTGGC
TTTGTGAGCATTCTCAGAAGCACTGTGGTCACCACACTCTTCTCATGGAGGAGGCTGCACAGGAGTATCGGGAGA
TGCTTGGCAAAGTGCTAGAAAAACTGAAGAAGGATGAGAAAGAGTTGGAGAAGTGGGAAGCTCATATTGAAGAA
GAAAGTACTTCCTGGAAGAATCAAATAGAGGGGGAGATAAGAGATGTCCGGGGTGAGTTTAAGAAAATGAGAAA
CATCCTAGACTCCGAGGAGAAAGAGCATCTGCAAAAACTGAAGAAAGAGGAAGAAGATGGTCTGAGGATCCTGG
CAGAGTCTAGAAAGCAGCTGGCCCAGGATGTCCAGTACTTGAGGGGGCTCA---
TCTCAGATGTGCAGTATCGGGTGCAGGGGTCAGCGAGGGCCATGTTGCA-GG---
---TAAGCC

>Octodon_degus_Trim5d

ATG---

AGAAACATCCTGGACTCTGAGGAGGAAAAGCACCTGAGAAAACTGAAGAAAGAGGAGGAAGATGTTCTGAGGG
ACCTGGCTGAGTCTGAAAAGGAGCTGGCCCAGGAGGCCCAGTCTGTGAGGGCGCTCA---
TCTCAGATGTGCAGCATCGGCTGCAGGGGTCAGCAAGGACCATGTTGCA-GGGTGTGAAGGACACCATA-------
GAAA----------GGTGTAAGTTATTAATTGTGAAGAAACCAAGAAGTAGCCCC---
AAGAAACAGAGGGTGGTGTTCCAAGCT

>Octodon_degus_Trim6

ATGACTTCAAGAGTTCTGGTGGACATCCGAGAGGAGGTCACCTGTCCCATCTGCCTGGAGATCCTGACAGAACCTT
TGAGCACAGACTGTGGCCACAGCTTCTGCCAGGCCTGCATCACAGAGAACAGTGAGAAATCAGGAGTCGGCCAA
GA---
AGAGGAGAACAGCTGTCCTGTGTGCCACTCTGGCTACCACCCCCGGGACCTCCGGCCTAATCGGCACCTGGCCAA
CATAGCAGAGCGGCTCAGAGAGGTGGTGCTGGCACCTA---
TAACCCAGCCACAGGTCGCTCTCTGTGCACACCATGGAGAGAAGCTCCAGCTCTTCTGTAAGGAGGATGGGAAGC
TGATTTGCTGGCTTTGTGAGCGCTCCCAGGAACACCGTGGTCACCATACCTTCCTCATGGAGGAGGTAGCTCAGGA

GTACCAGGAAATGTTCCTAGAATCACTGCAGAAGTTGAGGAAAGACGAGGAGGAAGCTGAGAGACTAACAGCTG
TTATCAGAGAGAAGAGGACATCCTGGAAGAATCAGGTGGAGCCTGAGAGACACAGGATCCAGAAAGAGTTTGAT
CGTCTACGCAGTGTCCTGGACAGAGAGGAGCAGCGGGAGCTGAGGAAGCTGGAGGAGGAAGAGCGGAAAGGG
CTGAGCATCATAGAGAAAGCCGAGGGTGAGATGATCCGCCAGAGCCAGTCCCTCAGAGAGCTCA---
TCTCCGACCTCGAGTACCGGTGCCAGGGGTCCACCGTGGACCTGCTGCA-GGATGTGAATGAGCTCACA-------
CAAA----------GGAGTGAATTCTGGACCCTGAAGAAGTTCCAAGCACTCCCC---
ACCAAGCTGAGAAGTGCCTTTCGAGCC

>Octodon_degus_Trim34

ATGGCTTCTAAAATCCTGACTGACCTAGAGAAGGAGGCAACCTGCCCCATCTGCCGGAAACAGCTGACAGAGCCG
CTGGGTCTAGCTTGTGGTCATGCCTTGTGCCCATCCTGCATCACTGTGGACAGAGAGGAGGCAGAGGAGGGCTCA
GG---
AAAGGAGTGCAGCTGTCCTGTGTGTGGCACCGGGTCCCCCCATGAAAATCTATGGGCCAATCAGCACCAGGCTGA
CACAGTGGAGAAACTCGGGGAGTTGAAGCTGAACACAG---
GTGTTGAGAAGAAGATAGCTTTCTGTGCCCTCCATGGGGAGAAACTTCAACTCTTCTGTAAGGAGGACCAAAAGG
TCATTTGCTGGCTCTGCGAGCGTTCTCAGGAACACTGTGGCCACCACACGTTTCTCGTGGAGGAGGTAGTCCAGGA
GTGCCAGGAGAGAGTGCAGGCAGCTCTGCAGAGGCTGCGGGAAGAGCAGCAGGAAGCTGAGAAGTTGAAAGCT
GCCATCATGGAAGACAGAATTGCTTGGAAGCATGAGATGAAGACAGAGAGACAATGGATAGAAAGTAAATTTTG
TTGGCTTAGAAGAGCCCTGCACAATGAGAAGCAGAGAGAGCTGCGCATCCTGGAGGAGAAAGAAAGGAAGACG
CTGGCTGACTGGGAGCAAGCCGAGGATGAGATGCTTCAGCAGAGCCAGGTTCTGGAAGAGCTCA---
TCTCAGAGCTGGAGCATCGGAGCCAGTGGGCAGCCGAGGAGCTGCTGCA-GGACACAAGTGGAATTTTG-------
AAAT----------GGAGTGAGATCTGGACAGTGAGGAAGCCAAAAACTCCTCCC---
AGGAAAACAGACACTCGACTGGAGTTT

>Octodon_degus_TrimCyp

ATGGCTTCAGAGGTCCTGGAGAATGTCAAGGAGGCGGTGACCTGCCCCATCTGCTTGGAGCTCATGAGAGAAGCC
GTGAGCATCAACTGTGGCCACAGTTTCTGCAAACACTGCATCACCTCAAACTATGAG---TCCACGGAACATGA------
AGGTGTGGGCCACTGCCCTGTGTGCCGAATAATGTACCAGTTTGAAAACCTGAGACCCAGTCGACACGTGGTCAA
CATAGTGGAGAGCCTTGGAAAGTTGCCACTGACCCCGA---------------
AGATAGATCTGTGCAGCCTCCATGGGGAGAAACTCCTGCTGTTTTGTAAGCAGGATGAGGATGTCATTTGCTGGCT
TTGTGAGCGTTCTCAGAAGCACCGTGGTCACTGCACGCTTTTAATGGAGGACGCTGAACGGAAGTATCGGAGAAC
ACTTCAAGAAATGCTAGAGAAGCTGACTCAGGATGAAAAAGAGTTTAAGAAGTGGGAAGCTCACATTGAAGAAG
AAAGAACTTCCTGGAAGAAACAAATACAGGAGGAGATGCAAAATGTCCAGGATGAATTTACAAAAATGAGAAAC
ATCCTGGACTCTGAGGAGAAAGAGCACCTGCAAAAACTGAAGCAAGAGGAAGAAAATGCTCTCAAGGTCCTGGC
CGAGTCTGAAAAGCAGCTGGCCCGGGACGCCCAGTCCGTGAGGGAGCTCA---
TCTCAGATGTGCAGCGTCGGCTGCAGGCATCAGCCATGGCCATGTTGCA-GGGTGTGAAGGACACCATA-------
GAAA----------GGAGTAAGTCATTGACTGTGCAGAAACCAAGAATTTGCCCT---
AAGAGACAGAAGATGGTGTTTCAAGCT

>Peromyscus_maniculatus_Trim6

ATGACGTCAGCAGTTCTGGTGGACATCCGCGATGAGGTCACCTGCCCTATCTGCTTGGAGCTCTTGACAGAACCCC
TGAGCATAGACTGCGGTCACAGCTTCTGCCAGGCCTGCATCACAGGAAACAGTGACAAGTTAGTGTTCAACCCAG
A---
AGGGAAGAGCAGCTGTCCTGTGTGCCGGACCGCCTACCAGCCCGGGAACCTCCGGCCTAATCGACACCTGGCTAT

CATAGTGAAGAGGCTCAGAGAGGTCGTTTTGGCCCCTG---
GAAAGCAGCTGGAGGTCATTCTTTGTGCAGTTCATGGAGAGAAACTCCAGCTTTTCTGCAAGGAGGATGGGAAGT
TAATTTGCTGGCTGTGTGAGCGATCTCAGGAGCACCATGGTCATCACACGTTCCTCATGGAGGAGGTGGCCCAGG
AGTACCAGGAGATGTTCCAGGAGTCTCTGAAGAAGCTGAGGAAGGAGCAGCAGGAAGCTGAGAGGCTAAAAGC
TCTTATCCAAGAGAAGAGGGAATCCTGGAAGAATCAGGTAGAGCCTGAGAGACACCGGATCCAGACTGAATTTAA
GCAGCTGCGGAGCATCCTGGACAGGGAGGAGCAGAGGGAACTGAAGAAACTGGAAGCGGAAGAGAGGAAGGG
GCTGAGCATCATAGAGAAGGCTGAGGGGGACCTGATCCACCAGAGCCAGTCACTGAAAGATCTCA---
TCTCAGACCTGGAGCACCGGTGCCAGGGGTCTGCAGTGGAACTGCTGCA-GGATGTGAATGATGTCACA-------
AAAA----------GGAGTGAGTTCTGGACCCTGAGGAAGCCCCAAGCTCTTCCC---
ACCAAGCTGAAAAGTTTGTTTCGAGCC

>Peromyscus_maniculatus_Trim12

ATGGCTTCAGAATTCTTGATGAATGTAAAGGAGGAGGTGACCTGTCCTATCTGTCTGGACCTCATGGTGGAACCTG
TGAGTGCTGATTGTGGTCACAGCTTCTGCCGAGCCTGCATCACATTGAACTGTGAG---TCTATTAAAGGCAAAGA--
-
GGGAGAGTTCACCTGCCCTGTGTGCCGAGTGAGTTACCTGTTTGGGAATCTGAGGCCTAATCGACATTTGGCCAAC
ATAGTGGAGAGGCTCAAGGGGTTCAAGTCCAGCCCAG---
AGGGGGAGCAGAAGTTGAATGTCTGTGCAAGGCATGGAGAGAAACTGCAGCTCTTCTGTGAGAAGGACAAGGT
GGCCATCTGCTGGCTTTGTGAGCGATCTCAGGAGCACCGTGGTCACCAAACAGCTCTCATCGAAGAGGTGGCCCA
AGACTACAAGGGGAAGCTCCAGGCAGCTCTGCAAAAGCTGATGGCAGACAAGAAAGAATTGGAGAACTGGAAA
GATGACCTTCAAAAGGAGAGAACTTTCTGGGAGAATCAAATACAGAAAGATGTAGAAAATGTTCAGATGGAGTTT
AGAAGACTGGGAGACATCCTGGACTCTGAGGAGAAGAATGAGGTGCAGAAGCTGATGCAAGAGAAGAAAGACA
TTATGAACAGCCTGGCAGAGTCTGAAAATAAGCATGCCCAGCAAAGCAAGTTGCTAGGAGACCTCA---
TCTCAGATGTGGAGCATCACTTGCAGTGCTCAACCATGGAAATGCTGCA-GGGTGTGGATGGCATCATA-------
AAAT----------GGAGTCACAGTTTTTCACTCACGAAGTCCAAAACCATCCCC---
AAGGAAGGAAGAAGAGTGTTCAGAGCC

>Peromyscus_maniculatus_Trim30b

ATGACCTCATCAGTCCTGGGGATGATCAAGGAGGAGGTGACCTGTCCTATCTGCCTTGACCTGATGGTGGAACCT
GTGAATGTGGATTGTGGTCACAGCTTCTGCCGAGCCTGCATCAAACGGAACTATGAG---
TCCAGCAAAGGCAAAGA---
AGAGGAGGGCATCTGCCCTGTGTGCCAACTTTGTTACATGTTTGGGAATCTGAGGCCTAATCGACATGTGGCCAAC
ATAGTGGAGAGGCTAACAGGGTTCAAGTCCAGCCCAG---
AGGAGGAGCAGAAGGTGAATGTCTGTGCACAACATGGAGAGAAACTCCAGCTCTTTTGTGAGAACGACATGGTG
GCCATCTGCTGGCTTTGTGAGCGATCTCAGGAACACCGTGGTCACCAAACAGATCTCATTGAAGAGGTGGCCCAT
GAGTACAAGGAGAAGCTCCAGACAGCTCTGCAGGAACAGATGGCAAAAGAGAAAAGATGTGATGAGTGGGAAA
ATGATCTTCAAGAGGAGAGAACTTTCTGGAAGAACCAAATACAGGGTGATGTAGAAAAAGTTCAGATGGAGTTTA
AAGGACTTCGGGAATTCCTGGACTCCAAGGAGAAGAATGAGGTGCAGAAGCTGAAGCAGGAGGAGGAAGACAT
TATGAACAGCTTGGCAGAGTCTGAAAGTGAGCTGGTGAAGCAGAGGGAGTCAGTGAGAGCCCTCA---
TCTCAGATCTGGAGCATCAGTTGCAGTGCTCAACCATGGAAATGCTGCA-GGGTGTGAATTCTGTCCTT-------
AAAA----------GGAGTCAGACCTTAAGACTGAAACAACCTGAAATGATCCCA---
AGAAAACAGAGAAGGATCTTCAGAGCT

>Peromyscus_maniculatus_Trim30c

ATGGACTCATCAGTCATGTTCATGATGGAGGAGGAGGTGACCTGTCCTATCTGCCTGGAGCTCCTGAATGAACCTG
TGAGTGCTGATTGTGACCACAGCTTCTGCCGAGCCTGCATCACACTGAACTATGAG---TCCAGCAAAAGCAAAGA---
AGGGGAGGGTGTCTGCCCTGTGTGCCGAGTGAGTTACCTGTTTGGGAATCTGAGGCCTAATCGACATGTGGCCAA
CATAGTGGAGAGGCTCAAGGGGCTCAAGTCCAACCTAA---
AGAAGCAGCAGAAGGTGAATGTCTGTGCACAACATGGAGAGAAACTCCAGCTCTTCTGTGAGAAGGACATGGTG
GCCATCTGCTGGCTTTGTGAGCGGTCTCAGGAGCACCGTGGCCACGTAACAGCTCTCATTGAAGAAGTGGCCTAT
AAGTATGAGGAGAAGCTCCAGGCAGCTCTGCAGATGCAGATGGCAAATGAGAAAAGATGTGATGAGTGGGAAG
ATGGCCTTCAAAAGGAGAGAACTTTCTGGAAGAACCAAATACAGGGTGATGTAGAAAAAGTTCAGATGGAGTTTA
AAGGACTTCGGGAATTCCTGGACTCCAAGGAGAAGAATGAGGTGCAGAAGCTGAAGCAGGAGGAGGAAGACAT
TATGAACAGCTTGGCAGAGTCTGAAAGTGAGCTGGTGAAGCAGAGGGAGTCAGTGAGAGCCCTCA---
TCTCAGATCTGGAGCATCAGTTGCAGTGCTCAACCATGGAAATGCTGCA-GGGTGTGAATTCTGTCCTT-------
AAAA----------GGAGTCAGACCTTAAGACTGAAACAACCTGAAATGATCCCA---
AGAAAACAGAGAAGGATCTTCAGAGCT

>Peromyscus_maniculatus_Trim30d

ATGGCCTCATCAGTCCTGGTGATGATCCAAGAGGAGGTGACCTGTCCCATCTGCCTGGAGCTCCTGAAGGAACCT
GTGAGTGCTGATTGTGACCACAGCTTCTGCCGAGCCTGCATCACACTGAACTATGAG---
TCCAGCAAAGTCAATGA---
AGGGGAGGGCATCTGCCCTGTGTGCCGAGTGAGTTACCAGTTTGGGAATCTGAGGCCTAATCGACAATTGGCCAA
CATAGTGGGGAAGCTAACAGGGTTCAAGTCCAGCCCAG---
AGGAGGAGCAGAAGGTGAATGTCTGTGCACAACATGGAGAGAAACTGCAGCTCTTCTGTAAGAAGGACAAGGTG
GCCATCTGCTGGCTTTGTGAGCGGTCTCAGGAGCACCGTGGTCACCAAACAGCTCTTATTGAAGAGGTGGCCCAA
GAGTACAGGGGGAAGCTCCAGGCAACTCTACAGAAACTGGTGGCAAAAGAAAAAATATGTGATGAGTGGAAAG
ATGACCTTCAAGAGGAGAGAACTTTCTGGAAGAACCAAATACAGGGTGATGTAGAAAAAGTTCAGATGGAGTTTA
AAGAACTTCGGGAATTCCTGGACTCCAAGGAGAAGAATGAGGTGCAGAAGCTGAAGCAGGAGGAGGAAGACAT
TATGAACAGCCTGGCAGAGTCTGAAAGTGAGCTGGTGAAGCAGAGGGAGTCAGTGAGAGACCTCA---
TCTCAGATGTGGAGCATCAGTTGCAGTGCTCAACCATGGAAATGCTGCA-GGGTATGAATTCTGTTCTA-------
ACAA----------GGAGTCAGACCTTAAGACTGAAACAACCTGAAATGATCCCA---
AGAAAACAGAGAAGGATCTTCCGCGCT

>Peromyscus_maniculatus_Trim34

ATGGCTTCAGCAGTTCCAATGCGTGTACAGAAGGAGGCCATTTGCCCCGTCTGCCAAGAGCTTTTGAAAGAACCCC
TGAGGTTAGGCTGTGGCCACAATGTATGCCAAGCCTGCATCACCATGAACAGAAAGAATGCAGTGATCAACCCCA
G---
AGGAAAAAGCAGCTGTCCTGTGTGTGGTACTAGATTCTCATTTGAAAATCTACAGTCCAGTCAGCATCTGGCAGAC
ATAGTAGAGAGACTCAGGGAAGTCGAGTTGAACCCTG---
ACCTTGAGAAAAAGAGAGATCTCTGTATACACCATGGAGAGAAACTACGTCTCTTCTGTAAGGAAGACAGGAAGG
TCATTTGCTGGGTTTGTGAGCGTTCTCGGGAGCATCATGGTCACCACACCTTCCTCCAGGAGGAAGCAGTCAAGGA
GTGTCAGGAGAATCTCCAAACAGCTCTCCAGAGGCTGAGGGAGGAGCACAAGAAGGCGGAGAAATTGGAAGCT
GACATCAAAGAAGACAGAATTTCTTGGAAGTGCCAGATCCAGACTGAGAGACAAAGGATACAAACGGGTTTTAAT
GAGCTCAGAAGAATCCTGAATGAAGAGGAACAGAGAGAGCTAAAAAGACTTCAGGAAGAGGAGCAGCTGATAC
TTGCCAGCCTGGCAGAGGCCGAGGCTGAGCTAGCTCAGCAGAGCCAGTTGGTGGAGGAACTTA---
TCTCAGATCTGGAGCGCCGGTGCCAGTGGTCAACTACAGAGCTGCTACA-GGATATGAGCGGTGTCTTG-------

AAAT----------GGAGTCAGATCTGGACACTGAAGAAGCCAAAAGCAGTTTCT---
AAGAAACTGAAGAAGATATTCCAAGCT

>Rattus_norvegicus_Trim5

ATGGCTTCAGAATTCGTGATGAATTTAAAAGAGGAGGTGACCTGTCCTATCTGCCTGGACCTGATGGTAGAACCTG
TGAGTGGAGATTGTGGTCACAGCTTCTGCCAAGCCTGCATCACGCTGAACTATGAA---TCCAGCAAATGCAATCA--
-
GGATGAGTTCATTTGCCCTGTGTGCCGAGTTAGTTACCTGTTTAAGAACCTGAGGCCCAATCGACATGTGGCCAAC
ATAGTGCAGAGGCTCAAAGAGTTCAAGTCCAGCCCAG---
AAGAGGAGCCGAAGGTGCTTTCTTGTGCAAGGCATGGAGAGAAACTCCAGCTCTTCTGTAAGAAGGACATGATGC
CCATCTGCTGGCTTTGTGAGCGATCTCAGGAGCACCGTGGACACCAAACAGTTCTCATTGAAGAGGTGGTCCAGG
AGTATAAGGAGAAGCTGCAGGCAGCTCTGCAAAAGCTGATGGCAGACAAGAAAGAATTTGAGAACTGGAAAGAT
GACCTTCAAAAGGAGAGAACTTACTGGCAGAATCAAATACAGAAAGATGTGGAAAATGTCCAGTCAGAGTTTAAA
GGAATGAGAGATATCATGGACTCTGAGGAGAAGAAGGAATTGCAGAAGCTGATGCAAGAGAAGGAAGACATTA
TGAGCAGCCTGGAAGAGTCTGAAAATGAGTATTCTCAGCAGAGTAAGTTGCTAGGAGACCTCA---
TCTTAGATGTGGAACATCAGTTACAATGCTCAGCCACGGAAATGCTGCA-GGGTGTAGAGAACACCATA-------
AAAC----------GGAGTCATACTTTTTCGATGAGGAAGCCCAAAACCATCCCC---
AAGGAACAAAGAAGAGTGTTCCGAGCC

>Rattus_norvegicus_Trim6

ATGACTTCAGCAGTCCTGGTGGACATCCGAGATGAAGTCACTTGCCCTATCTGCTTGGAGCTCCTGACAGAACCCC
TGAGCATAGATTGCGGTCATAGCTTCTGCCAGGCCTGCATCATAGGAAACAGTGATAATTCAGTGCTCAACCCAGA

AGGGAAGAGCAGCTGTCCTGTGTGCCGGACCGTCTACCAGCCTGGGAGCCTCCGTCCTAACCGGCACCTGGCCGC
CATAGTGAAAAGGCTCAGAGAAGTCGTGTTAGGCCCTG---
GAAAACAGCTCGAGGTCATTTTTTGTGCGCTTCATGGAGAGAAACTTCAACTTTTTTGCAAGGAGGATGGGAAGTT
AATTTGCTGGCTTTGTGAGCGATCTCTGGAGCACCGTGGTCATCACACATTCCTCATGGAGGAGGTAGCACAGGA
GTACCAGGACATGTTCCAGGAGTCTCTGAAGAAGCTGAGGAGGGAGCAGCAGGAAGCCGAGAAGCTAAAAGCTC
TTATCCAAGAGAAGAGGGAATCCTGGAAGAGTCAGGTGGAGCCTGAGAGACACCGGATCCAGACAGAGTTTAAT
CAGCTCCGAAGCATCCTGGACAGGGAGGAGCAGCGGGAACTGAAGAAACTGGAAGTGGAAGAGAGGAAGGGG
CTGAGCGTCATAGAAAAGGCCGAGGGTGACCTGATCCACCAGAGCCAGGCACTAAAAGACCTCA---
TCTCAGACCTGGAGCACCGGTGCCGGGGGTCCACAGTGGAACTGTTGCA-GGATGTGGGTGACGTCACA-------
AAAA----------GGAGTGAGTTCTGGACCTTGAAGAAGCCCCAAGCTCTCCCC---
ACCAAGCTGAAAAGTTTGTTTCGAGCC

>Rattus_norvegicus_Trim30

ATGGCCTCATCAGTCCTGGAAATGATAAAGGAGGAGGTGACCTGTCCTATCTGTCTGGAGCTCCTGAAGGAACCC
GTGAGTACTGATTGCAACCATAGCTTCTGCAGAGCCTGCATCACAATAAACTATGAG---
TCCAACAGGAACACAGA---
AGGGGAGGGCAGCTGCCCCGTGTGCCGAGTGTGTTACCTGTTTAGAAACTTAAGGCCTAATCGACATGTGGCCAA
CATAGTGGAGAGACTCAAGGGGTTCAAGTCCATCCCAG---
AGGAAGAACAGAAGGTGAATGTCTGTGCACAACATGGAGAGAAACTCCAGCTCTTCTGTAAGAAGGACAAGATG
GCCATCTGCTGGCTTTGTGAGCGATCTCAAGAGCACCATGGTCACAAGACAGCTCTGGTTGAAGAGGTGGACCAT
GAGTACAAGGAGAAGCTGCAGGATGCTCTGCAAAAGCTGATGGAAAAAGAGAAAACATGTGATGAATGGCAGG

ATGTCATTCAACAGCAGAGAACTTACTGGGAGAACCAAATACAGAGTGATATAAAAAATGTTCAAATAGAGATTA
ACGGGCTAAGAGAGCTCCTGGACTCCAAGGAGAATGAAGAGCTGCAGGAGCTGAAGAAAGAGAAAGAAGATGT
TTTTCAAAGGCTGGAAGAGTCTGAAAATGAGCTGGTGCAGCAGAGGGAGTCCGTGAGAGACTGCA---
TCTCAGATGTAGAACATCAGTTGGAGCTCTCAACTATGGAAATGCTCCA-GAGTGTGGAATATGTCCTG-------
AGAA----------GGAGTCAGACCTTGAAACTGAAACTGCCGGATATTATCTCA---GAAAGAAGAAGAAA---
GTTCCAAGCT

>Rattus_norvegicus_Trim30c

ATGGCCTCATCTGTCCTGGAGATGATAAAGGAGGAGGTGACCTGTCCTCTCTGTCTAGAGCTCCTGAAGGAACCC
GTGAGTGCTGATTGCAACCATAGCTTCTGCAGAGCCTGCATCACTCCGAACTATGAG---
TCCAACAGAAACACAGA---
AGGGCAGGGCAGCTGCCCTGTGTGCCGAGTTCGTTACCTGTTTAGGAACCTGAGGCCTAATCAACATGTGGCCAA
GATAGTTGAGAGTCTCAAGGGGTTCAAGTCCATTCCAG---
AGAAGGAGCAAAAGGTGAATGTTTGTGCACAACATGGAGAGAAACTCCAGCTCTTCTGTACGAAGGAGATGAGA
GCCATCTGTTGGGTTTGTGAGAGATCTCAGGACCACCGTGGTCACCAGACAGCTCTCATTGAAGAGCTGGACCAT
GAGTACAAGAGGAAGCTGCAGGATGCTCTGCAAAAGCTGATGGAAAAAGAGAAAACATGTGATGAATGGCAAG
AGAACGTTCAACAGCAGAGAACTTACTGGGAGACTCAAATACAGAGTGATGTAGAATATATTCAGAAGGAGTTTA
AATTACTAAGAGAGCTTCTGGACTCCAAGGAGAATGAAAAGCTGCAGGAGTTGAAGAAAGAGAAGGAAGATGTT
GTGAAAAGGTTGGAAAGGTCTGAAAATGAGCTGGTGCAGAGGAGGCAGCGGGTGAGAGACCTCA---
TCTCATATATGCAGCATCAGTTGAAGTTCTCAACCATGGACATGCAGCA-GGATGTGAATTCTCTTCTA-------
AGAAGTTATGGTGCGGAGTCAGACCTTGAAACTGAAACAGCCGCAAACTATCCCA---
AAAAGAAGAAGAAGAATGTTCCAAGCT

>Rattus_norvegicus_Trim34

ATGGCCTCAACAGATCAGACAGATGTACAGAAGGAGGCCACTTGCCCTGTCTGCCACGTGCTTTTGATAAAACCCT
TGAATCTAGGCTCTGGCCACCATGTATCCCAAGCCTGCCTCACCGTGAA---GAAGACTGCAGTGACCAACCCCAG--
-
AGGGAAAAGCCTCTGTCCTATGTGTGGCAATAGATTCTCATTTGAAAATCTACAGGCTAATAAGCATCTGGCAGAT
GTAGTAGAGAGACTCAGGGAAGTCAAGTTGAGCCCTG---
ACATTGGGAAAAAGGGAGATCTCTGCGTACACCATGGAGAGAAACTCCTTCTCTTCTGTCAAGAGGACAAGAAGG
TCATTTGCTGGGTTTGTGAGCGTTCTCAGGAGCATCGTGGTCACCACACCTTCCTCCGGGACGAAGCAGTCAGGGA
ATGTCAGGAGAATCTCCAGGAAGCTCTCAAGAGGCTGAGGAAGGAGCAGGAGAAGGTGGAGACATTTGAAGCT
GACATCAAAGAAGACAGAATTTCCTGGAAGCACCAGATCCAGACCGAGAGACAAAGGATCCAAACAGGCTATAA
TCAACTTAGAAGAATCCTGGACAAGGAGGAACAGAACGAGCTGAAAAGACTCAGGGAAGAGGAGCAGATGATA
CTAGACAGCCTGGCAGGGGCAGAGGCTGAGCTGGCTCAGCAGAGCCAGTTGGTGGAGGAACTCA---
TCTCGGATCTAGAGCGTCGGTGTGAGTGGTCAGACACAGAGCTGCTGCA-GGATGTAAGCGGTATCTTG-------
AAAT----------GGAGTCAGATCTGGACACTAAAGAAGCCAAAAGCAGTTTCT---
AAGAAACTGAAGATGGTATTCCAAGCT

Supplementary Data S2

>Castor_canadensis_Trim6

CCAGATCTGAAAAGGATGCTGCGAGTATTCAGAGAGCTGACAGATGTCCAAAGCTACTGGGTGGATGTGACCTTG
AACCCACACACAGCTAATTTAAATCTCATCCTGTCTAAAAACAGGAGACAAG----TGAGATTT--------GTG-GGTGC--
----TAAGCTGTCTGAA------CCTTCCCGTCTGGAAGAA-CATT-----------------------------ATAA------
CTGTAGTGTCCTCGGCTCTCAGCACTTCTCCTCAGGAAAGTACTACTGGGAGGTAGATGTGACCAAGAAGACTGCC
TGGATTCTGGGCGTGTGCAGCAAT---------TCAGGGGA------------ACCTACA------TTCTCTT--------------------------
TCAACCAGTATCCTC---------ACCAGCA------------------GAATGC--------
TTACTCCAGATTTCAGCCGCAGAGTGGATTCTGGGTGATTGGGTTGC---------
AGCACAAGCACGAATACAGAGCCTATGAGGA---CTCCTCCACTT------------
CCCTGCTTCTCTCCATGACGGTACCCCCTCGTCGTGTTGGGGTTTTCTTAGACTATGAGGCCGGCACTGTCTCATTTT
ACAATGTCACAAACCATGGCTTGCCCATCTACACCTTCTCTAAATATTACTTTCCTACAGCCCTTTGTCCATATTTTAA
TCCTTGCAGCTGTGCAGTCCCAATGACTCTACGTCGCCCAA------------------GTTCTTGA--------------------

>Castor_canadensis_Trim34

CCAGACCTGAGGGGGATGTGGCAAGTGTTTAAAGAGCTAACAAATGTCCGGTGCTACTGGGTGGACTTCACATTG
AATCCAGTCAACCTAAATTTGAATCTTGTCCTTTCAGAAGATCAGCGACAAG----TGAAATCT-------------GTGC------
CAATTTGGCCAGT--------------------TAAG-TGTT-----------------------------CTAA------
TTATTGTATCTTAGGCTCCCAATATTTCTCCTCAGGGAAACATTACTGGGAAATAGATGTATCCAAGAAAACTGCCT
GGATCCTTGGAGTATACAGTAGAAAACAT---TCTATAAA------------------G------AACAGTG--------------------------
GTAGACCAGGCACAG---------ATTTTCC------------------AGATGT--------
TTACTACAGATACAGACCTCAAAATGGCTACTGGGTTATAGGGTTAC---------
AAAACAAATTTAAGTATAACGCCTTTGAGGA---
CTCTCCCACCTCTTATCCAACACTTTTGACCCTCTTTATGGCTATTCCTCCCCATCGTGTTGGAGTTTTCCTCGACTAT
GATGGAGGCACTACCTCATTTTTCAATGTCACAAATCATGGGTCACTCATCTACAAGTTCTCTAAATGTTGCTTTTCT
CAGCCTGTTTATCCATATTTCAATCCTTGGGATTGTCCAGCCCCCATGACTCTGTGCCCACCAA------------------
GCTCCTGA--------------------

>Cavia_porcellus_Trim6

CCAGATCTGAAAAAGATGCTGCAAGTGTTTCGAGAGCTCACGGATGTCCAGAGCTACTGGGTGGATGTGACCATG
AATCCACACTCTGCTAATCTGAATCTCGTGCTGTCTAAAAACCGGAGACAGG----TGAGATTT--------GTG-GGTGC--
----TAAGCTGGCCGGA------CCATCCTGCCTGGAGGAA-CATT------------------------------ATGA------
CTGTAGCATACTGGGCTCTCAGCACTTCTCCTCAGGAAAATACTACTGGGAGGTGGATGTGACTAAGAAGACCGC
CTGGATCCTGGGCGTGTGCAGCCAT---------TCCGCAGG------------ACCTCCG------TTTTCTT--------------------------
GCAGCCGCTATGCCA---------ACAATCA------------------GAACAC--------
TTACTCCAGATACCAGCCACAGACCGGATACTGGGTGATCGGGTTAC---------
AGCACAAACGTGAATACCGAGCCTACGAGGA---CTCTTCCACCT------------
CCCTGCTCCTCTCCATGTCTGTGCCACCCCGTCGCATTGGGGTTTACTTGGACTATGAGGCTGGCACTGTCTCTTTTT
ATAATGTCACAAACCATGGCTTGCCCATCTACACTTTCTCTAAGTACTATTTCCCTACAGCTCTTTGTCCCTATTTCAA
TCCTTGTGACTGTGTGGTCCCGATGACCCTGAGGCGCCCAG------------------GTCCCTGA--------------------

>Cavia_porcellus_Trim34

TCAGACCTGACAACGATGCTGGAACTGAATAGAGAGCTCAAAGATATCCAAGGCTACTGGGTGGATTTTACACTG
AATCCAGTCAACTTAAATTTGAATCTTGTCCTGTCGGAAGATCACAGACAAG----TGAGACCT-------------GTGC-----

-CAATTTGGCCGGT--------------------TAGG-TGTG-----------------------------ATAA------
TTATGGTATATTAGGCTCCCAGTGTTTCTCCTCAGGAAAACATTATTGGGAAATAGATGTGTCTAGCAAGACTGCCT
GGGTCCTGGGCATATACTGTAGAAAA------CCTGTAAA------------------G------TTTGCTG--------------------------
TTAATCGCAGCACAA---------TCCTTTC------------------AAATCC--------
TTTCTCTAGGTACACACCTCAGCATGGCTACTGGGTTATAGGCTTAC---------
AAAAGAAACTTGAGTATTATGCCTTCGATGA---
ATCCTCTGCCTTTGACCCTAAGGTTCTGGCTCTCTCTGTGGCTCTTCCTCTCCATCGTGTTGGGGTTTTCCTCAACTTT
GAAGCAGGCACTGTCTCCTTTTTCAACATCACAAACCATGGGTCTCTCATCTACAGATTCTGTAAATGTTACTATTCT
CGGCCTGTTTATCCGTATTTCAATCCTTGGGACTGTCCAGCCCCCTTGACTTTGTGCCCTCCGA------------------
GCTCCTGA--------------------

>Chinchilla_lanigera_Trim6

CCAGATCTGAAAAAGATGCTGCAAGTGTTTCGAGAGCTCACCGACGTCCAGAGCTACTGGGTGGACGTGACCATG
AATCCACACTCTGCTAATTTAAATCTTGTGCTGTCTAAAAACCGGAGACAGG----TGAGATTC--------GTG-GGTGC--
----TAAGCTGACCGGA------CCGTCCCGTCTGGAAGAA-CATT-----------------------------ATGA------
CTGTAGCATCCTGGGCTCTCAGCACTTCTCCTCAGGAAAATACTACTGGGAGGTGGACGTGACGAAGAAGACTGC
CTGGATCCTGGGAGTGTGCAGCAAT---------TCCACAGG------------GCCTCCG------TTTGCTT--------------------------
GCAGCCGCTATCCTA---------ACAGTCA------------------GAACAC--------
TTACTCCAGGTACCAGCCACAGACCGGATACTGGGTGATCGGCTTAC---------
AGCACAAACGTGAATACAGAGCTTACGAGGA---CTCCTCCACCT------------
CCCTGCTGCTCTCCATGACCGTGCCCCCCCGCCGCATTGGGGTTTACTTGGACTATGAGGCTGGCACGGTGTCTTTT
TATAATGTCACGAACCATGGCTTGCCCATCTACACTTTCTCCAAGTACTATTTCCCCACAGCCCTTTGTCCATACTTC
AATCCTTGCGACTGCGTGGTCCCAATGACCCTGAGGCGCCCAG------------------GACCTTGA--------------------

>Chinchilla_lanigera_Trim34

CCAAGGCTGAGTACAGTGCTGCGAATGTTCAGAGAGCTGAAAGATATCCAAGACTACTGGGTGGATGTTACACTG
AATCCCGTCAACCTAAATTTGAATCTTGTGCTTTCGGAGGATCACAGACAAG----TGAGAGCT-------------GTGC-----
-CAATTTGGCCATT--------------------CAAG-TGTG-----------------------------ATAA------
TTATGGGATTTTAGGCTCCCAGTATTTCTCCTCGGGAAAACATTATTGGGAAATCGATGTATCCAACAAGACTGCCT
GGCTCCTGGGTGTATACTGTAGAAAAC-----CTTCTAGA----------------AAG------TTCGATA--------------------------
GTAGTTGCAGCACAA---------CCCGTAC------------------AAACAC--------
TTGCTCTAGGTACAGACCTCAACATGGCTACTGGGTTATAGGGTTAC---------
GGGAGAAATTTGAATATTACGCCTTCGATGA---
ATCTTCTTCTTCAGGTCCTAAGGTGGTGGCGCTTTCTGTGGATATTCCTCTCTGTCGCATTGGGGTTTTCCTCAACTT
TGAAGCAGGCACTGTCTCCTTTTACAACATCACGAACCATGGGTCACTCATCTACACATTCCGTAATTGTTTCTATCC
TCGGCCTGTTTATCCATATTTCAACCCCTGGAACTGTCCAGTCCCCTTGACTTTGTGCCCCCCAA------------------
GCTCCTGA--------------------

>Cricetulus_griseus_Trim6

CCGGATCTGAAAAAGATGCTTCGAGTCTTTCGAGAGTTGACAGACGTCCAAAACTACTGGGTGGACGTGACTCTG
AATCCACAGACGGCTAATTTAAATCTTGTCCTATCTAAAAACCGGAGACAGG----TGAGGTTT--------GTG-GGTGC-
-----CAAGCTGTCCAAG------CCTTCCTGTCTGGAGGAA-CATT-----------------------------ATGA------
CTGTAGTGTCCTGGGCTCTCAGCACTTCTCCTCAGGAAAATACTACTGGGAGGTGGATGTGACCAAGAAGACGGC
ATGGATCCTGGGTGTGTGCAGTAAC---------CAGGTGGA------------ACCCATG------TTCTCTT--------------------------

TCAGCCAGTATCCAG---------GCAAGCA------------------GAGTGC--------
CTACTCCAGGTATCAGCCCCAGAGCGGATACTGGGTGATTGGGCTGC---------
AGCATAAGCATGAATACAGAGCCTATGAGGA---CTCCTCCACCT------------
CCTTGCTCCTCTCTATGACAGTGCCACCTCGCCGCATAGGGGTTTTCTTAGACTACGAGGCTGGCACTGTATCTTTT
TATAACGTCACAAACCATGGCTTGCCCATCTACACCTTCTCTAAATATTACTTTCCTACCGCTCTTTGTCCATATTTTA
ATCCCTGCAGCTGTGTAGTCCCAATGACTCTGCGTCGACCAA------------------GCCCTTGA--------------------

>Cricetulus_griseus_Trim12

CCTGATCTGCAAGGCATGCTGCAAGTGCTGCAAGAGCTCATAGAAGCCCAACGTTACTGGGTTCAAGTGACATTG
ATTGAAAACAAC---AATCCAAACATTGTCATTACTGCGGACAAGAGACAAA----TAAGATAT--------GAA-GACCA---
---TCAAACAAGAAAT------GTTGCATCTGGGGGTCAG-AACT------------------------------GTCG------
TGAAGGTGTCCTGGGCTACCCTGCTATCCAATCAGGAAAACATTATTGGGAAATGGATGTGTCTGGAAAAGGTGC
CTGGGTTCTGGGATTAAGTGATGGAAGCTAC---CTCTTCAA------------TCCGATA------
TTTCATTCAAATGCTGAAA--------------GATGCCTAAATCCTTTAT------TTCGTTT------------------GGGTATCAGCAA-
-TGACTCACATTATCAACCTAAATATGGCTTCTGGGTCATAGGGTTGT---------
GGAACAAGTTTGTGTATAATGCTTTTGAGGAGTGTACTTTCACAGGCAAGCCCAGGGTCTTGACCCTCTCGCTGAT
GGTTCCTCCCTGTCGCGTTGGCGTTTTCCTTGACTATGCAGCTGGCACGCTCTCGTTTTACAATATTTCCCACCATGG
GACTCTCATCTATAGATTCTGTGCAAATTCCTTTCCTGATAGGGTTTTTCCATATTTTAATCCTATGGGATGTTCAGA
GCCAATGACAGTATGCTGGCCAG------------------ACTCTTAA--------------------

>Cricetulus_griseus_Trim30a

TCCGACCTGAAAGACATGCTGAAAGTGTTTCATGGGATCATGTATGCCCAGCGCTACTGGGTTCACGTGACCCTGC
CTCAAACCTAC---AATAAAAATGTTGTCATTAACATGGGCAAAAGACAAA----TACAATAT--------CGA-AAGGA-----
-TTATAGAAGACAT------TTGCAAGAT---TATGAG-ACCT-----------------------------ATGA------
TTTAGGGGTCCTTGGATATCCAGCTATCTATTCAGGGAAACATTACTGGGAAGTAGATGTGTCTGGAAGTGATGCC
TGGCTCCTGGGATTAAATGATGGGAGAT-----GTGCTCAA------------CCCTA-C------
CTTCATGCAGTGAATGAAATGCATGGCTTCCATGGCTTCAAAATCATGT--------ATAATTC------------------
TGTTGTTAAACA--TCATGTAACTTATCAGCCTAAACATGGCTACTGGGTTATAGGTATGG---------
TAAATACATCTTTATATCATGGCTTTGAAAAGTGTTCTTTCACCCCAAAT------------
ATCCTCTCTCTGACTCATCTTCCCAGTCGTGTTGGAGTTTTCCTGGACAGAGAAGCTTGCACTCTCTCATTTTATGAT
GTTTCCAATCATGGAGCTCTCATGTATAAATTCTGTGAACTTTCCTTCCCTGATGCAGTTTATCCATATTTTAATCCTA
TGGAATCATCAGAGCCAATGACAGTCTGTGGACCAC------------------CCTCCTAA--------------------

>Cricetulus_griseus_Trim30b

TCAGATCTGAAAGGCATGCTGGAAGTGTTTCATGAGCTCATGGATGCCCAACACTACTGGGTTCATGTGACCCTGC
CTCGAGTCTAC---AAGAAAAATGTTATCATTAATGTGGACAAAAAACAAA----TACAACAT--------CAA-AATAA------
GAATACAAGAAATAGAAGAATGTTTAGTATTTTTGGG-ACCT-----------------------------ATGA------
TTTAGGTGTCCTTGGATATCCAGCTATCCACTCAGGGAAACATTACTGGGAAGTAGACGTGTCTAGAAGTGATGCC
TGGCTCCTGGGATTAAATGATGGAAGAT-----GTGCTCAA------------CCCCA-A------
CATCATGGATTGAATAAAAAA------------CCCTTCAAAGTCATGT--------ATAATTC------------------
TGTTGTTAAACA--TTGTGTATATTATCAGCCTAAACATGGCTACTGGGTTATAGGTAAGA---------
AGAATAGGTCTGTATATAATGCCTTTGAAAATCGTTCTGTCACCCACAATGCCAGTGTCTTGGTCCTCTCTCTGACT
CATCCTCCCAGTCGTGTTGGAGTTTTCCTGGACAGAGAAGCTTGCACTCTTTCATTTTATGATGTCTCCAACCACGG

AGCTCTCATCTATAAATTCTGTGAACCTTCCTTCCCTAATACAGTTTATCCATATTTTAATCCTATGGAATCATCAGA
GCCAATGACAGTCTGTGGACCAC------------------CCTCCTAA--------------------

>Cricetulus_griseus_Trim30c

CCAGATATGAAAGGCATGCTGCAAGTGTTTCAAGGTCTCATGGATGCCCAACGCTACTGGGTTCATGTGTTCCTGT
GTCAAGTGCAC---AATAAAAACATTGTCATTACCGTGGACAAAAGACAAA----TACAACAT--------CGA-AATGA-----
-TTATAGAAGAAAT------TTGCAAGTT---TCTGAG-ACCT-----------------------------ATGA------
TTTAGGTGTCCTTGGATATCCAGCTATCCACTCAGGGAAGCATTACTGGGAAGTAGATGTGTCTAAAAGTGATGCC
TGGATCCTGGGATTAAATGATGGAAGAT-----GTGCTCAA-------------CCCAAA------
CTTCATTCAATGAATAAAAAG------------GGCATCAAACAGG-----------ATGATTC------------------TGATGTTAAGCA-
-GCATATAAATTATCAGCCTAAACATGGCTACTGGGTTATAGGGATGA---------
CAAATAGGTCTGTATATAATGCCTTTGTAGAGTGTTCTGTCACCCACAACGCCAGTGTCTTGATCCTCTCTCTGACTC
ATCCTCCCAGTCGTGTTGGAGTTTTCCTGGACAGAGAAGCTTGCACTCTCTCATTTTATGATGTCTCCAACCATGGA
GCTCTCATCTATAAATTCTATGAACCATCCTTCCCTAATGCAGTTTATCCATATTTTAATCCTATGGAATCATCACAG
CCAATGACAGTCTGTGGGCCAC------------------CATCCTAA--------------------

>Cricetulus_griseus_Trim30d

CCAGATGTGAAAGGCATGCTGCAAGTATATCAAGGCCTTATGGATGCCCAGCGCTACTGGGTTCATGTGACCCTG
CGTCAAGTGCAC---AATAAAAATGTTGACATCAACATGGAAAAAAGACAAA----TACAATGT--------CAA-TACAG---
---TTGTAGAAGAAAT------TTGCAAGAT---TATGAG-ACCT------------------------------ATGA------
TTTAGGTGTCCTTGGAATTCCAGCTATGCAGTCAGGGAAACATTACTGGGAAGTAGATGTGTCTAGAAGTGATGC
CTGGCTCCTAGGATTAAATGATGGAAGAT-----GTGCTCAA------------CCCCA-A------
CTTCATTCAAAGAATCAAAAG------------GGCATCATGC--------------ATAGTTC------------------TCATGATGAACA--
ACATGTAAATTTTCAACCTAAATATGGCTACTGGGTTATAGGTAAGA---------
AGAATAAGTCTATATATAATGCCTTTGTAGAGTGTTCTGTCACCCACAACGCCAGTGTCTTGGTCCTCTCTCTGACT
CGTCCTCCCAGTCGTGTTGGAGTTTTCCTGGACAGAGAAGCTTGCACTCTCTCATTTTATGATGTTTCCAACCATGG
AGCTCTCATCTATAAATTCTATGAACCTTCCTTCCCTCATACAGTTTATCCATATTTTAATCCTATGGAATCATTACAG
CCAATGACAGTCTGTAGGCCGC------------------CATCCTAA--------------------

>Cricetulus_griseus_Trim30e

CCAGATGTGAAAGGCATGCTGCAAGTGTTTCAAGGCCTCATGGATGCCCAACGCCACTGGGTTCCTGTAACCCTGC
GTCAAGGGTAC---AATAACAACGTTCTCATTGATATGGAAAAAAGACAAA----TACAATAT--------TTA-AATAA------
TTATGGAAGAAAA------TTGCAAGAT---AATGAG-ACCT-----------------------------TTGC------
ATTAGGTGTCCTTGGATATCCAGCTTTCCAGTCAGGGAAATATTACTGGGAAGTAGACGTGTCTAGAAGTGATGCC
TGGCTCCTAGGATTAAATGATGGAAGAT-----GTGCTCAA------------CCCCA-A------
CTTCATTCAATGAATCAAATG------------GGCATCATGT--------------ATAATTC------------------TCATGATAAACA--
ACATGTAAATTTTCAACCTAAATATGGCTACTGGGTTATAGGTAAGA---------
AGAATAAGTCTGTATATAATGCCTTTGTAGAGTGCTCTGTCACCCACAATGCCAGTGTCTTGACCCTCTCTCTGACT
CATCCTCCCAATCGTGTTGGAGTTTTCCTGGACAGAGAAGCTTGCACTCTCTCATTTTATGATGTTTCCAACCATGG
AGCTCTCATCTATAAATTCTGTGACCCTTCCTTCCCTAATACAGTTTATCCATATTTTAATCCTATGGAAACATCACA
GCCAATGACAGTCTGTGGACCAC------------------CCTCCTAA--------------------

>Cricetulus_griseus_Trim34

CCTGATCTGAGCGACATGCTCCAAAAGTTTAGAGAGCTAACAGCCGTCCGGGGCTACTGGGTGGACTTCACATTTA
ACCCAGAAAACCTCAATTTGAATCTGATTCTTTCAGAAGACCACAGACAAG----TGGCATCT-------------GTGC------
CCATTTGGCCATT--------------------TAAG-TGTT------------------------------ATAA------
TAATAGTATCTTGGGCTCCAAATGTTTCTCCTCAGGAAAACATTACTGGGAAGTGGATGTGTCTAAGAAGAGTGCC
TGGATCCTGGGAGTTTACGCTAGAAGACGT---ACTTTAAA------------------G------TTTGATG-------------------------
TTAGACGAGGCAAAA---------ATCAGCC------------------AAATGT--------
TCACCACAGATACAAACCTCAGAATGGCTACTGGGTTATAGGGTTGC---------
AGGGTGGATCAAAGTATAGTATTTTTGAGGA---
TTCTTCCAACTGTGATCCTACTGTTCTGACCCCCTTTGTGGCTGTCCCTCTCCATCGGGTTGGGGTTTTCCTTGACTG
TGAAGAAGGCATGGTGTCCTTCTTCAATGTCACGAACCATGGGTCACTGATTTACACGTTCTCTCAGTGCTGCTTTT
CCCAGCCTGCTTATCCATACTTCAATCCTTGGGACTGCCCAGCCCCCATGACCCTGTGTCTTCAGA------------------
ACTCCTGA--------------------

>Dipodomys_ordii_Trim5a

ACTGGTCTGAGAGGAGTGCTGCACATTTTTCCAGAGCTGACTGATGTCCGCCAATACTGGGTTCACGTGATAATGA
CTACAACGAAT---GATTCCAGAATTGACATTTCCCCGGATCAGAAACAGA----TAAGATACCTTTTTATGAA-
AATCCTTTTGGTTATCAACGAAGT------TTTTCTCAACTTCAAGAA-CATA------------------------------
ATTTACCTCTGCTAGGGGCCCTGGGTGCCCCTGTAATCACATCAGGGAAGCACTACTGGGAGGTAGATGTGTCTA
AGAAAACATCCTGGCACCTGGGATTAAGTGACGGAAGATATTTCACTATTTATAAGAAAGAATTCCAGCCC------
CTAAGCAGACGTGGTGGAAGA------------CGATTCAAACCCAGTCT-------GTCATAC------------------TG------------
TTATGGAGTTCCTCAACCTAAAATTGGTTACTGGATTATAGGGATGG---------
AGGATAATTCTGGGTATAAAGTTTTTGGGGAGGATCCTACCACCCATTCTCCTTTGTCCTTGCCCCTGTCTTTGCCT
GTCGCTCTTGAACGTGTTGGGGTTTTTGTAGACTACCAGGCTGGCTCAGTCTCATTTTACAGCACTACATACAATGC
ATTTCTCATCCACAAGTTCACTAACTGTGCCTTTCCCCATGAAGTCTATCCATATTTCAATCCTTTACAATGTTCAGAA
TCCATGACTCTATGTGATCCAGCTCTTAAGCCTTTTTACCCCTTTTGA--------------------

>Dipodomys_ordii_Trim5b

CCCGACCTGACAGGAATGCTGCAAGAGTTTCAAGAGCTGACAGATGCCCACCGTTATTGGGTTCATGTGACAATA
AATGAGAACAAT---CATCGGGAAATGGTCATTTCTAAAGATCAGAGACAGA----TAAGATAT--------AAAGAACAC--
--TATTATTTTTCAGACTCACGAGGTTTCCATGTAGGTCAA-CTTT------------------------------ACTC------
ACCTGGGGCCCTGGGGCTCCCTTTGATCACATCAGGGAAACATTACTGGGAAGTAGATTTGTCTAAGAAAGCATC
CTGGCTCCTGGGGTTAAGTGATGGAAGACACT--TTACTGGG------------
CCTAATAAATCATCCTGCTGCATGGGGTTACAAA------------GTGTTCAATACTTCTCTTCCGTGGCAAAATT----------
--------TAACATTGAGAA--AGAAAAACATTATCAACGCAAAAATGGCTACTGGATTATTGGGATGG---------
AAAACAAATATGAATATATGGCTTTTGAGCCAAGTCCTACTACCTATAATTCCTTAACCTCGAAGTTCTTCCCACCTA
CTCCTTTGAAGCGTGTTGGGGTTTTTCTAGATTATGAAGCTGGCACGGTCTCATTTTATAATGTTACATACCATGGG
CTTCTAATCTATAAATTCACCAAGTGTTGCTTTACCACTGAAGTCTTTCCATATTTCAACCCTTTACAATGTTCAGAAC
CCATGATGCTATG------------------------------CTGA--------------------

>Dipodomys_ordii_Trim5c

ACTGGTCTGAGAGGAGTGCTACGAGCATTTCAAGAGCTGACTGATGCCCGCCAATACTGGGTGCACGTGAGAGTG
ACTACAAATGAC---GATTGGAGAATTGACATTTCTAAGAATCAAAGAAAAA----TAAGATAC---------CAGTATAC----
--TCCTTCAGAAAGT------
TCATAAGAAAGTTGTAGGTTTTTCACAAAATGCATTTCAATTGCCAGGAAATAATTTACCTCTCCTAGGAGTCCTAG

GTGCCCTTGTAATCACATCAGGGAAGCACTACTGGGAGGTAGATGTGTCTAAGAAAACATCCTGGCTCCTGGGGT
TAAGTGATGGAAGAC-----ACTTCAGA--------------------------T----------------------------------GCAGGCCAAGTC----------
--ATGATCA-------------------TACTG---------TTATGAAGTTCCTCGGCCTAAAACTGGTTACTGGATTATAGGGATGG--
----
AGGATAAATCTGGGTGTAAAGCTTTCGGGGAGGATCCTACCACCCATTCTCCTTTGTCCTTGCCCCTGTCTTTGCCT
GTCGCTCTTGAACGTGTTGGGGTTTTTGTAGACTACCAGGCTGGCTCAGTCTCATTTTACAGCACTACATACAATGC
ATTTCTCATCTACAGGTTCACTAACTGTGCCTTTCCCTGTGAAGTCTATCCATATTTCAATCCTTTACAATGTCCAGA
ACCCATGATTCTATG--------------------------------CTGA---------------------

>Dipodomys_ordii_Trim6

CCAGATCTGAAACGGATGCTACGAGTATTTAGAGAGCTGACAGATGTCCAGAGCTACTGGGTGGATGTGACTCTC
AACCCACACACAGCTAATTTAAATCTCGTCCTATCTAAAAATCGGCGGCAAG----TGAGGTTT--------GTG-GGAGC--
----GAAGCTGTCAGAG------CCTTCCTGTCTGGAAGAA-CATC-------------------------------ACGA------
CTGCAGTGTCCTGGGCTCCCAGCTCTTCTCCTCGGGAAAATACTATTGGGAGGTCGATGTGTACAAGAAGTCGGCC
TGGATCCTGGGCGTGTGTAGTAATTCAGG----GCAACCGA--------------------------CCT--------------------------------
TCAGCCCGTATGCAC---------ACAAGCT------------------GAACAC--------
TTGCTCCAGGTATCAGCCACAGAGCGGGTACTGGGTGATTGGTTTGC---------
AGCATAAACACGAATACAGAGCCTATGAGGA---CTCTTCTGCCT------------
CCCTGCTCCTCTCCATGACAGTGCCCCCTCGCCGCATTGGGGTTTTCTTAGACTATGAGGCTGGCACTGTCTCCTTTT
ATAATGTCACCAACCACGGCTTACCCATCTACACCTTCTCGAAATACTACTTTCCTACTGCACTCTGTCCATATTTTA
ATCCTTGCAACTGTGGAGTCCCAATGACCCTGCGTCGCCCCA------------------GCTCCTGA--------------------

>Heterocephalus_glaber_Trim6

CCAGATCTGAAAAAGATACTGCAAGTGTTTCGAGCACTCACAGATGTCCAAAGCTACTGGGTGGACGTGACCATG
AATCCACACTCTGCAAACTTAAATCTTGTCCTGTCTAAAAACCGGAGACAGG----TGAGATTC--------GTG-GGTGT--
----TAAGCTGTCCCAA------CCGTCCTATCTGGAAGAA-CATT-------------------------------ATGA------
CTGTAGCATCCTAGGCTCTCAGCACTTCTCCTCAGGAAGATACTACTGGGAGGTGGATGTGACTAAGAAGACTGCC
TGGATCCTGGGGGTGTGCAGCAAT---------TCCACGG------------ACCTCCA------CTTTCTT---------------------------
TCAGCCAGTATCCTA---------ACAGTCA------------------GAACAC--------
TTACTCCAGATACCAGCCGCAGACTGGATACTGGGTGATAGGGCTAC---------
AGCACAAACGTGAATACAGAGCCTACGAGGA---CTCTTCTACCT------------
CCCTGCTCCTCTCCATGTCTGTGCCCCCTCGCCGCATTGGGGTTTACTTAGACTATGAGGCTGGCACCGTCTCCTTCT
ATAATGTCACAAACCACGGCTTGCCCATCTACACTTTCTCTAAGTATTACTTTCCGACAGCCCTTTGTCCATATTTCA
ATCCTTGCGACTGTGTAGTACCAATGACCCTGAGGCGCCCAG------------------GCCCTTGA--------------------

>Heterocephalus_glaber_Trim34

CCAGACCTGAGCAGGATGCTGTGGATGTTTAGAGAGTTAAAAGATGTTCAAGGCTACTGGGTGGACTTTACACTG
AATCCAGTGAACCTAAATTTGAATCTTGTCCTTTCGGAAGATCACAGACAAG----TGAGACCT-------------GTTC------
CAATTTGGCCATT--------------------TAAA-TGTT-------------------------------ATAA------
TTATGGTATTTTAGGCTCCCAGTATTTCTCCTCAGAGAAACATTATTGGGAAATAGACATATCCAACAAGACTGCCT
GGATCCTGGGAGTATACTGTAGAAAACGT---TCTAAAAA------------------G------TTTGGCG---------------------------
TTAGTCAAAGCACAA---------CCCATTC------------------AAATAA--------
TTACTCTAGATACAGACCTCGGTATGGCTACTGGGTTATAGGGCTAC---------
AGAAGAAATTTGAGTATTACGCCTTTGATGA---

AACGTCTACCTTTGATCCTAAGATTTTGACCCTCTCTGTGGCTATTCCTCTTCATCGAGTTGGGATTTTCCTCAACTTT
GAAGCAGGCACTGTCTCATTTTTCAACATCACAAACCATGGGTCACTCATCTACACATTCTGTAAATGTTCCTATTCT
TGGCCCGTTTATCCATATTTCAATCCTTGGAACTGTCCAGCTCCCTTGACTTTGTGCCCACCAA------------------
GCTCCTGA--------------------

>Homo_sapiens_Trim22

CCAGATCTGAGTGGGATGCTGCAAGTTCTTAAAGAGCTGACAGATGTCCAGTACTACTGGGTGGACGTGATGCTG
AATCCAGGCAGTGCCACTTCGAATGTTGCTATTTCTGTGGATCAGAGACAAG----TGAAAACT--------GTA-CGCAC--
----CTGCACATTTAAG------AATTCAAATCCATGTGAT-TTTT------------------------------CTGC------
TTTTGGTGTCTTCGGCTGCCAATATTTCTCTTCGGGGAAATATTACTGGGAAGTAGATGTGTCTGGAAAGATTGCC
TGGATCCTGGGCGTACACAGTAAAATAAGT---AGTCTGAATAAAAGGAAGAGCTCTGGG------TTTGCTT------------
--------------TTGATCCAAGTGTAA---------ATTATTC------------------AAAAGT--------
TTACTCCAGATATAGACCTCAATATGGCTACTGGGTTATAGGATTAC---------
AGAATACATGTGAATATAATGCTTTTGAGGA---
CTCCTCCTCTTCTGATCCCAAGGTTTTGACTCTCTTTATGGCTGTGCCTCCCTGTCGTATTGGGGTTTTCCTAGACTAT
GAGGCAGGCATTGTCTCATTTTTCAATGTCACAAACCACGGAGCACTCATCTACAAGTTCTCTGGATGTCGCTTTTC
TCGACCTGCTTATCCGTATTTCAATCCTTGGAACTGCCTAGTCCCCATGACTGTGTGCCCACCGA------------------
GCTCCTGA--------------------

>Jaculus_jaculus_Trim5

TTTGATCTGCAAGTAGCGCTGAAAGCATTTCGAGAGCTAACAGAGGCTCGATGCTACAGGGTTAAGGTGACACTG
GATCCAAGTACG---AATCCACATACCATCATTATTGATGACCAAAGAAGAA----TAAAATAC------------AACCC------
ACAGAGACAAAAT------------------GATACA-CTTC-----------------------------TTTC------
CAGGGGTGTTCTGGGCTCACCAGCTATCACATCAGGGAAGCATTACTGGGAGATAGATGTGTCTATGAAAAGTGA
CTGGCTCCTGGGAGTGAGTGGTAGAAGAT-----GCTCCCAA-------------CCGACG------TTTTCTCAAC------------------
-----ATACACAACGCACAACTGCAATCTATAAGTCCGCACCGAGCCTATTTCGTGCTTCTGTAAA--
TCATCCAGACTATTGGGACTCACCTGAGTACTGGGTCATAGGGATGA---------
AGAGTGGCCACACATACAATGCTTTTCAGGGGTTCTCCCCTTTCTCCGGTAATTTCTCCATGACCTTCTTTGTGTCTG
TCTCTCCCCATCGAATTGGAGTGTTCCTGGACTATGGTGCTTCCATGGTCTCCTTTTACAACATTACAAACAATGGA
GCACTTATGTGTAGATTCCGTGATTGCCGCTTTCCCCCTGAGGTTTTTCCGTATTTTAATCCTATGAATTGCTCACAG
CCAATGACGGTGTG-----------------------------CTGA--------------------

>Jaculus_jaculus_Trim6

CCAGATCTGAAAAGGATGCTGCGAGTCTTTAGAGAGCTGACAGACGTCCAAAGGTATTGGGTGGACGTGACTCTG
AATCCACACACGGCAAATTTAAATCTCGTCCTGTCTAAAAACCGGAGACAGG----TGAGATTC--------GTG-GGTGC-
-----CAAGCTGTGTGAG------CCTTCCCGTGCGGAGGAG-CATC------------------------------ATGA------
CTGCAGCATCCTGGGCTCTCAGCTCTTCTCTTCGGGGAAGTACTACTGGGAGGTGGATGTGACGAAGAAGACCGC
CTGGATCCTGGGGGTGTGCAGTAA---------CCCGGTGGC------------CCCGACG------CCCTCTT------------------------
-TCAGCCAGTACGCTAAGGCTG---ACGTGCA------------------GGCTGC--------
TCCCTCCAGGTACCAGCCACAGAGTGGGTACTGGGTGATCGGGCTAC---------
AGTACAGGCATGAGTACAGGGCTTATGAGGA---CTCCGCCACCT------------
CCCTGCTCCTCTCCATGTCGGTGCCCCCTCGCCGGGTCGGGGTTTTCTTAGACTACGAGGCCGGCACTGTCTCCTTT
TATAATGTTACAAACCATGGCTTGCCCATCTACACCTTCTCAAAGTATTACTTTCCCACTGCCCTGTGCCCGTATTTC
AGTCCTTGTAACTGTGTGGTCCCGATGACCCTGCGTCGCCCAA------------------GCCCTTGA--------------------

>Jaculus_jaculus_Trim34

CCCGACCTGAGTGGCATGCTGCAAATGTTTCGAGAGCTAACAGATGCCCGGAGCTACTGGGTGGACATCACATTG
AGTCAGGACAACCTAAATCTGGATCTCATACTTTCTGAAGACCAGAGACAAG----TGACATGC-------------GTGC-----
-CAATATGGCCATA--------------------TAAC-TGTT------------------------------GTAA------
TTATGGTATCTTAGGGTCCCAGTACTTCTCCTCAGGGAAACATTACTGGGAAATAGACGTGTCTAAGAAGACTGCC
TGGATCATCGGAGTATACTGTAGGAAACGC---TCTGCAAA------------------G------TCTTGTG--------------------------
TTAGACAAGGCAAAA---------GACTCCC------------------AAATGT--------
TTACTCCAGAGGCAGACCCCAAGATGACTACTGGGTTCTGTGGCTAC---------
AGAATGAAACCAAGTTCGGTGCCTTCGCGAA---
CTCTGTTACTTTCAATCCTATAGTTGTAACCCTCTATATGGCGATCCCTCCCCAACGGGTGGGGGTTTTCCTCAACTA
TGAAGCAGGCACTGTCTCCTTTTTCAATATCACAAACCATGGGTCCCTTATCTACAAGTTCTCTAATTGTGGCTTTCC
TCAGCCTGTGTATCCGTATTTCAGTCCCTGGAACTGCCCGGCCCCCATGACCCTGTGTCCACCGA------------------
GCTCTTGA--------------------

>Marmota_flaviventris_Trim5a

CCTGATCTGAAGGGGATGCTGCAGGTGTCTAAAAAGCTGACAGACGTTCAACGCTACTGGGTTCACCTGACACTG
TCTCCAAGCAAC---AATCCAAATATTGTCATTTCTGAGGACCAGAGACAAT----TAAGATAT--------GAA-CCCCA-----
-ATACTGGCGCAAG------------------CGTGGG-AATT------------------------------ATCA------
TGAAGGTGTTCTGGGCTACCCACCTATCACATCAGGAAAACATTACTGGATGGTAGATGTGTCCAGGAAGGAGGC
CTGGTATCTGGGATTGTGTGATAGAAGTT-----ATTTTCAA--
----------------TCCTCA------------------
AATTTCCAAGGACGGAGTAAAAATTATCAACCTAGATGTGGCTATTGGGTTATAGGGCTTCGTACATTTGAATATA
ATGTTGAATATAATGCTTTTGGGGAGGATGCTG---
CCCGTGATCCTTTGACCTTGGTCCTCTCTGTGACTGTTCCTCCACAACGTATCGGGGTTTTCTTAGATTATGAGGCTC
GTGAACTTTCATTTTACAATGTTACAAACCATGGGTTTCTTATCTATAAATTCTCCAGATGTTCCTTTCCTAAGGAAG
TTTTTCCTTATTTCAATCCTGGGACATGTCCAGAGCCCATGACACTACACTGGCCAA------------------GCTCTTGA-----
----

>Marmota_flaviventris_Trim5b

CCTGATCTGAAGGGGATGCTGCAGGTGTCTAAAAAGCTGACAGACGTTCAATGCTACTGGGTTCACCTGACACTG
TCTCCAAGCAAC---AATCCAAATATTGTCATTTCTAAGGACCAGAGACAAT----TAAGATAT--------GTA-ACCCA-----
-ATACTGGCGCAAG------------------TGTGGG-AATT------------------------------ATCA------
TGAAGGTGTTCTGGGCTACCCACCTATCACATCAGGAAAACATTACTGGATGGTAGATGTGTCCAAGAAAAGTGC
CTGGTCTCTGGGTTTGTGTGATGGAAAAT-----ATTTCGAA-------------------------T---
----------------GTGCATC------------------TGTCCCTGGACA--
AAGTAAAAATAATCAACCTACATGTGGCTACTGGGTTATAGGTCTTC------------
ACAGATTTCAATATAATGCTTTTTGGAAGAAAGGTG---
CCCATTATCCTTTGACCTTGGTCCTCTCTGTGACTGTTCCTCCACAACGTATTGGGGTTTTCCTAGATTATGAGGCTC
GTGAACTTTCATTTTACAATGTTACAAACCATGGGTTTCTTATCTATAAATTCTCCAAATGTTCCTTTCCTAAAGAAG
TTTTTCCTTACTTCAATCCTGAGACATGTCCAGAGCCCATGACACTACACTGGCCAA------------------GCTCTTGA-----
----

>Marmota_flaviventris_Trim6

CCAGATCTGAAAAAGATGCTGCGAGTGTTTCGAGAGCTGACAGATGTCCAGAGCTACTGGGTGGACGTGACTATG
AATCCCCACACGGCTAATTTAAATCTTGTCCTGTCGAAAAACCGGAGACAGG----TGAGATTC--------GTG-GGTAC-
-----TAAGCTGTCTGAG------ACTTCCTGTCTGGAAGAA-CATC------------------------------ATGA------
CTTTAGTGTCCTGGGCTCTCAGCAGTTCTCCTCAGGAAAATACTACTGGGAAGTAGACGTGACCAGGAAGACTGCC
TGGATCCTGGGAGTGTGTAGCAAT---------TCCATGGG------------ACCTGCA------TTCTCTG--------------------------
GCAGCCAGTTTGCTA---------ACAAGCA------------------GAATGC--------
CTATTCGAGATACCAACCACAGACTGGATACTGGGTGATTGGGTTAC---------
AGCATAAACATGAATATAGAGCCTATGAGGA---TGCTTCCACTT------------
CCCTGCTCCTGTCTATGACAGTGCCCCCTCGCCGCGTTGGAGTTTTCTTAGACTATGAGGCTGGCACTGTCTCCTTT
TATAATGTCACGAACCATGGCTTGCCCATCTACACCTTCACCAAATATTACTTTCCTACTGCCGTTTGTCCATATTTTA
ATCCTTGCGACTGTGTAGTCCCGATGACCCTGCGTCGCCCAA------------------GCTCCTGA--------------------

>Marmota_flaviventris_Trim34

CCAGATCTGAGGGGGATGCTGCAGAGCTTCAGAGAGCTAACAGATGCCCAATGCTACTGGGTGGACTTAACATTG
AATCCAGGCAACTTAAATTTGAATCTTGCACTTTCGGAAGATGAGAGACAAG----TAACAAGC-------------GTGT-----
-GTATTTGGCCACT--------------------TAAG-CATT------------------------------ACGA------
TTGTGGCATCTTAAGCTCCCAACATTTCTCTTCAGGGAAACATTACTGGGAAGTTGATGTATCCAAAAAGACCAGC
TGGATCCTGGGAGTACACTGTAGAAAACGT---TCTGCAAA------------------G------TATGCTG--------------------------
AGAGAAAAGATGCGA---------ATCATAC------------------AAATGT--------
GTCCTCCACATACAGACCTAAGTACGGCTACTGGGTTATAGGTTTAC---------
AGAATAACTTTGAATATATCGTCTTTGAGGA---
TTCTTCCAGCTCTGATCCTAAGGTGTTGGCCCTCTTTATGGCTATTCCTCCCCGTCGTGTTGGGGTTTTTCTCGACTA
TGAAGCAGGCATAGTCTCATTTCTCAACGTCACAAACCATGGGTCACTCATCTACAAATTCTCTAAATGTTATTTTTC
TCAGCCTGCTTATGCATATTTCAATCTTTATAAATGTCCAGCCCCCATGACACTGTGCCTACCAA------------------
ACTGCTGA--------------------

>Marmota_marmota_Trim5a

CCTGATCTGAAGGGGATGCTGCAGGTGTCTCAAAAGCTGACAGACGTTCAACGCTACTGGGTTCACCTGACACTG
TCTCCAAGCAAC---AATCCAAATATTGTCATTTCTGAGGACCAGAGACAAT----TAAGATAT-------------GTAC--
CCCAATACTGGCGCAAG------------------CGTGGG-AATT------------------------------ATCA------
TGAAGGTGTTCTGGGCTACCCACCTATCACATCAGGAAAACATTACTGGATGGTAGATGTATCCAGGAAGGAGGC
CTGGTATCTGGGATTGTGTGATAGAAGTT-----ATTTTCAA--
TCCTCAAATTTCCAA----------------------------------GGACG---------
GAGTAAAAATTATCAACCTAGATGTGGCTATTGGGTTATAGGGCTTCACACATTTGAATATAATGTTGAATATAAT
GCTTTTGGGGAGGATGCTG---
CCCGTGATCCTTTGACCTTGGTCCTCTCTGTGACTGTTCCTCCACAACGTATCGGGGTTTTCTTAGATTATGAGGCTC
GTGAACTTTCATTTTACAATGTTACAAACCATGGGTTTCTTATCTATAAATTCTCCAGATGTTCCTTTCCTAAGGAAG
TTTTTCCTTATTTCAATCCTGGGACATGTCCAGAGCCCATGACACTACACTGGCCAA------------------GCTCTTGA-----
----

>Marmota_marmota_Trim5b

TCTAATTT--------TTCTACA-------------------------------------GTTCACCTGACACTGTCTCCAAGCAAC---
AATCCAAATATTGTCATTTCTAAGGACCAGAGACAAT----TAAGATAT--------GTA-ACCCA------
ATACTGGCACAAG------------------CGTGGG-AATT------------------------------ATCA------

TGAAGGTGTTCTGGGCTACCCACGTATCACATCAGGAAAACATTACTGGATGGTAGATGTGTCCAAGAAAAGTGC
CTGGTCTCTGGGTTTGTGTGATGGAAAAT-----ATTTCGAA-------------------------T---
----------------GTGCATC------------------TGTCCCTGGACA--
AAGTAAAAATAATCAACCTACATGTGGCTACTGGGTTATAGGTCTTC------------
ACAGATTTCAATATAATGCTTTTTGGAAGAAAGGTG---
CCCATTATCCTTTGACCTTGGTCCTCTCTGTGACTGTTCCTCCACAACGTATTGGGGTTTTCCTAGATTATGAGGCTC
GTGAACTTTCATTTTACAATGTTACAAACCATGGGTTTCTTATCTATAAATTCTCCAGATGTTCCTTTCCTAAGGAAG
TTTTTCCTTATTTCAATCCTGGGACATGTCCAGAGCCCATGACACTACACTGGCCAA------------------GCTCTTGA-----
----

>Marmota_marmota_Trim6

CCAGATCTGAAAAAGATGCTGCGAGTGTTTCGAGAGCTGACAGATGTCCAGAGCTACTGGGTGGACGTGACTATG
AATCCCCACACGGCTAATTTAAATCTTGTCCTGTCGAAAAACCGGAGACAGG----TGAGATTC--------GTG-GGTAC-
-----TAAGCTGTCTGAG------ACTTCCTGTCTGGAAGAA-CATC-------------------------------ATGA------
CTTTAGTGTCCTGGGCTCTCAGCAGTTCTCCTCAGGAAAATACTACTGGGAAGTAGACGTGACCAGGAAGACTGCC
TGGATCCTGGGAGTGTGTAGCAAT---------TCCATGGG------------ACCTGCA------TTCTCTG--------------------------
GCAGCCAGTTTGCTA---------ACAAGCA------------------GAATGC--------
CTATTCGAGATACCAACCACAGACTGGATACTGGGTGATTGGGTTAC---------
AGCATAAACATGAATATAGAGCCTATGAGGA---TGCTTCCACTT------------
CCCTGCTCCTGTCTATGACAGTGCCCCCTCGCCGCGTTGGAGTTTTCTTAGACTATGAGGCTGGCACTGTCTCCTTT
TATAATGTCACGAACCATGGCTTGCCCATCTACACCTTCACCAAATATTACTTTCCTACTGCCGTTTGTCCATATTTTA
ATCCTTGCGACTGTGTAGTCCCGATGACCCTGCGTCGCCCAA------------------GCTCCTGA--------------------

>Marmota_marmota_Trim34

CCAGATCTGAGGGGGATGCTGCAGAGCTTCAGAGAGCTAACAGATGCCCAATGCTACTGGGTGGACTTAACATTG
AATCCAGGCAACTTAAATTTGAATCTTGCACTTTCGGAAGATGAGAGACAAG----TGACAAGC-------------GTGT----
--GTATTTGGCCACT--------------------TAAG-CGTT-------------------------------ACGA------
TTGTGGCATCTTAAGCTCCCAACATTTCTCTTCAGGGAAACATTACTGGGAAGTTGATGTATCCAAAAAGACCAGC
TGGATCCTGGGAGTACACTGTAGAAAACGT---TCTGCAAA------------------G------TATGCTG--------------------------
AGAGAAAAGATGCGA---------ATCATAC------------------AAATGT--------
GTCCTCCACGTACAGACCTAAGTACGGCTACTGGGTTATAGGTTTAC---------
AGAATAACTTTGAATATATCGTCTTTGAGGA---
TTCTTCCAGCTCTGATCCTAAGGTGTTGGCCCTCTTTATGGCTATTCCTCCCCGTCGTGTTGGGGTTTTTCTCGACTA
TGAAGCAGGCATAGTCTCATTTCTCAACGTCACAAACCATGGGTCACTCATCTACAAATTCTCTAAATGTTATTTTTC
TCAGCCTGCTTATGCATATTTCAATCTTTATAAATGTCCAGCCCCCATGACACTGTGCCTACCAA------------------
ACTGCTGA--------------------

>Meriones_unguiculatus_Trim6

CCGGATCTAAAGAAGATGCTGCGAGTCTTTCGAGAGCTGACAGGCGTCCAAAGCTACTGGGTGGACGTGACTCTG
AATCCGCAGACGGCTAACTTAAATCTTGTCCTGTCTAAAAACCGGAGACAGG----TGAGGTTT--------GTG-GGTGC-
-----CGAGCCGTCCGGG------CCCTCCTGTCCGGAAGAA-CATT-------------------------------ATGA------
CTGTGGCGTCCTGGGCTCTCAGCACTTCTCCTCAGGAAAGTACTACTGGGAGGTGGACGTGAGCAAGAAGACAGC
TTGGATCCTGGGCGTGTGCAGGGCC---------CCGGTGGA------------GCCCGTG------TCCTCAT-------------------------
-TTAGCCCCA---------------GCAAGCC------------------GCGGGC--------

CAGCCCCAGGTACCGGCCCCAGAGCGGGTACTGGGTGATAGGGCTGC---------
GGCATAAGCGCGAGTACAGAGCCCACGAGGA---CCCCTCCCCCT------------
CCCTGCTCCTGTCCCTGAGCGTGCCGCCTCGCCGCGTAGGGGTCTTCTTAGACTACGAGGCGGGCACGGTCTCCTT
TTATAACGTCACAAACCACGGCTTACCCATCTACACCTTCTCCAAGTATTACTTCCCTGCTGCCCTTTGTCCGTACTTT
AATCCCTGCAGCTGTGTGGTCCCGATGACCCTGCGGCGCCCCA------------------GCGCG----------------------

>Meriones_unguiculatus_Trim12

CCCGATCTGCAAGGCATGCTGCAAGTGTTGCAAGAGGTCACAGAGGCCCAGCGCTACTGGGTTCGAGTGACACTG
GTTGAAAACAAC---CATCCAAAAATTGCCATTACTGTGGACAAGAAACAAA----TACGATAT--------GAA-GACCA---
---TCAAACAAGAAAT------TCAAAAGCTAGGGATGAA-AGCT------------------------------GTCA------
TGAAGGCGTCATGGGACACCCAGCTCTCCAATCAGGAAAGCATTACTGGGAAGTAGATGTGTCTGGAAAAAGTGC
CTGGGTCCTGGGATTATGTGATGGAAGTTAC---CTCTTCAA------------TCCCATA------TTTCTCT-----------------------
---CACACCCAAGTCCATTAT------TTCGTTT------------------GGGTCTTAGTAA--
TGATTTACACTATCAACCTAAATATGGGTTCTGGGTTATAGGGCTGC---------
AGAAAAAGTACGTGTATAATGTTTTTGAGGAGTGTTCCCTCACAGGCAAGCCCAGCACCTTGACACTCTCTCTCAT
GGTTCCTCCCTGTCGTGTTGGCGTTTTCCTTGACTATGCAGCTGGCACGCTCTCGTTTTACAATATTTCCCACCATGG
GACTCTCATCTACAGATTCTGTTCAGTTTCCTTTCCTGACAGGGTTTTTCCATATTTTAATCCTATGAGGTGTTCAGA
GCCAATGACAGTATGCTGGCCAG------------------ACTCTTAG--------------------

>Meriones_unguiculatus_Trim30

CCAGATCTGAAAGGCATGTTGAAAGCATTTCAAGGGATTATGGATGTCCAGCAATACTGGGTTCGTGTGACTCTGT
CTCCAAACAAC---AGTGCAAA---TGTCCTTTATGAAAAGGAAAGACAAAAAATTACGATGA--------GTACAATGA---
---TTATAAAGGTGTT------TTGCAATTT---TCTGAG-AGTT------------------------------ATTA------
CTTGGGTGTCCTGGGAGGTCCACCCCTCCAGTCAGGGAAATATTACTGGGAAGTAGACGTGTCTGGATGTAAAGC
CTGGCTCCTTGGATTAAATAATGGACAACGTGCTCCACTTCA------------------G------
CTTCATCCAGGGCAGCCAGGG------------ATCTTCACATCCCAAT--------ATAATTC------------------
TGTTGATAAACA--ACGTGTAAATTATCAGCCTAAATATGGCTACTGGGTTATAGGGGTAA---------
AGGATAGTTCTTTATAT---
GCCTTTGATCAGTGTTATGAGACCAAAAATCCCAGTGTCTTGGCCCTCTCTCAGCGAGGCCCTCTCAGTCGTGTTGG
AGTGTTTCTGGACTACAAGGCTTCCACTCTCTTGTTCTATAACGTTTCTAACTGTGGAGCTCTCATCTACAGATTCTA
TGACCGTTCCTTCCCTCACACACTTTATCCATATTTTAATTCTATGGGATGTTCAAAGCCAGTGACCATATGTGAGCC
AC------------------CCTCGTAA--------------------

>Meriones_unguiculatus_Trim34

CCCGATCTGAGCGACATGCTACAGAAGTTTAGAGAGCTGACAGCTGTCCGGAGTTACTGGGCGGACTTCACATTC
AATCCAGAAAACCTCAATTTGAATCTTACTCTTTCAGAAGATCACAGACAGT----TGACTTCT-------------GTGC------
CCATTTGGCCATT--------------------TAAG-TGTT------------------------------ATAA------
TAATGGTATCTTGGGCTCCAAATGTTTCTCCTCAGGAAAACATTACTGGGAAGTGGATGTGTCTCAGAAGAATGCC
TGGATCCTGGGAGTGTATGCTCGGAAGCGA---AATTTAAA------------------G------TTTGATG--------------------------
TTAGAAGAGGCAAAA---------GTCAGCC------------------AAGTGT--------
TCACCACAGATACAAACCTCAGAATGGATACTGGGTTGTAGGGTTAC---------
AGGATGGATCGAAGTATAGTATCTTTGAGGA---
TTCTTGCAACGGTGACCCTACAGTTCTGACCCCCTTCGTGGCTGTCCCCCTCCATCGGGTCGGGGTTTTCCTCGACT
GTGAAGAGGGCATAGTGTCCTTCTTCAATGTCACAAACCATGGGTCACTCATCTACCAGTTCTCTCAATGCTGCTTT

TCCCAACCTGCATATCCATACTTCAATCCTTGGGACTGTCCAGCCCCCATGACGCTGTGTCTTCTCA-----------------
GCTCCTGA--------------------

>Microtus_ochrogaster_Trim6

CCGGACCTGGAAAAGATGCTGCGAGTCTTTCGAGAGTTGACAGACGTCCAGAGCTACTGGGTGGATGTGACGCT
GAACCCACAGACGGCCAATTTAAATCTTGTTCTGTCTAAGAACCGGAGACAGA----TGAGGTTT--------GTA-
GGTGC------CAAGCTGTCCGAG------TCTTCCTGTCTGGAAGAG-CATT-----------------------------ATGA------
CTGCAGTGTCCTGGGCTCTCAGCACTTCTCCTCGGGAAAATACTACTGGGAAGTGGACGTGACCAAGAAGACGGC
TTGGATCCTGGGTGTATGCAGTAAC---------CCAGTGGA------------ACCCGCA------TTCTCTT-------------------------
TCAGCCAGTATCCCA---------GCAAGCA------------------GAGCTC--------
CTACTCCAGGTATCAGCCCCGGAGCGGATACTGGGTGATTGGGCTGC---------
AGCATAAACACGAATACAGAGCCTATGAGGA---CTCCGCCACTT------------
CCCTGCTGCTGTCCATGGCAGTGCCACCTCGCCGCGTAGGGATTTTCTTAGACTATGAGGCTGGCACTGTCTCCTTC
TATAATGTCACAAACCATGGCTTGCCCATCTACACCTTCTCTAAGTATTACTTTCCCACTGCGCTTTGTCCCTATTTCA
ATCCCTGCAGTTGTGTGGTCCCAATGACCTTGCGTCGCCCGA------------------GCTCCTGA--------------------

>Microtus_ochrogaster_Trim12

CCTGATCTACAAGGCATGCTGCAAGTGCTTCAAGAGATCACAGAGGCCCAGCGCTACTGGGTTCAAGTGACGCTG
GTTGAGAACAAC---AATCCAAACACTGCCATTTCTGCAGACAAAAGACAAA----TAAGATAT------------GAAGA------
ACAAACAAGAAAT------TTTGCATCTGGGTGTGAG-AACT------------------------------CTCA------
TGATGGTGCCCTGGGCTACCCATCTATCCAATCAGGAAAGCATTATTGGGAAGTAGATGTGTCTGGAAAAGGTGC
CTGGGTTCTGGGATTAAGTGATGGAAGCTAC---CTCTTCAA------------TCCGATA------
TTTCATTCAAATGCTGAAA--------------GCCGCCTAAATCCCTTAT------TTCGTTT------------------GGGTATTAGCAA-
-TAATTCGCATTATCAACCTAAATATGGCTTCTGGGTTATAGGGCTGT---------
GGAACAAGTGCGTGTATAATGCTTTTGAGGAGTGTGCCTTCACAGGCAAGCCCAGTGTCTTGACCCTCTCGCTGAT
GGTTCCTCCCTGTCGAGTGGGCGTTTTCCTTGACCATGCAGCCGGCACTCTCTCGTTTTACAATATTTCCCACCATG
GGACCCTTATCTACAGATTCTGTGCAAATTCCTTTCCTGATAAAGTTTTTCCATATTTTAATCCTAGGGGATGTTCAG
TGCCCATGACAGTATGCTGGTCAG------------------ACTCGTAA--------------------

>Microtus_ochrogaster_Trim30a

CCGGATCTGAAAGGCATGATGCAAGTGTTTCAAGGGCTCCTGGATGCCCAGCGCCACTGGGTTCACGTGACCTTG
CCTCAACTCAAC---AATAAAAACATTGTCATTAACGTGGACAAAAGACAAA----TACAACAT--------AGA-AGTGG----
--CTATAGAAGAAAT------TTGCAAGTT---TCCGAT-TCCT-----------------------------ATGA------
TTTAGGTGTCCTAGGGTATCCAGCTATCTACTCAGGGAAACATTACTGGGAAGTGGACGTGTCTAGATGTGATGCC
TGGATCCTGGGAATAAATGATGGAAGAT-----GTGCTCAA------------CCCCA-G------
CTTCGTGCAGTGAATGAAAAG------------GGATTCAGAGTCATAA--------AAAATTA------------------
TATTGTTAAACA--GGATGTAAAATATCAGCCCCAATATGGCTACTGGGTTATAGGAATTA---------
CAAACACGTCTGTATGTAACGTCTTTGAGGTGTGCTCTGTTCCTCAAAACGCTAGTGTCTCGTTCCTTTCTCTGACTG
GCTCTCCCACTGGTGTTGGAGTTTTCCTGGACCGAGAAGCTTGCACTCTCTCGTTTTATGATGTTTCCAACCATGGA
GCTCTCATCTATAGATTCTATGAACCGAACTTCCCTAATGCCGTTTATCCATATTTTAATCCTATGGAGTCATCAGGG
CCAATGACAGTCTGTGGGCCAC------------------CCTCTTAA--------------------

>Microtus_ochrogaster_Trim30b

CCGGATCTGAAAGGCATGATGCAAGTCTTTCAAGGGCTCCGGGATGCCCAGCGCTACTGGGTTCATGTGAACCTG
CTTCAACTTAAC---AATAAAAACATTGTCATTAACGTGGACAAAAGACAAA----TACGACAT--------AGA-AGTGG----
--CTATAGAAGAAAT------TTGCAAGTT---TCTGAG-TCCT------------------------------ATGA------
TTTAGGTGTCCTAGGGTATCCAGCTATCTACTCAGGGAAACATTACTGGGAAGTGGACGTGTCTAGATGTGATGCC
TGGATCCTGGGAATAAATGATGGAAGAT-----GTGCTCAA------------CCCCA-G------
CTTCATGGAGTGAATCAAAAG------------GGCTTCAGAGTCATAA--------AAAATTC------------------
TGTTGTTAAACA--GGATGTAAGATATCAGCCCCAATATGGCTACTGGGTTATAGGAATTA---------
CAAACAGGTCTGTATATAATGTCTTTGAGGTGTGCTCTGTCCCTCAAAATGCTAGTGTCTCATTCCCTTCTCTGACTG
GCTCTCCCACTCGTATTGGAGTTTTCCTGGACCGAGAAGCTTGTACTCTCTTGTTTTATGATGTTTCCAACCATGGA
GCTCCTATCCATAGATTCTGCGACTGTTCTTCCTCTGGTGCGCTTTATCCATATTTTAACCCTATGGAGTCATCAGAG
CCAATGACAGTCTGTGGACCAC------------------CCTCCTAA--------------------

>Microtus_ochrogaster_Trim30c

CCGGATCTGAAGGGCACGCTGCAAGTGTTTCAAGGGTTCCGGGATGTCCAGTGCCACTGGGTACACGTGACCCTG
CAACAACCCAAA---AATAAAAACATTGTCATTAACGTGGACAAAAGACAAA----TACAACAT--------GGA-AGTGG---
---TCATAGAAGAACT------TTGCAAGTT---TCTGAG-TCCT------------------------------ATGA------
TTTAGGTGTCCTAGGGTATCCAGCCATCCACTCAGGAAAACATTACTGGGAAGTAGACGTGTCTAGATGTGATGCC
TGGCTCCTGGGAATAAATGATGGAAGAT-----GTGCTCGA-------------CCCCAG------
CTTCCTTCAATGAATCAACAG------------GGCTTCAAAGCCAAATATG-----ATCATTC------------------
TGATAATAAACA--GCATGTAAATTATCAGCCCAAATATGGCTACTGGGTTATAGGGATGA---------
TGGACGGGTCTGTATATAATGCCTTTGAAGAGTGTTCTGTCACCCACAATGTCAGTGTCTTGCTCCTCTCTCTGACT
TGTTCTCCAACTCGTGTTGGAGTTTTCCTGGACCGAGAAGCTTGCACTCTCTCATTTTATGATGTTTCCAACCATGG
AGCTCTCATCTATAGATTTTCTAAACCTTCCTTCCCTAATGTGGTTTATCCATATTTTAATCCTATGGGATGTTCAGA
GCCATTGACAGTCTGTGGGCCCC------------------CCTCCTAA--------------------

>Microtus_ochrogaster_Trim30d

CCGGATCTGAAGGGCATGCTGCAAGTGTTTCAAGGGCTCTTGGATGCCCAGCACCACTGGGTTCACGTGACCCTG
AATCAAACACAA---GATAAAAACATTGTCATTAATGAAGACAAAAGACAAA----TACAACAT--------CGA-AATGG---
---TAATAAAAGAAAT------TCTCAGATT---TCTAAG-ACCT------------------------------ACAA------
ATTGGGTGTCCTAGGGTATCCAGCTATTCACTCAGGGAAACATTACTGGGAAGTAGACGTGTCTAGATGTGATGC
CTGGCTCCTGGGAATAAATAATGGAAAAT-----GTGCTAAA------------CCCCA-G------
CTTTCTGCAGCAAATGAAAAG------------AACTTCAATGTCAAAT--------ATAATTC------------------
TAATGTTAAACGACACATGCAAATTATCAGCCCAAATGTGGCTACTGGGTTATAGGGATGA---------
TGGACGGGTCTGTATATAATGCCTTTGAAGAGTGTTCTGTCACCCACAATGCCAGTGTCTTGCTCCTCTCTCTGACT
CGTCCTCCCACTCGTGTTGGAGTTTTCCTGGACCGAGAAGCTTGCACTCTCTCATTTTATGATGTTTCCAACCATGG
AGCTCTCATCTATAGATTCTATGAACCGAACTTCCCTAATGCAGTTTATCCATATTTTAATCCTATGACATGTTCAGA
GCCATTGACAGTCTGTGGGCCAC------------------CCTCCTAA-------------------

>Microtus_ochrogaster_Trim30e

CCAGATCTGGAAGGCATGCTGCAAATATTTCAAGGGTTCCGGGAAGCCCAGCGCCACTGGGTACATGTGACCCTG
CATCAACTCAAC---AATAAAAACATTGTCATTAACGTGGACAAAAGACAAA----TACAACAT--------AGA-ACTGG----
--TTATAGAAGAACT------TTGCAAGTT---TCTGAG-TCCT------------------------------ATGA------
TTTAGGTGTCCTAGGGTATCCAGCTATCCACTCAGGGAAACATTACTGGGAAGTAGACGTGTCTAGATGTGATGCC
TGGCTCCTGGGAATAAATGATGGAAAAT-----GTGCTCAA------------CCCCA-G------

CTTCCTTCATTGAATCAACAG------------GGCGTGAAAGCCAAAT--------ATAATTC------------------
TGATGTTAACCA--ACATGTAAATTATCAGCCCAAATGTGGCTACTGGGTTATAGGGATGA---------
CAAACAGGTCTGTATATGAAGCCTTTGAAGAGTGTTCTGTAACCCACAAAGCCCGTATCTTGGTCTTCTCTCTGACT
CGTCCTCCCACTCGTGTTGGAGTTTTCCTGGACTGTGAAGCTTGCACTCTCTCATTTTATGATGTTTCCAACCATGGA
GCTCTCATCTATAAATTCTGTGAATCTTCCTTCCCTAATGTGGTTTATCCATATTTTAATCCTATGGGATGTTCAGAG
CCATTGACAGTCAGTGGGCCAC------------------CCTCCTAA--------------------

>Microtus_ochrogaster_Trim30f

CCGGATCTGAAGGGCATGCTGCAAGTGTTTCAAGGGTTCCGGGATGCCCAGCGCCACTGGGTTCACGTGATCCTG
CAACAACCCAAA---AATAAAAACATTGTCATTAACGTGAACAAAGACAAA----TACAACAT--------AGA-AGTGG---
---TTATAGAAGAACT------TTGCAAGTT---TCTGAG-TCCT------------------------------ATGA------
TTTAGGTATCCTGGGGTTTCCAGCTCTGCGCTCAGGGAAACATTACTGGGAAGTAGACGTGTCTAGATGTGATGCC
TGGCTCCTGGGAATAAATGACGGAAGAT-----GTGCTCAA------------CCCCA-G------
TTTAGTACAGTGAATGAAAAG------------GGCTTCAGAGTCAAAT--------ATAATTC------------------
TGATGTTAACCA--AGATGTAAATTATCAGCCCAAATGTGGCTACTGGGTTATAGGGATGA---------
CGGACAGGTCTGTATATAATGCCTTTGAAGAGTGTTCTGTTACCCACAATGCCAGTGTCTTGCTCCTCTCTCCGACT
CATCCTCCCACTCGTGTTGGAGTTTTCCTGGACCGAGAAGCTTGCACTCTCTCATTTTATGATGTTTCCAACCATGGA
GCTCTCATCTACAGATTCTATGAACCGAACTTCCCTAATGCAGTTTATCCATATTTTAATCCTATGATGTGTTCAAAG
CCATTGACAGTCTGTGGGCCAC------------------CTTCCTAA--------------------

>Microtus_ochrogaster_Trim34

CCTGATCTGAGCGACATGCTCCGACAGTTTAGAGAGTTAACAGCTGTCCGCGGCTATTGGGCGGATTTCACATTTA
ATCCAGAAAACCTAAATTTGAATCTTATTCTTTCAGAAGATCACAGACAAG----TGACATCT-------------GTGC------
CCATTTGGCCATT--------------------TAAG-TGTT------------------------------ATAA------
TAATGGTATCTTGGGCTCCAAATGTTTCTCCTCAGGGAAACATTACTGGGAAGTGGATGTGTCTAAGAAGAATGCC
TGGACACTGGGAGTTTACGTTAGAAAACGT---ACTTTAAA------------------G------TTTGATG--------------------------
TCAGGAGAGGCAAAA---------ACCAGCC------------------AAATGT--------
TTGCCACAGATACAAACCTCAGAATGGCTACTGGGTTATAGGGTTAC---------
AGGATGGATCAAAGTATAGTATCTTTGAGGA---
TTCTTCCAACTGTGACCCTACCATTCTGACCCCCTTTGTGGCTGCCCCTCTCCATCGGGTTGGGGTTTTCCTTGACTG
TGAAGAGGGCATAGTGTCCTTCTTCAATGTCACAAACCATGGGTCACTCATTTACACATTCTTTAAATGCTGCTTTTC
CCACCCTGCTTATCCATACTTTAATCCTTGGGATTGCCCAGCTCCCATGACCCTGTGTCCTGTGA------------------
ACTCCTGA--------------------

>Mus_musculus_Trim5

CCTGACCTGCAAGGCATGCTGCAAGTGCTGCAAGAGGTCACAGAGGCTCAACGCTACTGGGTTCAGGTGACCCTG
GTTCAAAATAAC---CATCCAAACATTGCCATTACTGACGACAAAAGACAAG----TACGATAT--------GAA-GACCA----
--CCAAGCAAGTAAT------CTTATACACGAGTGTGAA-AACT------------------------------CTCA------
TGAAGGCGTCCTGGGACATCCAGCTATCCAATCTGGAAAACATTACTGGGAAGTAGATGTGTCTGGAAAAGGTGC
TTGGGTTCTGGGGTTAAGTGATGGAAGCTAC---CTCTTCTG------------TCCAATA------TTTCATTCAAATGCTGAAA-
-------------GAAATCG-----------------TTTATTTTGTAG-------------GGGGATTAAGAA--
TGATTCACATTATCAACCAAAATATGGCTTCTGGGTTATAGGGCTGT---------
GGAAAAAGTCTGTGTATAATGCTTTTGAGGAGTGTCCTTTCACAGGCAAGCCCAGTGTCTTGACCCTGTCTCTGAT
GGTCCCGCCCTGTCGTGTTGGTGTTTTCCTTGACTATGCAGCTGGGACTCTCTCATTTTACAATATTTCCAACAATG

GGACTCTTATCTACAGATTCTGCACAGCTTCCTTTCCTGATAGGGTTTTTCCATATTTTAATCCCACGGGAAGTTCAG
AGCCGATGACAATTTGCTGGCCAG------------------ACTCTTAA--------------------

>Mus_musculus_Trim6

CCGGATCTGAGGAAGATGCTAAAAGTCTTTAGAGAGCTGACAGATGTCCAAAGCTATTGGGTGGACGTGACACTG
AATCCACAGACGGCTAATTTAAATCTTGTCCTGTCGAAAAATCGGAGACAGG----TGAGGTTT--------GTG-GGTGC-
-----CCAGCTGTCCGAG------CCATCCAGTCTGGAAGAA-CATT-----------------------------ATGA------
CTGTAGTGTCCTGGGCTCTCAGCACTTCTCCTCAGGAAAATACTACTGGGAAGTGGACGTGAGCAAGAAGACGGC
ATGGATCTTGGGTGTATGCAGTACC---------CCGGTGGA------------TCCCATG------TTCTCTT-------------------------
TCAGCCAGTACTCCA---------GCAAGCA------------------GGGCGC--------
CTACTCCAGGTATCAGCCACAGTGTGGATACTGGGTGATTGGCTTGC---------
AGTGTAAGCACGAGTACAGAGCCTATGAGGA---TTCCTCCCCGT------------
CCCTGCTCCTCTCCATGACCGTGCCACCTCGACGCATAGGGATTTTCTTAGACTGTGAGGCTGGCACGGTCTCCTTT
TATAATGTCACAAACCACGGCTTGCCCATCTACACCTTCTCGAAGTATTACTTTCCTTCTGCCCTTTGTCCCTATTTTA
ATCCCTGCAGCTGTATAGTCCCGATGACCCTGCGGCGGCCAA------------------CTTCCTGA--------------------

>Mus_musculus_Trim12c

CCTGACCTGCAAGGCATGCTGCAAGTGCTGCAAGAGGTCACAGAGGCTCAACGCTACTGGGTTCAGGTGACGCTG
GTTCAAAACAAC---AATCCAAACATTGCCATTACTGATGACAAAAGGCAAA----TACGATAT--------GAA-GACCT----
--CCAAGCAAGGAAT------CTTGAACATGGCTGTGAA-AACT------------------------------CTCA------
TGAAGGTGTCCTGGGACACCCAGTTATCCAATCAGGAAAACATTACTGGGAAGTAGATGTGTCTGGAAAAGGTGC
TTGGGTTCTGGGGTTAAGTGATGGAAGCTAC---CTCTTCAA------------TCCAATA------
TTTCGTTCAAATGCTGAAAGAAA----------CCCTTTATTTCGCAG---------------------------------GGGGATTAAGAA--
TGATTCACATTATCAACCAAAATATGGCTTCTGGGTTATAGGGCTGT---------
GGAAAAAGTCTGTGTATAATGCTTTTGAGGAGTGTCCTTTCACAGGCAAGCCCAGTGTCTTGACCCTGTCTCTGAT
GGTCCCGCCCTGTCGTGTTGGTGTTTTCCTTGACTATGCAGCTGGGACTCTCTCATTTTACAATATTTCCAACAATG
GGACTCTTATCTACAGATTCTGTACAGCTTCCTTTCCTGATAGGGTTTTTCCATATTTTAATCCCATGGGAAGTTCAG
AGCCCATGACAATTTGCTGGCCAG------------------ACTCTTAA--------------------

>Mus_musculus_Trim30a

CCAGATCTGAAAGGCATGCTGCAAGTGTATCAAGGACTCATGGATATCCAGCAATACTGGGTTCATATGACTCTAC
ATGCAAGGAAC---AATGCAGTCATTGCCATTAACAAAGAAAAAAGACAAA----TACAGTAT--------AGA-AGTTA-----
-CAATACGG------------TTCCAGTT---TCTGAG-ATCT-----------------------------ACCA------
TTTGGGTGTCCTGGGATATCCAGCTCTTTCCTCAGGGAAGCATTACTGGGAAGTAGACATATCTAGAAGTGATGCC
TGGCTCCTCGGATTAAATGACGGAAA--GT---GTGCTCAA------------CCCCA-A------
CTTCACTCAAAGGAAGAAATG------------GGCATCAAAAAAAACC--------TTCATTC------------------
TCAGATCAAACA--AAATGTATTGTTTCAGCCTAAATGTGGCTACTGGGTTATAGGGATGA---------
AGAATCCGTCTGTATACAAGGCCTTTGATGAGTGTTCTATCACCCACAATTCCAGTATCCTGGTCATCTCTCTGCCT
GATCGTCCCAGTCGTGTCGGAGTTTTCCTGGATCGGAAAGCTGGCACTCTCTCATTTTATGATGTTTCTAACTGCGG
TGCTCTCATCTATAGGTTCTATGACCCTGCCTTCCCTGTTGAAGTCTATCCATATTTTAATCCTATGAAATGTTCAGA
GCCAATGACTATATGCGGGCCAC------------------CCTCCTAA--------------------

>Mus_musculus_Trim30b

---A----
TGCAGCAT--------GCA-AATTC------CTATAGAAGGAAT------TTGCAAATT---TCTGAG-ATCT---------------------------
---ATCA------
TTTTGTTGTCCTGGGATATCCAACTATTGGCACAGGGGAACAATACCTGGAAGTAGACATGTCTAGAAGTGATGCC
TGGCTCCTGGGATTAAATCATGGACC--AC---ATGCTGCA------------CCCCA-A------CTTTGTTCTATGAATGAAATG-
-----------TTCCCTAATGTCAAATT-----TCATGATAC------------------TGATAT---ACA--
GCATGAAACTTATCAGTCTAAATATGGCTACTGGATTATAGGGATGA---------AGTATAGGTCTGTATA---
TGCCTTTGATAAGTGTCCTGTCACCTACAAT-
TCAGTGTCTTGGCCCTCTCTCTGTCTGGTCCTCTCAGTCATGTTGGAGTTTTCCTGTCCTGGGAAGCTAGGACTCTCT
CATTTTATGATGTTTCTACCTATGAAGCTTTCATCTATTGGTTCTATGACCCTTCCTTCCCTGATACGG----
TCAATATTTTAA---

>Mus_musculus_Trim30c

CAGGATCTGAATGTCATGCTGCAAGCAATTCAAGGGCTCATGTATGTCCGACGATATTGGGTTCATGTGACTCTCT
ATGCAAACAAC---CATGCAGTAATTGCCATTAACAAAGAAAAAAGACAAA----TACAACAT--------ACA-AGTTA-----
-CTATAAAAGGAAT------CTGCAAATT---TCTAAG-ACCT------------------------------ATAA------
CTTGGGTGTCCTGGGATATCCAGCTATCTGCTCAGGGAAACATTACTGGGAAGTAGACGTGTCTAGAAAAAAGAC
CTGGATCCTGGGATTAAATGATGGACTGT-----GTGTTCAA-------------CCTCAA------
CTTCATTCTATAAGTGAAATG------------GGCTTCAAAGTCAAAT--------ATAATTC-----------------
TAGTGTGAAACA--ATGTGGTAATTATCAGCATAAATATGGCTACTGGGTTATAGGGATGA---------
AGAATTGGTCTGTATACAATGCCTCTGATGAGTGTTCTGTTACCCACAATTCCAGTGTTTTGGCCCTCTCTCTGTCTG
GTCCTCCCAGTCGTGTTGGAGTTTTCCTGGACCGGGAAGCTTGTACTCTCTCATTTTATGATGTTTCTAACTTTGGA
GCTCTCATCTATAGGTTCTATGAACCTTTCTTCCCTCATACAGTCTATCCATATTTTAATCCTATGGAATGTTCAGAG
CCAATGACAGTATGTGGGCCAT------------------CCTCATCAATCTCTGTGGAAACACAGTC

>Mus_musculus_Trim30d

CCACATTTGAAAGGCATGCTACAGTCATTTGAAGGGCTCATGGATGTTCAGCAATACTGGGTTCATATGACTCTAC
ATGCAAGGAAC---AATGCAGTCATTGCCATTAACAAAGAAAAAAGACAAA----TACAGTAT--------AGA-AGTTA-----
-CAATACGG------------TTCCAGTT---TCTGAG-ATCT------------------------------ACCA------
TTTGGGTGTCCTGGGATATCCAGCTCTTTCCTCAGGGAAGCATTACTGGGAAGTAGACATATCTAGAAGTGATGCC
TGGCTCCTCGGATTAAATGACGGAAA--GT---GTGCTCAA------------CCCCA-A------
CTTCACTCAAAGGAAGAAATG------------GGCATCAAAAAAAAAT--------ATCATTC-----------------
TCATATTAAACA--AAATGTAACGTTTCAGCCTAAATGTGGCTACTGGGTTATAGGGATGA---------
AGAATTCATCTGTATACAATGCCTTTGATGAGTGTTCTATCACCCACAATTCCAGTGTCTTGGCCCTCTCTCTGCCTG
ATCGTCCCAGTCGTGTAGGAGTTTTCCTGGACCAGGAAGTTTGCACTCTCTCATTTTATGATGTTTCTAACTCTGGA
GCTCTCATCTATAGATTCTATGACCCTTCCTTCCCTGTTGAAGTCTATCCATATTTTAATCCTATGGAATGTTCAGAG
CCAATGACAGTATGCGGACCAC------------------CATCCTAA-------------------

>Mus_musculus_Trim34a

CCTGATCTGAGTGGCATGCTACAAAAGTTTAGAGAGCTAACAGCTGTCCGGGCCTACTGGGACAACTTCACATTTA
ATCCAGAAAACCTAAATTTGAATCTTATACTTTCAGAAGACCACAGACAAG----TGACATCT-------------GTGT------
CCATTTGGCCCTT--------------------TAAG-TGTT------------------------------GTAA------
TAACGGTATCTTGGGCTCCAAATGTTTCTCCTCAGGAAAACATTACTGGGAAGTGGATGTGTCTGAAAAGAAGGC
CTGGACCCTGGGAGTTTACACTAGAAAACGA---ACTTTAAG------------------G------TTTGATG--------------------------

TTAGACAACGCAAAG---------GTCAGCC------------------AAATGG--------
TTACCACAGATACAAACCACAGAATGGCTACTGGGTTATAGGGTTAC---------
AGCATGGATCGAAGTATAGTATCTTTGAGGA---
TTCTTCCAACTGTGACCCTACTGTTCTGAACCCCTTTGTGGCCACCCCTCTCCATCGGGTTGGGGTTTTTCTTGACTG
TGAAGAGGGCACAGTATCCTTCCTCAATGTCACCAACCATGGATCACTCATTTACAAGTTCTCCCAATGCTGCTTTT
CCCAACCTGCCTATCCATACTTCAATCCTTGGGACTGTCCAGCCCCCATGACCCTGTGTCCTCTGA------------------
ACTCCTGA--------------------

>Mus_musculus_Trim34b

CCTGATCTGAGTGGCATGCTACAAAAGTTCAGAGAGCTATCAGCTGTCCGGGCCTACTGGGACAACTTCACATTTA
ATCCAGAAAACCTAAATTTGAATCTTATACTTTCAGAAGACCACAGACAAG----TGACATCT-------------GTGT------
CCATTTGGCCCTT--------------------TAAG-TGTT------------------------------GTAA------
TAATGGTATTTTGGGCTCCAAATGTTTCTCCTCAGGAAAACATTACTGGGAAGTGGATGTGTCTGAAAAGAATGCC
TGGACCCTGGGAGTTTACACTAGAAAACGA---ACTTTAAG------------------G------TTTGATG-------------------------
TTAGACAACGCAAAG---------GTCAGCC------------------AAATGG--------
TTACCACAGATACAAACCACAGAATGGCTACTGGGTTATAGGGTTAC---------
AGCATGGATCGAAGTATAGTATCTTTGAGGA---
TTCTTCCAACTGTGACCCTACTGTTCTGAACCCCTTTGTGGCCACCCCTCTCCATCGGGTTGGGATTTTCCTTGACTG
TGAAGAGGGCACAGTGTCCTTCCTCAATGTCACCAACCATGGATCACTCATTTACAAGTTCTCCCAATGCTGCTTTT
CCCAACCTGCCTATCCATACTTCAATCCTTGGGACTGTCCAGCCCCCATGACCCTGTGTCCTCTGA------------------
ACTCCTGA--------------------

>Octodon_degus_Trim6

CCAGATCTGAAAAAGATGCTGCAAGTGTTTCGAGAGCTCACAGATGTCCAGAGTTACTGGGTGGATGTGACTATG
GATCCACACTCTGCTAATTTAAATCTTGTGCTGTCTAAGAACCGGAGGCAGG----TGAGATTT--------GTG-GGTGC--
----TAAGCTGGCCGGA------CCCTCCTGTCTGGAAGAG-CACT------------------------------TTGA------
CTGTAGTGTGTTGGGCTCTCAGCGCTTCTCCTCAGGAAAGTTCTACTGGGAGGTGGATGTGACCAAGAAGATGGC
CTGGATCTTGGGTGTGTGCAGCAGC---------TCCATGGA------------CCCTCCA------TCTGCTT-------------------------
GCAGCCGCTACCCTA---------ATGGTCA------------------GAACAC--------
TTACTCTAGATACCAGCCACAGACCGGGTACTGGGTGATCGGCCTAC---------
AGCACAAACGTGAATACAGGGCGTATGAGGA---CTCTGCCAGCT------------
CCCTGCTCCTCTCCATGACTGTGCCGCCCCGCCGCATTGGGGTTTACTTGGACTATGAGGCTGGCACCGTGTCTTTC
TATAACGTCACAAACCATGGTTTGCCCATCTACACTTTCTCTAAGTACTATTTCCGACAGCCCTTTGTCCCTATTTCA
ATCCTTGTGACTGTGTTGTCCCAATGACCCTGAGGCGTCCAG------------------GTCCTTGA--------------------

>Octodon_degus_Trim34

CCAGACCTGCGCAGGGTGCTGTGTCTGTATAGAGAGCTAAAAGATATCCAAGGATACTGGGAGGATTTTACACTG
AATCCAGTCAACCTGAATTTGAATCTTGTCCTTTCGGAAGATCACAGACAAG----TGAGAGCT-------------GTTC------
CCATTTGGCCATT--------------------TAAG-AGTG-----------------------------ATAA------
TTATGGTATTTTAGGCTCCCAGTATTTCTCCTCAGGAAAACATTATTGGGAAGTAGATGTATCCAACAAGACTGCCT
GGGTCCTGGGCGTGTACTGTAGAAAACCT---TCTGTGAA------------------G------CTGGCTA-------------------------
GCCGTCTCAGCACTG---------CCCACTT------------------GAAAAC--------
TTGGTCAAGGTACAAACCGGAACATGGCTACTGGGTCATAGGGTTAC---------
GGAAGAAACATGGGTATCGTGCCTTCGACGA---

ATCTCCTACCTTTGATCCAAAGGTTTTGGCCCTTTCTGTGGCCATTCCTCTCCGTCGCATTGGAGTTTTCCTCAGCTT
GGAAGCAGGCACTGTCTCCTTTTTCAACATCACAGACAATGGATCGCTCATCTACCAGTTCCATAAATGTTGCCATC
CTCAGCCCCTTTATCCCTATTTCAATCCTGGGAACTGCCCAGCCCCCTTGACTTTGTGCC------------------------
GCCCCTGA--------------------

>Peromyscus_maniculatus_Trim6

CCAGATCTGAAAAGGATGCTGCGAGTCTTTAGAGAGCTGACAGACGTCCAAAGCTACTGGGTGGACGTGACCCTG
AATCCACAGACGGCTAATTTAAATCTTGTCCTGGCTAAAAACCGGAGGCAGG----TGAGGTTT--------GTA-GGTGC-
-----CAAGCTGTCCGAG------TCTTCCTGTCTGGAAGAA-CATT------------------------------TTGA------
TTGTAGTGTCCTGGGTTCTCAGCACTTCTCCTCAGGAAAATACTACTGGGAGGTGGACGTGACCAAGAAGACGGC
TTGGATCCTGGGTGTATGCAGTAAC---------CCGGTGGA------------ACCCATG------TTCTCTT--------------------------
TCAGCCAGTATCCCA---------GCAAGCA------------------GAGTGC--------
CCACTCCAGGTATCAGCCCCAGAGCGGATACTGGGTGATTGGGTTGC---------
AACATAAGCATGAGTACAGAGCCTATGAGGA---CTCCTCCACCT------------
CCCTGCTCCTCTCCATGACAGTGCCCCCTCGCCGCGTAGGGGTTTTCTTAGACTATGAGGCTGGCACTGTCTCCTTT
TATAATGTCACAAACCATGGCCTGCCCATCTACACCTTCTCTAAGTATTACTTTCCTACTGCCCTTTGTCCGTATTTCA
ATCCCTGCAGCTGTGTGGTCCCCATGACTCTGCGTCGCCCAA------------------GCTCTTGA--------------------

>Peromyscus_maniculatus_Trim12

CCTGACCTGCGAGGCATGCTGCAGGTGCTGCAAGAGCTCATAGAGGCCCAACGCTACTGGGTTCAAGTGACACTG
GTCAAGAACAAC---AATCCAAACATTGCCATTACCGAGGACAAGAGACAAA----TAAGATAT--------GAA-GACCG--
----TCAAACAAGAAAT------TTTGCACCCGGGGGTGAG-AGCT------------------------------GTCA------
TGAAGGTGTCCTGGGCTACCCAGCTATCCAGTCAGGAAAACATTATTGGGAAGTAGATGTGTCTGGAAAAGGTGC
CTGGGTTCTGGGATTAAGTGATGGAAGCTAC---CTCTTCAA------------TCCGATA------
TTTCGTGCAAATGCTGAAA--------------GACACCTAGATCCCTTAT------TCCGCTT------------------
GGGTATTAGTAA--TGATTCACGTTATCAACCTAGATATGGCTTCTGGGTTATAGGGCTGT---------
GGAATAAGTGCGTGTATAATGCCTTTGAGGAGTGTGCTTTCACAGGCAAGCCCAGTGTCTTGACTCTATCTCTGAT
GGTTCCTCCCTGTCGTGTTGGCATTTTCCTTGACTATGCAGCTGGCACCCTCTCGTTTTACAATATTTCCCACCATGG
GACTCTCATCTATAGATTCTGTGCAAATTCCTTTCCTGATAGGGTTTTCCCATATTTTAATCCTATGGGATGTTCAGA
GCCAATGACAGTGTGCTGGTCAG------------------ACTCTTAA--------------------

>Peromyscus_maniculatus_Trim30a

---AATTCTAGAAGAAATTTGCAAATTTC---
-------------TGAGACCT--ATGG------
TTTGGGTGTCCTGGGATACCCAGCTTTGCACTCAGGGAAACATTACTGGGAAGTGGATGTGTCTAGAAATGATGC
CTGGCTCCTGGGATTAAATGATGGAAAAT-----GTGCTCAA------------CCCCA-A------
CTTTGTGCACTGAATGAAAAG------------GGCATCAAAGTCCAATATG-----ATAATTC------------------
TAATGTGAAACA--GCATGTAAATTATCAACCTAAATATGGCTACTGGGTTATAGGGATGA---------
AGAATGGGTCTATCTATAATGCCTTTGAAGAATGTTCTGTCACTCACAATGCCAGTGTCTTGGTCCTCTCTCTGACT
CGTCGTCCCAGTCGTGTTGGAGTTTTCTTGGACAGAGAAGCTTGTACTCTCTCATTTTATGATGTTTCCAACCATGG
AGCTCTCATCTATAGATTCTGTGAACCTTCCTTCCCTGATGAAGTTTATCCATATTTTAATCCTATGTCATGTTTAGA
GCCAATGACAGTATGTGGGCCAC------------------CCTCCTAA--------------------

>Peromyscus_maniculatus_Trim30b

CCAGATCTGAAAGGCATGCTGCAAGTGTTTCAAGGGCTCATGGATGCTCAACAATACTGGGTTCATGTGACCCTGA
ATCAAGTCCAC---AATGAAAACATTGCTGTTAATGAGGACAAACGACAAA----TACAACAT--------CGA-AATAA-----
-TTCTAGAAGAAAT------TTGCAAATT---TCTGAG-ACCT-----------------------------ATGG------
TTTGGGTGTCCTGGGATACCCAGCTTTGCACTCAGGGAAACATTACTGGGAAGTGGATGTGTCTAGAAATGATGC
CTGGCTCCTGGGATTAAATGATGGAAAAT-----GTGCTCAA------------CCCCA-A------
CTTTGTGCACTGAATGAAAAG------------GGCATCAAAGTCCAATATG-----ATAATTC------------------
TAATGTGAAACA--GCATGTAAATTATCAACCTAAATATGGCTACTGGGTTATAGGGATGA---------
AGAATGGGTCTATCTATAATGCCTTTGAAGAATGTTCTGTCACTCACAATGCCAGTGTCTTGGTCCTCTCTCTGACT
CGTCGTCCCAGTCGTGTTGGAGTTTTCTTGGACAGAGAAGCTTGTACTCTCTCATTTTATGATGTTTCCAACCATGG
AGCTCTCATCTATAGATTCTGTGAACCTTCCTTCCCTGATGAAGTTTATCCATATTTTAATCCTATGTCATGTTTAGA
GCCAATGACAGTATGTGGGCCAC------------------CCTCCTAA--------------------

>Peromyscus_maniculatus_Trim30c

CCAGATCTGAAAGGCATGCTGCAAGTGTTTCAAGGGCTCATGGATGCTCAACAATACTGGGTTCATGTGACCCTGA
ATCAAGTCCAC---AATGAAAACATTGCTGTTAATGAGGACAAACGACAAA----TACAACAT--------CGA-AATAA-----
-TTCTAGAAGAAAT------TTGCAAATT---TCTGAG-ACCT-----------------------------ATGG------
TTTGGGTGTCCTGGGATACCCAGCTTTGCACTCAGGGAAACATTACTGGGAAGTGGATGTGTCTAGAAATGATGC
CTGGCTCCTGGGATTAAATGATGGAAAAT-----GTGCTCAA------------CCCCA-A------
CTTTGTGCACTGAATGAAAAG------------GGCATCAAAGTCCAATATG-----ATAATTC------------------
TAATGTGAAACA--GCATGTAAATTATCAACCTAAATATGGCTACTGGGTTATAGGGATGA---------
AGAATGGGTCTATCTATAATGCCTTTGAAGAATGTTCTGTCACTCACAATGCCAGTGTCTTGGTCCTCTCTCTGACT
CGTCGTCCCAGTCGTGTTGGAGTTTTCTTGGACAGAGAAGCTTGTACTCTCTCATTTTATGATGTTTCCAACCATGG
AGCTCTCATCTATAGATTCTGTGAACCTTCCTTCCCTGATGAAGTTTATCCATATTTTAATCCTATGTCATGTTTAGA
GCCAATGACAGTATGTGGGCCAC------------------CCTCCTAA--------------------

>Peromyscus_maniculatus_Trim30d

CCAGATCTGAAAGGCATGCTGCAAGTGTTTCAAGGGCTCATGGATGCTCAACAATACTGGGTTCATGTGACCCTGC
ATGGAACCCAC---GATAAAAACGTTGTCATTGATAAGGACAAAGGAGAAA----TACAACAT--------CGA-AATGT----
--TAACAGAAAAAAT------CCGCAAGTT---TCTGAG-ACCT-----------------------------ATAA------
GTTGGGTGTTCTGGGATATCCAGCTATCTACTCAGGGAAACATTACTGGGAAATAGATGTGTCTAGAAGTGATGC
CTGGCTCCTGGGATTAAATGATGGAAAAC-----GTTCTCAA-------------CCCAAA------
CTTCATGCACCAAATGAAAAG------------GGCATCAAGGTCAAATACG-----ATGATTC------------------
TGATGTGAAAAA--GCATGTAAATTATCAACCTAAATATGGCTACTGGGTTATAGGGATGA---------
AGAATGGGTCTGTATATAATGCCTTTGAAGAATGTTCTGTCACTCACAATGGCAGTGTCTTGGTCCTCTCTTTGATT
CGTCGTCCCAGTCGTGTTGGAGTTTTCCTGGACAGAGAAGCTTGTACTCTCTCATTTTATGATGTTTCCAACCATGG
AGCTCTCATCTATAGATTCTGTGAACCTTCCTTCCCTGATGAAGTTTATCCATATTTTAATCCTATGTCATGTTTAGA
GCCAATGACAGTATGTGGGCCAC------------------CCTCCTAA--------------------

>Peromyscus_maniculatus_Trim34

CCTGATCTGAGAGACATGCTCTGCAAGTTCAGAAAGCTAACAGCTGTCCGTGGCTACTGGGCGGACTTCACATTTA
ATCCAGAAAACCTAAATTTGAATCTTATTCTTTCAGAAGATCACAGACAAG----TGACATCT-------------GTGC------
CCATTTGGCCATT--------------------TAAG-TGTT-----------------------------ATAA------
TAATGGTGTCTTGGGCTCCAAATGTTTCTTCTCAGGAAAACATTACTGGGAAGTGGATGTGTCCAAGAAGAGTGCC
TGGACTCTGGGAGTTTATGCTAGAAAACGTAAAAATTTAAA------------------G------TTTGATG-------------------------

-TTAGACGAGGCAAAA---------ATCAGCC------------------AAATGT--------
TTACCACAGATACAAACCTCAGAATGGCTACTGGGTTATAGGGTTAC---------
AGGATGGATCAAAGTATAGTATCTTTGAGGA---
TTCTTCCAACTGTGACCCTACAGTTCTGACCCCCTTTGTGGCTGTCCCTCTCCATCGGGTTGGGGTTTTCCTTGACTG
TGAAGAGGGCATAGTGTCCTTCTTTAATGTCACAAACCACGGGTCACCCATTTACACATTCTCTCAATGCTGCTTTT
CCAAACCTGTGTATCCATACTTCAATCCTTGGGACTGCCCAGCCCCCATGACCCTGTGTCTTCTGA------------------
ACTCCTGA--------------------

>Rattus_norvegicus_Trim5

CCTGATCTGCAAGGCATGCTGCAAGTGTTACAAGAGGTCACAGAGGCCCAACGCTACTGGGTTCAAGTGACGCTG
GTTGAAAGCAAC---AATCCAAACATTTTCATTACCGCCGACAAAAGACAGA----TACGATAT--------GAA-GACCA----
--CCAAGCAAGACAT------TTTGCCCGTCCGACTGAA-AACT------------------------------GTCA------
TGCAGGTGTCCTGGGATACCCAGCTATCCAATCAGGAAAACACTACTGGGAAGTAGATGTGTCTGGAAAAGGTTC
TTGGGTTCTGGGATTAAGTGATGGAAGCTAC---CTCTTTAA------------TCCAATA------
TTTCGTTCAAATGCAGAAA--------------GACCCCCAAACCCCCCCTATGATTCTCGTTT------------------
GAGTCTTAGTAA--TGATTCACATTATCAACCTAAATATGGCTTCTGGGTTATAGGGCTGT---------
GGGGAAATTCTGTGTATAATGCTTTTGAGGAGTGTACGTTCACAGGCAAGCCCAGTGTGTTGACCCTCTCTCTGAT
GGTCCGACCCTGTCGTGTCGGTATTTTCCTCGACTGTGCAGCTGGCACCCTCTCGTTTTACAATATTTCCAACCATG
GCACTCTTATCTACAGATTCTGTGCAGGTTCCTTTCCTGATAGGGTTTTTCCATATTTTAACCCCATGGGAAGTTCAG
AGCCATTGACAATATGCTGGCCAG------------------ACTCTTAA--------------------

>Rattus_norvegicus_Trim6

CCGGATCTGAAGAAGATGCTGCGAGTCTTTAGAGAGCTGACAGACGTCCAAAGCTACTGGGTGGACGTGACTCTG
AATCCACAGACGGCTAATTTAAATCTTGTCCTGTCGAAAAACCGGAGACAGG----TGAGGTTT--------GTG-GGTGC-
-----CAAGCTGTCGGAG------CCGTCTAGTCTGGAAGAA-CATT------------------------------ATGA------
CTGTAGTGTCCTGGGTTCTCAGCACTTCTCCTCAGGCAAATACTACTGGGAAGTGGATGTGACCAAGAAGACGGC
GTGGATCTTGGGTGTATGCAGTACC---------CCTGTGGA------------ACCCATG------TTCTCCT-------------------------
TCAGCCAGTACTCCA---------GTAAGCA------------------GGGTGC--------
CTACTCTCGGTATCAGCCACAGAGTGGATACTGGGTGATAGGTTTGC---------
AGCGTAAGCACGAGTACAGAGCCTATGAGGA---CTCCTCCCCAT------------
CCCTGCTCCTCTCCATGACGGTGCCACCTCGACGCATAGGGGTTTTCTTAGACTATGAGGCTGGCACGGTCTCCTTT
TATAATGTCACAAACCATGGCTTGCCCATCTACACCTTCTCCAAGTATTACTTCCCTACTGCCCTTTGTCCGTATTTTA
ATCCCTGCAGCTGTGTAGTCCCGATGACTCTGCGGCGCCCAA------------------CTTCCTGA--------------------

>Rattus_norvegicus_Trim30

CCAGATTTGAAAGGCATGCTGCAAGCATTTGAAGGGCTCATGGATATCCAGCGATACTGGGTTCATGTAACTCCAT
GTCAATATAAC---AAGGAAATCGTTTCCATTAACAAAAATAAAGGACAAA----TACAATGT--------GCA-AGTCA------
CTATAGGAGGAAT------CGTCAAGTT---TCTGAG-ACTT------------------------------TCCA------
TTTAGGCGTCGTGGGATATCCAGCTATTCAGTCAGGGAAGCACTACTGGGAAGTAGACGTGTCTACATGTGATGC
CTGGCTCCTTGGATTAAGTGATGGAAAAT-----GTGCTCAA-------------------------CCCCATTTAATGGGTGAAATG---
---------GGCTTCAAAATGAAAC--------TTAATTC------------------TAATTTTAACCA--
AATTGAAATGTATCAGCCTAAATGTGGCTACTGGGTTATAGGGATGA---------
AGGATAGGTCTGTATACAATGCCTTTGATGAGTGCTCTATCACCCACAATCCTAGTGTCTTGGTTCTCTGTCTGCCT
CATGCTCCCAGTCGTGTTGGAGTTTTCGTGGACCGGGAAGCTTGCACTCTCTCATTTTATGATGTTTCTAACTCAGG

AGCTCTCATCTATAGGTTCTATTACCCTTCCTTCCCTCATAGAGTCTTTCCATATTTTAATCCTTTGGAATGTTTGAAG
CCAATGACAGTATGTGGACCAC------------------CCTCTTAA--------------------

>Rattus_norvegicus_Trim30c

CAGGATATGAAAGGCATGCTGCAAGCATATCTAGGGCTCATGGATCTCCGGCGATACTGGGTTCATATGAATCTA
CATGCAAACAAC---CATGCAGTCATTGCCGTTAACAAAGAAAAAAGACAAA----TACAACAT--------ACA-AGTTA----
--CTATAAAAGGAAT------TTACAAATT---TCTGAG-ATCT-----------------------------ATAA------
CTTGAGTGTCCTGGGATACCCAGCTATCCACTCAGGAAAACATTACTGGGAAGTAGATGTGTCTAGAAAAAATGCC
TGGCTCCTGGGAGTAAATGATGGACTGT-----GTGCTCAA-------------CCTCAA------CTTCATTCTATAACTGAAATG-
-----------AGCTTCAGTGCCAAAT--------ATAATTC------------------TTGTGTTGAACA--
ACATGGAAATTATCAGCCGAAATATGGCTACTGGGTTATAGGAATGA---------AGAATAGGTC---------
AGCCTCTGATGAGTGTTTTGTTTCTCATAATTCCAGTGTCTCGACTCTCTCTCTGCCTTGTCCTCCCACTCGTGTTGG
AGTTTTCCTGGACCGGGAAGCTTGCACTCTCTCATTTTATGATGTTTCTAACTTTGGAGGTCTTATCTATAGGTTCCA
TAACCCTTTCTTCCCTGATACACTCTATCCATATTTTAATCCTATGGAATGTTCAGAGCCAATGACAGTATGTAGGCC
AC------------------CTTCTTCAAACTCTGTAGGAAGATAG--

>Rattus_norvegicus_Trim34

CCTGATCTAAGTGGCATGCTACAAAAGTTTAGAGAGCTAACAGCTGTCCGGGCCTACTGGGCGGACTTCACGTTTA
ATCCAGAAAACCTAAATTTGAATCTCATTCTTTCAGAAGATCATAGACAAG----TGACATCT-------------GTGC------
CCATTTGGCCCTT--------------------TAAG-TGTT------------------------------GTAA------
TAATGGTATCTTGGGCTCCAAATGTTTCTTGTCAGGAAAACATTACTGGGAAGTGGATGTGTCTGAAAAGAATGCC
TGGACCCTGGGAGTTTACTCTAGAAAACGT---ACTTTGAA------------------G------TTTGATG--------------------------
TTAGACGATGCAGCAAAA------GTCAGCC------------------AAATGG--------
TTACCACAGATACAAACCTCAGAATGGCTACTGGGTTATAGGGTTAC---------
AGGATGGATCAAAGTATAGTATCTTTGAGGA---
TTCTTCCAACTGTGGCCCTACAGTTCTGAACCCCTTTGTGGCCACCCCTCTCCATCGGATTGGGGTTTTCCTTGACTG
TGAAGAGGGCATAGTGTCCTTCTTCAATGTCACAAACCACGGGTCACTCATTTACAAGTTTACCCAATGCCGCTTTT
CCCAACCTGCCTATCCATACTTCAATCCTTGGGACTGCCCAGCCCCCATGACCCTCTGTCCTCGGA------------------
ACGCCTGA--------------------

Supplementary Data S3

>Jaculus_jaculus_Trim6

---------ATGACGTCGGCACTCCTGGTGGACATCCG---
AGATGAAGTGACATGTCCCATCTGCCTGGAGCTCCTGACAGAACCCCTGAGCATAGACTGTGGCCACAGCTTCTGC
CAGGCCTGCATCATGGCCAATGATGACAATTCACTGGTCAGCCAAGAAGGGAAGAGCAGCTGTCCTGTGTGCCAC
GCCAGCTACCAGACTGTGAACCTCCGGCCGAATCGACACCTCGCCAGCATCGTGAAGAGGCTCAGAGATGTGGCC
CTGGGCCCGGGGCAGCGGCTCGAGGCCATGCTTTGTGCGCTTCATGGG---
GAGAAGCTCCAGCTCTTCTGCAAGGAGGACGGGAAGCTCATTTGCTGGCTTTGTGAGCGTGCTCAGGAGCACCAC
GGTCACCGAACATTCCTCATGGAAGAGGTGGCCCAGGAGTAC---CAGGAGA--------
TGTTCCAGGAGTCTCTGAAGAAGCTAAAGAAAGAGCAGCAGGAAGCTGAGCGGCTAAAAACTGTCATCAGAGAG
AAGAGGTCGTCCTG---------
GAAGAATCAGCTGGAGCCCGAGAGACGCAGGATCCAGACAGAGTTCAGTCGATTGAGAAGCATCCTGGACAGAG
AGGAGCAGCGGGCACTGAAGAAGCTGGAAGAAGAACAGAGGAAGGGGCTGAGCATCATAGAGAAGGCCGAGG
GAGACCTGATCCACCAGAGCCAGTCCCTGACAGAGCTCATCTCTGACCTGGAGCACCGCTGCCAGGGGTCCA------
CGGTGGATCTGCTG---CAGGATGTGA----GTGATGTCACAAAAA----------GGAGTG--
AGTTCTGGACCCTGAGGAAACCCCAACCTCTCCC---CACCAA------------------GCTGAGAAGCATGTT---TCGAGCT--
----

>Microtus_ochrogaster_Trim6

---------ATGACAACCGTGCTTCTGGAGGACATCCG---
AGAGGAAGTCACCTGCCCTATCTGCTTGGAGCTCCTGACAGAACCCCTGAGCATAGACTGTGGTCACAGCTTCTGC
CAGGCCTGCATTACAGGGTACAGTCACAGGTCAGTGCTCAACCAAGAAGGGAAGAGCATCTGTCCTGTGTGCCAG
ACCACCTACCAGCCTGGGAACCTCCGCCCTAACCGACACCTGGCCACCATAGTGAAGAGGCTCAAAGAGATCGTG
TTGGGCCCGGGAAAGCAGCCAGAGGTCATTCTTTGTGCTCTTCATGGA---
GAGAAACTCCAGCTTTTTTGCAAGGAGGATGGGAAGTTAATTTGCTGGCTTTGTGAGCGATCTCCGGAGCACCGT
GGTCACCACACATTCCTCATGGAGGAGGTGGCTCAGGAGTAC---CAGGAAC--------
TGTTCCAGGAGTCTCTGAAGAAGCTGAGGAAGGAGCAGCAGGAAGCTGAGAACCTAAAAGTTCTTATCAAAGAG
AAGAGGGAATCCTG---------
GAAGAGTCAGGTGGAGCCTGAGAGACACCGGATCCAGACCGAATTTAAGCAGCTGCGGAGCATCCTGGACCGAG
AGGAGCAGAGGGAACTGAAGAAACTGGAAATAGAAGAGAGGAAGGGGCTGAGCATCATAGAGAAGGCCGAGG
GGGACCTGATCCACCAGAGCCAGTCACTAAAGGATCTCATCTCCGACCTTGAGCACCGCTGCCAGGGGTCCA------
CAGTGGAGCTGCTG---CAGGATGTGA----GTGATGTCACAAAAA----------GGAGTG--
AGTTCTGGACCCTGAGGAAGCCCCAAACTCTACC---CACCAA------------------GCTCAGAAGTTTGTT---CCGAGCC--
----

>Mus_musculus_Trim6

---------ATGACTTCAACAGTCTTGGTGGACATCCG---
AGATGAAGTAACCTGCCCTATCTGCTTGGAGCTCCTGACAGAACCCCTGAGCATTGATTGTGGCCATAGCTTCTGC
CAGGTCTGCATCATAGGAAACAGTAATAATTCAGTGTTCGGCCAAGGAGGGAGGAGCAGCTGTCCTGTGTGCCG
GACCTCCTATCAGCCTGGGAACCTCCGTCCTAATCGGCACCTGGCCGCCATAGTGAAGAGGCTCAGAGAGGTTGC
GTTGTGCCCTGGAAAACAACTCGAGGTCATTTTTTGTGCGCTTCATGGA---
GAGAAACTCCAGCTCTTTTGCAAGGAGGATGGGAAGTTAATTTGCTGGCTTTGTGAACGATCTCAGGAGCACCGT
GGTCATCACACATTCCTCATGGAGGAGGTGGCCCAGGAGTAC---CAGGACA--------
TGTTCCAGGAGTCTCTGAAGAAATTGAGGAGGGAGCAGCAGGAAGCCGAGAAGCTAAAAGCTCTTATCCAGGAG
AAGAGGGAATCCTG---------

GAAGAGTCAGGTGGAGCCTGAGAAACGCCGGATCCAGACAGAGTTTAAGCAGCTCCGAAGCATCCTGGACAGGG
AGGAGCAGCGGGAACTGAAGAAACTGGAAGTGGAAGAGAGGAAGGGGCTGAGCATCATAGAAAAGGCCGAGG
GTGACCTGATCCACCAGAGCCAGTCACTGAAGGACCTCATCTCAGACCTGGAGCACCGGTGCCAGGGGTCCA------
CCGTAGAACTGCTG---CAGGATGTGG----GTGATGTTACAAAAA----------GGAGTG--
AGTTCTGGACCCTGAGGAAGCCCCAAGCTCTCCC---CACCAA------------------GCTGAAAAGTTTGTT---TCGAGCA--
----

>Rattus_norvegicus_Trim6

---------ATGACTTCAGCAGTCCTGGTGGACATCCG---
AGATGAAGTCACTTGCCCTATCTGCTTGGAGCTCCTGACAGAACCCCTGAGCATAGATTGCGGTCATAGCTTCTGC
CAGGCCTGCATCATAGGAAACAGTGATAATTCAGTGCTCAACCCAGAAGGGAAGAGCAGCTGTCCTGTGTGCCGG
ACCGTCTACCAGCCTGGGAGCCTCCGTCCTAACCGGCACCTGGCCGCCATAGTGAAAAGGCTCAGAGAAGTCGTG
TTAGGCCCTGGAAAACAGCTCGAGGTCATTTTTTGTGCGCTTCATGGA---
GAGAAACTTCAACTTTTTTGCAAGGAGGATGGGAAGTTAATTTGCTGGCTTTGTGAGCGATCTCTGGAGCACCGTG
GTCATCACACATTCCTCATGGAGGAGGTAGCACAGGAGTAC---CAGGACA--------
TGTTCCAGGAGTCTCTGAAGAAGCTGAGGAGGGAGCAGCAGGAAGCCGAGAAGCTAAAAGCTCTTATCCAAGAG
AAGAGGGAATCCTG---------
GAAGAGTCAGGTGGAGCCTGAGAGACACCGGATCCAGACAGAGTTTAATCAGCTCCGAAGCATCCTGGACAGGG
AGGAGCAGCGGGAACTGAAGAAACTGGAAGTGGAAGAGAGGAAGGGGCTGAGCGTCATAGAAAAGGCCGAGG
GTGACCTGATCCACCAGAGCCAGGCACTAAAAGACCTCATCTCAGACCTGGAGCACCGGTGCCGGGGGTCCA------
CAGTGGAACTGTTG---CAGGATGTGG----GTGACGTCACAAAAA----------GGAGTG--
AGTTCTGGACCTTGAAGAAGCCCCAAGCTCTCCC---CACCAA------------------GCTGAAAAGTTTGTT---TCGAGCC--
----

>Cricetulus_griseus_Trim6

---------ATGACTTCAGCAGTTCTGGTGGACATCCG---
AGATGAAGTCACCTGCCCTATCTGCTTGGAGCTACTGACAGAACCCCTTAGTATAGATTGTGGCCACAGCTTCTGC
CAGGACTGCATCACAGGAAGCAGTGACAAGTCAGTGCCCAACCAAGAAGGGAAGAACCGCTGTCCTGTGTGCCG
GACAGCCTACCAGCCCGAGAACCTCCGGCCTAATCGACACCTGGCCATCATAGTGAAGAGGTTCAGAGGGACCGT
GTTGGGCCCAGGGAAGCAGAGAGAGGTCATTCTTTGTGGGCTTCATGGA---
GAGAAACTCCAGCTTTTCTGCAAGGAAGATGGGAAATTAATTTGCTGGCTTTGTGAGCGATCTCAAGAACACCATG
GTCATCACACATTCCTCATGGAGGAGGTGGCCAAGGACTAC---CAGGAGA--------
TGTTCCAGGAGTCTCTGAAGAAGCTGAGGAAGGAGCAGCAGGAAGCCGAGCGGCTAAAAGCTCTTATCCAAGAG
AAGAGGGAGTCCTG---------
GAAGAATCAGGTGGAGCCTGAGAGACACCGGATCCGGACGGAGTTTAAGCACCTGCGGAGCATCCTGGACCGGG
AGGAGCAGAGGGAACTGAGGAAACTGGAAGTAGAAGAGAAGAAGGGTCTGAGCATCATAGAGAAAGCGGAGG
GGGACCTGATCCACCAGAGTCAGTCATTGAAAGATCTCATCTCAGACCTGGAGCACCGGTGCCAGGGGTCCA------
CAGTGGAGCTGCTG---CAGGATGTGA----GCGATGTCACAAAAA----------GGAGTG--
AGTTCTGGACCCTGAGGAAGCCTCAAGCTCTCCC---CACCAA------------------GCTGAAAAGTTTGTT---TCGAGCC--
----

>Peromyscus_maniculatus_Trim6

---------ATGACGTCAGCAGTTCTGGTGGACATCCG---
CGATGAGGTCACCTGCCCTATCTGCTTGGAGCTCTTGACAGAACCCCTGAGCATAGACTGCGGTCACAGCTTCTGC

CAGGCCTGCATCACAGGAAACAGTGACAAGTTAGTGTTCAACCCAGAAGGGAAGAGCAGCTGTCCTGTGTGCCG
GACCGCCTACCAGCCCGGGAACCTCCGGCCTAATCGACACCTGGCTATCATAGTGAAGAGGCTCAGAGAGGTCGT
TTTGGCCCCTGGAAAGCAGCTGGAGGTCATTCTTTGTGCAGTTCATGGA---
GAGAAACTCCAGCTTTTCTGCAAGGAGGATGGGAAGTTAATTTGCTGGCTGTGTGAGCGATCTCAGGAGCACCAT
GGTCATCACACGTTCCTCATGGAGGAGGTGGCCCAGGAGTAC---CAGGAGA--------
TGTTCCAGGAGTCTCTGAAGAAGCTGAGGAAGGAGCAGCAGGAAGCTGAGAGGCTAAAAGCTCTTATCCAAGAG
AAGAGGGAATCCTG---------
GAAGAATCAGGTAGAGCCTGAGAGACACCGGATCCAGACTGAATTTAAGCAGCTGCGGAGCATCCTGGACAGGG
AGGAGCAGAGGGAACTGAAGAAACTGGAAGCGGAAGAGAGGAAGGGGCTGAGCATCATAGAGAAGGCTGAGG
GGGACCTGATCCACCAGAGCCAGTCACTGAAAGATCTCATCTCAGACCTGGAGCACCGGTGCCAGGGGTCTG------
CAGTGGAACTGCTG---CAGGATGTGA----ATGATGTCACAAAAA----------GGAGTG--
AGTTCTGGACCCTGAGGAAGCCCCAAGCTCTTCC---CACCAA------------------GCTGAAAAGTTTGTT---TCGAGCC--
----

>Cavia_porcellus_Trim6

---------ATGACTTCAAGAGTTTTGGTGGACATCCG---
AGAGGAGGTGACCTGTCCCATCTGCCTGGAGCTGCTGACAGAACCATTGAGCATAGACTGTGGCCACAGCTTCTG
CCAAGCCTGCATCGCAGAGAACACTGAGAAATCAGTGATTGGCCAAGAAGAGGAGAGCAGCTGTCCTGTGTGCC
AGACCAGCTACCAACCCCAGCACCTCCGGCCTAATCGGCACTTGGCCAACATAGCAGAGCGGCTCCGAGAGGTAG
TGTTGGGACCAGAAAGACAGACAACGGTCATTCTTTGTGCACAGCATGGA---
GAGAAACTCCAGCTTTTCTGTAAGGAAGATGGGACGCTAATTTGCTGGCTTTGTGAGCGTTCTCAGGAGCACCGC
GGTCACCACACATTCCTCATGGAGGAGGTAGCCCAGGAGTAC---CAGGAAA--------
TGTTCCAAGAGTCGCTGAAAAAGTTGAGGAAGGAAGAGGAGGAAGCTGAGAGACTAAAAGCTGTTATCAGAGAA
AAGAGGGCATCCTG---------
GAAGAATCAGATGGAGCCTGAGAGGCACAGGATCCAGAAAGAGTTTAATCAACTGCGAAACATCCTAGACAAAG
AAGAACAACGGGAACTGAGGAAGCTGGAGGAGGAACAGAGGAAGGGGTTGAGCATTATCGAGAAAGCTGAGG
GTGAGGTGATCCGCCAGAGCCAGTCCCTGAGAGAGCTCATCTCAGATCTGGAGTTCCGATGCCACGGCTCCA------
CAGTGGATCTGCTG---CAGGATGTGA----ATGATCTCACACAAA----------GGAGTG--
AGTTCTGGGCCCTGAGGAAGCCCCAAGTGCTCCC---CACCAA------------------GCTGAGAAGTGCCTT---TCGAGCC-
----

>Dipodomys_ordii_Trim6

---------ATGAGTTCAGCAGTTCTGGTGGACATCCA---
AGAGGAGGTGACTTGCCCCATCTGCCTGGAACTACTCACAGAACCCCTGAGCATCGACTGTGGCCACAGCTTCTGC
AAAGCCTGCATCACAAGGAACAGTGAAGAATCAGTGCGCCACCGAGAAGAGAAGAGCAGCTGTCCTGTGTGCAA
GGCCAGCTACCAGCCTGGGAGCCTCCGGCCTAATCGGCATCTGGCCAACATAGTAAAGAGGCTCAGGGAGGTAG
TGTTGGGCCCAGGGAAGCAGGCAAAGGTCATTCTTTGTGCACAGCATGGT---
GAGAAACTCCAGCTCTTCTGTAAGGAGGACGGGGAGTTGATTTGCTGGCTTTGTGAGCGATCTCAGGAGCACCGT
GGTCACCACACATTCCTCATGGAGGAGGTTGCCCAGGAGTAC---CAGGAAA--------
TGTTCCAGGAGTCTCTGAAGAAGCTGAGAAAAGAGCAGCAAGAAGCTGAGAGGCTAATAGCTGTGATAAGAGAG
AATAGAACATCTTG---------
GAAGAGTCAGATGGAGCCTGAGAGACATAGAATCCAGAAAGAATTTAATAAGCTGCGGAACATTCTGGACAGAG
AGGAGCAGCAGGAACTGAAGAAGCTGGAGGAGGAAGAGAGGAAAGGGTTGGGCATTATGGAGAAAGCAGAGG
GTGAGCTGATCCACCAGAACCAGTCCCTGAGGGAGCTCATCTCAGACCTGGAGCGGCGATGCCAGGGATCCA-----

-CAGTAGATCTGCTA---CAGGACGTGA----GTGATGTCACGAAAA----------GGAATG--
AGTTCTGGACCTTGAGGGAGCCTCTGGCTCTCCC---CACCAA-----------------GCTGAGAAGTAAGTT---CCGAGCC-
----

>NM_006074.5:177-1673Homosapienstripartitemotifcontaining22(TRIM22)transcriptvariant1mRNA

---------ATGGATTTCTCAGTAAAGGTAGACATAGA---
GAAGGAGGTGACCTGCCCCATCTGCCTGGAGCTCCTGACAGAACCTCTGAGCCTAGATTGTGGCCACAGCTTCTGC
CAAGCCTGCATCACTGCAAAGATCAAGGAGTCAGTGATCATCTCAAGAGGGGAAAGCAGCTGTCCTGTGTGTCAG
ACCAGATTCCAGCCTGGGAACCTCCGACCTAATCGGCATCTGGCCAACATAGTTGAGAGAGTCAAAGAGGTCAAG
ATGAGCCCACAGGAGGGGCAGAAGAGAGATGTCTGTGAGCACCATGGA---
AAAAAACTCCAGATCTTCTGTAAGGAGGATGGAAAAGTCATTTGCTGGGTTTGTGAACTGTCTCAGGAACACCAA
GGTCACCAAACATTCCGCATAAACGAGGTGGTCAAGGAATGT---CAGGAAA--------
AGCTGCAGGTAGCCCTGCAGAGGCTGATAAAGGAGGATCAAGAGGCTGAGAAGCTGGAAGATGACATCAGACA
AGAGAGAACCGCCTG---------
GAAGAATTATATCCAGATCGAGAGACAGAAGATTCTGAAAGGGTTCAATGAAATGAGAGTCATCTTGGACAATGA
GGAGCAGAGAGAGCTGCAAAAGCTGGAGGAAGGTGAGGTGAATGTGCTGGATAACCTGGCAGCAGCTACAGAC
CAGCTGGTCCAGCAGAGGCAGGATGCCAGCACGCTCATCTCAGATCTCCAGCGGAGGTTGAGGGGATCGT------
CAGTAGAGATGCTG---CAGGATGTGA----TTGACGTCATGAAAA----------GGAGTG--
AAAGCTGGACATTGAAGAAGCCAAAATCTGTTTC---CAAGAA-----------------ACTAAAGAGTGTATT---CCGAGTA-
----

>Cavia_porcellus_Trim34

---------ATGGCTTCCAAAACCCTGGCAAACCTAGA---
GAAGGAGGTGACCTGCCCCATCTGCCTGGAGCTGCTGACAGAACCCCTGAGTCTAGGCTGTGGCCACAGCTTGTG
CCAAGCCTGCGTCACTCTGCACAACGAG------------
AAGACAGGAAAAGACAGCGGCTGTCCTGTGTGTGGAATCAGGGACCCCCGTGGAAATCTGTGGCCTAATCACCAC
CTGGCTGACATAGTGGAGAAACTCAGGGAGGTGAAGTTGAGCACTGGCATTGGAAAGAAGGGAGATTTCTGTGC
CCTCCATGGA---
GAGAAACTTCAACTCTTCTGTAAGGATGATGGAAAAGTCATTTGCTGGCTCTGCGAGCGTTCTCAGGAGCACCGTG
GTCACCATACATTCCTCATGGAGGAGGTAGCCCAGGAGTGT---CAGGAGA--------
AGCTACAGGCAGCTCTCAAGAGGCTGAGGAAAGAGCAGCAGGAAGCTGAGAAATTGAAAGCTGACATCAGAGA
AGACAAACTTTCTTG---------
GAAGTATCAGATGCAGAATGAGAAACAAAGGATACGAGTAGAATTTAACTGGTTTAGACACATCCTAGAAAATGA
GGAACAGAGGGAGCTGCGAAGATTGGAGAAAGAAGAGAAGGCGAAACTGGATATTTTGGCACAAGCTGAGGCT
GAGCTGGTACAGCAGAGCCAGGTGGTGACAGAGCTAATCTCAGATCTGGAGCGCCGCAGTCTGTGGTCAG------
CAGTGGAGCTGCTG---CAGGACATGA----GTGGAATTATGAAAT----------GGAGTG--
AGATCTGGACACTAAGGAAGCCAAAAACTCCTTC---CAAGAG-----------------ATTGAAGAGAGGGCT---
TTGTGCT---

>Jaculus_jaculus_Trim34

---------ATGGCTGCAGCAGTCCTCATG------------
GAGGAGGCCACATGTCCTATCTGCCTGGAGCTTTTGACAGAGCCCGTGAGTCTGGACTGTGGCCACAGCTCGTGT
CGAGCCTGCCTGTCCACGCA------------
GGGCATCGGCCCCGCCAGGAAAACCAGCTGCGCTGTGTGTGGTACCCGGTGCTCAGCTGAGAACCTGGGGGTGA

ATCGGCCCCTGGCAGGCATAGTAGAGAGACTCAGGGAGGTCAGGATGAGGACTGACACTGAAAAGAAGGAAGA
CTTGTGTGTACGCCATGGG---
GAGAAGCTGCTTCTCTTCTGTGAGGAAGACAGGAAGGTCATCTGCTGGCTCTGTGAGCGTTCTCGGGAGCATCAT
GGCCACCATACCTACCTCCTGGAGGAGGTAGCCACAGAGTGT---CAGGAGA--------
AGCTTCGGAGTGCTCTCGAGAGACTGAGGAGGCAGCAGCGGGAAGCTGAGCAACTGGGAGCTGACATCAGAGA
AGAGAGGATTTCTTG---------
GCAGGTTCAAATACAGACTGAGAGACAAAGGATACAAACAGGATTTAACCGGCTTCGAAGCATCCTAGACAGCGA
GGAGCAGAGAGAGCTGCGGAGACTGGAGGCGGAGGAGCGGAAGATCCTGCACAGCCTGGCAGAGGCCGAGGC
CGAGCTGGCTCAGCAGAGCCGGGTGGTGACCGAGCTCATTGAGGATCTGGAGCGCAGAGGCCGGTGGTCGC-----
-CGAGGGAGCTGCTC---CAGGATATGA----GTGGGATCATGAAAT----------GGAGTG--
AGGTCTGGACACTGAAGAAACCAAAGGCTCTTCC---CAAGAA------------------ACTGAAGACTGTATT---CTGTACC-
----

>Rattus_norvegicus_Trim34

---------ATGGCCTCAACAGATCAGACAGATGTACA---
GAAGGAGGCCACTTGCCCTGTCTGCCACGTGCTTTTGATAAAACCCTTGAATCTAGGCTCTGGCCACCATGTATCCC
AAGCCTGCCTCACCGTGAA---
GAAGACTGCAGTGACCAACCCCAGAGGGAAAAGCCTCTGTCCTATGTGTGGCAATAGATTCTCATTTGAAAATCTA
CAGGCTAATAAGCATCTGGCAGATGTAGTAGAGAGACTCAGGGAAGTCAAGTTGAGCCCTGACATTGGGAAAAA
GGGAGATCTCTGCGTACACCATGGA---
GAGAAACTCCTTCTCTTCTGTCAAGAGGACAAGAAGGTCATTTGCTGGGTTTGTGAGCGTTCTCAGGAGCATCGTG
GTCACCACACCTTCCTCCGGGACGAAGCAGTCAGGGAATGT---CAGGAGA--------
ATCTCCAGGAAGCTCTCAAGAGGCTGAGGAAGGAGCAGGAGAAGGTGGAGACATTTGAAGCTGACATCAAAGAA
GACAGAATTTCCTG---------
GAAGCACCAGATCCAGACCGAGAGACAAAGGATCCAAACAGGCTATAATCAACTTAGAAGAATCCTGGACAAGG
AGGAACAGAACGAGCTGAAAAGACTCAGGGAAGAGGAGCAGATGATACTAGACAGCCTGGCAGGGGCAGAGGC
TGAGCTGGCTCAGCAGAGCCAGTTGGTGGAGGAACTCATCTCGGATCTAGAGCGTCGGTGTGAGTGGTCAG------
ACACAGAGCTGCTG---CAGGATGTAA----GCGGTATCTTGAAAT----------GGAGTC--
AGATCTGGACACTAAAGAAGCCAAAAGCAGTTTC---TAAGAA------------------ACTGAAGATGGTATT---CCAAGCT-
----

>Mus_musculus_Trim34a

---------ATGGCCTCAACAGGTCTGACGAATATACA---
GGAGAAGACCACTTGCCCTGTCTGCCAAGAGCTTTTGACCAAAGCCCTGAGTCTAGGCTGTGGCCACCGTGTATGC
CAAGCCTGTCTCATCACGAAGAAGAAT---
GCAGTGATCAACCCCAGAGAGAAAAGCAGCTGTCCTGTGTGTGGTACTAGATTCTCGTTGGAAAATCTACAGGCT
AATAAACATCTGGCAAATGTAGTAGAGAGACTCGGGGAGGTTAAATTGAAGCCTGACATTGGGACAAAGAGAGA
TCTCTGTGTACACCATGGA---
GAGAAGCTCCTTCTCTTCTGTAAGGAGGACAAGAAGGCCATTTGCTGGGTTTGTGAGCGTTCTCAGGAGCATCGT
GGTCACCACACATTCCTCTGGGAGGAAGCAGTCAGGGAATGT---CAGGAGA--------
ATCTCCAGAAAGCTCTCACGAGGCTGAGGAAGGAGCAGGAGAAGGTGGAGACATTGGAAGCTGACATCAAAGA
AGACAGACTTTCCTG---------
GAAGTGCCAGGTCCAGACTGAGAGACAAAGGATTCAAACAGGCTTTAATCAGCTTAGAAGAATCCTGGACAAGG
AGGAACAGAGAGAGCTGAAAAGACTCAGAGAAGAGGAGCAGATGATACTGGACAGCCTGGCGGGGGCAGAGG

CTGAGCTGGCTCAACAGAGTCAGTTGGTGGAGGAACTTATCTCGGATCTAGAGCTGCGGCGTGAGTGGTCAG-----
-ATACAGAGTTGCTG---CAGGATATGA----GCGGTATTTTGAAAT----------GGAGTC--
AGATCTGGACACTGAAGAAGCCAAAAGCAGTTTC---TAAGAA------------------ACTGAGCATGGTATT---CCAAGCT-
----

>Mus_musculus_Trim34b

---------ATGGCCTCAACAGGCCCGACGAATATACA---
GGAGAAGACCACTTGCCCTGTCTGCCAAGAGCTTTTGACCAAAGCCCTGAGTCTAGGCTGTGGCCACCTTGTATGC
CAAGCCTGTCTCATCTCGAACAAGAAT---
GCAGTGATCAACCCCAGAGGGAAAAGCAGCTGTCCTGTGTGTGGTACTAGATTCTCGTTGGAAAATCTACAGGCT
AATAAACATCTGGCAAATGTAGTAGAGAGACTCGGGGAGGTTAAATTGAAGCCTGACATTGGGACAAAGAGAGA
TCTCTGTGTACACCATGGA---
GAGAAGCTCCTTCTCTTCTGTAAGGAGGACAAGAAGGTCATTTGCTGGGTTTGTGAGCGTTCTCAGGAGCATCGT
GGTCACCACACATTCCTCTGGGAGGAAGCAGTCAGGGAATGT---CAGGAGA--------
ATCTCCAGAAAGCTCTCACGAGGCTGAGGAAGGAGCAGGAGAAGGTGGAGACATTGGAAGCTGACATCAAAGA
AGACAGACTTTCCTG---------
GAAGCGCCAGGTCCAGACTGAGAGACAAAGGATTCAAACAGGCTTTAATCAGCTTAGAAGAATCCTGGACAAGG
AGGAACAGAGAGAGCTGAAAAGACTCAGAGAAGAGGAGCAGATGATACTGGACAGCCTGGCGGGGGCAGAGG
CTGAGCTGGCTCAACAGAGTCAGTTGGTGGAGGAACTTATCTCGGATCTAGAGCTGCGGCGTGAGTGGTCAG-----
-ATACAGAGCTGCTG---CAGGATATGA----GCGGTATTTTGAAAT----------GGAGTC--
AGATCTGGACACTGAAGAAGCCAAAAGCAGTTTC---TAAGAA------------------ACTGAGCATGGTATT---CCAAGCT-
----

>Microtus_ochrogaster_Trim34

---------ATGGCTTCGACAGTTCCGATGAATGCAGA---
GAAGGGGGCCATTTGCCCTGTCTGCCATGAACTTTTGAAAGAACCCACGAGTCTAGGCTGTGGCCACATTGCATGC
AAAGCCTGCATCACCACAAACAAGAAGAATGCAGTGATCAACCCCAGAGGGAGAAGCAGCTGTCCTGTGTGTGGT
ACTAGATTCTCATTTGAAAATCTACAGGTTAATCAACATCTGGCAAACGTAGTAGAGAGACTTAGGGAAGTCAAG
GTGAACCCTGACATTGGGGAAAAAAGAGATCTCTGTATCCACCATGGA---
GAGAAACTCCTTCTCTTCTGTAAGGAAGATAGAAAGATCATTTGCTGGGTTTGTGAGCGTTCTCAGGAGCATCGTG
GTCACCACACTTTCCTCCGGGAGGAAGCAGTCAAGGAATGT---CAGGAGA--------
ATATCCAGAAAGCTCTCGAGAGGCTGAGGAAGGAACAGGAGAAGGCGAAGAAATTGGAAGCTGACATTGAAGA
AGACAGAATTTCCTG---------
GAAGTGCCAGATCCAGACTGAGAGACAAAGGATACAAACAGGTTTTGATCAGCTTAGAAGAATCCTGGACGAGG
AGGAGCAGAGAGAGCTGAAAAGACTTGGGGAAGAGGAGCAGCTGATACTCGACAGCCTGGCGGAGGCAGAAGC
TGAGCTGGCTCAACAGAGCCAGTTGGTCCAGGAACTTATTTCCGGTCTGGAGCTTCGGTGCCAGTGGCCAG------
TGACAGAGCTGCTG---CAGGATATGA----GCGGTACCTTGAAAT----------GGAGTC--
AGATCTGGACGTTGAAGAAGCCAAAAGCGGTTTC---TAGGAA------------------AGTGAAGAAGGTATT---
CCAAGCT--

>Cricetulus_griseus_Trim34

---------ATGGCTTCAGCAGTTCCGATGAATGTAAA---
AGAGGAGACCATTTGCCCTATCTGCCAAGAGCTTTTGAAAGAACCCCTGAGTTTAGGCTGTGGCCACAATGTATGC
CAAGCCTGCATCACCATGAACAAGAAGAATGCAGTGGTCAACAGCAAAGGGAAAAGCAGCTGTCCTGTGTGTGG

TACTAGATTCTTATTTAAAAATCTACAGGTTAATCGGCATCTGGCAGATATAGTAGCAAGACTTAGGGAAGTCAAG
TTGAACCCTGACACTGGGACAAAAAGAGATCTCTGTATACATCACGGA---
GAGAAACTCCTTCTCTTCTGTAAGGAAGATAGGAAGGTCATTTGCCGACTTTGTATGCATTCTCGGGAGCATCTTG
ATCACAACACCTTCCTCCGGGAGGAAGCAGTCAAGGAATAT---CAGGAGA--------
CTCTCCAGAAAGCTCTCAAGAGGCTGAGGGAGGAGCAGGAGAAGGCAGAGAAATTGGAAGCTGACATCAAAGA
AGACAGAATCTCCTG---------
GAAGTGCCAGATCCAGAATGAGAGACAAAGGATACAAACTGGGTTTAATGAGCTTAGGAGAACCCTGAACGAGG
AGGAACAGAGAGAGCTGAAAAGACTGGGGGAGGAGGAGCAGCTGATAGAGGACAGCCTGGCAGAGGCGGAGG
CTGAGCTGGCTCAACAGAGCCAGTTGGTGGAAGAACTTATCTCAGATCTGGAGCGTCGGTGCCAGTGGTCAG-----
-CCATAGAGCTGCTG---CAGGATATGA----GCGGTATCTTGAAAT----------GGAGTC--
AGATCTGGACATTGAAGAAGCCAAAAGTGGTTTC---TAAGAA-----------------ACTGAAGGTGGTATT---
CCAAGCT--

>Peromyscus_maniculatus_Trim34

---------ATGGCTTCAGCAGTTCCAATGCGTGTACA---
GAAGGAGGCCATTTGCCCCGTCTGCCAAGAGCTTTTGAAAGAACCCCTGAGGTTAGGCTGTGGCCACAATGTATG
CCAAGCCTGCATCACCATGAACAGAAAGAATGCAGTGATCAACCCCAGAGGAAAAAGCAGCTGTCCTGTGTGTGG
TACTAGATTCTCATTTGAAAATCTACAGTCCAGTCAGCATCTGGCAGACATAGTAGAGAGACTCAGGGAAGTCGA
GTTGAACCCTGACCTTGAGAAAAAGAGAGATCTCTGTATACACCATGGA---
GAGAAACTACGTCTCTTCTGTAAGGAAGACAGGAAGGTCATTTGCTGGGTTTGTGAGCGTTCTCGGGAGCATCAT
GGTCACCACACCTTCCTCCAGGAGGAAGCAGTCAAGGAGTGT---CAGGAGA--------
ATCTCCAAACAGCTCTCCAGAGGCTGAGGGAGGAGCACAAGAAGGCGGAGAAATTGGAAGCTGACATCAAAGAA
GACAGAATTTCTTG---------
GAAGTGCCAGATCCAGACTGAGAGACAAAGGATACAAACGGGTTTTAATGAGCTCAGAAGAATCCTGAATGAAG
AGGAACAGAGAGAGCTAAAAAGACTTCAGGAAGAGGAGCAGCTGATACTTGCCAGCCTGGCAGAGGCCGAGGC
TGAGCTAGCTCAGCAGAGCCAGTTGGTGGAGGAACTTATCTCAGATCTGGAGCGCCGGTGCCAGTGGTCAA------
CTACAGAGCTGCTA---CAGGATATGA----GCGGTGTCTTGAAAT----------GGAGTC--
AGATCTGGACACTGAAGAAGCCAAAAGCAGTTTC---TAAGAA-----------------ACTGAAGAAGATATT---
CCAAGCT--

>Castor_canadensis_Trim5-ps

TGGGAACCTCCAACCTAATCAGCACCTGGCCAGCATAGTGAAGAGGCTCAGAGAGATAGTGTTGAGCTCAGAAAA
ACAGCCAAAGGTATCTCTTTGTGCACTCCATGGA---
GAGAAACTCCTATTCTTCTGTAAGAAGGATAAGAAGGTCATTTGCTGGCTCTGTGAGTGTTCTCAGGACCACTGTG
GTCATAATGTGTTCCTCGTGGGGGAGGTTGCCGAGGAGTAC---AAG------------TTTCCCAGACCTACCT-------------
-----------------GATAAATAGAGTGCCCCTTTC---------------
TCTTTCTCATTCCTGAAGAATCAAATACAGTTCGAGATATAACATGTTCAGGTTGCGTTTAAGCACCTGAGAGACAT
TCTGGACTCTGAGGAGGCAAAGGAGCAGCAAAAGCTGAAGAAAAAGAAGGAAGATATTTTGAAAAGCCTGGTAG
AGTCAGAAAATGAGCTGGTACAACTGAGTCCATTGGTGAGAGATCTCATCTCAGATCTGGAACATCGACTGCAAG
GCTCAG------CACTGCTACTACTGAATCAAGTTGTGGTTTTTCTAGACTATGAAACT---------
AGGACACTCTCATTTTACAATGTTACAAATCATAGATTTCTCATCTATAAATTTTCTTCATGTTCCTTTTCTGAAAAAT
TTTTCCAACCAAGCTCTTAAACATTTTGA-------------------------------

>Cavia_porcellus_Trim5-ps2

--AATCTTCTTTCTCCCTCC--
--CTTTCCT----------------------TGAGAACTT----CCCAAT----------------------
---------------------------------GATAAGAGTTATTTTGTTTTGCATCTCATGTGACTGAGTGACTCTCTCTTTCTT--------
---ATCCCT--

GAGGAATCAAATGCAGGAGGAGATAGAAAGTGTCAAGGATGCATTTAAAAAAATTAGAGCCACCCTGGAATCTG
AAAAGGAGATGAATCTGCAAAAACTGAAGACAGAGGAGAAAGGTACTCTGAATGGCCTGGCAGAGTCTGAGAG
GGAGCTGACCCAGCAGAGCCAGCTGGTGAGAGAGCTCATCTCAGATGTGCAGCATCGGCTGCAGGGGTCAGTTA
TGTCCATGGAGCTGCAGGA-CATAGTTAAGTACCATGATTTTTCAGAGATATCTGA---
GAACTCTAATCCCTGGACTGTGACCCTTTCTCCAACTGTTCC---TCCAAG----------------TAGTGTCAGAGCTTTT---
CCAGGCT---

>Meriones_unguiculatus_Trim30

ATGGCAACTATGGCCTCATCAATCCTGGCGAAGATAAA---
GGAGGAGGTGACCTGTCCCATCTGTCTGGAGCTCCTGAAGGAACCTGTGAGTGCAGACTGTGGTCACAGCTTCTG
CCGAGCCTGCATCACACTGAACTATGAG---
TCCAGCACAGGCAAAGAAGGGGAGGGCAGCTGCCCTGTGTGCCGAGTTAGTTACCTGTTTGGAAATCTGAGGCCC
AATCGACATGCAGCCAATATAGTGGAGAGTCTCAAAGGGTTTAAGTCCATTCCTGAGGAGGAGCAGAAGGTAAAT
GTCTGTGAACAACATGGA---
GAGAAGCTCCAGCTCTTCTGTGAGAATGACATGACAGCCATCTGCTGGCTTTGTGAGCGATCTCAGGATCATCGTG
GTCACCACACAGCTCTCATTGAAGAGGCTGCTGAAAAGTAC---AAGGGGA--------
AGCTGCAGGCAGCTCTGCAGACACTGATGGCGAATGAAAAAACATGTGATGAATGGCAAGATGACCTCCAACAG
GAGAGAACTTACTG---------
GGAGGATCAAATACAGAGTGATGTAGACAATGTTCAGAAGGAATTTGAAGGACTAAGAGACGTCCTGGCCTCCA
AGGAGAATGAAGAGCTGCGGAAGCTGATGGAAGAGAAGGAAGATATTGTACAAAGGCTGGAGAAGTCTGAAAA
TGAGCTCTCCAGGCAGAGGGAGTCAGTGAGAGACTGCATCTCAGATGTGGAACATCATTTGGAGAGCTCAA------
CCATGGAAATGCTG---CAGAATGTGA----ATTGTGTCCTAAAAA----------GGAGTA--
AAGCTTTGAAACTGCAACAGCCTGAAATGATCC------AGAA-----------------AAAAAAAAGAATATT---
CCATGTGCCA--

>Mus_musculus_Trim30c

------ATGATGGCCTCCTCAGCTCTGGCAATGGCAAA---
GGAAGAGGTGACCTGTCCAATCTGTCTGGAGCTTCTGAAGGAACCTGTGAGCACTGATTGTGGTCACAGCTTCTG
CCAAACCTGTATCATACTGAACTATGTG---
TCCAACAGAAGAATGGATGGGGTAGGCAGCTGCCCTGTGTGCCGGGTTGGTTACCTGTTTGAGAATCTGAGACCT
AATCAAAACATGATCAACATAGTAAAAAGGATCAAAGAGTTGAAGTCTATCCCAGAGGAGAAGGAGAAAGTGTTT
TACTGTGCACAACATGGA---
GAGAAACTCAAGCTCTTCTGTAAGGAGGACAGGATGGCCATCTGCTGGGTTTGTGAGAGATCTCAGAAGCACCGT
GGTCACCAGACAGCTCTCATTGAAGAGGTGGACCAGGAGTAC---AAGGAGA--------
AGCTGCAGGCAGCTCTGAAGAAGCTGATGGAAAATGAAAAAAGATGTGATAAATGGCAGGATGACCTTCAGCAA
CAGAGAGCTGACTG---------
GGAGAATCAAATACAGCGTGATGTGGAATATGTTCAGATGGAGCTTAAAGGACTAAGAGACCTCCTGGACTCCAA
GGAGAATGAGGGGCTGCAGGAGCTGAAGAAAGAGAAGGAAGAGGTTATGGAAAAGCTGGAAGAGTCTGAAAA

TGAGCTAAGGGAGCAGACAGAGTTGGTGAGAGACCTCATCTCAGATGTGGGACATCAGTTGGCGCTCTCAA------
CCATGGAAATGCTG---CAGGGCATGA----ATTCTGTTCTAACAA----------GGATTC--
AGGTCCTGAGACTGAAACAGCCTCAAACTATCCC---CCCAAA------------------AAGAAGAAGAACATA---
CCAAGTTCAGGATCAAGTTCAG--------------------------------

>Mus_musculus_Trim30a

---------ATGGCCTCATCAGTCCTGGAGATGATAAA---
GGAGGAAGTAACCTGTCCTATCTGTTTGGAGCTCCTGAAGGAACCTGTGAGTGCTGATTGTAACCACAGCTTCTGC
AGAGCCTGCATCACACTGAATTATGAG---
TCCAACAGAAACACAGACGGGAAGGGCAACTGCCCTGTATGCCGAGTTCCTTACCCATTTGGGAATCTGAGGCCT
AATCTACATGTGGCCAACATAGTAGAGAGGCTCAAGGGATTCAAGTCCATTCCAGAGGAGGAGCAGAAGGTGAA
TATCTGTGCACAACATGGA---
GAGAAACTCCGGCTCTTCTGTAGGAAGGACATGATGGTCATCTGCTGGCTTTGTGAGCGATCTCAGGAGCACCGT
GGTCACCAAACAGCTCTCATTGAAGAGGTTGACCAAGAATAC---AAGGAGA--------
AGCTGCAGGGAGCTCTGTGGAAGCTGATGAAAAAGGCAAAAATATGTGATGAATGGCAGGATGACCTTCAACTG
CAGAGAGTTGACTG---------
GGAGAACCAAATACAGATCAATGTAGAAAATGTTCAGAGACAGTTTAAAGGACTAAGAGACCTCCTGGACTCCAA
GGAGAATGAGGAGCTGCAGAAGCTGAAGAAAGAGAAGAAAGAGGTTATGGAAAAGCTGGAAGAGTCTGAAAAT
GAGCTGGAGGATCAGACAGAGTTGGTGAGAGACCTCATCTCAGATGTGGAACATCATTTGGAGCTCTCAA------
CCTTAGAAATGCTG---CAGGGTGCAA----ATTGTGTCCTGAGAA----------GGAGTC--
AGTCCTTAAGCCTGCAACAGCCCCAAACTGTCCC---CCAAAA------------------GAGAAAAAGAACATT---
CCAAGCTCCA--

>Mus_musculus_Trim30d

---------ATGGCCTCATCAGTCCTGGAGATGATAAA---
GGAAGAAGTGACCTGCCCAATCTGTCTGGAGCTCTTGAAGGAATCTGTGAGTGCTGACTGTAACCACAGCTTCTGC
AGAGCCTGCATCACACTGCACTATGAG---
TCTAACAGAAACCCAGAAGGGGAGGGCAACTGCCCTGTGTGCCGAGTTCCTTACCTGTTTGAGAATCTGAGGCCT
AATCGACATGTTGCCAACATAGTAGAGAGGCTCAAGGGATTCAAGTCCATCCCAGAGGAGGAGCAGAAGGTTAA
TGCCTGTGTAGAACATGGA---
GAGAAACTCCAGCTCTTCTGTAAGGAGGACATGATGGCCATCTGCTGGCTTTGTAAGCAATCCCAGGAGCACAGT
GGTCACCAAACAGCTCTCATTGAAGAGGTCAACCATGAATAC---AAGGAGA--------
AGCTACAGGCAGCTCTGCAGAAGCTGATGGAAAATGAGAAAAGATGTGATGAATGGCAGGATGACCTTCAGCAA
CAGAGAGCTGACTG---------
GGAGAACCAAATACACAGTGATATAGAAGACATTCAGATAGAGTTTAATGGACTAAGAGACCTCCTAGACTCCAA
GGAGAATGAGAAGCTGCAGGAGCTGAAGAAAGAGAAGGAAGAGGTTATGGAAAAGCTGGAAGAGTCTGAACAT
GAGCTAAGGGAGCAGAGGGAGTTGGTGAGAGACCTCATCTCATATGTTCAGCATCAGTTGGAGCTGTCAG------
TCATGGAAATGAAG---CAGGGTGTAA----ATTATGTCCTGACAA----------GCATTC--
AGACCATGACACTGAAGCAGCCCCAAATAGTTCCCCCAAAAAG------------------AAGAAGAGGAACATC---
CAAAGCTCCA--

>Rattus_norvegicus_Trim30

---------ATGGCCTCATCAGTCCTGGAAATGATAAA---
GGAGGAGGTGACCTGTCCTATCTGTCTGGAGCTCCTGAAGGAACCCGTGAGTACTGATTGCAACCATAGCTTCTGC

AGAGCCTGCATCACAATAAACTATGAG---
TCCAACAGGAACACAGAAGGGGAGGGCAGCTGCCCCGTGTGCCGAGTGTGTTACCTGTTTAGAAACTTAAGGCCT
AATCGACATGTGGCCAACATAGTGGAGAGACTCAAGGGGTTCAAGTCCATCCCAGAGGAAGAACAGAAGGTGAA
TGTCTGTGCACAACATGGA---
GAGAAACTCCAGCTCTTCTGTAAGAAGGACAAGATGGCCATCTGCTGGCTTTGTGAGCGATCTCAAGAGCACCAT
GGTCACAAGACAGCTCTGGTTGAAGAGGTGGACCATGAGTAC---AAGGAGA--------
AGCTGCAGGATGCTCTGCAAAAGCTGATGGAAAAAGAGAAAACATGTGATGAATGGCAGGATGTCATTCAACAG
CAGAGAACTTACTG---------
GGAGAACCAAATACAGAGTGATATAAAAAATGTTCAAATAGAGATTAACGGGCTAAGAGAGCTCCTGGACTCCAA
GGAGAATGAAGAGCTGCAGGAGCTGAAGAAAGAGAAAGAAGATGTTTTTCAAAGGCTGGAAGAGTCTGAAAAT
GAGCTGGTGCAGCAGAGGGAGTCCGTGAGAGACTGCATCTCAGATGTAGAACATCAGTTGGAGCTCTCAA------
CTATGGAAATGCTC---CAGAGTGTGG----AATATGTCCTGAGAA----------GGAGTC--
AGACCTTGAAACTGAAACTGCCGGATATTATCTC------AGA------------------AAGAAGAAGAAAGTT---
CCAAGCTCCA---

>Rattus_norvegicus_Trim30c

---------ATGGCCTCATCTGTCCTGGAGATGATAAA---
GGAGGAGGTGACCTGTCCTCTCTGTCTAGAGCTCCTGAAGGAACCCGTGAGTGCTGATTGCAACCATAGCTTCTGC
AGAGCCTGCATCACTCCGAACTATGAG---
TCCAACAGAAACACAGAAGGGCAGGGCAGCTGCCCTGTGTGCCGAGTTCGTTACCTGTTTAGGAACCTGAGGCCT
AATCAACATGTGGCCAAGATAGTTGAGAGTCTCAAGGGGTTCAAGTCCATTCCAGAGAAGGAGCAAAAGGTGAA
TGTTTGTGCACAACATGGA---
GAGAAACTCCAGCTCTTCTGTACGAAGGAGATGAGAGCCATCTGTTGGGTTTGTGAGAGATCTCAGGACCACCGT
GGTCACCAGACAGCTCTCATTGAAGAGCTGGACCATGAGTAC---AAGAGGA--------
AGCTGCAGGATGCTCTGCAAAAGCTGATGGAAAAAGAGAAAACATGTGATGAATGGCAAGAGAACGTTCAACAG
CAGAGAACTTACTG---------
GGAGACTCAAATACAGAGTGATGTAGAATATATTCAGAAGGAGTTTAAATTACTAAGAGAGCTTCTGGACTCCAA
GGAGAATGAAAAGCTGCAGGAGTTGAAGAAAGAGAAGGAAGATGTTGTGAAAAGGTTGGAAAGGTCTGAAAAT
GAGCTGGTGCAGAGGAGGCAGCGGGTGAGAGACCTCATCTCATATATGCAGCATCAGTTGAAGTTCTCAA------
CCATGGACATGCAG---CAGGATGTGA----ATTCTCTTCTAAGAAGTTATGGTGCGGAGTC--
AGACCTTGAAACTGAAACAGCCGCAAACTATCCC---AAAAAG------------------AAGAAGAAGAATGTT---
CCAAGCTCAG---

>Microtus_ochrogaster_Trim12

---------ATGGCTTCAGAATTCATGATGAATGTCAA---
GGAGGAGGTGACCTGTCCTATCTGTCTGGACCTCATGGTAGAGCCTGTGAGTGCAGATTGTGGTCACAGCTTCTG
CCGAGCCTGCATCACACTGAACTATGAG---
TCGAGCAAAGGCAAAGAGGGTGAGTTCATCTGTCCTGTGTGCCGAGTGAGTTACCTGTTTGGTGATCTGAGGCCT
AATCGGCATGTGGCCAACATAGTGGAGAGGCTCAAGGAGTTCAAGTCCAGCTCAGGGGAGGAGCAGAAGGTGA
ATGTCTGTGCAAAGCATGGA---
GAGAAACTCCAGCTCTTCTGTGAGAAGGACAAGGTGGCCATCTGCTGGCTTTGTGAGCGATCTCAGGAGCACCGA
GGACACCAAACAGCTCTCATTGAAGAGGCGGCCCATGAGTAC---AAGGGGA--------
AGCTCCAGGTGTCTCTGCAAAAGCTGATGTCAGACAAGAAAGAATTAAAGAGCTGGGAAGATGACCTTCAAAAG
GAGAGAACTTCCTG---------

GGAGAATCAAATACAGAAAGATGTTGAAAACGTTCAGACAGAATTTAGAAGACTAAGAGACACCCTGGACTCTGA
AGAGAAGAATGAGCTGCAGAAGCTGACGCAAGAGAGGGAAGACATTCTGAGCAACCTGGCAGAGTCTGAAAGT
AAGCATGCCCAACAGAGCAAGTTGCTAGGAGACCTCATCTCAGATGTGGAACGTCAGCTGCAGTGCTCAG------
CCATGGAGATGCTG---CAGGGAGTGG----ATGACATCATAAAAT----------GGAGTC--
AGGCCTTTTCACTGACGAAGCCCAAAGCCATCCC---CAAGAA------------------ACGAAGACGAGTGTT---
CCGAGCCCCT---

>Mus_musculus_Trim12c

---------ATGGCTTCACAATTCATGAAGAATTTAAA---
GGAGGAAGTGACCTGTCCTCTCTGTCTGAACCTGATGGTGAAACCTGTGAGTGCAGATTGTGGTCACAGCTTCTGC
CAAGGCTGCATCACGGTGTACTTTGAA---
TCCACCAAATGCGATAAGGAAATGTTCAGTTGCCCTGTGTGCCGACTTAGTTACCAGTCTAGCAATCTGAGGCCTA
ATCTACATGTGGCCAACATAGTAGAGAGGCTCAAAGAGTTCAAGCCTAGCCCAGAAGAGGAGCAGAAAGTGTTTA
ACTGTGCAAGACATGGA---
GAGAAACTCCAGCTCTTCTGTAGGAAGGACATGATGGCCATCTGCTGGCTTTGTGAGCGATCTCAGGAGCACCGG
GGCCACAAAACAGCTCTCATTGAAGAGGTGGCCCAGGAGTAC---AAGGAGC--------
AGCTGCAGGTAGTTCTGCAAAGGCTGATGGCAGACAAGAAAGAATTTGAAAACTGGAAAGATGACCTTCAGAAG
GATAGAACTTACTG---------
GGAGAATCAAATACAGAAAGATGTGGAGAATGTTCAGTCAGAGTTTAAACGAATGAGGGATATCATGAACTCTGA
AGAGAAGAAGGAATTGCAGAAGCTGAAGCAAGAGAAGGAAAACATTATCAACAAACTGGCAGAGTCTGAAAATG
AGCATGCTCAGCAGAGCAAGTTGCTAGAAGACTTCATTTCAGATGTGGAACATCAGTTACAGTGCTCAG------
ACATAGAAATACTG---CAGGGTGTGG----AGAACATCATAAAAC----------GGAGTC--
ATACTTTTTCGATGAAGAAGCCCAAAGCCATCGC---CAGGGA------------------ACAAAGAAA---GTT---CCGAGCC---
----

>Rattus_norvegicus_Trim5

---------ATGGCTTCAGAATTCGTGATGAATTTAAA---
AGAGGAGGTGACCTGTCCTATCTGCCTGGACCTGATGGTAGAACCTGTGAGTGGAGATTGTGGTCACAGCTTCTG
CCAAGCCTGCATCACGCTGAACTATGAA---
TCCAGCAAATGCAATCAGGATGAGTTCATTTGCCCTGTGTGCCGAGTTAGTTACCTGTTTAAGAACCTGAGGCCCA
ATCGACATGTGGCCAACATAGTGCAGAGGCTCAAAGAGTTCAAGTCCAGCCCAGAAGAGGAGCCGAAGGTGCTT
TCTTGTGCAAGGCATGGA---
GAGAAACTCCAGCTCTTCTGTAAGAAGGACATGATGCCCATCTGCTGGCTTTGTGAGCGATCTCAGGAGCACCGT
GGACACCAAACAGTTCTCATTGAAGAGGTGGTCCAGGAGTAT---AAGGAGA--------
AGCTGCAGGCAGCTCTGCAAAAGCTGATGGCAGACAAGAAAGAATTTGAGAACTGGAAAGATGACCTTCAAAAG
GAGAGAACTTACTG---------
GCAGAATCAAATACAGAAAGATGTGGAAAATGTCCAGTCAGAGTTTAAAGGAATGAGAGATATCATGGACTCTGA
GGAGAAGAAGGAATTGCAGAAGCTGATGCAAGAGAAGGAAGACATTATGAGCAGCCTGGAAGAGTCTGAAAAT
GAGTATTCTCAGCAGAGTAAGTTGCTAGGAGACCTCATCTTAGATGTGGAACATCAGTTACAATGCTCAG------
CCACGGAAATGCTG---CAGGGTGTAG----AGAACACCATAAAAC----------GGAGTC--
ATACTTTTTCGATGAGGAAGCCCAAAACCATCCC---CAAGGA------------------ACAAAGAAGAGTGTT---CCGAGCC-
----

>Meriones_unguiculatus_Trim12

---------ATGGCTTCAGAATTCATGGTAAATTTAAA---
GGAGGAGGTGACCTGTCCCATCTGTCTGGACCTGATGGTGGAACCCGTGAGTGCAGATTGTGGTCACAGCTTCTG
CCGAGCCTGCATCACACTGAACTATGAG---
TCCAGCAAAAGCAAAGAGGAAGAGTTCATCTGTCCTGTGTGCCGAGTTAGTTACCTGTTTGGGAATCTGAGGCCT
AATCGACATGTGGCCAACATAGTGGAGAGGCTCAAGGGCTTCAAGTCCAGCCCAGAA---
GAGCAGAAGGTGTTTCACTGTGCAAGGCACGGA---
GAGAAACTCCAGCTCTTCTGTGAGAAGGACAAGGTGGCCATCTGCTGGATTTGTGAGCGATCTCAGGAGCACCGT
GGTCACCAAACAGCTCTCATTGAGGAGGTGGCCTGTGAGTAC---AAGGAGA--------
AGCTGCAGGCAGCTCTGCAGAAGCTGATGGCAGACAAGAAAGAATCTGAGAACTGGAAAGATGACCTCCAACAG
GAGAGAACTTATTG---------
GGAGAGTAAAATACAGAAAGATGTAGAAAACGTTCAGACAGAGTTTAAACGAATGGAAGACATCCTGGACTCTG
AGAAGAAGAATGAATTGCAGAAGCTGATGCAAGAAAAGGAAGACATTATTAACAGCCTGGCAGAGTCTGAAAAT
GAGCATGCCCAGCAGAGCAAGTTGCTAGGAGACCTCATCTCAGATGTGGAGCATCAGTTGGGGTGCTCAG------
CCATGGAAATGCTG---CAGGGAGTAG----ACAGCATCATAAACC----------GGAGTC--
ATACTTTCTCGCTGACAAAGCCCAAAACCATCCC---CAAGGA------------------ACAAAGAAGAGTGTT---
CCGAGCCCCC---

>Microtus_ochrogaster_Trim30a

---------ATGGCCTCATCAGTCCTGGGGATGGTCAA---
GGAGGAGGTGACCTGTCCTATCTGTCTGGACCTCATGGTAGAGCCTGTGAGTGCAGATTGTGGTCACAGCTTCTG
CCGAGCCTGCATCACACTGAACTATGAG---
TCCAGCAAAGACAAAGAAGGGGAGGGCATCTGCCCTGTGTGCCGAGTTAGTTACCTGTTTGGGAATCTGAGGCCT
AATTGGCATGTGGCCAACATAGTGGAGAGGCTAACAGGTTTCAAGTCCAGCTCTGGGGAGGAGCAGAAGGTGAA
TGTCTGTGCAAAGCATGGA---
GAGAAACTCCAGCTCTTCTGTGAGAAGGACATGATGATTATCTGCTGGCTTTGTGAGAGATCTCAGGATCACCGTG
GTCACCAAACAGCTCTCATTGAAGAGGTGGCCACTAAATAC---AAG---
--GGAGTGACAG--GGAGTCCATGGTGGTCAGAGTTCACACTG----------

TCTGAGATGTGGAG---------------TCAA------T-----------------------------CTTTTGTCCCTA-------------GGAGTC--
AGACCTTATGTCTGAAACAACCCAAAATGATTCC---AAGAAA------------------ACAGAGTAAGATCTT---
CCGAGCTCCG---

>Microtus_ochrogaster_Trim30b

---------ATGGCCTCATCAGTCCTGGGGATGGTCAA---
GGAGGAGGTGACCTGTCCTATCTGTCTGGACCTCATGGTGGAGCCTGTGAGTGCAGATTGTGGTCACAGCTTCTG
CCGAGCCTGCATCACACTGAACTATGAG---
TCCAACAAACACGAAGAAGGAGAGAGCATCTGCCCTGTATGTCGAGATAGTTACCTGTTTGGGAATCTGAGGCCT
AATTGGCATTTGGCCAACATAGTGGAGAGGCTAACAGGGTTCAAGACAAGCTCAGGGGAGGAGCAGAAGGTGA
ATGTCTGTGCACAACATGGA---
GAGAAACTCCAGCTCTTCTGTGAGAAGGACATGATGGCCATCTGCTGGCTTTGTGAGAGATCTCAGGAGTACTGT
GGTCACCATACAGTTCTCATTGAAGAGGTGGCCACTAAATAC---AAGGCGA--------
TGCTCCAGTCTGCCCTGGAGATGCAGATGGCTAATGAGGAAAGATGTGACCAGTGGGAAGATGACCTTCAAAAG
GAGAGAACTTTCTG---------
GGAGAAAAAAATACAGAGCAATGTAGAAAAGGTCCAGAAGAAGTTTAAAGAAATGCACGAATTCCTGTATTCGG

AGGAGAAGAATAAGCTGCAGAAGCTGAGGCAAAAGGAGGAAGACATTGTCAAAAGACTGGAAAAGTCTGAAAA
TGATCTGGTGAAGCAGAGGGAGTCAGTGAGTGACCTCATCTCAGATCTGGAGCATGGGTTGCAGTGCTCAT------
CCATAGAGATGCTG---CAGGGTGTGA----ATCATGTCCTAACAA----------GGAGTC--
AGACCTTAAGTCTGAAACAGCCCAAAATGATTCC---AAGAAA------------------ACAGAGAAAGATCTT---
CCGAGCTCCG--

>Microtus_ochrogaster_Trim30-ps

---------ATGACCTCATCAGTCCTGGAGATGATCAA---
GGAGGAGGTGACCTGTCCTATCTGCCTGGAGCTTCTGAAAGAACCTGTGAGTGCCGATTGTGACCACATCTTCTGC
CGAGCCTGCATCACTCTGAACTATGAA---
TCCAGAAAAGGCAAAGAAGGGGAGGGCATCTGCCCTGTGTGCCGAGAATGTTACCTGTTTGGGAATCTGAAGCCT
ATTTGACATGTAGCCAACATAGTGGAGAGGCTAAAGGAGTTCAAGTCCAGCCCAGAGGAGGAGCAGAAGGTGAA
TGTCTGTGCA-AACATGGA---
GAGAAACTCCAGCTCTTCTGTGAGAAGGAAATGGTGGCCATCTGCTGGCTTTGTGAGAGATCTCAGGAGCACCGT
GGACACCAAACAGCTCTCATTGAAGAGGTGGCCAATAAGTAT---GAGGGGA--------
TGCTTCAGGCTGCCCTGGAGATGCAGATGGCTAATGAGGAAAGATGTGACCAGTGGGAAGACGACCTTCAAAAG
GAGAAAGTTTCCTG---------
GGAGAACCAAATACAGAGTGAGGTAGAAAATGTTCAGAAGGAGTTTAAAATACTGCGGGAATTCCTGGACTCCA
AGGAGAAAAATGAGCTACAGAAGCTGATGAATGAGAAGGAAGACATCCTAGACATCCTGGAAGGTTTTCAAACT
GAGCTGGAGAAGCAGAGCAAGTCAGTGAGAGAACTGATCTCAGATCTGGAGCATTGGTTGCAGTGCTCAA------
CCATAGAGATGCTG---CAAGGTGTGA----ATTGTGTTTTAACAA----------GGAGTC--
AGAATTTAAGTCTGAAACAGCCCGAAATCACCTG---GAGAAA------------------AGAGAGAAACATCTT---
CCGAGCTCCAGATCTGAAGGGCATGCTGTAAGTGTTTCAAGGGCACTGGGATGC

>Microtus_ochrogaster_Trim30c

---------ATGGCCTCATTAGTACTGGGGATGATCAA---
GGAGGAGGTGACCTGTCCTATCTGCCTGGAGCTTCTGAAAGAACCTGTGAGTGCCGATTGTGACCACAGCTTCTG
CCGAGCCTGCATCACTCTGAACTATGAG---
TCCAGCAAAGGCAAAGAAGGTGAAGGTACCTGCCCTGTGTGCCGAGTTACTTATATGTTTGAGAATCTGAGGCCT
AATCAGCATGTGACCAACATAGTGGAGAGGCTCAAGGAGTTCAAGTCTAGCCCAGAGGAGGAACAGAAGGTCAA
TGTCTGTGCACAACATGGA---
GAGAAACTCCAGCTCTTCTGTGAGAAGGACATGGTGGCCATCTGCTGGCTTTGTGAGAGATCTCAGGAGCACCGT
GGTCATCAAACAGCTCTCATTGAAGAGGTGGCCAATAAGTAC---AAGGGGA--------
TGCTCCAGGCTGCCCTGAAGATGCAGATGGCTAATGAGGAAAGATGTGACCAGTGGGAAGACGACCTTCAAAAG
GAGAAAGTTTCCTG---------
GGAGAACCAAATACAGAGTGAGGTAGAAAACGTTGAGAAGGAGTTTAAAGGACTGTGGGAATTCCTGAAAACTG
AGGAGACTAATGAGCTGCAGAAGCTGATGAAAGAGAAGGAAGACATTCTGGACATCTTGGAAGATTCTCAAAAT
GAGCTGAAGAAGCAGAGGGAGTCAGTGAGAGACCTCATCTCAGATCTGGAGCATAGCTTGCAGTGCTCAA------
CCATGGAGATGCTG---CAGGGTGTGA----TTTGTGTTTTAACAA----------GGAGTC--
AGTACTTAAGACTGAAACAGCCTAAAATGGTCCC---GAGAAG------------------AAAGAGAAAGATCTT---
CAGAGCTCCG--

>Cavia_porcellus_Trim5-ps1

ATGGCTTCATCCATTCTGGAGAATATCAAGGTGGAGGAGATGTCCTGCCCCATCTGCCTAGAGCTCATGACAGAAC
CTGTAAGCACTTACTGTGGCCACAGTTTCTGCAAACCATACATCACTTCAAACTATGAG---
TCCATGGAACATGAACAAGGAGTGAGCCACTGCCCTGTGTGCCAAGTCCCTTACCAGTTTGAAAACCTGAGGCCC
ACTTGCCATGTGGCCAACATAGTGGAGAGGCTCAAGGAGTTGACACTGAGCCCA------------
AAGGCAGATCACTGTGACCTCCATGAG---
GAGAAACTCCTATTATTCTGTAAGGAGGGCAGGAAGCTCATTTGCTGGCTTTGTGAGCATTCTCAGGGACACTGTG
GTCACAATGTGCTTCACCTGGAGCAGGCTAC--AGGAATAT---
CAGGTGACTTCTTCTTCCTACCTCCCTTTCCCTAAGAACTTCACAATGAAAACAGTTATTCTG----
TTTTGCATCTCATGT---GATTGAGGGACTCTCTTTTTCATCCCTGAAGACTCAAATGCAGGAGGAGAGG-
AAAGTGTCAAGGATGCATTTTAAAAAATTAGAACCACCCTGAAAT-TGAGGAGGAGA----------------------------------
----

>Chinchilla_lanigera_Trim5-ps

---------ATGGCTTCGTCAGTCCTGGAGGATGTCAG---
GCAGGAGGTGACCTGCGCCATCTGCCTGGAGCTCATGACAGAACCTGTGAGCACCGACTATGGCCACACTTTCTG
CAAACTCTGCATCAGTTGGATCCATACT---GTCATCGAAGAGGAAAATGAAGTGAGCTGCTGCCCA--
GTGCCGAGTCACTTACCATATTGAAAACTTGAGGCCCAGTCAACATGTGGCCAACATAGTAGAGAGGCTCGGGGA
CGTGACCCTGACCCCA------------CAGGCAGATCAGTGGGACCTCCATGGG---
GAGAAACGCCTGCTGCTCTGTTGGCAAGATGGGAAGGTCATTTGCTGGCTTTGTGAGCATTCTCAGGAGCACCGC
GGACACCACACGCTTCTCATGCAGGAGGCTGAGCAGGTGTAC---CGGGTAA--
--GAGG--
TCTGAGCTGTGGGAAGAAA---
--TCAGA----AAACACC---------------------------ACCTGCTA--G--------------------------
----

>Octodon_degus_Trim5-ps2

---------ATGGCTTCGGAGGTCCTGGAGAATGTCAA---
GGAGGAGGTGACCTGCCCCATCTGTTTGGAGCTCTTGAGAGAACCCGTGAGCACTGACTGTGGCCACTTTTTCTGC
AAACTCTGCATCACCTCAAACTATGAG---
TCCACAGAACATGAAGAAGGTGTGGGCCACTGCCCCGTGTGCCGAGTTACGTACCAGTTTGAAAACCTGAGGCCC
AGTCGACATGTGGCCAACATAGTGGAGAGCCTTGGAAAGTTGCCACTGACCCCA------------
AAGATAGATCTGTGCAGCCTCCATGGG---
GAGAAACTCCTGCTGTTTTGTAAGCAGGATGGGAATGTCATTTGCTGGCTTTGTGAGCGTTCTCAGAAGCACCATG
GTCACTGGACGCTTTTAATGGAGGAGGCTGAAGGGGAGTAT---CGGGTAA------------------
AGTGCTAGAGAAACTGACTGAGGATGACAAAGAGTTTAAGAAGTGGGGAGTTCACATTGAAGAAGAAAGAACTT
CCTGGAAGTTCCTGAAGAAACAAATACAGGAGGAGATACAAAATGTCCAGGATGAATTTACAAAAATGAGAAACA
TCCTGGACTCTGAGGAGAAAGAGCACCTGCAAAAGCTGAAGCAAGAGGAAGAAAATGCTCTCGAGGTCCTGGCC
GAGTCTGAAAAGCAGCTGGCCCTGGACGCCCAGTCGGTGAGGGAGCTCATCTCAGATGTGCAGCGTCGGCTGCA
GGCATCAG------CCATGGCCATGTTG---CAGGTA---------------------------------GGAGTA--
AGTCATTGACTGTGCAGAAACCAAGAATTTGTCC---TAAGAG------------------ACAGAAGATGGTGTT---
CCAAGCTCCTGATCTGAGACAGTATCTCCAATCTCATCTAGGTCA---------

>Octodon_degus_Trim5-ps1

---------ATGGCTTCCTTAGTCCTGGAGTATGTCAA---
GAAGGATGTGACTTGCCCCATCTGCCTGGAGCTCATGACAGAACCTGTGAGTACTGACTGTGGCCACTGTTTCTGC
AAAATCTGCATCACCTTATGCTATAAG---
TTCATGGACCATGGAGAAGGAGTGGGCCACTGTCCAGTGTGCCGAGTCAGGTACCGGTTTGAAAACCTGAGGCCT
GGTCAACATGTGGCCAACCTAGTGGAGAGGCTCAGGAAGCTGTCACTGG--CCAGA----------
AAGGCAGATCTCTGTGACCTCCATGGG---
TATGAACACTTGTTGTTCTGTAAGGAGGATGGGACAGTCATTTGCTGGCTTTGTGAGCATTCTTGGAAGCATCATG
GACACCACACAGTTCTTATGGAGGAGGCTGCACAGGAGTATCCACAGGAGA--------
AGCTCCGTGAAGTGCTAGAGAAGCTGATGTAGGATGAGAAAAAGGTTGAGAAGTGGGAAGTTCACACTGAAGAA
GAAAGAACTTCCTGGAAGTTCTTGAAGAAAAAAATACAGGGGGAGAAAGAAAGTGGTTTGGCTGAGTTTGCAAA
AATGAGAAACATCCTGGACTCTGAGGAGAAACTT---
CTGCAAAAACTGAAGAAAGAGGAAGAAGATGCTCTGAGGGTTCTGGCTCAGTCTGAAAAGCAGCTGGCCCAGGA
GGCCCAGTCTGTGAGGGCGCTCATGTCAGATGTGCAGCACTGGGTGCAGGGGTTAG------AGATTGCCATGTTG---
CAGG---TAA----------------------------GGAGTA--AGTTATTGACTGTGAAGGAACCAAGAACTTGCCC---CAAGAG---
--------------ACAGAATATGGTGTT---CCAAACTCCTGATCTGAAAGGGATTATCCAATTGCATCAAGAGG----------

Supplementary Data S4

>Jaculus_jaculus_Trim5

TTTGATCTGCAAGTAGCGCTGAAAGCATTTCGAGAGCTAACAGAGGCTCGATGCTACAGGGTTAAGGTGACACTG
GATCCAA---GTACGAATCCACATACCATCATTATTGATGACCAAAGAAGAA--------TAAAATA-CAAC-----
CCACAGAGA------------
CAAAATGATACACTTCTTTCCAGGGGTGTTCTGGGCTCACCAGCTATCACATCAGGGAAGCATTACTGGGAGATAG
ATGTGTCTATGAAAAGTGACTGGCTCCTGGGAGTGAGT--------GGTAGAAGATG--------------------CTCCCA-
ACCGACGTTTTCTCAACATACACAACGCACAACTGCA---
ATCTATAAGTCCGCACCGAGCCTATTTCGTGCTTCTGTAAA--T------------
CATCCAGACTATTGGGACTCACCTGAGTACTGGGTCATAGGGATGA---------
AGAGTGGCCACACATACAATGCTTTT--
CAGGGGTTCTCCCCTTTCTCCGGTAATTTCTCCATGACCTTCTTTGTGTCTGTCTCTCCCCATCGAATTGGAGTGTTC
CTGGACTATGGTGCTTCCATGGTCTCCTTTTACAACATTACAAACAATGGAGCACTTATGTGTAGATTCCGTGATTG
CC----------------------------GCTTTC---------CCCCTGAGGTTTTTCC-----------
GTATTTTAATCCTATGAATTGCTCACAG---CCAATGACGGTG----------TGCTGA--

>Jaculus_jaculus_Trim6

CCAGATCTGAAAAGGATGCTGCGAGTCTTTAGAGAGCTGACAGACGTCCAAAGGTATTGGGTGGACGTGACTCTG
AATCCACACACGGCAAATTTAAATCTCGTCCTGTCTAAAAACCGGAGACAGG----TGAGATTCGTG-
GGTGCCAAGCTGTGTGAGCCT------
TCCCGTGCGGAGGAGCATCATGACTGCAGCATCCTGGGCTCTCAGCTCTTCTCTTCGGGGAAGTACTACTGGGAG
GTGGATGTGACGAAGAAGACCGCCTGGATCCTGGGGGTGTGCAGTAACCCGGTGGCCCCGACGCCCT-------------
CTTTCA-GCCAG-----TACGCTAAGGCTGACGTG--------------------------------------CAGGCTGCT--------------------
CCCTCCAGGTACCAGCCACAGAGTGGGTACTGGGTGATCGGGCTAC---------
AGTACAGGCATGAGTACAGGGCTTAT--GAGGA---CTCCGCCACCT------------
CCCTGCTCCTCTCCATGTCGGTGCCCCCTCGCCGGGTCGGGGTTTTCTTAGACTACGAGGCCGGCACTGTCTCCTTT
TATAATGTTACAAACCATGGCTTGCCCATCTACACCTTCTCAAAGTATT-----------------------------ACTTTC---------
CCACTGCCCTGTGCCC-----------GTATTTCAGTCCTTGTAACTGTGTGGTC---CCGATGACCCTG----------
CGTCGCCCA--------AGCCCTTGA-----------------------

>Meriones_unguiculatus_Trim6

CCGGATCTAAAGAAGATGCTGCGAGTCTTTCGAGAGCTGACAGGCGTCCAAAGCTACTGGGTGGACGTGACTCTG
AATCCGCAGACGGCTAACTTAAATCTTGTCCTGTCTAAAAACCGGAGACAGG----TGAGGTTTGTG-
GGTGCCGAGCCGTCCGGGCCC------
TCCTGTCCGGAAGAACATTATGACTGTGGCGTCCTGGGCTCTCAGCACTTCTCCTCAGGAAAGTACTACTGGGAGG
TGGACGTGAGCAAGAAGACAGCTTGGATCCTGGGCGTGTGCAGGGCCCCGGTGGAGCCCGTGT-----------------
CCTC---------ATTTAGCCCCAGCAAGCCGCGG------------GCC--
AGCCCCAGGTACCGGCCCCAGAGCGGGTACTGGGTGATAGGGCTGC---------
GGCATAAGCGCGAGTACAGAGCCCAC--GAGGA---CCCCTCCCCCT------------
CCCTGCTCCTGTCCCTGAGCGTGCCGCCTCGCCGCGTAGGGGTCTTCTTAGACTACGAGGCGGGCACGGTCTCCTT
TTATAACGTCACAAACCACGGCTTACCCATCTACACCTTCTCCAAGTATT-----------------------------ACTTCC---------

CTGCTGCCCTTTGTCC-----------GTACTTTAATCCCTGCAGCTGTGTGGTC---CCGATGACCCTG----------
CGGCGCCCC--------AGCGCG--------------------------

>Mus_musculus_Trim6

CCGGATCTGAGGAAGATGCTAAAAGTCTTTAGAGAGCTGACAGATGTCCAAAGCTATTGGGTGGACGTGACACTG
AATCCACAGACGGCTAATTTAAATCTTGTCCTGTCGAAAAATCGGAGACAGG----TGAGGTTTGTG-
GGTGCCCAGCTGTCCGAGCCA------
TCCAGTCTGGAAGAACATTATGACTGTAGTGTCCTGGGCTCTCAGCACTTCTCCTCAGGAAAATACTACTGGGAAG
TGGACGTGAGCAAGAAGACGGCATGGATCTTGGGTGTATGCAGTACCCCGGTGGATCCCATGTTCT--------------
CTTTCA-GCCAG-----TACTCCAGCAAGCAGGGC------------GCC---
TACTCCAGGTATCAGCCACAGTGTGGATACTGGGTGATTGGCTTGC---------
AGTGTAAGCACGAGTACAGAGCCTAT--GAGGA---TTCCTCCCCGT------------
CCCTGCTCCTCTCCATGACCGTGCCACCTCGACGCATAGGGATTTTCTTAGACTGTGAGGCTGGCACGGTCTCCTTT
TATAATGTCACAAACCACGGCTTGCCCATCTACACCTTCTCGAAGTATT-----------------------------ACTTTC---------
CTTCTGCCCTTTGTCC-----------CTATTTTAATCCCTGCAGCTGTATAGTC---CCGATGACCCTG----------
CGGCGGCCA--------ACTTCCTGA-----------------------

>Rattus_norvegicus_Trim6

CCGGATCTGAAGAAGATGCTGCGAGTCTTTAGAGAGCTGACAGACGTCCAAAGCTACTGGGTGGACGTGACTCTG
AATCCACAGACGGCTAATTTAAATCTTGTCCTGTCGAAAAACCGGAGACAGG----TGAGGTTTGTG-
GGTGCCAAGCTGTCGGAGCCG------
TCTAGTCTGGAAGAACATTATGACTGTAGTGTCCTGGGTTCTCAGCACTTCTCCTCAGGCAAATACTACTGGGAAG
TGGATGTGACCAAGAAGACGGCGTGGATCTTGGGTGTATGCAGTACCCCTGTGGAACCCATGTTCT--------------
CCTTCA-GCCAG-----TACTCCAGTAAGCAGGGT------------GCC---
TACTCTCGGTATCAGCCACAGAGTGGATACTGGGTGATAGGTTTGC---------
AGCGTAAGCACGAGTACAGAGCCTAT--GAGGA---CTCCTCCCCAT------------
CCCTGCTCCTCTCCATGACGGTGCCACCTCGACGCATAGGGGTTTCTTAGACTATGAGGCTGGCACGGTCTCCTTT
TATAATGTCACAAACCATGGCTTGCCCATCTACACCTTCTCCAAGTATT-----------------------------ACTTCC---------
CTACTGCCCTTTGTCC-----------GTATTTTAATCCCTGCAGCTGTGTAGTC---CCGATGACTCTG----------
CGGCGCCCA--------ACTTCCTGA-----------------------

>Microtus_ochrogaster_Trim6

CCGGACCTGGAAAAGATGCTGCGAGTCTTTCGAGAGTTGACAGACGTCCAGAGCTACTGGGTGGATGTGACGCT
GAACCCACAGACGGCCAATTTAAATCTTGTTCTGTCTAAGAACCGGAGACAGA----TGAGGTTTGTA-
GGTGCCAAGCTGTCCGAGTCT------
TCCTGTCTGGAAGAGCATTATGACTGCAGTGTCCTGGGCTCTCAGCACTTCTCCTCGGGAAAATACTACTGGGAAG
TGGACGTGACCAAGAAGACGGCTTGGATCCTGGGTGTATGCAGTAACCCAGTGGAACCCGCATTCT--------------
CTTTCA-GCCAG-----TATCCCAGCAAGCAGAGC------------TCC---
TACTCCAGGTATCAGCCCCGGAGCGGATACTGGGTGATTGGGCTGC---------
AGCATAAACACGAATACAGAGCCTAT--GAGGA---CTCCGCCACTT------------
CCCTGCTGCTGTCCATGGCAGTGCCACCTCGCCGCGTAGGGATTTTCTTAGACTATGAGGCTGGCACTGTCTCCTTC

TATAATGTCACAAACCATGGCTTGCCCATCTACACCTTCTCTAAGTATT----------------------------ACTTTC---------
CCACTGCGCTTTGTCC-----------CTATTTCAATCCCTGCAGTTGTGTGGTC---CCAATGACCTTG----------
CGTCGCCCG--------AGCTCCTGA-----------------------

>Cricetulus_griseus_Trim6

CCGGATCTGAAAAAGATGCTTCGAGTCTTTCGAGAGTTGACAGACGTCCAAAACTACTGGGTGGACGTGACTCTG
AATCCACAGACGGCTAATTTAAATCTTGTCCTATCTAAAAACCGGAGACAGG----TGAGGTTTGTG-
GGTGCCAAGCTGTCCAAGCCT------
TCCTGTCTGGAGGAACATTATGACTGTAGTGTCCTGGGCTCTCAGCACTTCTCCTCAGGAAAATACTACTGGGAGG
TGGATGTGACCAAGAAGACGGCATGGATCCTGGGTGTGTGCAGTAACCAGGTGGAACCCATGTTCT--------------
CTTTCA-GCCAG-----TATCCAGGCAAGCAGAGT------------GCC---
TACTCCAGGTATCAGCCCCAGAGCGGATACTGGGTGATTGGGCTGC---------
AGCATAAGCATGAATACAGAGCCTAT--GAGGA---CTCCTCCACCT------------
CCTTGCTCCTCTCTATGACAGTGCCACCTCGCCGCATAGGGGTTTTCTTAGACTACGAGGCTGGCACTGTATCTTTT
TATAACGTCACAAACCATGGCTTGCCCATCTACACCTTCTCTAAATATT----------------------------ACTTTC---------
CTACCGCTCTTTGTCC-----------ATATTTTAATCCCTGCAGCTGTGTAGTC---CCAATGACTCTG----------
CGTCGACCA--------AGCCCTTGA-----------------------

>Peromyscus_maniculatus_Trim6

CCAGATCTGAAAAGGATGCTGCGAGTCTTTAGAGAGCTGACAGACGTCCAAAGCTACTGGGTGGACGTGACCCTG
AATCCACAGACGGCTAATTTAAATCTTGTCCTGGCTAAAAACCGGAGGCAGG----TGAGGTTTGTA-
GGTGCCAAGCTGTCCGAGTCT------
TCCTGTCTGGAAGAACATTTTGATTGTAGTGTCCTGGGTTCTCAGCACTTCTCCTCAGGAAAATACTACTGGGAGGT
GGACGTGACCAAGAAGACGGCTTGGATCCTGGGTGTATGCAGTAACCCGGTGGAACCCATGTTCT--------------
CTTTCA-GCCAG-----TATCCCAGCAAGCAGAGT------------GCC---
CACTCCAGGTATCAGCCCCAGAGCGGATACTGGGTGATTGGGTTGC---------
AACATAAGCATGAGTACAGAGCCTAT--GAGGA---CTCCTCCACCT------------
CCCTGCTCCTCTCCATGACAGTGCCCCCTCGCCGCGTAGGGGTTTTCTTAGACTATGAGGCTGGCACTGTCTCCTTT
TATAATGTCACAAACCATGGCCTGCCCATCTACACCTTCTCTAAGTATT----------------------------ACTTTC---------
CTACTGCCCTTTGTCC-----------GTATTTCAATCCCTGCAGCTGTGTGGTC---CCCATGACTCTG----------
CGTCGCCCA--------AGCTCTTGA-----------------------

>Meriones_unguiculatus_Trim30

CCAGATCTGAAAGGCATGTTGAAAGCATTTCAAGGGATTATGGATGTCCAGCAATACTGGGTTCGTGTGACTCTGT
CTCCAA---ACAACAGTGCAAA---
TGTCCTTTATGAAAAGGAAAGACAAAAAATTACGATGAGTACAATGATTATAAAGGTGTT---------
TTGCAATTTTCTGAGAGTTATTACTTGGGTGTCCTGGGAGGTCCACCCCTCCAGTCAGGGAAATATTACTGGGAAG
TAGACGTGTCTGGATGTAAAGCCTGGCTCCTTGGATTAAAT--------AATGGACAACG--T-----------------GCTCCA-
CTTCA-GCTTCATCCAGGGCAGCCAGGG------------ATCTTCACATCCCAAT---ATA---ATTCTGTTGATAAACA--A----
--------CGTGTAAATTATCAGCCTAAATATGGCTACTGGGTTATAGGGGTAA---------AGGATAGTTCTTTATA---
TGCCTTT--

GATCAGTGTTATGAGACCAAAAATCCCAGTGTCTTGGCCCTCTCTCAGCGAGGCCCTCTCAGTCGTGTTGGAGTGT
TTCTGGACTACAAGGCTTCCACTCTCTTGTTCTATAACGTTTCTAACTGTGGAGCTCTCATCTACAGATTCTATGACC
GTT---------------------------CCTTCC---------CTCACACACTTTATCC----------
ATATTTTAATTCTATGGGATGTTCAAAG---CCAGTGACCATA----------TGTGAGCCA--------CCCTCGTAA------------
----

>Mus_musculus_Trim30b

---A----
TGCAGCATGCA-AATTCCTATAGAAGGAAT---------
TTGCAAATTTCTGAGATCTATCATTTTGTTGTCCTGGGATATCCAACTATTGGCACAGGGGAACAATACCTGGAAGT
AGACATGTCTAGAAGTGATGCCTGGCTCCTGGGATTAAAT--------CATGGACCACAT------------------GCTGCA-
CCCCA-ACTTTGTTCTATGAATGAAATG------------TTCCCTAATGTCAAAT------TTCATGATACTGATATACAG--------
------CATGAAACTTATCAGTCTAAATATGGCTACTGGATTATAGGGATGA---------AGTATAGGTCTGTATA---
TGCCTTT--GATAAGTGTCCTGTCACCTACAATTC-
AGTGTCTTGGCCCTCTCTCTGTCTGGTCCTCTCAGTCATGTTGGAGTTTTCCTGTCCTGGGAAGCTAGGACTCTCTC
ATTTTATGATGTTTCTACCTATGAAGCTTTCATCTATTGGTTCTATGACCCTT---------------------------CCTTCC-------
--CTGATACGGTCA--------------ATATTTTAA---

>Rattus_norvegicus_Trim30-ps

TTCACATGACTCTACATCAAAAACCAAACAAGGCAAGCATTGTCATTAACAAAGAGAAAAGACAAA----
TACCACACAAA---TTCCTGTAGAAGGAAT---------
TTGCAGATTTCTGAGATCTATCACTTTGGTGTCCTGGGATATGCAACTACCTCCTCAGGGAAACATTACTTGGAAGT
AGACATGTCTAGAAGTGATGCCTGGTTCCTGGGATTAAAT--------GATGGACCACA--T----------------GCTGCA-
CCCCA-ACTTTGTTTAATGAATGAAATG------------TTCTCTAATGTCAAAT------TTGATTCTGATGATAAACA---------
-----GCATGAAACTTATCAGTCTAAATATTGCTCCTTGGTTATAGGGATGA---------ACAATTG------
ATACAGTGTTTCC--
GATGAGTATTCTGTCACCCACCATTCCAGTGTCTTGGCCCTCTCTCTGCCTGGTCTTCCCAGTCATAGTGAAGTTTTC
CTGAATGGGGAAACTTACGCTCTTCAGTTTGATGGCGTTTCTACCTATGAAGCTTCCATCTATA-
ATTCTATGAGGCTCATGGATGTCCAGTGGTACTGGGGAGTCAGACCTTCAGATTGAAACAGCCACAAATGATCCC
AAAAAGAAGAACATTCCAAGCTCTGGATCTGAACAGCACTTCCAAGGAGAATGAGGAGTCAGATGTGGAACATCA
GCTGGAGCTCTCAACCGTGGACATGCTGCAGTTTCTC

>Microtus_ochrogaster_Trim30a

CCGGATCTGAAAGGCATGATGCAAGTGTTTCAAGGGCTCCTGGATGCCCAGCGCCACTGGGTTCACGTGACCTTG
CCTCAAC---TCAACAATAAAAACATTGTCATTAACGTGGACAAAAGACAAA----TACAACATAGA-
AGTGGCTATAGAAGAAAT---------
TTGCAAGTTTCCGATTCCTATGATTTAGGTGTCCTAGGGTATCCAGCTATCTACTCAGGGAAACATTACTGGGAAGT
GGACGTGTCTAGATGTGATGCCTGGATCCTGGGAATAAAT--------GATGGAAGATG--T----------------GCTCAA-
CCCCA-GCTTCGTGCAGTGAATGAAAAG------------GGATTCAGAGTCATAA---AAA---ATTATATTGTTAAACA--G--
----------GATGTAAAATATCAGCCCCAATATGGCTACTGGGTTATAGGAATTA---------
CAAACACGTCTGTATGTAACGTCTTT--

GAGGTGTGCTCTGTTCCTCAAAACGCTAGTGTCTCGTTCCTTTCTCTGACTGGCTCTCCCACTGGTGTTGGAGTTTTC
CTGGACCGAGAAGCTTGCACTCTCTCGTTTTATGATGTTTCCAACCATGGAGCTCTCATCTATAGATTCTATGAACC
GA-----------------------------ACTTCC---------CTAATGCCGTTTATCC-----------
ATATTTTAATCCTATGGAGTCATCAGGG---CCAATGACAGTC----------TGTGGGCCA--------CCCTCTTAA-------------
----

>Microtus_ochrogaster_Trim30b

CCGGATCTGAAAGGCATGATGCAAGTCTTTCAAGGGCTCCGGGATGCCCAGCGCTACTGGGTTCATGTGAACCTG
CTTCAAC---TTAACAATAAAAACATTGTCATTAACGTGGACAAAAGACAAA----TACGACATAGA-
AGTGGCTATAGAAGAAAT---------
TTGCAAGTTTCTGAGTCCTATGATTTAGGTGTCCTAGGGTATCCAGCTATCTACTCAGGGAAACATTACTGGGAAG
TGGACGTGTCTAGATGTGATGCCTGGATCCTGGGAATAAAT--------GATGGAAGATG--T-----------------GCTCAA-
CCCCA-GCTTCATGGAGTGAATCAAAAG------------GGCTTCAGAGTCATAA---AAA---ATTCTGTTGTTAAACA--G---
---------GATGTAAGATATCAGCCCCAATATGGCTACTGGGTTATAGGAATTA---------
CAAACAGGTCTGTATATAATGTCTTT--
GAGGTGTGCTCTGTCCCTCAAAATGCTAGTGTCTCATTCCCTTCTCTGACTGGCTCTCCCACTCGTATTGGAGTTTTC
CTGGACCGAGAAGCTTGTACTCTCTTGTTTTATGATGTTTCCAACCATGGAGCTCCTATCCATAGATTCTGCG-
ACTGT-----------------------------TCTTCC--------TCTGGTGCGCTTTATCC-----------
ATATTTTAACCCTATGGAGTCATCAGAG---CCAATGACAGTC----------TGTGGACCA--------CCCTCCTAA-------------
----

>Microtus_ochrogaster_Trim30d

CCGGATCTGAAGGGCATGCTGCAAGTGTTTCAAGGGCTCTTGGATGCCCAGCACCACTGGGTTCACGTGACCCTG
AATCAAA---CACAAGATAAAAACATTGTCATTAATGAAGACAAAAGACAAA----TACAACATCGA-
AATGGTAATAAAAGAAAT---------
TCTCAGATTTCTAAGACCTACAAATTGGGTGTCCTAGGGTATCCAGCTATTCACTCAGGGAAACATTACTGGGAAG
TAGACGTGTCTAGATGTGATGCCTGGCTCCTGGGAATAAAT--------AATGGAAAATG--T-----------------GCTAAA-
CCCCA-GCTTTCTGCAGCAAATGAAAAG------------AACTTCAATGTCAAAT---ATA---ATTCTAATGTTAAACGACA--
----------CATGCAAATTATCAGCCCAAATGTGGCTACTGGGTTATAGGGATGA---------
TGGACGGGTCTGTATATAATGCCTTT--
GAAGAGTGTTCTGTCACCCACAATGCCAGTGTCTTGCTCCTCTCTCTGACTCGTCCTCCCACTCGTGTTGGAGTTTTC
CTGGACCGAGAAGCTTGCACTCTCTCATTTTATGATGTTTCCAACCATGGAGCTCTCATCTATAGATTCTATGAACC
GA-----------------------------ACTTCC---------CTAATGCAGTTTATCC-----------
ATATTTTAATCCTATGACATGTTCAGAG---CCATTGACAGTC----------TGTGGGCCA--------CCCTCCTAA--------------
----

>Microtus_ochrogaster_Trim30f

CCGGATCTGAAGGGCATGCTGCAAGTGTTTCAAGGGTTCCGGGATGCCCAGCGCCACTGGGTTCACGTGATCCTG
CAACAAC---CCAAAAATAAAAACATTGTCATTAACGTGAACAAAAGACAAA----TACAACATAGA-
AGTGGTTATAGAAGAACT---------
TTGCAAGTTTCTGAGTCCTATGATTTAGGTATCCTGGGGTTTCCAGCTCTGCGCTCAGGGAAACATTACTGGGAAG

TAGACGTGTCTAGATGTGATGCCTGGCTCCTGGGAATAAAT--------GACGGAAGATG--T-----------------GCTCAA-
CCCCA-GTTTAGTACAGTGAATGAAAAG------------GGCTTCAGAGTCAAAT---ATA---ATTCTGATGTTAACCA--A---
---------GATGTAAATTATCAGCCCAAATGTGGCTACTGGGTTATAGGGATGA---------
CGGACAGGTCTGTATATAATGCCTTT--
GAAGAGTGTTCTGTTACCCACAATGCCAGTGTCTTGCTCCTCTCTCCGACTCATCCTCCCACTCGTGTTGGAGTTTTC
CTGGACCGAGAAGCTTGCACTCTCTCATTTTATGATGTTTCCAACCATGGAGCTCTCATCTACAGATTCTATGAACC
GA----------------------------ACTTCC---------CTAATGCAGTTTATCC-----------
ATATTTTAATCCTATGATGTGTTCAAAG---CCATTGACAGTC----------TGTGGGCCA--------CCTTCCTAA--------------
----

>Microtus_ochrogaster_Trim30c

CCGGATCTGAAGGGCACGCTGCAAGTGTTTCAAGGGTTCCGGGATGTCCAGTGCCACTGGGTACACGTGACCCTG
CAACAAC---CCAAAAATAAAAACATTGTCATTAACGTGGACAAAAGACAAA----TACAACATGGA-
AGTGGTCATAGAAGAACT---------
TTGCAAGTTTCTGAGTCCTATGATTTAGGTGTCCTAGGGTATCCAGCCATCCACTCAGGAAAACATTACTGGGAAG
TAGACGTGTCTAGATGTGATGCCTGGCTCCTGGGAATAAAT--------GATGGAAGATG--T-----------------GCTCGA-
CCCCA-GCTTCCTTCAATGAATCAACAG------------GGCTTCAAAGCCAAAT---ATGATCATTCTGATAATAAACA--G-
-----------CATGTAAATTATCAGCCCAAATATGGCTACTGGGTTATAGGGATGA---------
TGGACGGGTCTGTATATAATGCCTTT--
GAAGAGTGTTCTGTCACCCACAATGTCAGTGTCTTGCTCCTCTCTCTGACTTGTTCTCCAACTCGTGTTGGAGTTTTC
CTGGACCGAGAAGCTTGCACTCTCTCATTTTATGATGTTTCCAACCATGGAGCTCTCATCTATAGATTTTCTAAACCT
T----------------------------CCTTCC---------CTAATGTGGTTTATCC-----------
ATATTTTAATCCTATGGGATGTTCAGAG---CCATTGACAGTC----------TGTGGGCCC--------CCCTCCTAA-------------
----

>Microtus_ochrogaster_Trim30e

CCAGATCTGGAAGGCATGCTGCAAATATTTCAAGGGTTCCGGGAAGCCCAGCGCCACTGGGTACATGTGACCCTG
CATCAAC---TCAACAATAAAAACATTGTCATTAACGTGGACAAAAGACAAA----TACAACATAGA-
ACTGGTTATAGAAGAACT---------
TTGCAAGTTTCTGAGTCCTATGATTTAGGTGTCCTAGGGTATCCAGCTATCCACTCAGGGAAACATTACTGGGAAG
TAGACGTGTCTAGATGTGATGCCTGGCTCCTGGGAATAAAT--------GATGGAAAATG--T-----------------GCTCAA-
CCCCA-GCTTCCTTCATTGAATCAACAG------------GGCGTGAAAGCCAAAT---ATA---ATTCTGATGTTAACCA--A----
--------CATGTAAATTATCAGCCCAAATGTGGCTACTGGGTTATAGGGATGA---------
CAAACAGGTCTGTATATGAAGCCTTT--
GAAGAGTGTTCTGTAACCCACAAAGCCCGTATCTTGGTCTTCTCTCTGACTCGTCCTCCCACTCGTGTTGGAGTTTT
CCTGGACTGTGAAGCTTGCACTCTCTCATTTTATGATGTTTCCAACCATGGAGCTCTCATCTATAAATTCTGTGAATC
TT----------------------------CCTTCC---------CTAATGTGGTTTATCC-----------
ATATTTTAATCCTATGGGATGTTCAGAG---CCATTGACAGTC----------AGTGGGCCA--------CCCTCCTAA-------------
----

>Cricetulus_griseus_Trim30a

TCCGACCTGAAAGACATGCTGAAAGTGTTTCATGGGATCATGTATGCCCAGCGCTACTGGGTTCACGTGACCCTGC
CTCAAA---CCTACAATAAAAATGTTGTCATTAACATGGGCAAAAGACAAA----TACAATATCGA-
AAGGATTATAGAAGACAT---------
TTGCAAGATTATGAGACCTATGATTTAGGGGTCCTTGGATATCCAGCTATCTATTCAGGGAAACATTACTGGGAAG
TAGATGTGTCTGGAAGTGATGCCTGGCTCCTGGGATTAAAT--------GATGGGAGATG--T-----------------GCTCAA-
CCCTA-CCTTCATGCAGTGAATGAAATGCATGGCTTCCATGGCTTCAAAATCATGT---ATA---
ATTCTGTTGTTAAACA--T------------CATGTAACTTATCAGCCTAAACATGGCTACTGGGTTATAGGTATGG---------
TAAATACATCTTTATATCATGGCTTT--GAAAAGTGTTCTTTCACCCCAAAT------------
ATCCTCTCTCTGACTCATCTTCCCAGTCGTGTTGGAGTTTTCCTGGACAGAGAAGCTTGCACTCTCTCATTTTATGAT
GTTTCCAATCATGGAGCTCTCATGTATAAATTCTGTGAACTTT-----------------------------CCTTCC---------
CTGATGCAGTTTATCC-----------ATATTTTAATCCTATGGAATCATCAGAG---CCAATGACAGTC----------
TGTGGACCA--------CCCTCCTAA----------------------

>Cricetulus_griseus_Trim30b

TCAGATCTGAAAGGCATGCTGGAAGTGTTTCATGAGCTCATGGATGCCCAACACTACTGGGTTCATGTGACCCTGC
CTCGAG---TCTACAAGAAAAATGTTATCATTAATGTGGACAAAAAACAAA----TACAACATCAA-
AATAAGAATACAAGAAATAGAAGAATGTTTAGTATTTTTGGGACCTATGATTTAGGTGTCCTTGGATATCCAGCTA
TCCACTCAGGGAAACATTACTGGGAAGTAGACGTGTCTAGAAGTGATGCCTGGCTCCTGGGATTAAAT--------
GATGGAAGATG--T-----------------GCTCAA-CCCCA-ACATCATGGATTGAATAAAAAA------------
CCCTTCAAAGTCATGT---ATA---ATTCTGTTGTTAAACA--T------------
TGTGTATATTATCAGCCTAAACATGGCTACTGGGTTATAGGTAAGA---------
AGAATAGGTCTGTATATAATGCCTTT--
GAAAATCGTTCTGTCACCCACAATGCCAGTGTCTTGGTCCTCTCTCTGACTCATCCTCCCAGTCGTGTTGGAGTTTTC
CTGGACAGAGAAGCTTGCACTCTTTCATTTTATGATGTCTCCAACCACGGAGCTCTCATCTATAAATTCTGTGAACC
TT-----------------------------CCTTCC---------CTAATACAGTTTATCC-----------
ATATTTTAATCCTATGGAATCATCAGAG---CCAATGACAGTC----------TGTGGACCA--------CCCTCCTAA-------------
----

>Cricetulus_griseus_Trim30c

CCAGATATGAAAGGCATGCTGCAAGTGTTTCAAGGTCTCATGGATGCCCAACGCTACTGGGTTCATGTGTTCCTGT
GTCAAG---TGCACAATAAAAACATTGTCATTACCGTGGACAAAAGACAAA----TACAACATCGA-
AATGATTATAGAAGAAAT---------
TTGCAAGTTTCTGAGACCTATGATTTAGGTGTCCTTGGATATCCAGCTATCCACTCAGGGAAGCATTACTGGGAAG
TAGATGTGTCTAAAAGTGATGCCTGGATCCTGGGATTAAAT--------GATGGAAGATG--T-----------------GCTCAA-
CCCAA-ACTTCATTCAATGAATAAAAAG------------GGC------ATCAAACAGGATG---ATTCTGATGTTAAGCA--G-----
-------CATATAAATTATCAGCCTAAACATGGCTACTGGGTTATAGGGATGA---------
CAAATAGGTCTGTATATAATGCCTTT--
GTAGAGTGTTCTGTCACCCACAACGCCAGTGTCTTGATCCTCTCTCTGACTCATCCTCCCAGTCGTGTTGGAGTTTTC
CTGGACAGAGAAGCTTGCACTCTCTCATTTTATGATGTCTCCAACCATGGAGCTCTCATCTATAAATTCTATGAACC
AT-----------------------------CCTTCC---------CTAATGCAGTTTATCC-----------

ATATTTTAATCCTATGGAATCATCACAG---CCAATGACAGTC----------TGTGGGCCA--------CCATCCTAA-------------
----

>Cricetulus_griseus_Trim30d

CCAGATGTGAAAGGCATGCTGCAAGTATATCAAGGCCTTATGGATGCCCAGCGCTACTGGGTTCATGTGACCCTG
CGTCAAG---TGCACAATAAAAATGTTGACATCAACATGGAAAAAAGACAAA----TACAATGTCAA-
TACAGTTGTAGAAGAAAT---------
TTGCAAGATTATGAGACCTATGATTTAGGTGTCCTTGGAATTCCAGCTATGCAGTCAGGGAAACATTACTGGGAAG
TAGATGTGTCTAGAAGTGATGCCTGGCTCCTAGGATTAAAT--------GATGGAAGATG--T-----------------GCTCAA-
CCCCA-ACTTCATTCAAAGAATCAAAAG------------GGC------ATCATGC---ATA---GTTCTCATGATGAACA--A--------
----CATGTAAATTTTCAACCTAAATATGGCTACTGGGTTATAGGTAAGA---------
AGAATAAGTCTATATATAATGCCTTT--
GTAGAGTGTTCTGTCACCCACAACGCCAGTGTCTTGGTCCTCTCTCTGACTCGTCCTCCCAGTCGTGTTGGAGTTTT
CCTGGACAGAGAAGCTTGCACTCTCTCATTTTATGATGTTTCCAACCATGGAGCTCTCATCTATAAATTCTATGAAC
CTT-----------------------------CCTTCC---------CTCATACAGTTTATCC-----------
ATATTTTAATCCTATGGAATCATTACAG---CCAATGACAGTC----------TGTAGGCCG--------CCATCCTAA-------------
----

>Cricetulus_griseus_Trim30e

CCAGATGTGAAAGGCATGCTGCAAGTGTTTCAAGGCCTCATGGATGCCCAACGCCACTGGGTTCCTGTAACCCTGC
GTCAAG---GGTACAATAACAACGTTCTCATTGATATGGAAAAAAGACAAA----TACAATATTTA-
AATAATTATGGAAGAAAA---------
TTGCAAGATAATGAGACCTTTGCATTAGGTGTCCTTGGATATCCAGCTTTCCAGTCAGGGAAATATTACTGGGAAG
TAGACGTGTCTAGAAGTGATGCCTGGCTCCTAGGATTAAAT--------GATGGAAGATG--T-----------------GCTCAA-
CCCCA-ACTTCATTCAATGAATCAAATG------------GGC------ATCATGT---ATA---ATTCTCATGATAAACA--A---------
---CATGTAAATTTTCAACCTAAATATGGCTACTGGGTTATAGGTAAGA---------
AGAATAAGTCTGTATATAATGCCTTT--
GTAGAGTGCTCTGTCACCCACAATGCCAGTGTCTTGACCCTCTCTCTGACTCATCCTCCCAATCGTGTTGGAGTTTTC
CTGGACAGAGAAGCTTGCACTCTCTCATTTTATGATGTTTCCAACCATGGAGCTCTCATCTATAAATTCTGTGACCCT
T-----------------------------CCTTCC---------CTAATACAGTTTATCC-----------
ATATTTTAATCCTATGGAAACATCACAG---CCAATGACAGTC----------TGTGGACCA--------CCCTCCTAA-------------
----

>Mus_musculus_Trim30c

CAGGATCTGAATGTCATGCTGCAAGCAATTCAAGGGCTCATGTATGTCCGACGATATTGGGTTCATGTGACTCTCT
ATGCAA---ACAACCATGCAGTAATTGCCATTAACAAAGAAAAAAGACAAA----TACAACATACA-
AGTTACTATAAAAGGAAT---------
CTGCAAATTTCTAAGACCTATAACTTGGGTGTCCTGGGATATCCAGCTATCTGCTCAGGGAAACATTACTGGGAAG
TAGACGTGTCTAGAAAAAAGACCTGGATCCTGGGATTAAAT--------GATGGACTGTG--T-----------------GTTCAA-
CCTCA-ACTTCATTCTATAAGTGAAATG------------GGCTTCAAAGTCAAAT---ATA---ATTCTAGTGTGAAACA--A----
--------TGTGGTAATTATCAGCATAAATATGGCTACTGGGTTATAGGGATGA---------

AGAATTGGTCTGTATACAATGCCTCT--
GATGAGTGTTCTGTTACCCACAATTCCAGTGTTTTGGCCCTCTCTCTGTCTGGTCCTCCCAGTCGTGTTGGAGTTTTC
CTGGACCGGGAAGCTTGTACTCTCTCATTTTATGATGTTTCTAACTTTGGAGCTCTCATCTATAGGTTCTATGAACCT
T---------------------------TCTTCC---------CTCATACAGTCTATCC-----------
ATATTTTAATCCTATGGAATGTTCAGAG---CCAATGACAGTA----------TGTGGGCCA--------
TCCTCATCAATCTCTGTGGAAACACAGTC---

>Rattus_norvegicus_Trim30c

CAGGATATGAAAGGCATGCTGCAAGCATATCTAGGGCTCATGGATCTCCGGCGATACTGGGTTCATATGAATCTA
CATGCAA---ACAACCATGCAGTCATTGCCGTTAACAAAGAAAAAAGACAAA----TACAACATACA-
AGTTACTATAAAAGGAAT---------
TTACAAATTTCTGAGATCTATAACTTGAGTGTCCTGGGATACCCAGCTATCCACTCAGGAAAACATTACTGGGAAG
TAGATGTGTCTAGAAAAAATGCCTGGCTCCTGGGAGTAAAT--------GATGGACTGTG--T----------------GCTCAA-
CCTCA-ACTTCATTCTATAACTGAAATG------------AGCTTCAGTGCCAAAT---ATA---ATTCTTGTGTTGAACA--A-----
-------CATGGAAATTATCAGCCGAAATATGGCTACTGGGTTATAGGAATGA---------AGAATAGGTC---------
AGCCTCT--
GATGAGTGTTTTGTTTCTCATAATTCCAGTGTCTCGACTCTCTCTCTGCCTTGTCCTCCCACTCGTGTTGGAGTTTTCC
TGGACCGGGAAGCTTGCACTCTCTCATTTTATGATGTTTCTAACTTTGGAGGTCTTATCTATAGGTTCCATAACCCTT
----------------------------TCTTCC---------CTGATACACTCTATCC-----------ATATTTTAATCCTATGGAATGTTCAGAG-
--CCAATGACAGTA----------TGTAGGCCA--------CCTTCTTCAAACTCTGTAGGAAGATAG-----

>Rattus_norvegicus_Trim30

CCAGATTTGAAAGGCATGCTGCAAGCATTTGAAGGGCTCATGGATATCCAGCGATACTGGGTTCATGTAACTCCAT
GTCAAT---ATAACAAGGAAATCGTTTCCATTAACAAAAATAAAGGACAAA----TACAATGTGCA-
AGTCACTATAGGAGGAAT---------
CGTCAAGTTTCTGAGACTTTCCATTTAGGCGTCGTGGGATATCCAGCTATTCAGTCAGGGAAGCACTACTGGGAAG
TAGACGTGTCTACATGTGATGCCTGGCTCCTTGGATTAAGT--------GATGGAAAATG--T----------------GCTCAA-
CCC-------CATTTAATGGGTGAAATG------------GGCTTCAAAATGAAAC---TTA---ATTCTAATTTTAACCA--A---------
---ATTGAAATGTATCAGCCTAAATGTGGCTACTGGGTTATAGGGATGA---------
AGGATAGGTCTGTATACAATGCCTTT--
GATGAGTGCTCTATCACCCACAATCCTAGTGTCTTGGTTCTCTGTCTGCCTCATGCTCCCAGTCGTGTTGGAGTTTTC
GTGGACCGGGAAGCTTGCACTCTCTCATTTTATGATGTTTCTAACTCAGGAGCTCTCATCTATAGGTTCTATTACCCT
T---------------------------CCTTCC---------CTCATAGAGTCTTTCC-----------
ATATTTTAATCCTTTGGAATGTTTGAAG---CCAATGACAGTA----------TGTGGACCA--------CCCTCTTAA-------------
----

>Mus_musculus_Trim30e-ps1

AGCTCCAGATCTGAAAAGCATGCTGCAAGTGTATCAAGGGCTCATGGATTTCCAGAAACACTGGGTTCATGTGACT
CTACAAGAAA---ACAACCAGGAAAGCATTG----------AAACAAAAGACAAA----TAGGACATATG-
AGTAACTATAGGAAGAAT---------
ATTCAAATTTCTGAGACCTACCATTTGAGTGTACTGGGATACCCAGCTATTTCCTCAGGGAAGCATTACTGGGAAG
TTGACGTGTCTAGAAGTGATGCCTGGCTCCTCGGATTAAAT--------GATGGAAAGTG--T----------------GCTCAG-

CCTCA-ATTTCATTCAATGAATGAAATA------------AGCTTCAAAATGCAAC---ATA---ATTCTAATGATAAACA--
AAATCATCAGCCTAATGTAATGTATCAGCCTAAATATGGCTACTGGGTTATAGGGATGA---------
GGTATAAGGTTGTATACAAGGCCTTT--
GATGAGTGTTCTATCACCCACGATTCCAGTGTCTTAGCCCTCTCTCTGTCTGGTCGTCCCAGTCTCGGCGGAGTTTT
CCTGGATCAGGAAGCTTGCACTCTCTCATTTTATGATGTTTCTAACTGTGGAGCTCTCATCTACAGGTTCTATGACCC
TT----------------------------TCTTCC---------CTCCTATACTCTATCC-----------
ATATTTTAATCCTATGAATTGTACAGGT---CCTATGATTATA----------TGCGGGCCA--------
CCCTCCTAAACCCTCACCTATATCTATACAGT

>Mus_musculus_Trim30a

CCAGATCTGAAAGGCATGCTGCAAGTGTATCAAGGACTCATGGATATCCAGCAATACTGGGTTCATATGACTCTAC
ATGCAA---GGAACAATGCAGTCATTGCCATTAACAAAGAAAAAAGACAAA----TACAGTATAGA-
AGTTACAATACG---------------
GTTCCAGTTTCTGAGATCTACCATTTGGGTGTCCTGGGATATCCAGCTCTTTCCTCAGGGAAGCATTACTGGGAAGT
AGACATATCTAGAAGTGATGCCTGGCTCCTCGGATTAAAT--------GACGGAAAGTG--T-----------------GCTCAA-
CCCCA-ACTTCACTCAAAGGAAGAAATG------------GGCATCAAAAAAAACC---TTC---ATTCTCAGATCAAACA--A---
---------AATGTATTGTTTCAGCCTAAATGTGGCTACTGGGTTATAGGGATGA---------
AGAATCCGTCTGTATACAAGGCCTTT--
GATGAGTGTTCTATCACCCACAATTCCAGTATCCTGGTCATCTCTCTGCCTGATCGTCCCAGTCGTGTCGGAGTTTT
CCTGGATCGGAAAGCTGGCACTCTCTCATTTTATGATGTTTCTAACTGCGGTGCTCTCATCTATAGGTTCTATGACC
CTG----------------------------CCTTCC---------CTGTTGAAGTCTATCC-----------
ATATTTTAATCCTATGAAATGTTCAGAG---CCAATGACTATA----------TGCGGGCCA--------CCCTCCTAA-------------
----

>Mus_musculus_Trim30d

CCACATTTGAAAGGCATGCTACAGTCATTTGAAGGGCTCATGGATGTTCAGCAATACTGGGTTCATATGACTCTAC
ATGCAA---GGAACAATGCAGTCATTGCCATTAACAAAGAAAAAAGACAAA----TACAGTATAGA-
AGTTACAATACG---------------
GTTCCAGTTTCTGAGATCTACCATTTGGGTGTCCTGGGATATCCAGCTCTTTCCTCAGGGAAGCATTACTGGGAAGT
AGACATATCTAGAAGTGATGCCTGGCTCCTCGGATTAAAT--------GACGGAAAGTG--T-----------------GCTCAA-
CCCCA-ACTTCACTCAAAGGAAGAAATG------------GGCATCAAAAAAAAAT---ATC---ATTCTCATATTAAACA--A---
---------AATGTAACGTTTCAGCCTAAATGTGGCTACTGGGTTATAGGGATGA---------
AGAATTCATCTGTATACAATGCCTTT--
GATGAGTGTTCTATCACCCACAATTCCAGTGTCTTGGCCCTCTCTCTGCCTGATCGTCCCAGTCGTGTAGGAGTTTT
CCTGGACCAGGAAGTTTGCACTCTCTCATTTTATGATGTTTCTAACTCTGGAGCTCTCATCTATAGATTCTATGACCC
TT----------------------------CCTTCC---------CTGTTGAAGTCTATCC-----------
ATATTTTAATCCTATGGAATGTTCAGAG---CCAATGACAGTA----------TGCGGACCA--------CCATCCTAA-------------
----

>Marmota_marmota_Trim5a

CCTGATCTGAAGGGGATGCTGCAGGTGTCTCAAAAGCTGACAGACGTTCAACGCTACTGGGTTCACCTGACACTG

TCTCCAA---GCAACAATCCAAATATTGTCATTTCTGAGGACCAGAGACAAT----TAAGATATGTA-CCCCAATAC------
----
TGGCGCAAGCGTGGGAATTATCATGAAGGTGTTCTGGGCTACCCACCTATCACATCAGGAAAACATTACTGGATG
GTAGATGTATCCAGGAAGGAGGCCTGGTATCTGGGATTGTGT--------GATAGAAGTTA--T----------------
TTTCAATCCTCAAATTTCCAAGGACGGAGTAAAAA---
-TTATCAACCTAGATGTGGCTATTGGGTTATAGGGCTTCACACATTTGAATATAATGTTGAATATAATGCTTTT--
GGGGAGGATGCTG---
CCCGTGATCCTTTGACCTTGGTCCTCTCTGTGACTGTTCCTCCACAACGTATCGGGGTTTTCTTAGATTATGAGGCTC
GTGAACTTTCATTTTACAATGTTACAAACCATGGGTTTCTTATCTATAAATTCTCCAGATGTT--------------------------
--CCTTTC---------CTAAGGAAGTTTTTCC-----------TTATTTCAATCCTGGGACATGTCCAGAG---CCCATGACACTA---
-------CACTGGCCA--------AGCTCTTGA----------------------

>Marmota_marmota_Trim5b

----TCTAATTT--------TTCTACA------------------------------------GTTCACCTGACACTGTCTCCAA---
GCAACAATCCAAATATTGTCATTTCTAAGGACCAGAGACAAT----TAAGATATGTA-ACCCAATAC-----------------
TGGCACAAGCGTGGGAATTATCATGAAGGTGTTCTGGGCTACCCACGTATCACATCAGGAAAACATTACTGGATG
GTAGATGTGTCCAAGAAAAGTGCCTGGTCTCTGGGTTTGTGT--------GATGGAAAATA--T----------------TTCGAA--
--TGTGCATCTGTCCCTGGACAAAGTA--
AAAATAATCAACCTACATGTGGCTACTGGGTTATAGGTCTTC------------
ACAGATTTCAATATAATGCTTTTTGGAAGAAAGGT-----
GCCCATTATCCTTTGACCTTGGTCCTCTCTGTGACTGTTCCTCCACAACGTATTGGGGTTTTCCTAGATTATGAGGCT
CGTGAACTTTCATTTTACAATGTTACAAACCATGGGTTTCTTATCTATAAATTCTCCAGATGTT-------------------------
---CCTTTC---------CTAAGGAAGTTTTTCC-----------TTATTTCAATCCTGGGACATGTCCAGAG---CCCATGACACTA--
--------CACTGGCCA--------AGCTCTTGA----------------------

>Meriones_unguiculatus_Trim12

CCCGATCTGCAAGGCATGCTGCAAGTGTTGCAAGAGGTCACAGAGGCCCAGCGCTACTGGGTTCGAGTGACACTG
GTTGAAA---ACAACCATCCAAAAATTGCCATTACTGTGGACAAGAAACAAA----TACGATATGAA-
GACCATCAAACAAGAAATTCA------
AAAGCTAGGGATGAAAGCTGTCATGAAGGCGTCATGGGACACCCAGCTCTCCAATCAGGAAAGCATTACTGGGA
AGTAGATGTGTCTGGAAAAAGTGCCTGGGTCCTGGGATTATGT--------GATGGAAGTTACCT----------------
CTTCAA-TCCCATATTTCTCTCA-----------------------CACCCAAGTCCATTA------TTTCGTTTGGGTCTTAGTAA--T----
--------GATTTACACTATCAACCTAAATATGGGTTCTGGGTTATAGGGCTGC---------
AGAAAAAGTACGTGTATAATGTTTTT--
GAGGAGTGTTCCCTCACAGGCAAGCCCAGCACCTTGACACTCTCTCTCATGGTTCCTCCCTGTCGTGTTGGCGTTTT
CCTTGACTATGCAGCTGGCACGCTCTCGTTTTACAATATTTCCCACCATGGGACTCTCATCTACAGATTCTGTTCAGT
TT---------------------------CCTTTC---------CTGACAGGGTTTTTCC-----------
ATATTTTAATCCTATGAGGTGTTCAGAG---CCAATGACAGTA----------TGCTGGCCA--------GACTCTTAG------------
----

>Microtus_ochrogaster_Trim12

CCTGATCTACAAGGCATGCTGCAAGTGCTTCAAGAGATCACAGAGGCCCAGCGCTACTGGGTTCAAGTGACGCTG

GTTGAGA---ACAACAATCCAAACACTGCCATTTCTGCAGACAAAAGACAAA----TAAGATATGAA-GAA---
CAAACAAGAAATTTT------
GCATCTGGGTGTGAGAACTCTCATGATGGTGCCCTGGGCTACCCATCTATCCAATCAGGAAAGCATTATTGGGAA
GTAGATGTGTCTGGAAAAGGTGCCTGGGTTCTGGGATTAAGT--------GATGGAAGCTACCT-----------------
CTTCAA-TCCGATATTTCATTCAAATGCTGAAAGC------------CGCCTAAATCCCTTA------
TTTCGTTTGGGTATTAGCAA--T------------AATTCGCATTATCAACCTAAATATGGCTTCTGGGTTATAGGGCTGT-----
----GGAACAAGTGCGTGTATAATGCTTTT--
GAGGAGTGTGCCTTCACAGGCAAGCCCAGTGTCTTGACCCTCTCGCTGATGGTTCCTCCCTGTCGAGTGGGCGTTT
TCCTTGACCATGCAGCCGGCACTCTCTCGTTTTACAATATTTCCCACCATGGGACCCTTATCTACAGATTCTGTGCAA
ATT-----------------------------CCTTTC---------CTGATAAAGTTTTTCC-----------
ATATTTTAATCCTAGGGGATGTTCAGTG---CCCATGACAGTA----------TGCTGGTCA--------GACTCGTAA------------
----

>Cricetulus_griseus_Trim12

CCTGATCTGCAAGGCATGCTGCAAGTGCTGCAAGAGCTCATAGAAGCCCAACGTTACTGGGTTCAAGTGACATTG
ATTGAAA---ACAACAATCCAAACATTGTCATTACTGCGGACAAGAGACAAA----TAAGATATGAA-
GACCATCAAACAAGAAATGTT------
GCATCTGGGGGTCAGAACTGTCGTGAAGGTGTCCTGGGCTACCCTGCTATCCAATCAGGAAAACATTATTGGGAA
ATGGATGTGTCTGGAAAAGGTGCCTGGGTTCTGGGATTAAGT--------GATGGAAGCTACCT-----------------
CTTCAA-TCCGATATTTCATTCAAATGCTGAAAGA------------TGCCTAAATCCTTTA------
TTTCGTTTGGGTATCAGCAA--T------------GACTCACATTATCAACCTAAATATGGCTTCTGGGTCATAGGGTTGT----
-----GGAACAAGTTTGTGTATAATGCTTTT--
GAGGAGTGTACTTTCACAGGCAAGCCCAGGGTCTTGACCCTCTCGCTGATGGTTCCTCCCTGTCGCGTTGGCGTTT
TCCTTGACTATGCAGCTGGCACGCTCTCGTTTTACAATATTTCCCACCATGGGACTCTCATCTATAGATTCTGTGCAA
ATT-----------------------------CCTTTC---------CTGATAGGGTTTTTCC-----------
ATATTTTAATCCTATGGGATGTTCAGAG---CCAATGACAGTA----------TGCTGGCCA--------GACTCTTAA-------------
----

>Peromyscus_maniculatus_Trim12

CCTGACCTGCGAGGCATGCTGCAGGTGCTGCAAGAGCTCATAGAGGCCCAACGCTACTGGGTTCAAGTGACACTG
GTCAAGA---ACAACAATCCAAACATTGCCATTACCGAGGACAAGAGACAAA----TAAGATATGAA-
GACCGTCAAACAAGAAATTTT------
GCACCCGGGGGTGAGAGCTGTCATGAAGGTGTCCTGGGCTACCCAGCTATCCAGTCAGGAAAACATTATTGGGAA
GTAGATGTGTCTGGAAAAGGTGCCTGGGTTCTGGGATTAAGT--------GATGGAAGCTACCT-----------------
CTTCAA-TCCGATATTTCGTGCAAATGCTGAAAGA------------CACCTAGATCCCTTA------
TTCCGCTTGGGTATTAGTAA--T------------GATTCACGTTATCAACCTAGATATGGCTTCTGGGTTATAGGGCTGT----
-----GGAATAAGTGCGTGTATAATGCCTTT--
GAGGAGTGTGCTTTCACAGGCAAGCCCAGTGTCTTGACTCTATCTCTGATGGTTCCTCCCTGTCGTGTTGGCATTTT
CCTTGACTATGCAGCTGGCACCCTCTCGTTTTACAATATTTCCCACCATGGGACTCTCATCTATAGATTCTGTGCAAA
TT-----------------------------CCTTTC---------CTGATAGGGTTTTCCC-----------
ATATTTTAATCCTATGGGATGTTCAGAG---CCAATGACAGTG----------TGCTGGTCA--------GACTCTTAA-------------
----

```
>Rattus_norvegicus_Trim5

----
CCTGATCTGCAAGGCATGCTGCAAGTGTTACAAGAGGTCACAGAGGCCCAACGCTACTGGGTTCAAGTGACGCTG
GTTGAAA---GCAACAATCCAAACATTTTCATTACCGCCGACAAAAGACAGA----TACGATATGAA-
GACCACCAAGCAAGACATTTT------
GCCCGTCCGACTGAAAACTGTCATGCAGGTGTCCTGGGATACCCAGCTATCCAATCAGGAAAACACTACTGGGAA
GTAGATGTGTCTGGAAAAGGTTCTTGGGTTCTGGGATTAAGT--------GATGGAAGCTACCT-----------------
CTTTAA-TCCAATATTTCGTTCAAATGCAGAAAGA------------
CCCCCAAACCCCCCCTATGATTCTCGTTTGAGTCTTAGTAA--T------------
GATTCACATTATCAACCTAAATATGGCTTCTGGGTTATAGGGCTGT---------
GGGGAAATTCTGTGTATAATGCTTTT--
GAGGAGTGTACGTTCACAGGCAAGCCCAGTGTGTTGACCCTCTCTCTGATGGTCCGACCCTGTCGTGTCGGTATTT
TCCTCGACTGTGCAGCTGGCACCCTCTCGTTTTACAATATTTCCAACCATGGCACTCTTATCTACAGATTCTGTGCAG
GTT----------------------------CCTTTC---------CTGATAGGGTTTTTCC-----------
ATATTTTAACCCCATGGGAAGTTCAGAG---CCATTGACAATA----------TGCTGGCCA--------GACTCTTAA-------------
----------

>Mus_musculus_Trim5

----
CCTGACCTGCAAGGCATGCTGCAAGTGCTGCAAGAGGTCACAGAGGCTCAACGCTACTGGGTTCAGGTGACCCTG
GTTCAAA---ATAACCATCCAAACATTGCCATTACTGACGACAAAAGACAAG----TACGATATGAA-
GACCACCAAGCAAGTAATCTT------
ATACACGAGTGTGAAAACTCTCATGAAGGCGTCCTGGGACATCCAGCTATCCAATCTGGAAAACATTACTGGGAA
GTAGATGTGTCTGGAAAAGGTGCTTGGGTTCTGGGGTTAAGT--------GATGGAAGCTACCT-----------------
CTTCTG-TCCAATATTTCATTCAAATGCTGAAAG------------------AAATCGTTTA------
TTTTGTAGGGGGATTAAGAA--T------------GATTCACATTATCAACCAAAATATGGCTTCTGGGTTATAGGGCTGT---
------GGAAAAAGTCTGTGTATAATGCTTTT--
GAGGAGTGTCCTTTCACAGGCAAGCCCAGTGTCTTGACCCTGTCTCTGATGGTCCCGCCCTGTCGTGTTGGTGTTTT
CCTTGACTATGCAGCTGGGACTCTCTCATTTTACAATATTTCCAACAATGGGACTCTTATCTACAGATTCTGCACAGC
TT----------------------------CCTTTC---------CTGATAGGGTTTTTCC-----------
ATATTTTAATCCCACGGGAAGTTCAGAG---CCGATGACAATT----------TGCTGGCCA--------GACTCTTAA-------------
----------

>Mus_musculus_Trim12c

----
CCTGACCTGCAAGGCATGCTGCAAGTGCTGCAAGAGGTCACAGAGGCTCAACGCTACTGGGTTCAGGTGACGCTG
GTTCAAA---ACAACAATCCAAACATTGCCATTACTGATGACAAAAGGCAAA----TACGATATGAA-
GACCTCCAAGCAAGGAATCTT------
GAACATGGCTGTGAAAACTCTCATGAAGGTGTCCTGGGACACCCAGTTATCCAATCAGGAAAACATTACTGGGAA
GTAGATGTGTCTGGAAAAGGTGCTTGGGTTCTGGGGTTAAGT--------GATGGAAGCTACCT-----------------
CTTCAA-TCCAATATTTCGTTCAAATGCTGAAAG------------------AAACCCTTTA------
TTTCGCAGGGGGATTAAGAA--T------------GATTCACATTATCAACCAAAATATGGCTTCTGGGTTATAGGGCTGT---
------GGAAAAAGTCTGTGTATAATGCTTTT--
GAGGAGTGTCCTTTCACAGGCAAGCCCAGTGTCTTGACCCTGTCTCTGATGGTCCCGCCCTGTCGTGTTGGTGTTTT
```

CCTTGACTATGCAGCTGGGACTCTCTCATTTTACAATATTTCCAACAATGGGACTCTTATCTACAGATTCTGTACAGC
TT-----------------------------CCTTTC---------CTGATAGGGTTTTTCC-----------
ATATTTTAATCCCATGGGAAGTTCAGAG---CCCATGACAATT----------TGCTGGCCA--------GACTCTTAA-------------
----

>Homo_sapiens_Trim22

CCAGATCTGAGTGGGATGCTGCAAGTTCTTAAAGAGCTGACAGATGTCCAGTACTACTGGGTGGACGTGATGCTG
AATCCAGGCAGTGCCACTTCGAATGTTGCTATTTCTGTGGATCAGAGACAAG----TGAAAACTGTA-
CGCACCTGCACATTTAAGAAT------
TCAAATCCATGTGATTTTTCTGCTTTTGGTGTCTTCGGCTGCCAATATTTCTCTTCGGGGAAATATTACTGGGAAGT
AGATGTGTCTGGAAAGATTGCCTGGATCCTGGGCGTACAC--------
AGTAAAATAAGTAGTCTGAATAAAAGGAAGAGCTCTGGGTTTGCTTTTGATCCAAGTGTAAA-------------------------
--------------TTATTCAAAAGTT--------------------
TACTCCAGATATAGACCTCAATATGGCTACTGGGTTATAGGATTAC---------
AGAATACATGTGAATATAATGCTTTT--GAGGA---
CTCCTCCTCTTCTGATCCCAAGGTTTTGACTCTCTTTATGGCTGTGCCTCCCTGTCGTATTGGGGTTTTCCTAGACTAT
GAGGCAGGCATTGTCTCATTTTTCAATGTCACAAACCACGGAGCACTCATCTACAAGTTCTCTGGATGTC------------
-----------------GCTTTT---------CTCGACCTGCTTATCC-----------GTATTTCAATCCTTGGAACTGCCTAGTC---
CCCATGACTGTG----------TGCCCACCG--------AGCTCCTGA----------------------

>Jaculus_jaculus_Trim34

CCCGACCTGAGTGGCATGCTGCAAATGTTTCGAGAGCTAACAGATGCCCGGAGCTACTGGGTGGACATCACATTG
AGTCAGGACAACCTAAATCTGGATCTCATACTTTCTGAAGACCAGAGACAAG----TGACATGCGTG-
CCAATATGGCCATATAAC------------------------
TGTTGTAATTATGGTATCTTAGGGTCCCAGTACTTCTCCTCAGGGAAACATTACTGGGAAATAGACGTGTCTAAGA
AGACTGCCTGGATCATCGGAGTATAC--------TGTAGGAAACG----CT--------------CTGCAA-
AGTCTTGTGTTAGACAAGGCAAAAGACT-------------------------------------CCCAAATGTT--------------------
TACTCCAGAGGCAGACCCCAAGATGACTACTGGGTTCTGTGGCTAC---------
AGAATGAAACCAAGTTCGGTGCCTTC--GCGAA---
CTCTGTTACTTTCAATCCTATAGTTGTAACCCTCTATATGGCGATCCCTCCCCAACGGGTGGGGGTTTTCCTCAACTA
TGAAGCAGGCACTGTCTCCTTTTTCAATATCACAAACCATGGGTCCCTTATCTACAAGTTCTCTAATTGTG------------
-----------------GCTTTC---------CTCAGCCTGTGTATCC-----------GTATTTCAGTCCCTGGAACTGCCCGGCC---
CCCATGACCCTG----------TGTCCACCG--------AGCTCTTGA----------------------

>Meriones_unguiculatus_Trim34

CCCGATCTGAGCGACATGCTACAGAAGTTTAGAGAGCTGACAGCTGTCCGGAGTTACTGGGCGGACTTCACATTC
AATCCAGAAAACCTCAATTTGAATCTTACTCTTTCAGAAGATCACAGACAGT----TGACTTCTGTG-
CCCATTTGGCCA------------------------
TTTAAGTGTTATAATAATGGTATCTTGGGCTCCAAATGTTTCTCCTCAGGAAAACATTACTGGGAAGTGGATGTGTC
TCAGAAGAATGCCTGGATCCTGGGAGTGTAT--------GCTCGGAAGCGAAA-----------------TTTAAAGTTTGA-----
TGTTAGAAGAGGCAAAAG-----------------------------------TCAGCCAAGTGTT--------------------

CACCACAGATACAAACCTCAGAATGGATACTGGGTTGTAGGGTTAC---------
AGGATGGATCGAAGTATAGTATCTTT--GAGGA---
TTCTTGCAACGGTGACCCTACAGTTCTGACCCCCTTCGTGGCTGTCCCCCTCCATCGGGTCGGGGTTTTCCTCGACT
GTGAAGAGGGCATAGTGTCCTTCTTCAATGTCACAAACCATGGGTCACTCATCTACCAGTTCTCTCAATGCT---------
--------------------GCTTTT---------CCCAACCTGCATATCC-----------ATACTTCAATCCTTGGGACTGTCCAGCC---
CCCATGACGCTG----------TGTCTTCTC--------AGCTCCTGA-----------------------

>Rattus_norvegicus_Trim34

CCTGATCTAAGTGGCATGCTACAAAAGTTTAGAGAGCTAACAGCTGTCCGGGCCTACTGGGCGGACTTCACGTTTA
ATCCAGAAAACCTAAATTTGAATCTCATTCTTTCAGAAGATCATAGACAAG----TGACATCTGTG-CCCATTTGGCCC-
----
TTTAAGTGTTGTAATAATGGTATCTTGGGCTCCAAATGTTTCTTGTCAGGAAAACATTACTGGGAAGTGGATGTGT
CTGAAAAGAATGCCTGGACCCTGGGAGTTTAC--------TCTAGAAAACGTAC-----------------TTTGAA-GTTTG-
ATGTTAGACGATGCAGCAAAAG-----------------------------------TCAGCCAAATGGT--------------------
TACCACAGATACAAACCTCAGAATGGCTACTGGGTTATAGGGTTAC---------
AGGATGGATCAAAGTATAGTATCTTT--GAGGA---
TTCTTCCAACTGTGGCCCTACAGTTCTGAACCCCTTTGTGGCCACCCCTCTCCATCGGATTGGGGTTTTCCTTGACTG
TGAAGAGGGCATAGTGTCCTTCTTCAATGTCACAAACCACGGGTCACTCATTTACAAGTTTACCCAATGCC-----------
------------------GCTTTT---------CCCAACCTGCCTATCC-----------ATACTTCAATCCTTGGGACTGCCCAGCC---
CCCATGACCCTC----------TGTCCTCGG--------AACGCCTGA-----------------------

>Mus_musculus_Trim34a

CCTGATCTGAGTGGCATGCTACAAAAGTTTAGAGAGCTAACAGCTGTCCGGGCCTACTGGGACAACTTCACATTTA
ATCCAGAAAACCTAAATTTGAATCTTATACTTTCAGAAGACCACAGACAAG----TGACATCTGTG-
TCCATTTGGCCC------------------------
TTTAAGTGTTGTAATAACGGTATCTTGGGCTCCAAATGTTTCTCCTCAGGAAAACATTACTGGGAAGTGGATGTGT
CTGAAAAGAAGGCCTGGACCCTGGGAGTTTAC--------ACTAGAAAACGAAC-----------------TTTAAGGTTTGA-----
TGTTAGACAACGCAAAGG-----------------------------------TCAGCCAAATGGT--------------------
TACCACAGATACAAACCACAGAATGGCTACTGGGTTATAGGGTTAC---------
AGCATGGATCGAAGTATAGTATCTTT--GAGGA---
TTCTTCCAACTGTGACCCTACTGTTCTGAACCCCTTTGTGGCCACCCCTCTCCATCGGGTTGGGGTTTTTCTTGACTG
TGAAGAGGGCACAGTATCCTTCCTCAATGTCACCAACCATGGATCACTCATTTACAAGTTCTCCCAATGCT-----------
------------------GCTTTT---------CCCAACCTGCCTATCC-----------ATACTTCAATCCTTGGGACTGTCCAGCC---
CCCATGACCCTG----------TGTCCTCTG--------AACTCCTGA-----------------------

>Mus_musculus_Trim34b

CCTGATCTGAGTGGCATGCTACAAAAGTTCAGAGAGCTATCAGCTGTCCGGGCCTACTGGGACAACTTCACATTTA
ATCCAGAAAACCTAAATTTGAATCTTATACTTTCAGAAGACCACAGACAAG----TGACATCTGTG-
TCCATTTGGCCC------------------------
TTTAAGTGTTGTAATAATGGTATTTTGGGCTCCAAATGTTTCTCCTCAGGAAAACATTACTGGGAAGTGGATGTGTC
TGAAAAGAATGCCTGGACCCTGGGAGTTTAC--------ACTAGAAAACGAAC-----------------TTTAAGGTTTGA-----

TGTTAGACAACGCAAAGG---------------------------------TCAGCCAAATGGT--------------------
TACCACAGATACAAACCACAGAATGGCTACTGGGTTATAGGGTTAC---------
AGCATGGATCGAAGTATAGTATCTTT--GAGGA---
TTCTTCCAACTGTGACCCTACTGTTCTGAACCCCTTTGTGGCCACCCCTCTCCATCGGGTTGGGATTTTCCTTGACTG
TGAAGAGGGCACAGTGTCCTTCCTCAATGTCACCAACCATGGATCACTCATTTACAAGTTCTCCCAATGCT-----------
------------------GCTTTT---------CCCAACCTGCCTATCC-----------ATACTTCAATCCTTGGGACTGTCCAGCC---
CCCATGACCCTG----------TGTCCTCTG--------AACTCCTGA-----------------------

>Cricetulus_griseus_Trim34

CCTGATCTGAGCGACATGCTCCAAAAGTTTAGAGAGCTAACAGCCGTCCGGGGCTACTGGGTGGACTTCACATTTA
ACCCAGAAAACCTCAATTTGAATCTGATTCTTTCAGAAGACCACAGACAAG----TGGCATCTGTG-
CCCATTTGGCCA------------------------
TTTAAGTGTTATAATAATAGTATCTTGGGCTCCAAATGTTTCTCCTCAGGAAAACATTACTGGGAAGTGGATGTGTC
TAAGAAGAGTGCCTGGATCCTGGGAGTTTAC--------GCTAGAAGACGTAC-----------------TTTAAAGTTTGA-----
TGTTAGACGAGGCAAAAA---------------------------------TCAGCCAAATGTT--------------------
CACCACAGATACAAACCTCAGAATGGCTACTGGGTTATAGGGTTGC---------
AGGGTGGATCAAAGTATAGTATTTTT--GAGGA---
TTCTTCCAACTGTGATCCTACTGTTCTGACCCCCTTTGTGGCTGTCCCTCTCCATCGGGTTGGGGTTTTCCTTGACTG
TGAAGAAGGCATGGTGTCCTTCTTCAATGTCACGAACCATGGGTCACTGATTTACACGTTCTCTCAGTGCT-----------
------------------GCTTTT---------CCCAGCCTGCTTATCC-----------ATACTTCAATCCTTGGGACTGCCCAGCC---
CCCATGACCCTG----------TGTCTTCAG--------AACTCCTGA-----------------------

>Microtus_ochrogaster_Trim34

CCTGATCTGAGCGACATGCTCCGACAGTTTAGAGAGTTAACAGCTGTCCGCGGCTATTGGGCGGATTTCACATTTA
ATCCAGAAAACCTAAATTTGAATCTTATTCTTTCAGAAGATCACAGACAAG----TGACATCTGTG-CCCATTTGGCCA-
----
TTTAAGTGTTATAATAATGGTATCTTGGGCTCCAAATGTTTCTCCTCAGGGAAACATTACTGGGAAGTGGATGTGT
CTAAGAAGAATGCCTGGACACTGGGAGTTTAC--------GTTAGAAAACGTAC-----------------TTTAAAGTTTGA-----
TGTCAGGAGAGGCAAAAA---------------------------------CCAGCCAAATGTT--------------------
TGCCACAGATACAAACCTCAGAATGGCTACTGGGTTATAGGGTTAC---------
AGGATGGATCAAAGTATAGTATCTTT--GAGGA---
TTCTTCCAACTGTGACCCTACCATTCTGACCCCCTTTGTGGCTGCCCCTCTCCATCGGGTTGGGGTTTTCCTTGACTG
TGAAGAGGGCATAGTGTCCTTCTTCAATGTCACAAACCATGGGTCACTCATTTACACATTCTTTAAATGCT-----------
------------------GCTTTT---------CCCACCCTGCTTATCC-----------ATACTTTAATCCTTGGGATTGCCCAGCT---
CCCATGACCCTG----------TGTCCTGTG--------AACTCCTGA-----------------------

>Peromyscus_maniculatus_Trim34

CCTGATCTGAGAGACATGCTCTGCAAGTTCAGAAAGCTAACAGCTGTCCGTGGCTACTGGGCGGACTTCACATTTA
ATCCAGAAAACCTAAATTTGAATCTTATTCTTTCAGAAGATCACAGACAAG----TGACATCTGTG-
CCCATTTGGCCATTTAAG------------------------
TGTTATAATAATGGTGTCTTGGGCTCCAAATGTTTCTTCTCAGGAAAACATTACTGGGAAGTGGATGTGTCCAAGA

AGAGTGCCTGGACTCTGGGAGTTTAT--------GCTAGAAAACGTAAAAA--------------TTTAAA-GTTTG-
ATGTTAGACGAGGCAAAAA--------------------------------------TCAGCCAAATGTT--------------------
TACCACAGATACAAACCTCAGAATGGCTACTGGGTTATAGGGTTAC---------
AGGATGGATCAAAGTATAGTATCTTT--GAGGA---
TTCTTCCAACTGTGACCCTACAGTTCTGACCCCCTTTGTGGCTGTCCCTCTCCATCGGGTTGGGGTTTTCCTTGACTG
TGAAGAGGGCATAGTGTCCTTCTTTAATGTCACAAACCACGGGTCACCCATTTACACATTCTCTCAATGCT-----------
------------------GCTTTT---------CCAAACCTGTGTATCC-----------ATACTTCAATCCTTGGGACTGCCCAGCC---
CCCATGACCCTG----------TGTCTTCTG--------AACTCCTGA-----------------------

Supplementary Table S1. Annotation of the rodent *Trim* clade of genes found in the *Trim5* cluster.

Species	NCBI assigned gene symbol	Gene clade based on phylogenetic analysis	RefSeq Accession No. for mRNA sequence used in phylogenetic analysis	Approximate cluster size (bp)
Mus musculus	Trim6	Trim6	NM_001013616	320,000
	Trim34a	Trim34	NM_030684	
	Trim5	Trim12	NM_001310602	
	Trim12a	Trim12	NM_023835	
	Trim34b	Trim34	NM_001243916	
	Trim12c	Trim12	NM_001146007	
	Gm38525	Trim30	XM_017312449	
	Trim30b	Trim30	NM_175648	
	Trim30c	Trim30	XR_869856	
	Trim30a	Trim30	NM_009099	
	Trim30d	Trim30	NM_199146	
	Trim30e-ps1	Trim30	NR_033673	
Rattus norvegicus	Trim6	Trim6	NM_001170461	131,000
	Trim34	Trim34	NM_001276491	
	Trim5	Trim12	NM_001014023	
	LOC102555738	Trim30	Not annotated	
	Trim30c	Trim30	XM_006229915	
	Trim30	Trim30	XM_006229917	
Meriones unguiculatus	LOC110558325	Trim6	XM_021653168	73,000
		Trim34		
	LOC110558330	Trim12	XM_021653174	
	LOC110558329	Trim30	XM_021653172	
Cricetulus griseus	LOC100752691	Trim30	XM_027407899	317,000
	LOC113830641	Trim30	XM_027407906	
	LOC100763830	Trim6	XM_027407915	
		Trim34	XM_027407917	
	LOC100762680	Trim12	XM_027407921	
	LOC100762389	Trim30	XM_027407922	
	LOC100772719	Trim30	XM_027407924	
	LOC100771630	Trim30	XM_027407927	
Microtus ochrogaster	LOC101994551	Trim30	XM_005370243	384,000
	LOC101997233	Trim30	XM_005370165	
	Trim6	Trim6	XM_026777564	
	LOC101999235	Trim34	XM_026777562	
	LOC101999497	Trim12	XM_013354827	
	LOC101999766	Trim30	Not annotated	

<table>
<tr><td rowspan="4"></td><td>LOC102000031</td><td>Trim30</td><td>XM_026777575</td><td rowspan="4"></td></tr>
<tr><td>LOC102000314</td><td>Trim30</td><td>XM_013354828</td></tr>
<tr><td>LOC102000592</td><td>Trim30</td><td>XM_005370176</td></tr>
<tr><td>LOC102000872</td><td>Trim30</td><td>XM_005370178</td></tr>
<tr><td rowspan="7">Peromyscus maniculatus</td><td>Trim6</td><td>Trim6</td><td>XM_006979023</td><td rowspan="7">142,000</td></tr>
<tr><td>LOC102909699</td><td>Trim34</td><td>XM_015997716</td></tr>
<tr><td>LOC102910020</td><td>Trim12</td><td>XM_015997715</td></tr>
<tr><td>LOC102905163</td><td>Trim30</td><td>XM_015997704</td></tr>
<tr><td rowspan="3">LOC102910346</td><td>Trim30</td><td>XM_015997722</td></tr>
<tr><td>Trim30</td><td>XM_015997719</td></tr>
<tr><td>Trim30</td><td>XM_006979028</td></tr>
<tr><td rowspan="3">Jaculus Jaculus</td><td>Trim6</td><td>Trim6</td><td>XM_012950142</td><td rowspan="3">46,500</td></tr>
<tr><td>LOC101594518</td><td>Trim34</td><td>XM_012950198</td></tr>
<tr><td>LOC101611632</td><td>Trim5</td><td>XM_012950138</td></tr>
<tr><td rowspan="4">Dipodomys ordii</td><td>Trim6</td><td>Trim6</td><td>XM_013035606</td><td rowspan="4">145,000</td></tr>
<tr><td>LOC106000347</td><td>Trim5</td><td>XM_013035600</td></tr>
<tr><td>LOC106000349</td><td>Trim5</td><td>XM_013035603</td></tr>
<tr><td>LOC106000348</td><td>Trim5</td><td>XM_013035602</td></tr>
<tr><td rowspan="3">Castor Canadensis</td><td>Trim6</td><td>Trim6</td><td>XM_020165122</td><td rowspan="3">62,500</td></tr>
<tr><td>Trim34</td><td>Trim34</td><td>XM_020165121</td></tr>
<tr><td>Not annotated</td><td>Trim5</td><td>Not annotated</td></tr>
<tr><td rowspan="4">Marmota marmota</td><td rowspan="2">Trim6</td><td>Trim6</td><td>XM_015477548</td><td rowspan="4">162,000</td></tr>
<tr><td>Trim34</td><td>XM_015477543</td></tr>
<tr><td>LOC107135589</td><td>Trim5</td><td>XM_015477550</td></tr>
<tr><td>LOC107157382</td><td>Trim5</td><td>XM_015503770</td></tr>
<tr><td rowspan="4">Marmota flaviventris</td><td rowspan="2">Trim6</td><td>Trim6</td><td>XM_027942600</td><td rowspan="4">143,000</td></tr>
<tr><td>Trim34</td><td>XM_027942597</td></tr>
<tr><td>Trim5</td><td>Trim5</td><td>XM_027942535</td></tr>
<tr><td>Trim5</td><td>Trim5</td><td>XM_027942737</td></tr>
<tr><td rowspan="8">Cavia porcellus</td><td>Trim6</td><td>Trim6</td><td>XM_023563098</td><td rowspan="8">202,000</td></tr>
<tr><td>LOC100716056</td><td>Trim5</td><td>XM_023563103</td></tr>
<tr><td>LOC100715776</td><td>Trim5</td><td>XM_013147645</td></tr>
<tr><td>Not annotated</td><td>Trim5</td><td>Not annotated</td></tr>
<tr><td>LOC100715495</td><td>Trim5</td><td>XM_013147641</td></tr>
<tr><td>LOC100715212</td><td>Trim34</td><td>XM_003465340</td></tr>
<tr><td>LOC100714924</td><td>Trim5</td><td>XM_013147639</td></tr>
<tr><td>Not annotated</td><td>Trim5</td><td>Not annotated</td></tr>
<tr><td rowspan="9">Octodon degus</td><td>LOC105743655</td><td>Trim6</td><td>XM_023702137</td><td rowspan="9">354,000</td></tr>
<tr><td>Not annotated</td><td>Trim5</td><td>Not annotated</td></tr>
<tr><td>LOC101569936</td><td>Trim34</td><td>XM_012517106</td></tr>
<tr><td>LOC105743656</td><td>Trim5</td><td>XM_023702118</td></tr>
<tr><td>LOC101570337</td><td>Trim5</td><td>XM_023702120</td></tr>
<tr><td>LOC101570739</td><td>Trim5</td><td>XM_012517109</td></tr>
<tr><td>LOC101571138</td><td>Trim5</td><td>XM_023702063</td></tr>
<tr><td>LOC101581987</td><td>Trim5</td><td>XM_004642012</td></tr>
<tr><td>Not annotated</td><td>Trim5</td><td>Not annotated</td></tr>
<tr><td rowspan="2">Chinchilla lanigera</td><td>Trim6</td><td>Trim6</td><td>XM_005380095</td><td rowspan="2">116,000</td></tr>
<tr><td>LOC102007986</td><td>Trim5</td><td>XM_013512002</td></tr>
</table>

	LOC102008935	Trim5	XM_013512006	
	LOC106147995	Trim5	XM_013511731	
	LOC102009273	Trim34	XM_005380098	
	LOC102007543	Trim5	XM_013511732	
	LOC106147996	Trim5	Not annotated	
Heterocephalus glaber	Trim6	Trim6	XM_004863365	97,000
	Trim34	Trim34	XM_013065032	
	LOC101716219	Trim5	XM_013064937	

Supplementary Table S2. Primers used in this study

Primer description	Sequence
SPRY exon 8 **(1F)**	5’ ACT TGT CAG TAC AAA CCA ACA TGG 3’
SPRY exon 8 **(2F)**	5’ TAA CTG TTT CTG CCT TTG CTC TTT 3’
CypA internal **(1R)**	5’ GCT GTC TTT GGA ACT TTG TCT GC 3’
CypA internal **(2R)**	5’ CCA GCA GCC GTG ATG TCG AAG 3’
CypA intron forward **(3F)**	5’ ACC TGG ATG CCC TTC ATA GAG 3’
CypA intron reverse **(3R)**	5’ GAC TGA TGC ATA CTC TGC CAG CAT 3’
CypA DNA forward for sequencing **(4F)**	5’ GGT TTT GAA TGA GGT AGT TGG T 3’
CypA DNA reverse for sequencing **(4R)**	5’ AGA GAC CCT GAT GCT CTG GAC A 3’
Peromyscus TrimCyp forward for sequencing **(5F)**	5’ GGT TGG TGT CTG AAG GTG TGA 3’
Peromyscus TrimCyp reverse for sequencing **(5R)**	5’ AGC GAA GGT TAC AGG GTA CTG 3’
omTRIMCyp forward **(6F)**	5’ GCC ACC ATG GCT TCC AGA ATC CTG 3’
omTRIMCyp reverse **(6R)**	5’ AAG TTG TCC ACA GTC AGC AAT GGT GA 3’
peroTrimCyp forward **(7F)**	5’ GCCACCATGGCCTCATCAGTCCTGGT 3’
peroTrimCyp reverse **(7R)**	5’ GAGTTGTCCACAGTCGGAAAT 3’
peroTrimSPRY cDNA forward **(8F)**	5’ GTG TCA GGA GTC AGA ACA GAC AGA C 3’
peroTrimSPRY cDNA reverse **(8R)**	5’ GTA AAA AAA TCC ATG TTG GTT TGT ACT GAC AAG TG 3’
peroTrimSPRY CDS reverse **(9R)**	5’ GGA GGG TGG CCC ACA TAC TGT CAT T 3’
